# Individual‐level interventions for reducing occupational stress in healthcare workers

**DOI:** 10.1002/14651858.CD002892.pub6

**Published:** 2023-05-12

**Authors:** Sietske J Tamminga, Lima M Emal, Julitta S Boschman, Alice Levasseur, Anilkrishna Thota, Jani H Ruotsalainen, Roosmarijn MC Schelvis, Karen Nieuwenhuijsen, Henk F Molen

**Affiliations:** Public and Occupational HealthAmsterdam UMC location University of AmsterdamAmsterdamNetherlands; Societal Participation & HealthAmsterdam Public Health Research InstituteAmsterdamNetherlands; Faculté des sciences de l'éducationUniversité LavalQuébecCanada; Independent ConsultantTorontoCanada; Institute of Public Health and Clinical NutritionUniversity of Eastern FinlandKuopioFinland; Body@Work, Research Center on Work, Health and TechnologyTNO/VUmcAmsterdamNetherlands

**Keywords:** Humans, Anxiety, Anxiety/diagnosis, Emotions, Health Personnel, Health Personnel/psychology, Occupational Stress, Occupational Stress/prevention & control, Psychotherapy, Psychotherapy/methods

## Abstract

**Background:**

Healthcare workers can suffer from work‐related stress as a result of an imbalance of demands, skills and social support at work. This may lead to stress, burnout and psychosomatic problems, and deterioration of service provision. This is an update of a Cochrane Review that was last updated in 2015, which has been split into this review and a review on organisational‐level interventions.

**Objectives:**

To evaluate the effectiveness of stress‐reduction interventions targeting individual healthcare workers compared to no intervention, wait list, placebo, no stress‐reduction intervention or another type of stress‐reduction intervention in reducing stress symptoms.

**Search methods:**

We used the previous version of the review as one source of studies (search date: November 2013). We searched the Cochrane Central Register of Controlled Trials (CENTRAL), MEDLINE, Embase, PsycINFO, CINAHL, Web of Science and a trials register from 2013 up to February 2022.

**Selection criteria:**

We included randomised controlled trials (RCT) evaluating the effectiveness of stress interventions directed at healthcare workers. We included only interventions targeted at individual healthcare workers aimed at reducing stress symptoms.

**Data collection and analysis:**

Review authors independently selected trials for inclusion, assessed risk of bias and extracted data. We used standard methodological procedures expected by Cochrane. We categorised interventions into ones that:

1. focus one’s attention **on** the (modification of the) experience of stress (thoughts, feelings, behaviour);

2. focus one’s attention **away** from the experience of stress by various means of psychological disengagement (e.g. relaxing, exercise);

3. alter work‐related risk factors on an individual level; and ones that

4. combine two or more of the above.

The crucial outcome measure was stress symptoms measured with various self‐reported questionnaires such as the Maslach Burnout Inventory (MBI), measured at short term (up to and including three months after the intervention ended), medium term (> 3 to 12 months after the intervention ended), and long term follow‐up (> 12 months after the intervention ended).

**Main results:**

This is the second update of the original Cochrane Review published in 2006, Issue 4. This review update includes 89 new studies, bringing the total number of studies in the current review to 117 with a total of 11,119 participants randomised.

The number of participants per study arm was ≥ 50 in 32 studies. The most important risk of bias was the lack of blinding of participants.

**Focus on the experience of stress versus no intervention/wait list/placebo/no stress‐reduction intervention**

Fifty‐two studies studied an intervention in which one's focus is on the experience of stress. Overall, such interventions may result in a reduction in stress symptoms in the short term (standardised mean difference (SMD) ‐0.37, 95% confidence interval (CI) ‐0.52 to ‐0.23; 41 RCTs; 3645 participants; low‐certainty evidence) and medium term (SMD ‐0.43, 95% CI ‐0.71 to ‐0.14; 19 RCTs; 1851 participants; low‐certainty evidence). The SMD of the short‐term result translates back to 4.6 points fewer on the MBI‐emotional exhaustion scale (MBI‐EE, a scale from 0 to 54). The evidence is very uncertain (one RCT; 68 participants, very low‐certainty evidence) about the long‐term effect on stress symptoms of focusing one's attention on the experience of stress.

**Focus away from the experience of stress versus no intervention/wait list/placebo/no stress‐reduction intervention **

Forty‐two studies studied an intervention in which one's focus is away from the experience of stress. Overall, such interventions may result in a reduction in stress symptoms in the short term (SMD ‐0.55, 95 CI ‐0.70 to ‐0.40; 35 RCTs; 2366 participants; low‐certainty evidence) and medium term (SMD ‐0.41 95% CI ‐0.79 to ‐0.03; 6 RCTs; 427 participants; low‐certainty evidence). The SMD on the short term translates back to 6.8 fewer points on the MBI‐EE. No studies reported the long‐term effect.

**Focus on work‐related, individual‐level factors versus no intervention/no stress‐reduction intervention**

Seven studies studied an intervention in which the focus is on altering work‐related factors. The evidence is very uncertain about the short‐term effects (no pooled effect estimate; three RCTs; 87 participants; very low‐certainty evidence) and medium‐term effects and long‐term effects (no pooled effect estimate; two RCTs; 152 participants, and one RCT; 161 participants, very low‐certainty evidence) of this type of stress management intervention.

**A combination of individual‐level interventions versus no intervention/wait list/no stress‐reduction intervention**

Seventeen studies studied a combination of interventions. In the short‐term, this type of intervention may result in a reduction in stress symptoms (SMD ‐0.67 95%, CI ‐0.95 to ‐0.39; 15 RCTs; 1003 participants; low‐certainty evidence). The SMD translates back to 8.2 fewer points on the MBI‐EE. On the medium term, a combination of individual‐level interventions may result in a reduction in stress symptoms, but the evidence does not exclude no effect (SMD ‐0.48, 95% CI ‐0.95 to 0.00; 6 RCTs; 574 participants; low‐certainty evidence). The evidence is very uncertain about the long term effects of a combination of interventions on stress symptoms (one RCT, 88 participants; very low‐certainty evidence).

**Focus on stress versus other intervention type **

Three studies compared focusing on stress versus focusing away from stress and one study a combination of interventions versus focusing on stress. The evidence is very uncertain about which type of intervention is better or if their effect is similar.

**Authors' conclusions:**

Our review shows that there may be an effect on stress reduction in healthcare workers from individual‐level stress interventions, whether they focus one's attention on or away from the experience of stress. This effect may last up to a year after the end of the intervention. A combination of interventions may be beneficial as well, at least in the short term. Long‐term effects of individual‐level stress management interventions remain unknown. The same applies for interventions on (individual‐level) work‐related risk factors.

The bias assessment of the studies in this review showed the need for methodologically better‐designed and executed studies, as nearly all studies suffered from poor reporting of the randomisation procedures, lack of blinding of participants and lack of trial registration. Better‐designed trials with larger sample sizes are required to increase the certainty of the evidence. Last, there is a need for more studies on interventions which focus on work‐related risk factors.

## Plain language summary

**The effect of individual‐level interventions for reducing stress in healthcare workers**

**Key messages**

‐ Individual‐level interventions in which one’s attention is **on** the experience of stress (like focusing on thoughts, feelings, behaviour) or **away** from the experience of stress (like exercising, relaxing) may reduce stress among healthcare workers up to one year after the intervention.

‐ A combination of individual‐level interventions may reduce stress up to a couple of months after the intervention.

‐ We do not know if interventions that focus on work‐related risk factors on an individual level have any effect on stress.

**What is stress?**

There is currently no clear definition of (work‐related) stress. This review is about healthcare workers with low levels of stress to moderate distress and burnout, which might lead to depression and anxiety but does not have to. People with stress can experience physical symptoms like headaches, muscle tension or pain, but also mental symptoms, like impaired concentration. They can also have behavioural problems (like conflicts with other people) and emotional problems (like emotional instability).

**What can be done about stress among healthcare workers?**

Stress among healthcare workers can be tackled at an organisational level, but also at an individual level. Stress management interventions at the individual‐level aim to:

‐ focus one’s attention **on** the experience of stress (thoughts, feelings, behaviour), for example by cognitive‐behavioural therapy or coping skills training;

‐ focus one’s attention **away** from the experience of stress, for example by yoga, Tai Chi, drawing, or acupuncture;

‐ alter work‐related risk factors on an individual level, such as alterations in work demands.

**What did we want to find out?**

We wanted to find out if various types of individual‐level stress management interventions are better than no intervention (or another intervention) to reduce stress among healthcare workers currently working as such.

**What did we do**

We searched for studies that looked at stress management interventions in healthcare workers and reported on stress symptoms. The healthcare workforce comprises a wide variety of professions and occupations who provide some type of healthcare service, including direct care practitioners and allied professionals.

We compared and summarised the results of the studies and rated our confidence in the evidence, based on factors such as study methods and study size.

**What did we find?**

We found a total of 117 studies that involved a total of 11,119 healthcare workers. Most studies followed their participants up to three months and some up to 12 months, but only few longer than a year.

We found that there may be an effect on stress reduction in healthcare workers from stress management interventions, whether they focus one's attention on or away from the experience of stress. This effect may last up to a year after the end of the intervention. A combination of interventions may be beneficial as well, at least in the short term. The long‐term effects of stress management interventions, longer than a year after the intervention has ended, remain unknown. The same applies for interventions on (individual‐level) work‐related risk factors.

**What are the limitations of the evidence?**

The estimates of the effects of individual‐level stress management interventions may be biassed because of a lack of blinding of the participants in the included studies. Furthermore, many studies were relatively small. Taken together, our confidence in the effects we found is reduced.

**How up to date is this evidence?**

The evidence is up‐to‐date to February 2022.

## Summary of findings

**Summary of findings 1:** An intervention in which one's attention is on the experience of stress (feelings, thoughts, behavior) compared to no intervention/wait list/placebo/no stress‐reduction intervention for stress reduction in healthcare workers

**An intervention in which one's attention is on the experience of stress compared to no intervention/wait list/placebo/no stress‐reduction intervention for stress reduction in healthcare workers**				
**Patient or population:** healthcare workers **Setting:** various healthcare settings **Intervention:** an intervention in which one's attention is on the experience of stress **Comparison:** no intervention/wait list/placebo/no stress‐reduction intervention				
**Outcomes**	Anticipated absolute effects^*^ (95% CI)	**№ of participants (studies)**	**Certainty of the evidence (GRADE)**	**What happens**
	**Effect with an intervention in which one's attention is on the experience of stress**			
Stress symptoms (follow‐up up to and including 3 months after end of intervention)	SMD 0.37 lower (0.52 lower to 0.23 lower)	3645 (41 RCTs)	⊕⊕⊝⊝ Low^1^	On the short term, an intervention in which one's attention is on the experience of stress may result in a reduction in stress symptoms. The standardized mean difference translates back to 4.6 fewer (6.4 fewer to 2.8 fewer) points on the MBI‐emotional exhaustion scale^2^.
Stress symptoms (follow‐up > 3 to 12 months after end of intervention)	SMD 0.43 lower (0.71 lower to 0.14 lower)	1851 (19 RCTs)	⊕⊕⊝⊝ Low^1^	On the medium term, focus one's attention on the experience of stress may result in a reduction in stress symptoms. The standardized mean difference translates back to 5.3 fewer (8.7 fewer to 1.7 fewer) points on the MBI‐emotional exhaustion scale^3^.
Stress symptoms (follow‐up >12 months after end of intervention)	no effect estimate	68 (1 RCT)	⊕⊝⊝⊝ Very low ^2^	The evidence is very uncertain about the long‐term effect on stress symptoms of focusing one's attention on the experience of stress.
*The risk in the intervention group (and its 95% confidence interval) is based on the assumed risk in the comparison group and the **relative effect** of the intervention (and its 95% CI). **CI:** confidence interval; **SMD:** standardized mean difference				
**GRADE Working Group grades of evidence** **High certainty:** we are very confident that the true effect lies close to that of the estimate of the effect. **Moderate certainty:** we are moderately confident in the effect estimate: the true effect is likely to be close to the estimate of the effect, but there is a possibility that it is substantially different. **Low certainty:** our confidence in the effect estimate is limited: the true effect may be substantially different from the estimate of the effect. **Very low certainty:** we have very little confidence in the effect estimate: the true effect is likely to be substantially different from the estimate of effect.				

^1^ The certainty of the evidence was downgraded by two levels for very serious risk of bias (bias arising from the randomisation process and lack of blinding; i.e. performance bias) in combination with some inconsistency and suspicion of publication bias. ^2^ The certainty of the evidence was downgraded by three levels for very serious risk of bias (bias arising from the randomisation process and lack of blinding; i.e. performance bias) and very serious imprecision (small sample size, the confidence interval includes both a benefit and a harm). ^3^ The MBI‐Emotional exhaustion scale has a total score of 54 and we used the mean score (23.6) and standard deviation (12.2) of the control healthcare workers population in Fiol DeRoque 2021 as reference for interpreting the effect sizes. A score below 18 points is regarded as a low score on emotional exhaustion and a score above 36 as a high score on emotional exhaustion (Maslach 1996).

**Summary of findings 2:** An intervention in which one's attention is away from the experience of stress compared to no intervention/wait list/placebo/no stress‐reduction intervention for stress reduction in healthcare workers

**An intervention in which one's attention is away from the experience of stress compared to no intervention/wait list/placebo/no stress‐reduction intervention for stress reduction in healthcare workers**				
**Patient or population:** healthcare workers **Setting:** various healthcare settings **Intervention:** an intervention in which one's attention is away from the experience of stress **Comparison:** no intervention/wait list/placebo/no stress‐reduction intervention				
**Outcomes**	Anticipated absolute effects^*^ (95% CI)	**№ of participants (studies)**	**Certainty of the evidence (GRADE)**	**What happens**
	**Risk with an intervention in which one's attention is away from the experience of stress**			
Stress symptoms (follow‐up up to and including 3 months after end of intervention)	SMD 0.55 lower (0.70 lower to 0.40 lower)	2366 (35 RCTs)	⊕⊕⊝⊝ Low ^1^	On the short term, an intervention in which one's attention is away from the experience of stress may result in a reduction in stress symptoms. The standardized mean difference translates back to 6.8 fewer (8.6 fewer to 4.9 fewer) points on the MBI‐emotional exhaustion scale^2^.
Stress symptoms (follow‐up > 3 to 12 months after end of intervention)	SMD 0.41 lower (0.79 lower to 0.03 lower)	427 (6 RCTs)	⊕⊕⊝⊝ Low ^1^	On the medium term, an intervention in which one's attention is away from the experience of stress may result in a reduction in stress symptoms. The standardized mean difference translates back to 5.0 fewer (9.7 fewer to 0.4 fewer) points on the MBI‐emotional exhaustion scale^2^.
Stress symptoms (follow‐up >12 months after end of intervention)	‐	(0 RCTs)	‐	No studies reported the long‐term effect on stress symptoms of focusing one's attention away from the experience of stress.
*The risk in the intervention group (and its 95% confidence interval) is based on the assumed risk in the comparison group and the **relative effect** of the intervention (and its 95% CI). **CI:** confidence interval; **SMD:** standardized mean difference				
**GRADE Working Group grades of evidence** **High certainty:** we are very confident that the true effect lies close to that of the estimate of the effect. **Moderate certainty:** we are moderately confident in the effect estimate: the true effect is likely to be close to the estimate of the effect, but there is a possibility that it is substantially different. **Low certainty:** our confidence in the effect estimate is limited: the true effect may be substantially different from the estimate of the effect. **Very low certainty:** we have very little confidence in the effect estimate: the true effect is likely to be substantially different from the estimate of effect.				

^1^ The certainty of the evidence was downgraded by two levels for very serious risk of bias (bias arising from the randomisation process and lack of blinding; i.e. performance bias) in combination with some inconsistency and suspicion of publication bias. ^3^ The MBI‐emotional exhaustion scale has a total score of 54 and we used the mean score (23.6) and standard deviation (12.2) of the control healthcare workers population in Fiol DeRoque 2021 as reference for interpreting the effect sizes. A score below 18 points is regarded as a low score on emotional exhaustion and a score above 36 as a high score on emotional exhaustion (Maslach 1996).

**Summary of findings 3:** An intervention in which the focus is on work‐related risk factors on an individual level compared to no intervention/no stress‐reduction interventionfor stress reduction in healthcare workers

**An intervention in which the focus is on work‐related risk factors on an individual level compared to no intervention/no stress‐reduction intervention for stress reduction in healthcare workers**				
**Patient or population:** healthcare workers **Setting:** various healthcare settings **Intervention:** an intervention in which the focus is on work‐related risk factors on an individual level **Comparison:** No intervention/no stress‐reduction intervention				
**Outcomes**	Anticipated absolute effects^*^ (95% CI)	**№ of participants (studies)**	**Certainty of the evidence (GRADE)**	**What happens**
	**Effect with an intervention in which the focus is on work‐related risk factors on an individual level**			
Stress symptoms (follow‐up up to and including 3 months after end of intervention)	no effect estimate	87 (3 RCTs)	⊕⊝⊝⊝ Very low ^1^	The evidence is very uncertain about the short‐term effect of an intervention in which the focus is on work‐related risk factors on stress symptoms.
Stress symptoms (follow‐up > 3 to 12 months after end of intervention)	no effect estimate	152 (2 RCTs)	⊕⊝⊝⊝ Very low ^2^	The evidence is very uncertain about the medium‐term effect of an intervention in which the focus is on work‐related risk factors on stress symptoms.
Stress symptoms (follow‐up >12 months after end of intervention)	no effect estimate	161 (1 RCT)	⊕⊝⊝⊝ Very low ^2^	The evidence is very uncertain about the long‐term effect of an intervention in which the focus is on work‐related risk factors on stress symptoms.
*The risk in the intervention group (and its 95% confidence interval) is based on the assumed risk in the comparison group and the **relative effect** of the intervention (and its 95% CI). **CI:** confidence interval; **SMD:** standardized mean difference; **MD**: mean difference				
**GRADE Working Group grades of evidence** **High certainty:** we are very confident that the true effect lies close to that of the estimate of the effect. **Moderate certainty:** we are moderately confident in the effect estimate: the true effect is likely to be close to the estimate of the effect, but there is a possibility that it is substantially different. **Low certainty:** our confidence in the effect estimate is limited: the true effect may be substantially different from the estimate of the effect. **Very low certainty:** we have very little confidence in the effect estimate: the true effect is likely to be substantially different from the estimate of effect.				

^1^ The certainty of the evidence was downgraded by three levels for very serious risk of bias (bias arising from the randomisation process and lack of blinding; i.e. performance bias), inconsistency and very serious imprecision (small sample size, the confidence interval includes both a benefit and a harm). ^2^ The certainty of the evidence was downgraded by three levels for very serious risk of bias (bias arising from the randomisation process and lack of blinding; i.e. performance bias) and very serious imprecision (small sample size, the confidence interval includes both a benefit and no effect).

**Summary of findings 4:** A combination of individual‐level interventions compared to no intervention/wait list/no stress‐reduction intervention for stress reduction in healthcare workers

**A combination of individual‐level interventions compared to no intervention/wait list/no stress‐reduction intervention for stress reduction in healthcare workers**				
**Patient or population:** healthcare workers **Setting:** various healthcare settings **Intervention:** a combination of individual‐level interventions **Comparison:** no intervention/wait list/no stress‐reduction intervention				
**Outcomes**	Anticipated absolute effects^*^ (95% CI)	**№ of participants (studies)**	**Certainty of the evidence (GRADE)**	**What happens**
	**Effect with a combination of individual‐level interventions**			
Stress symptoms (follow‐up up to and including 3 months after end of intervention)	SMD 0.67 lower (0.95 lower to 0.39 lower)	1003 (15 RCTs)	⊕⊕⊝⊝ Low ^1^	On the short term, a combination of individual‐level interventions may result in a reduction in stress symptoms. The standardized mean difference translates back to 8.2 fewer (11.7 fewer to 4.8 fewer) points on the MBI‐Emotional exhaustion scale^4^.
Stress symptoms (follow‐up > 3 to 12 months after end of intervention)	SMD 0.48 lower (0.95 lower to 0.00)	574 (6 RCTs)	⊕⊕⊝⊝ Low ^2^	On the medium term, a combination of individual‐level interventions may result in a reduction in stress symptoms, but the evidence does not exclude no effect. The standardized mean difference translates back to 5.9 fewer points (11.7 fewer to no difference) on the MBI‐Emotional exhaustion scale^4^.
Stress symptoms (follow‐up >12 months after end of intervention)	no effect estimate	88 (1 RCT)	⊕⊝⊝⊝ Very low ^3^	The evidence is very uncertain about the long‐term effect of a combination of individual‐level interventions on stress symptoms.
**CI:** confidence interval; **SMD:** standardized mean difference				
**GRADE Working Group grades of evidence** **High certainty:** we are very confident that the true effect lies close to that of the estimate of the effect. **Moderate certainty:** we are moderately confident in the effect estimate: the true effect is likely to be close to the estimate of the effect, but there is a possibility that it is substantially different. **Low certainty:** our confidence in the effect estimate is limited: the true effect may be substantially different from the estimate of the effect. **Very low certainty:** we have very little confidence in the effect estimate: the true effect is likely to be substantially different from the estimate of effect.				

^1^ The certainty of the evidence was downgraded by two levels for very serious risk of bias (bias arising from the randomisation process and lack of blinding; i.e. performance bias) in combination with some inconsistency and suspicion of publication bias. ^2^ The certainty of the evidence was downgraded by two levels for very serious risk of bias (lack of blinding; i.e. performance bias) and inconsistency. We did not downgrade for imprecision, as the wide confidence interval is due to the inconsistency between study results. ^3^ The certainty of the evidence was downgraded by three levels for very serious risk of bias (bias arising from the randomisation process and lack of blinding; i.e. performance bias) and very serious imprecision (small sample size, the confidence interval includes both a benefit and a harm). ^4^ The MBI‐emotional exhaustion scale has a total score of 54 and we used the mean score (23.6) and standard deviation (12.2) of the control HCW population in Fiol DeRoque 2021 as reference for interpreting the effect sizes. A score below 18 points is regarded as a low score on emotional exhaustion and a score above 36 as a high score on emotional exhaustion (Maslach 1996).

## Background

This is the second update of the original Cochrane Review (Marine 2006) published in 2006, Issue 4. Healthcare workers can suffer from work‐related stress as a result of organisational factors and an imbalance of demands, skills, and social support at work. Prolonged exposure to these factors negatively impacts the service these workers are able to provide (Tawfik 2019). Frequently, this leads to severe distress, burnout, or psychosomatic disorders amongst healthcare works and subsequent deterioration in service quality (Tawfik 2019).

### Description of the condition

Healthcare workers are at high risk of work‐related stress compared to the general working population. Prolonged exposure to work‐related stressors can overwhelm the coping capacities of healthcare workers leading to work‐related stress, which can gradually develop into a Stress‐Related Disorder (SRD) (van der Molen 2020). Symptoms of stress or SRDs, can manifest as physical (e.g. headaches, muscle tension or pain), mental (impaired concentration), behavioural (conflict with other people), and emotional (emotional instability) problems (van Dam 2021). 

It is challenging to determine the prevalence of SRDs globally as there is little agreement on the case definition (De Hert 2020). However, multiple studies report high levels of stress and burnout in groups of healthcare workers representing various disciplines. For example, Bridgeman 2018 reported that 30% to 70% of physicians and nurses experience burnout symptoms, while another study reported that 56% of anaesthesiologists experience burnout symptoms (Bridgeman 2018; De Hert 2020; Sanfilippo 2017).

There are a variety of factors in the workplace that may contribute to SRDs, such as lack of role clarity, effort–reward imbalance, systemic inequities, lack of social support, high emotional demands, and lack of decision authority (Bridgeman 2018; van der Molen 2020). Besides, personal factors, such as perfectionism or high standards, may also add to stress suggesting a multifactorial contribution to the development of SRDs (De Hert 2020).

The consequences of SRDs in healthcare workers are more far‐reaching than in some other professions as they can adversely affect the quality of patient care (Shanafelt 2010). Furthermore, the negative health effects for the individual healthcare worker should also not be underestimated as SRDs have been associated with coronary health problems, but also with low job satisfaction and cynicism (Bridgeman 2018; Costello 2016). SRDs may also affect healthcare organisations due to increased turnover rates and absenteeism (Maunder 2006). SRDs also have a large economic impact (Hassard 2018) which makes identifying effective interventions to reduce this burden an urgent one.

### Description of the intervention

Interventions at both the organisational level and the individual employee level are needed to prevent and reduce work‐related stress in healthcare workers. The scope of this review is limited to stress management interventions at the individual level. After the previous update of this review, we decided to modify our approach in describing individual‐level stress interventions. For this purpose, we looked at stress as a generic term that refers to two distinct concepts, namely ‘stressors’ (environmental characteristics, or thoughts which cause an adverse reaction in the individual) and ‘strain’ (the individual’s adverse reaction to the stressor) (Bamber 2006; Beehr 1987; Knapp 1988). Given these concepts of stress, one can differentiate three separate avenues of intervention: 1. factors in one’s environment (e.g. work) that cause stress (the focus of the Cochrane Review by Giga 2018), 2. one’s thoughts relating to stress, and 3. the adverse emotional experience resulting from the former two. Various cognitive‐behavioural approaches aim to alleviate the experience of stress and prevent it from becoming chronic (e.g. burnout, depression or somatic illness) by changing the ways in which an individual worker thinks about and manages the perception of stressors in his/her work and the resulting thoughts and feelings. The third approach springs from the idea that the emotional experience of stress is harmful in itself, especially when extended over a long period of time, and so the aim of intervention is to alleviate the emotional response directly by, for example, relaxation techniques. In effect, something else is brought in to take the place mostly occupied by stressful thoughts and feelings (Bamber 2006; Beehr 1987; Knapp 1988). In order to maximise usability and intuitiveness of the results of our review, we reframed the latter two approaches as interventions that focus at thoughts and feelings related to stress and as interventions in which the focus is turned away from thoughts and feelings related to stress.

We conceptualised four distinct approaches to addressing work‐related stress at the individual level:

1. focus one’s attention on the (modification of the) experience of stress (thoughts, feelings, behaviours); 
2. focus one’s attention away from the experience of stress by various means of psychological disengagement; 
3. alter work‐related risk factors on an individual level; and
4. combine two or more of the above.

The first approach consists of, but is not limited to, the following: cognitive‐behavioural techniques: assertiveness training, coping skills training, and communication skills training. The second approach includes approaches such as relaxation, massage, mindfulness meditation, exercise (e.g. yoga, tai chi, stretch‐release, drawing, acupuncture, etc.), and playing or listening to music. 

Note that with regard to mindfulness it is sometimes difficult to judge whether the central element of the intervention is to focus on the experience of stress or away from it. For example, the general principles of mindfulness‐based stress reduction and cognitive‐behavioural therapy are similar such as increased awareness, regulation, cognitive flexibility and goals‐based behaviours. However, some studies such as mindfulness‐meditation solely used mindfulness techniques to shift focus away from the experience of stress by directing attention to the present moment (Hofmann 2017; Tang 2015). We therefore categorise interventions like mindfulness‐based stress reduction as type 1 and mindfulness‐based meditation as type 2. 

The third approach focus' on work‐related risk factors and typically includes planning, scheduling, adjusting work demands on an individual level. 

The last approach consists of a combination of two or more of the first three approaches. For instance, combining cognitive behavioural techniques with relaxation. 

### How the intervention might work

By focusing on the experience of stress and its possible causes, it may be possible to manage one's thoughts, feelings, behaviours and to change these by learning new techniques to do so (Beck 2005). For example, cognitive behavioural therapy (CBT) focus' on the thoughts and feelings that drive behaviours. The overarching goal of this approach is to manage stress at work is to help individuals control the automatic thoughts that exacerbate emotional difficulties such as severe distress, burnout, and depression (Beck 2005).

By diverting one's attention away from the experience of stress by means of relaxation, exercise, or something else, it may be possible to reduce the overall experience of stress (Creswell 2014). The goal is to induce a state of mental and bodily calm in order to counteract the agitation caused by stress. This can be achieved by, for example, being a passive recipient of a massage (Mahdizadeh 2019), or by actively performing various exercises such as yoga (Fang 2015). The focus is thus directed towards a specific relaxing activity and away from the unpleasant thoughts and feelings associated with stress (Borges 2021).

Modifying work‐related risk factors on an individual level may also influence stress levels. An example of this approach is that healthcare workers can have a say in their own work schedule or can make adjustments to their workloads or receive training to identify what may cause stress and think about alterations they could make to their job to discuss with their supervisor (Arrigoni 2015).

### Why it is important to do this review

An extensive number of reviews have been published on the effectiveness of interventions to reduce stress in healthcare workers (Aryankhesal 2019; Busireddy 2016; Patel 2019; Sanfilippo 2017; Wiederhold 2018; Zhang 2020). However, some reviews are focused on one specific group of healthcare workers like nurses or physicians (Aryankhesal 2019) and other reviews have only focused on the effectiveness of one type of intervention such as mindfulness (Fendel 2021). To the best of our knowledge there are no up‐to‐date reviews that examine the effectiveness of various types of individual‐level interventions aimed at reducing stress in various healthcare workers to provide a more complete overview. Despite the fact that healthcare workers consist of a multitude of job tasks and titles they still form a reasonably homogeneous population such that it is reasonable to assume interventions directed to them would achieve roughly similar results regardless of specific job title.

It is important to offer healthcare workers interventions that are aimed at reducing the adverse effects of stress. When prevention is offered in a timely manner, it can reduce stress and prevent SRDs. It is therefore important to investigate which interventions are effective (Alberdi 2016). Prevention of SRDs has several advantages. Firstly, it can protect the health of the healthcare workers (Bridgeman 2018). Second, it is also better for the quality of patient care (De Hert 2020). And lastly, there is already a shortage of healthcare workers due to high turnover rates and effective prevention of SRDs may help reduce this. However, there is no consensus about which interventions are effective to prevent SRDs in healthcare workers. It is therefore important to publish an updated version of this review, also because healthcare workers have been affected more by SRDs than before the COVID‐19 pandemic (Blake 2020).

Because the characteristics of interventions designed for healthcare workers may be different from those of other occupations, the aim of this review is to determine the effectiveness of interventions to reduce SRDs specifically in healthcare workers. Given the large amount of included studies in the review evaluating all stress interventions in healthcare workers (Ruotsalainen 2015), the update was divided into this review on individual‐level interventions and another one by Giga 2018 which focus' solely on organisational interventions. These two reviews together supersede the review that was first published in 2006 (Marine 2006) and updated in 2015 (Ruotsalainen 2015). Since this review focus' on individual‐level interventions, studies that solely focused on organisational factors (i.e. quantitative demands, emotional tasks, variation of work, influence at work) are excluded. 

## Objectives

To evaluate the effectiveness of stress‐reduction interventions targeting individual healthcare workers compared to no intervention, wait list, placebo, no stress‐reduction intervention or another type of stress‐reduction intervention in reducing stress symptoms. 

## Methods

### Criteria for considering studies for this review

#### Types of studies

Consistent with the previous versions of this review, we limited inclusion to randomised controlled trials (RCTs) to evaluate intervention effectiveness. We only included completed studies published in peer‐reviewed scientific journals; abstracts without accompanying full texts and dissertations were excluded. 

#### Types of participants

We included studies in which the interventions were directed at healthcare workers who had not actively sought help for conditions such as burnout, depression, or anxiety disorder. This included all healthcare workers and trainees in any healthcare setting engaged in clinical work. We excluded studies in which any portion of participants were not doing clinical work, e.g. administrators, receptionists or when the outcomes were not reported separately for the participants who were doing clinical work. Personal caregivers who were family members or friends were excluded from this review.

#### Types of interventions

We included RCTs that evaluated the effectiveness of any type of intervention for individual healthcare workers aimed at preventing or reducing symptoms of stress. We excluded interventions targeting healthcare organisations because they are covered by the Giga 2018 review. Generally, four approaches to managing work‐related stress at the individual level can be distinguished:

1. focus one’s attention on the (modification of the) experience of stress (thoughts, feelings, behaviour); 

2. focus one’s attention away from the experience of stress by various means of psychological disengagement; 

3. alter work‐related risk factors on an individual level; and

4. combine two or more of the above.

Interventions such as mindfulness‐based stress reduction which focus on increasing awareness, regulation, cognitive flexibility and goals‐based behaviour directly related to stress were classified as type 1, whereas mindfulness ‐based meditation (Hofmann 2017; Tang 2015) that aim to shift attention away from the experience of stress and unpleasant thoughts was in type 2. 

We included all trials that compared the effectiveness of an active intervention with no intervention (including usual care), wait list, a placebo intervention, no stress‐reduction intervention or to another type of stress‐reduction intervention. 

The distinction between no intervention, wait list, placebo intervention, and no stress‐reduction intervention is not always apparent. We considered the comparison with a placebo intervention when participants were blinded to group assignment and both groups were told that they received a stress reduction intervention and the placebo intervention has no ‘active ingredient’. For instance when transcranial magnetic stimulation is compared to sham transcranial magnetic stimulation (Kim 2016). Trials with placebo arms were combined with those with no‐intervention controls, wait list controls and no stress‐reduction intervention controls in the meta‐analysis.

We considered the comparison with another type of stress‐reduction intervention when both groups received some kind of stress reduction intervention that was not part of regular care. In this comparison participants may or may not be blinded to group assignment. The comparison could include only different types of interventions, for instance, type 1 versus type 2 (psycho‐educational stress management (SMC) vs mindfulness‐based stress reduction (MSBR) (Errazuriz 2022). 

#### Types of outcome measures

We included studies that evaluated the effectiveness of interventions using validated and standardised self‐report questionnaires measuring symptoms of work‐related stress or burnout. We deemed all other outcomes that do not measure stress or its effects on individuals beyond the scope of this review. Examples of excluded outcomes are: risk factors for stress (such as workload, conflicts, support), coping skills, knowledge or attitude change, work performance, patient satisfaction and claims from clients, employee absenteeism and turnover.

We considered the following follow‐up times for outcome measurement:

- short term defined as up to and including three months after the intervention has been completed;
- medium term defined as more than three months up to 12 months; and
- long term defined as 12 months or longer.

##### Primary outcomes

Validated and standardised self‐report questionnaires measuring symptoms of work‐related stress or burnout examples of these measures include the following. 

1. Perceived Stress Scale (PSS) (Cohen 1983).
2. Maslach Burnout Inventory (MBI) (comprised of three subscales: emotional exhaustion, depersonalisation, personal accomplishment) (Maslach 1982).
3. Depression Anxiety Stress Scale (DASS) (Lovibond 1995).
4. General Health Questionnaire (GHQ) (Goldberg 1991).
5. Oldenburg Burnout Inventory (OBI) (Demerouti 2003).
6. Visual Analogue Scale ‐ stress symptoms.
7. Copenhagen Burnout Inventory (CBI) (Kristensen 2005).

##### Secondary outcomes

For secondary outcomes we considered all outcome measures of the detrimental effects of stress or burnout. These included measures such as: (a) Psychological symptoms: anxiety and depression, such as the State‐Trait Anxiety Inventory (Spielberger 1970), Beck Depression Inventory (BDI) (Beck 1961) and Hospital Anxiety Depression Scale (HADS) (Zigmond 1983); (b) Measures of the cost‐effectiveness of interventions, such as incremental cost‐effectiveness ratios (ICERs), incremental cost‐per‐QALY (quality‐adjusted life year) and cost‐benefit ratios. Studies that reported only one or more of the secondary outcomes without any primary outcomes were excluded.

### Search methods for identification of studies

We used a replacement approach and used the previous review (Ruotsalainen 2015) as one source of studies. Hence, two sources were used:

1. Included studies in the previous version of this review (Ruotsalainen 2015), search date up to November 2013.
2. Electronic searches (2013 to February 2022)

#### Electronic searches

1. Cochrane Central Register of Controlled Trials (CENTRAL) (*The Cochrane Library*, 2013 to February 2022)
2. MEDLINE/PubMed (2013 to February 2022)
3. Embase (2013 to February 2022)
4. PsycINFO (2013 to February 2022)
5. CINAHL/EBSCO (2013 to February 2022)
6. Web of Science (2013 to February 2022)

#### Searching other resources

We examined the reference lists from included articles and reviews for any additional eligible studies.

### Data collection and analysis

#### Selection of studies

We used Covidence (Covidence 2022) for screening. Six review authors (ST, LE, AL, AT, KN, HM) independently screened titles and abstracts followed by full‐texts against the inclusion criteria. If there was any disagreement, the two review authors involved discussed this until disagreement was resolved.

#### Data extraction and management

Three review authors conducted the extraction of data by using a made‐to‐measure data extraction form in Covidence (ST, LE, AL) (Covidence 2022). Data extraction of the outcomes was done independently by the three review authors or researchers and students from the medical faculty of the University of Amsterdam. One review author checked all data extraction and reached consensus in cases of conflict. All questions concerning data extraction processes were resolved by discussion with all review authors.

#### Assessment of risk of bias in included studies

We used the Cochrane risk of bias tool (Higgins 2011) to assess the risk of bias in included studies. The tool includes the following assessment items: adequate sequence generation, allocation concealment, blinding, incomplete outcome data addressed, selective outcome reporting, and other bias. 

#### Measures of treatment effect

We plotted the results of each trial as means and standard deviations (SDs) for continuous outcomes. Because in many cases different instruments were used to measure stress, we transformed the means into standardised mean differences (SMDs). 

In many cases multiple similar outcome measures were used, or an instrument had several subscales but no summary measure. In case of multiple similar outcomes, we chose the outcome which we deemed to best represent a measure of stress symptoms in healthcare workers, such as the PSS (Cohen 1983). When study authors used subscales such as with the MBI (Maslach 1996), we chose the subscale that in our view best represented stress, such as the emotional exhaustion scale of the MBI (Maslach 1996). 

#### Unit of analysis issues

For studies that employed a cluster‐randomised design and that reported sufficient data to be included in the meta‐analysis and that did not make an allowance for the design effect, we calculated the design effect based on a fairly large assumed intra‐cluster correlation (ICC) of 0.10. Even though we did not find information for the ICC)for these types of studies we assumed that 0.10 would be a realistic estimate. We used studies from implementation research to support this assumption (Campbell 2001). We followed the methods stated in the Cochrane Handbook for Systematic Reviews of Interventions (Cochrane Handbook, Higgins 2022) for the calculations: design effect = 1+(M‐1)*ICC, where M is the average cluster size and ICC is the intra‐cluster correlation coefficient.

For studies with multiple study arms and one control condition, we combined groups to create a single pair‐wise comparison with the control condition.

For studies with multiple study arms and no control condition, we entered the first two study arms in the meta‐analysis. 

#### Dealing with missing data

 Where necessary, we sought missing data (means and standard deviations (SDs)) from authors. In total, 16 study authors either provided data that had not been published in their articles which enabled us to enter these studies into the meta‐analyses, provided clarification on their published article, or referred us to supplementary information (Barattucci 2019; Cohen‐Katz 2005; Dunne 2019; Dyrbye 2019; Errazuriz 2022; Gärtner 2013; Jensen 2006; Kline 2020; Moody 2013a; Oman 2006; Ozgundondu 2019; Pehlivan 2020; Sampson 2019; Sawyer 2021; West 2014; West 2021).

Where necessary and possible, we used WebPlotDigitizer (Rohatgi, 2022) to retrieve means and SDs from figures for the following studies: CezardaCosta 2019; Cheng 2015; Luthar 2017; Kline 2020 (control group only). 

When SDs were not reported we calculated them from other reported values according to the methods stated in the Cochrane Handbook (Higgins 2022). 

For West 1984 we took the means and SDs that resulted from the post‐hoc comparisons in the repeated measures analyses. For Norvell 1987, we took the post‐treatment values and calculated SDs based on the P value. We calculated a t‐value from this P value even though the authors used a Mann‐Whitney U test. For Shapiro 2005, we took the post‐treatment values and the F‐value reported by the authors. We calculated a t‐value and subsequent SDs by taking the square root of the F‐value as the t‐value. For Tsai 1993, we took the post‐treatment values from the figure reporting the results of the repeated measures' analysis. We took the reported P value belonging to the repeated measures' analysis as if it had resulted from a t‐test and calculated the SDs based on this t‐value. For Ewers 2002, we took the post‐treatment scores and the P values belonging to the independent t‐tests to calculate a t‐value and subsequently SDs. 

For Dahlgren 2022 and Gunasingam 2015, we calculated SDs based on 95% confidence intervals (CIs). For CezardaCosta 2019, Mao 2021 and Riley 2017 we calculated SDs based on the standard error (SE.) For Seidel 2021, the N per group was not reported, we assumed that there were an equal number of participants in the two study groups, i.e. 41 and 42. 

Lee 2021, Mealer 2014 and Ozgundondu 2019 reported their stress outcomes with a median and interquartile range (IQR). In accordance with the Cochrane Handbook (Higgins 2022) we requested mean and SDs. In the case we did not receive a response, we entered the median and IQR in the meta‐analysis, and we assumed that outcome data were normally distributed. 

Participants are included in the groups to which they were originally randomised, but missing data for participants were not included in the denominator.

In the case missing SDs were either not provided by the study authors or could not be calculated, these missing data were not imputed. 

#### Assessment of heterogeneity

We assessed heterogeneity in line with GRADE guidance (Schünemann 2013). We deemed an I² value of more than 50% to indicate considerable heterogeneity. When we identified heterogeneity, we tried to understand the reasons for the heterogeneity by exploring the options outlined in the Cochrane Handbook (Higgins 2022) and we investigated the presence of outlying studies. When the heterogeneity could not be explained, we downgraded the certainty of the evidence. In addition, we calculated the prediction intervals, to provide information about how much the true effect size varies across studies calculated with CMA Prediction Intervals.

#### Assessment of reporting biases

We avoided reporting bias by including studies and not articles. If multiple articles reported results from a single study, we consolidated all the data from all articles under one study ID only. We avoided language bias by including studies in any language. Because standardised mean differences (SMDs) are related to their standard error (SE) (Zwetsloot 2017), we did not use the SEs to generate a funnel plot instead we used the sample size as recommended by Zwetsloot 2017. The funnel plots were generated in STATA 17 (STATA 2022).

#### Data synthesis

We combined studies that we deemed sufficiently similar regarding participants, intervention, control, outcome and follow‐up time in one comparison.

We pooled the results statistically when the outcomes were similar concepts, such as perceived stress symptoms. Because many instruments were used, we used SMDs to combine the stress‐related outcomes using meta‐analysis. Not all instruments used one summary score, but presented the results of various subscales. In cases where there was no summary measure, we chose the subscale that best represented a measure of stress. For example, for this analysis, we used only the emotional exhaustion subscale of the Maslach Burnout Inventory (MBI). In this way, we considered the various stress symptoms scales to measure the same concept. We pooled the results using a random‐effects model. 

To interpret the effect size, the mean (23.6) and SD (12.2) on the MBI of the control healthcare worker population in Fiol DeRoque 2021 was used. The MBI‐Emotional exhaustion scale has a total score of 54. A score below 18 points is regarded as a low score on emotional exhaustion and a score above 36 as a high score on emotional exhaustion (Maslach 1996).

#### Subgroup analysis and investigation of heterogeneity

We conducted the following subgroup analyses and incorporated them in all comparisons:

- type of intervention (see Types of interventions for more details);
- length of follow‐up (see Types of outcome measures for more details);
- type of outcome (see Primary outcomes; Secondary outcomes for more details).

On top of those subgroups, we considered the subgroups mentioned in the original protocol, i.e. type of healthcare worker and duration and intensity of the intervention (Marine 2000). When considering those subgroups, we took into account that subgroup effects on top of the current subgroups in interventions and outcomes may prove spurious and may not explain all the variability in the extent of inconsistency, as most putative subgroup effects ultimately prove spurious (Schünemann 2013). 

##### Type of healthcare worker

For the current update of the review that includes only individual‐level interventions, we considered a subgroup analysis by type of healthcare worker as redundant. The reason is that we think that the intervention types included in this review work the same way for various healthcare workers (e.g. physicians, nurses). The previous findings of this review and a recent publication on this topic (de Wijn 2022) supported this assumption. In the previous review update (Ruotsalainen 2015), it was concluded that a subgroup analyses based on type of healthcare worker did not explain heterogeneity (“Since working conditions differ considerable between various occupations in health care, we analysed if there were differences in the effects of CBT and relaxation between various occupations. We did so only for comparisons with sufficient studies: CBT versus no intervention and relaxation versus no intervention. We ignored the previous subgroups in the CBT and relaxation intervention categories and divided the studies according to the occupation of the participants into nurses, physicians, all staff and other healthcare professionals. There were no differences between these subgroups. Within the subgroups however, there was still considerable statistical heterogeneity. We therefore do not think that the occupation of the participants explains statistical heterogeneity between studies.") Therefore, we cancelled this subgroup analysis and reported this in the section "Differences between protocol and review". 

##### Duration and intensity of the intervention

For this update, we discussed the proposed subgroup analyses based on the duration and intensity of the intervention as stated in the original protocol (Marine 2000). We discussed what a proper grouping would be and found that dividing the studies in short or longer and intense or less intense interventions would be an arbitrary approach as no definition was formulated a priori. Moreover, such a grouping would ideally be based on a mixture of the duration and intensity of the intervention (e.g. number of sessions, the length of the sessions, homework assignments) and the compliance with the intervention. However, we explored whether the arbitrary cut‐off for duration of the intervention of 12 weeks shows an effect in effect size. We added this in the "Differences between protocol and review". 

#### Compliance

de Wijn 2022 found that stress management interventions for nurses in which the sample was exposed to the majority of the planned sessions reached greater effect sizes compared to interventions in which the compliance to the intervention/attendance to the planned sessions was lower. This finding should be interpreted with caution due to a lot of missing data (de Wijn 2022). However, we aimed to explore if the effect sizes based on studies in which participants attended 80% or more of the scheduled sessions would differ from the studies where participants attended less than 80% of the scheduled sessions. We added this in the "Differences between protocol and review".   

#### Sensitivity analysis

To assess the effect of risk of bias on the pooled results, we performed a sensitivity analysis in which we excluded studies with a high risk of bias and assessed whether this changed the results appreciably. We defined a study having a high risk of bias overall when we judged it to have a high risk of bias in three or more domains. 

#### Summary of findings and assessment of the certainty of the evidence

We used the GRADE approach to assess the certainty of the body of evidence for the intervention categories and comparisons most important for health decision‐making (Guyatt 2011). A priori, we decided that the comparisons of an intervention with no intervention are most important for decision‐making for the primary outcome of stress symptoms only for all three follow‐up times. Comparisons of one intervention versus another intervention were considered to be less informative. We downgraded the certainty of the evidence by one to three levels depending on the seriousness of the violations in each domain. We considered the risk of bias tables for each study in that intervention category to assess the risk of bias for an intervention category. We downgraded the certainty of the evidence if there were one or more limitations in the following domains: risk of bias, consistency, directness of the evidence, precision of the pooled estimate and the possibility of publication bias. All statements on the effects of interventions, such as in the summary of finding tables and the conclusion were worded in line with the recommendations on communicating findings when using the GRADE approach (Santesso 2020). Review authors ST and JB undertook GRADE, which was then also discussed with JR, RS, KN, LE and HM until consensus was reached.  

## Results

### Description of studies

#### Results of the search

From the initial set of included articles for the earlier systematic review (Ruotsalainen 2015), we included 28 eligible articles. Furthermore, we included one previously excluded article (Gärtner 2013). The systematic searches updated in 2018 and February 2022 yielded altogether 4776 references, excluding duplicates. We assessed 254 full‐text articles for eligibility and excluded 160. This left 92 new articles. Put together, 120 articles describing 117 studies fulfilled our inclusion criteria (Figure 1). 

**1:**
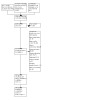


In addition, we located eight ongoing studies (Baker 2015; Bateman 2020; Bratt 2022; Kuribayashi 2019; Ng 2019; Pérula‐de Torres 2019; Rees 2018; Weiner 2020) for which we could not find published outcome data. 

In addition, six studies were published in a language other than English or Dutch, for which we were currently unable to find professional translation (Ahmadi 2019; Ghods 2017; Lu 2020; Rogala 2016; Taft 2021; Xiao Yan 2019). In three studies (Fei 2019; Klatt 2012; Valipour 2020) no full‐text was available, and two (Imamura 2019; Sasaki 2021) did not report on stress symptoms despite being specified in the trial protocol. Details for these studies are provided in "Characteristics of studies awaiting classification". 

We sought additional information regarding study details and statistical data or both from 26 included studies, and we received a response from 11 study authors (Barattucci 2019; Dunne 2019; Dyrbye 2019; Errazuriz 2022; Kline 2020; Ozgundondu 2019; Pehlivan 2020; Sampson 2019; Sawyer 2021; West 2014; West 2021). From the set of included studies from Ruotsalainen 2015, the previous author team received additional information from five study authors (Cohen‐Katz 2005; Gärtner 2013; Jensen 2006; Moody 2013a; Oman 2006).

Nine included studies could not be included in the meta‐analysis for various reasons. For Chen 2015, Duchemin 2015, Ghawadra 2020, Martins 2011, Novoa 2014, Palumbo 2012, Schrijnemaekers 2003 and Tonarelli 2018 it was due to missing data. Our efforts to reach these study authors were unsuccessful. Leao 2017 used a dichotomous outcome measure that we could not enter into the meta‐analysis.  

One final update search was run on the 26th of September 2022, yielding 555 records excluding duplicates. We assessed 29 full‐text articles for eligibility and excluded 12. Seventeen articles were added to the "Studies awaiting classification" and will be considered in the next update of this review. We furthermore assessed 44 trial registration records and excluded 42. Two were added to the "Ongoing studies" (Al‐Hammouri 2022; Jeffers 2017). 

#### Included studies

This second review update included 117 included studies (11,119 participants), this included an additional 89 studies (8691 participants) since the last update (Ruotsalainen 2015). 

#### Study designs

Of the 117 included studies, 109 were individually randomised controlled trials (RCTs), and eight were cluster‐RCTs.

Seven of the cluster‐RCTs that we included in the meta‐analysis had a unit of analysis error. In other words, these studies ignored the clustering of the data in their analysis. To address this we used a formula (see "Unit of analysis issues") to calculate the design effect based on average cluster size (M) and an intra‐cluster correlation coefficient. We calculated the design effect as 2.4 for the Barbosa 2015 study (four clusters, M = 14.5), 1.5 for Gärtner 2013 (57 clusters, M = 5.4); 2.01 for the Jensen 2006 study (19 clusters, M = 11.05), 2.0 for Kesselheim 2020 (nine clusters, M = 11.1), 3.15 for Sampson 2019 (four clusters, M = 22.5); 3.7 for Verdes Montenegro Atalaya 2021 (six clusters, M = 27.5) and 3.5 for Sharif 2013 (two clusters, M = 26). We used the design effect to reduce the number of participants in both intervention and control groups if we were able to use quantitative outcome data in meta‐analyses.

#### Country and time period

Forty‐one studies had been carried out in North America, 35 in Europe, 19 in Asia, nine in the Middle East, nine in South America, three in Oceania and one on two continents.

#### Type of settings and participants

Altogether 94 of the included studies had been conducted in hospitals, four in residential care homes for the elderly or persons with disabilities, 14 in mixed or other healthcare settings, and five in a Medical Emergency service. Sixty studies included exclusively nurses, 23 included physicians, and 34 various or other healthcare staff. Almost all studies (N = 105) did not formulate inclusion or exclusion criteria based on stress symptom levels, while 12 studies (Behnammoghadam 2019; Chen 2015; Ghawadra 2020; Günüsen 2010; Kurebayashi 2012; Kurebayashi 2014; Montibeler 2018; Novoa 2014; Peterson 2008; Prado 2018; Saganha 2012; Stanton 1988) included healthcare workers with a medium and/or high level of stress symptoms only. 

#### Sample sizes

The total number of participants randomised was 11,119. The number of participants per study arm was (Simmons 2018) < 50 in 85 studies (Alexander 2015; Amutio 2015; Aranda Ausern 2016; Axisa 2019; Bagheri 2019; Barbosa 2015; Behnammoghadam 2019; Bernburg 2019; Bernburg 2020; Brennan 2006; CezardaCosta 2019; Cheng 2015; Chesak 2020; Cho 2021; Cohen‐Katz 2005; Concilio 2021; Copeland 2021; deSouza 2021; Dincer 2021; Duchemin 2015; Dunne 2019; Dyrbye 2019; Emani 2020; Errazuriz 2022; Ewers 2002; Gollwitzer 2018; Gunasingam 2015; Günüsen 2010; Hilcove 2021; Ho 2021; Huang 2020a; Janzarik 2022; Kavurmaci 2022; Kharatzadeh 2020; Kim 2016; Kline 2020; Kurebayashi 2012; Leao 2017; Lebares 2021; Lee 1994; Lee 2021; Lin 2015; Lin 2019; Luthar 2017; Mache 2015; Mache 2016; Mache 2017; Mache 2018; Mackenzie 2006; Martins 2011; McGonagle 2020; Mealer 2014; Medisauskaite 2019; Moench 2021; Montibeler 2018; Moody 2013a; Norvell 1987; Novoa 2014; OBrien 2019; Oman 2006; Ozbas 2016; Ozgundondu 2019; Palumbo 2012; Pehlivan 2020; Prado 2018; Redhead 2011; Reynolds 1993; Riley 2017; Saganha 2012; Sampson 2019; Sawyer 2021; Schroeder 2018; Seidel 2021; Shapiro 2005; Sharif 2013; Shin 2020; Sood 2011; Stanton 1988; Tonarelli 2018; Verdes Montenegro Atalaya 2021; West 1984; West 2014; Yazdani 2010; Yung 2004; Zarvijani 2021) and ≥ 50 in 32 studies (Barattucci 2019; Brazier 2022; Chen 2015; Dahlgren 2022; Dyrbye 2016; ElKhamali 2018; Fendel 2021; Finnema 2005; Fiol DeRoque 2021; Foji 2020; Frogeli 2020; Gärtner 2013; Ghawadra 2020; Grabbe 2020; Hersch 2016; Huang 2020; Jensen 2006; Kesselheim 2020; Kurebayashi 2014; Lee 2020; Mandal 2021; Mao 2021; McConachie 2014; Melchior 1996; Montaner 2021; PelitAksu 2020; Peterson 2008;  Schrijnemaekers 2003; Tsai 1993; Wei 2017; West 2021; Xie 2020). 

#### Interventions

Fifty‐two studies examined the effectiveness of focusing on the experience of stress (Amutio 2015; Axisa 2019; Bagheri 2019; Barattucci 2019; Behnammoghadam 2019; Cheng 2015; Chesak 2020; Dyrbye 2016; Dyrbye 2019; Errazuriz 2022; Fendel 2021; Fiol DeRoque 2021; Foji 2020; Frogeli 2020; Gärtner 2013; Ghawadra 2020; Gollwitzer 2018; Grabbe 2020; Gunasingam 2015; Günüsen 2010; Huang 2020; Huang 2020a; Jensen 2006; Kesselheim 2020; Kharatzadeh 2020; Lee 1994; Lee 2020; Lin 2019; Mache 2015; Mache 2016; Mache 2017; Mache 2018; Mackenzie 2006; Mao 2021; Martins 2011; McConachie 2014; McGonagle 2020; Medisauskaite 2019; Moody 2013a; Pehlivan 2020; Riley 2017; Sampson 2019; Sawyer 2021; Schroeder 2018; Sharif 2013; Tonarelli 2018; Verdes Montenegro Atalaya 2021; Wei 2017; West 1984; West 2021; Xie 2020; Zarvijani 2021). The content of the interventions varies for instance from cognitive‐behavioral therapy to emotional skills training. 

Forty‐two studies examined the effectiveness of focusing away of the experience of stress (Alexander 2015; Aranda Ausern 2016; Brennan 2006; CezardaCosta 2019; Chen 2015; Cho 2021; Cohen‐Katz 2005; Copeland 2021; Dahlgren 2022; deSouza 2021; Dincer 2021; Duchemin 2015; Dunne 2019; Emani 2020; Errazuriz 2022; Hilcove 2021; Ho 2021; Kavurmaci 2022; Kim 2016; Kline 2020; Kurebayashi 2012; Kurebayashi 2014; Leao 2017; Lebares 2021; Lee 2021; Lin 2015; Mandal 2021; Montibeler 2018; Novoa 2014; Oman 2006; Ozgundondu 2019; Palumbo 2012; PelitAksu 2020; Prado 2018; Saganha 2012; Seidel 2021; Shapiro 2005; Shin 2020; Stanton 1988; Tsai 1993; Yazdani 2010; Yung 2004). The content of the interventions varies from yoga to meditation to music listening. 

Seven studies examined ways to alter work‐related risk factors on an individual level (Concilio 2021; Ewers 2002; Finnema 2005; Melchior 1996; Peterson 2008; Redhead 2011; Schrijnemaekers 2003).

Seventeen studies examined a combination of interventions (Barbosa 2015; Bernburg 2019; Bernburg 2020; Brazier 2022; ElKhamali 2018; Hersch 2016; Janzarik 2022; Luthar 2017; Mealer 2014; Moench 2021; Montaner 2021; Norvell 1987; OBrien 2019; Ozbas 2016; Reynolds 1993; Sood 2011; West 2014).

Three studies compared only two different types of stress prevention interventions with one another (Barbosa 2015; Riley 2017; Xie 2020). 

The duration of the intervention ranged from one session (e.g. Axisa 2019) to 12 weeks (e.g. Chesak 2020) with most interventions lasting a few sessions only.

#### Type of control group 

Most included studies used a no‐intervention control group (N = 72). Another 27 studies used a waiting‐list control group. Eight studies used a no stress‐reduction control group (Brennan 2006; Concilio 2021; Grabbe 2020; Jensen 2006; Mao 2021; Tsai 1993; West 2014; Tonarelli 2018) and another seven studies used a placebo control group (Chen 2015; Fiol DeRoque 2021; Kim 2016; Lee 2021; Prado 2018; Shin 2020; Novoa 2014). 

#### Multiple intervention arms

Fifteen studies compared two or more active stress interventions with a control condition (Cheng 2015; Copeland 2021; Errazuriz 2022; Gärtner 2013; Gollwitzer 2018; Günüsen 2010; Kline 2020; Kurebayashi 2012; Kurebayashi 2014; Leao 2017; Lebares 2021; Pehlivan 2020; Verdes Montenegro Atalaya 2021; West 1984; Yung 2004).

With Cheng 2015; Copeland 2021; Gärtner 2013; Gollwitzer 2018; Günüsen 2010; Kline 2020; Kurebayashi 2012; Kurebayashi 2014; Pehlivan 2020; Verdes Montenegro Atalaya 2021; Yung 2004, we combined intervention arms to create a single pair‐wise comparison. 

With Lebares 2021 we entered both interventions in the same comparison with the two control groups. We entered the intervention reported in Errazuriz 2022 in different comparisons. West 1984 had five study arms but finally reported data only on one study arm versus a no‐intervention or no‐effect condition. We used this as an intervention versus no‐intervention comparison. We did not enter Leao 2017 in the comparison as the outcomes were dichotomous. 

#### Multiple control arms

Four studies included two control arms (Jensen 2006; Novoa 2014; Lebares 2021; Prado 2018). With Jensen 2006 we compared the intervention arm with the no stress‐reduction intervention arm. With Prado 2018 we used the placebo control arm instead of the wait list control group. We did not include Novoa 2014 in the meta‐analysis due to missing values. With Lebares 2021 we compared both control arms to the two intervention arms. 

#### Outcomes

Altogether 43 studies used the Maslach Burnout Inventory (MBI) while 29 studies used the Perceived Stress Scale (PSS). The remaining studies used stress symptom questionnaires such as Perceived Stress Questionnaire (PSQ), Depression Anxiety Stress Scale (DASS‐stress), or General Health Questionnaire (GHQ). Twenty‐one studies reported a depression or anxiety outcome measure such as the State‐Trait Anxiety Inventory (STAI), DASS or Center for Epidemiologic Studies Depression Scale (CES‐D). 

Only one study (Gärtner 2013) reported the cost‐effectiveness of their intervention. 

#### Follow‐up  

##### *(i) Short term*

There were 105 studies with an outcome measurement between the end of the intervention up to and including three months after the intervention. 

##### * (ii) Medium term*

In 34 studies there was a follow‐up measurement between three and 12 months after intervention. 

##### *(iii) Long term*

Only four studies had a follow‐up measurement more than 12 months after the intervention. 

#### Excluded studies

The main reasons for excluding studies from this review were as follows (see the "Characteristics of excluded studies" for more detail).

1. Wrong outcomes

2. Wrong study design

3. Wrong publication type

4. Wrong population (not only healthcare workers)

5.  Wrong intervention

### Risk of bias in included studies

In general, we judged most included studies to suffer from methodological issues, with at least two items that we judged to put them at a high risk of bias (Figure 2). We judged only four studies to have no domain with a high risk of bias or to NOT have more than two domains with an uncertain risk of bias (Barbosa 2015; Cheng 2015; deSouza 2021; Fiol DeRoque 2021). 

**2:**
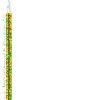
Risk of bias summary: review authors' judgements about each risk of bias item for each included study.

Blinding was consistently problematic in almost all studies because study authors used self‐report to assess stress symptoms as the participants and the providers could not be blinded to the intervention. However, in 14 studies participants were (tried to be) blinded to group assignment (Barbosa 2015; Chen 2015; Cheng 2015; Concilio 2021; deSouza 2021; Fiol DeRoque 2021; Kim 2016; Lebares 2021; Lee 2021; Luthar 2017; Medisauskaite 2019; Novoa 2014; Prado 2018; Shin 2020). 

#### Allocation

Half of the included studies did not clearly describe the method for generating random numbers or did not employ a truly random sequence. It is surprising to note that some of these studies provided only sparse details on the randomisation process. Details of allocation concealment were frequently lacking. In most of the included studies, we assumed that randomisation was applied blind to all eligible participants at the same time. If this assumption is correct then researchers and participants could not foresee assignment. We therefore rated this as unclear risk of bias. 

#### Blinding

We considered the reporting of stress symptoms by questionnaires as an outcome assessment that could be biased by knowledge of the intervention. We judged that it could be possible that a participant in the intervention group, knowing that they have gone through a six‐week course of stress management, would rate their stress symptoms more favourably than a person in the control group. This would create an overestimation of the effect of the intervention. Most authors mentioned that blinding could be an issue, but also discussed that blinding was not possible in these circumstances. We rated these studies as having a high risk of bias. However, in 16 studies, participants were (tried to be) blinded to group assignment. These mainly came from the second category of interventions (focussing away from stress) such as aromatherapy, auriculotherapy or acupressure or studies in which two or more active interventions were compared with one another. 

#### Incomplete outcome data

Twenty‐five percent of the included studies had attrition rates exceeding 20% of the initial sample. When explanations for loss‐to‐follow‐up were missing, when reasons were not entirely random, or when the responders differed from non‐responders on baseline characteristics, we judged these studies to be at high risk of attrition bias. In some studies, it was unclear whether participants dropped out and the studies were therefore labelled as being at unclear risk of bias.

#### Selective reporting

It is surprising to note that most studies lack a study protocol or trial registration. When studies lacked a protocol, it was difficult to judge if outcomes were reported as planned. If the authors mentioned a protocol, we reviewed the protocol for a priori outcomes. If there was no mention of a protocol we looked online to see whether there was a protocol published. If not, we judged reporting in the study based on the methods and results sections.”. In most studies there was no indication of selective outcome reporting. In one study (Jensen 2006) only significant differences were reported, which we took to be a sign of high risk of bias. In Finnema 2005 the results for nursing assistants consisted of covariance analyses that were not prespecified and because of this, we judged the study to be at high risk of bias. In four studies (Bagheri 2019; Dahlgren 2022; ElKhamali 2018; Errazuriz 2022) the trial protocol mentioned a stress symptom questionnaire that was not reported in the included studies, which we took to be a sign of high risk of bias. In Dincer 2021 participants randomised to the intervention group that did not attend the: Emotional Freedom Techniques (EFT) session (n = 5) were excluded, which we took to be a sign of high risk of bias. In Kurebayashi 2012 the authors present data separately for participants who had high SSL scores to begin with but not at all for participants with a moderate SSL score, which was also categorised as high risk of bias. 

#### Other potential sources of bias

There were several risks of bias that came up in addition to the risks mentioned above, such as low or unclear compliance with the intervention or low or unclear response rate. If other biases were not apparent, we judged the other potential source of bias as low in the risk of bias tool.

### Effects of interventions

See: Table 1; Table 2; Table 3; Table 4

See: Table 1; Table 2; Table 3; and Table 4 and GRADE assessment of the primary outcomes at the end of this section for full description of how we rated the certainty of the evidence.  

#### 1. Focus one's attention on the experience of stress vs. no intervention/wait list/placebo/no stress‐reduction intervention

##### 1.1. Any symptoms of stress‐related outcome (Follow‐up to 3 months after the end of the intervention)

We combined the results of 41 studies. There was a standardised mean difference (SMD; of ‐0.37, 95% confidence interval (CI) ‐0.52 to ‐0.23) showing difference in stress symptoms between the interventions that focus one's attention on the experience of stress and no intervention/wait list/placebo/no stress‐reduction intervention up to and including three months after the end of the intervention (3645 participants; low‐certainty evidence; Analysis 1.1). We found considerable heterogeneity (I^2^ = 77%) and a 95% prediction interval from ‐1.19 to 0.45. When excluding three outlying SMDs, I^2^ reduced to 57%. The funnel plot revealed a lack of studies in the right part of the funnel where the negative studies would be expected, indicating that there could be publication bias (Figure 3). 

**3:**
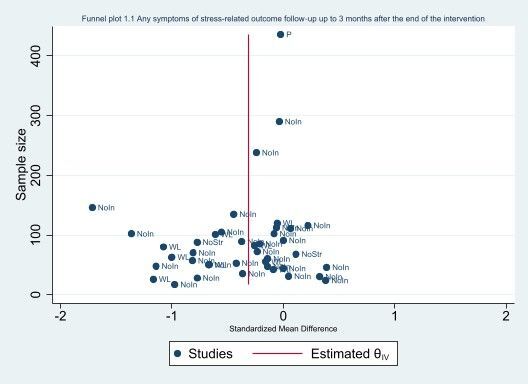
Funnel plot (1.1 Any symptoms of stress‐related outcome (follow‐up up to 3 months after the end of the intervention)

##### 1.2 Any symptoms of stress‐related outcome (Follow‐up from > 3 to 12 months after the end of the intervention)

Results from 19 studies suggested that an intervention focusing on the experience of stress decreased stress symptoms more than no intervention/wait list/no stress‐reduction intervention (SMD ‐0.43, 95% CI ‐0.71 to ‐0.14; 1851 participants; low‐certainty evidence; Analysis 1.2) at > 3 to 12 months follow‐up. We found considerable heterogeneity (I^2^ = 88%) and a 95% prediction interval from ‐1.70 to 0.84. When excluding two outlying SMDs, I^2^ reduced to 42%. The funnel plot revealed a lack of studies in the right part of the funnel where the negative studies would be expected, indicating that there could be publication bias (Figure 4). 

**4:**
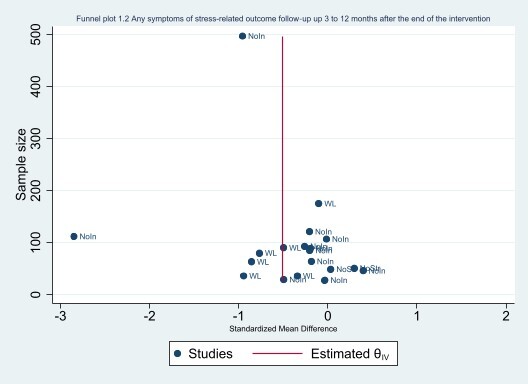
Funnel plot (1.2 Any symptoms of stress‐related outcome (follow‐up > 3 to 12 months after the end of the intervention))

##### 1.3 Any symptoms of stress‐related outcome (Follow‐up > 12 months after the end of the intervention)

One study combining two intervention arms showed no differences (mean difference (MD) 0.40, 95% CI ‐1.50 to 2.30) in stress symptoms of an intervention focusing on the experience of stress compared to no intervention at >12 months of follow‐up (68 participants; very low‐certainty evidence; Analysis 1.3). It was not possible to study heterogeneity or publication bias. 

##### 1.4 Psychological symptoms (Follow‐up to and including 3 months after the end of the intervention)

Eight studies showed no differences in psychological symptoms after interventions focusing on the experience of stress more than after no intervention/wait list up to and including three months after the intervention (SMD ‐0.27, 95% CI‐0.58 to 0.03; 742 participants; Analysis 1.4).

##### 1.5 Psychological symptoms (Follow‐up from > 3 to 12 months after the end of the intervention)

Three studies showed no differences in psychological symptoms in the interventions focusing on the experience of stress compared to no intervention participants on psychological symptoms > 3 to 12 months after the intervention (no pooled effect estimate; 196 participants; Analysis 1.5).

#### 2. Focus one's attention away from the experience of stress vs. no intervention/wait list/placebo/no stress‐reduction intervention 

##### 2.1. Any symptoms of stress‐related outcome (Follow‐up to and including 3 months after the end of the intervention)

We combined the results of 35 studies. This resulted in a SMD of ‐0.55 (95% CI ‐0.70 to ‐0.40) showing that stress symptoms were reduced with interventions that focus one's attention away from the experience of stress when compared to no intervention/wait list/placebo/no stress‐reduction intervention and when measured up to and including three months after the end of the intervention (2366 participants; low certainty‐evidence; Analysis 2.1). We found considerable heterogeneity (I^2^ = 68%) and a 95% prediction interval from ‐1.33 to 0.23. When excluding one outlying SMD, I^2^ reduced to 33%. The funnel plot revealed a lack of studies in the right part of the funnel where the negative studies would be expected, indicating that there could be publication bias (Figure 5). 

**5:**
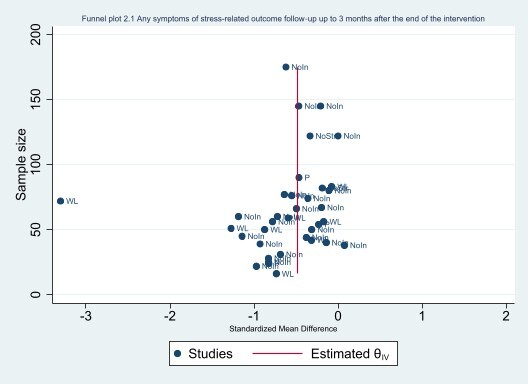
Funnel plot (2.1 Any symptoms of stress‐related outcome (follow‐up up to 3 months after the end of the intervention))

##### 2.2 Any symptoms of stress‐related outcome (Follow‐up from >3 to 12 months after the end of the intervention)

Results from six studies indicated that an intervention focusing away from the experience of stress decreased stress symptoms more than no intervention/wait list (SMD ‐0.41, 95% CI ‐0.79 to ‐0.03; 427 participants; low‐certainty evidence; Analysis 2.2) at > 3 to 12 months follow‐up. We found considerable heterogeneity (I^2^ = 71%) and a 95% prediction interval from ‐1.71 to 0.89. When excluding one outlying SMD, I^2^ reduced to 0%. It was not possible to study publication bias with a funnel plot due to the low number of studies included in the analysis. 

##### 2.3 Any symptoms of stress‐related outcome (Follow‐up from > 12 months after the end of the intervention)

No data found for this outcome.

##### 2.4 Psychological symptoms (Follow‐up to and including 3 months after the end of the intervention)

Seven studies found that an intervention focusing away from the experience of stress resulted in an SMD of ‐1.07 (95% CI ‐1.95 to ‐0.19) of psychological symptoms compared to no intervention/wait list/placebo up to and including three months after the end of the intervention (378 participants; Analysis 2.3). No data found for other follow‐up measurements of psychological symptoms.

#### 3. Focus on work‐related risk factors on an individual level vs. no intervention/no stress‐reduction intervention

##### 3.1. Any symptoms of stress‐related outcome (Follow‐up to and including 3 months after the end of the intervention)

One study showed that focusing on work‐related risk factors on an individual level decreased stress symptoms more than no intervention up to and including three months after the end of the intervention (SMD ‐1.23; 95% CI ‐2.21 to ‐0.26) while two others showed no difference in stress symptoms (no pooled effect estimate; 87 participants; very low‐certainty evidence; Analysis 3.1). We found considerable heterogeneity (I^2^ = 70%) and a 95% prediction interval from ‐10.12 to 9.68. It was not possible to study publication bias. 

##### 3.2 Any symptoms of stress‐related outcome (Follow‐up from > 3 to 12 months after the end of the intervention)

One study showed that focusing on work‐related risk factors on an individual level decreased stress symptoms more than no intervention >3 to 12 months after the end of the intervention (SMD ‐0.38, 95% CI ‐0.73 to ‐0.03) while one study showed no difference in stress symptoms (SMD 0.09, 95% CI ‐0.78 to 0.95) (no pooled effect estimate; 152 participants; very low‐certainty evidence; Analysis 3.2). With two studies, no funnel plot could be made. 

##### 3.3 Any symptoms of stress‐related outcome (Follow‐up > 12 months after the end of the intervention)

One study showed no difference (MD ‐1.52, 95% CI ‐3.61 to 0.57) in stress symptoms of focusing on work‐related risk factors on an individual level > 12 months after the end of the intervention (161 participants; very low‐certainty evidence; Analysis 3.3).

##### 3.4 Psychological symptoms (Follow‐up from > 3 to 12 months after the end of the intervention)

One study showed no effect (MD ‐1.07, 95% CI ‐2.90 to 0.76) of focusing on work‐related risk factors on an individual level on psychological symptoms > 3 to 12 months after the end of the intervention (110 participants; Analysis 3.4).

#### 4. Combination of intervention types vs. No intervention/wait list/no stress‐reduction intervention

##### 4.1. Any symptoms of stress‐related outcome (Follow‐up to and including 3 months after the end of the intervention)

We combined the results of 15 studies and found a SMD of ‐0.67 (95% CI ‐0.95 to ‐0.39) showing less stress symptoms after the combined interventions and no intervention/wait list/no stress‐reduction intervention up to and including three months (1003 participants; low‐certainty evidence; Analysis 4.1). We found considerable heterogeneity (I^2^ = 77%) and a 95% prediction interval from ‐1.75 to 0.41. When excluding three outlying SMDs, I^2^ reduced to 46%. The funnel plot revealed a lack of studies in the right part of the funnel where the negative studies would be expected, indicating that there could be publication bias (Figure 6). 

**6:**
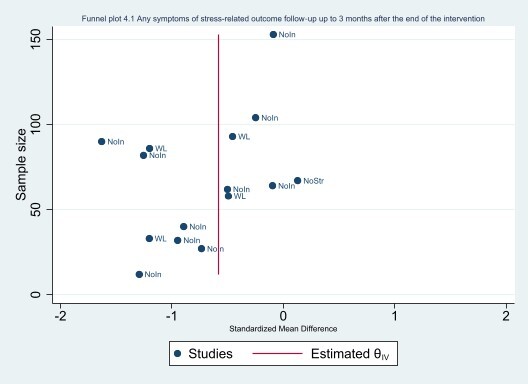
Funnel plot (4.1 Any symptoms of stress‐related outcome (follow‐up up to 3 months after the end of the intervention))

##### 4.2 Any symptoms of stress‐related outcome (Follow‐up from > 3 to 12 months after the end of the intervention)

According to six studies, a combined intervention did not decrease stress symptoms more than no intervention/wait list/no stress‐reduction intervention at > 3 to 12 months follow‐up (SMD ‐0.48, 95% CI ‐0.95 to 0.00; 574 participants; low‐certainty evidence; Analysis 4.2). We found considerable heterogeneity (I^2^ = 87%) and a 95% prediction interval from ‐1.85 to 1.79. It was not possible to study heterogeneity or publication bias. 

##### 4.3 Any symptoms of stress‐related outcome (Follow‐up > 12 months after the end of the intervention)

One study showed no difference (MD ‐1.80, 95% CI ‐5.74 to 2.14) in stress symptoms of a combined intervention on an individual level > 12 months after the end of the intervention (88 participants; very low‐certainty evidence; Analysis 4.3). It was not possible to study heterogeneity or publication bias. 

##### 4.4 Psychological symptoms (Follow‐up to and including 3 months after the end of the intervention)

Four studies showed no differences in psychological symptoms of a combination of intervention types > 3 to 2 months after the end of the intervention (no pooled effect estimate, 192 participants; Analysis 4.4).

##### 4.5 Psychological symptoms (Follow‐up from > 3 to 12 months after the end of the intervention)

One study (MD ‐2.20, 95% CI ‐5.88 to 1.48) showed no differences in psychological symptoms of a combined intervention > 3 to 12 months after the end of the intervention on psychological symptoms (91 participants; Analysis 4.5).

##### 4.6 Psychological symptoms (Follow‐up from > 12 months after the end of the intervention)

One study showed no differences (MD ‐2.10, 95% CI ‐5.43 to 1.23) in psychological symptoms of a combined intervention > 12 months after the end of the intervention on psychological symptoms (88 participants; Analysis 4.6).

#### 5. Focus one's attention on the experience of stress vs. focus one's attention away from the experience of stress

##### 5.1. Any symptoms of stress‐related outcome (Follow‐up to and including 3 months after the end of the intervention)

Three studies showed no differences in stress symptoms of focusing one's attention on the experience of stress versus focusing one's attention away from the experience of stress up to an including three months after the end of the intervention (no pooled effect estimate, 193 participants; Analysis 5.1). 

##### 5.2 Any symptoms of stress‐related outcome (Follow‐up from > 3 to 12 months after the end of the intervention)

Two studies showed no differences in stress symptoms of focusing one's attention on the experience of stress versus focusing one's attention away from the experience of stress > 3 to 12 months after the end of the intervention (no pooled effect estimate, 74 participants; Analysis 5.2) at > 3 to 12 months follow‐up. 

##### 5.3 Any symptoms of stress‐related outcome (Follow‐up > 12 months after the end of the intervention)

One study showed no differences in stress symptoms of an intervention focusing on the experience of stress compared to focusing away from stress at > 12 months of follow‐up (38 participants; Analysis 5.3).

##### 5.4 Psychological symptoms (Follow‐up to and including 3 months after the end of the intervention)

One study showed no differences in on psychological symptoms of an intervention focusing on the experience of stress compared to focusing away from stress (38 participants; Analysis 5.4).

##### 5.5 Psychological symptoms (Follow‐up from > 3 to 12 months after the end of the intervention)

No data found for this outcome.

#### 6. Combination of interventions vs. focus one's attention on the experience of stress

##### 6.1. Any symptoms of stress‐related outcome (Follow‐up to and including 3 months after the end of the intervention)

One study showed no differences in stress symptoms of a combined intervention versus focus one's attention on the experience of stress up to and including three months after the end of the intervention (no effect estimate; 24 participants; Analysis 6.1).

##### 6.2 Any symptoms of stress‐related outcome (Follow‐up from > 3 to 12 months after the end of the intervention)

One study showed no differences in stress symptoms of a combined intervention versus focus one's attention on the experience of stress > 3 to 12 months after the end of the intervention (no effect estimate; 24 participants; Analysis 6.2).

##### 6.3 Psychological symptoms 

No data found for this outcome.

#### GRADE assessment

##### 1. Focus one's attention on the experience of stress vs. no intervention

The certainty of the evidence for Analysis 1.1 and Analysis 1.2 was downgraded by two levels for very serious risk of bias (bias arising from the randomisation process and lack of blinding; i.e. performance bias) in combination with some inconsistency and suspicion of publication bias. The certainty of the evidence for Analysis 1.3 was downgraded by three levels for very serious risk of bias (bias arising from the randomisation process and lack of blinding; i.e. performance bias) and very serious imprecision (small sample size, the confidence interval includes both a benefit and a harm).

##### 2. Focus one's attention away from the experience of stress vs. no intervention

The certainty of the evidence for Analysis 2.1 and Analysis 2.2 was downgraded by two levels for very serious risk of bias (bias arising from the randomisation process and lack of blinding; i.e. performance bias) in combination with  some inconsistency and suspicion of publication bias. 

##### 3. Focus on work‐related risk factors on an individual level vs. no intervention

The certainty of the evidence for Analysis 3.1 was downgraded by three levels for very serious risk of bias (bias arising from the randomisation process and lack of blinding; i.e. performance bias), inconsistency and very serious imprecision (small sample size, the confidence interval includes both a benefit and a harm). The certainty of the evidence for Analysis 3.2 and Analysis 3.3 was downgraded by three levels for very serious risk of bias (bias arising from the randomisation process and lack of blinding; i.e. performance bias) and very serious imprecision (small sample size, the confidence interval includes both a benefit and no effect).

##### 4. Combination of intervention types vs. no intervention

The certainty of the evidence for Analysis 4.1 was downgraded by two levels for very serious risk of bias (bias arising from the randomisation process and lack of blinding; i.e. performance bias) in combination with some inconsistency and suspicion of publication bias. The certainty of the evidence for Analysis 4.2 was downgraded by two levels for very serious risk of bias (lack of blinding; i.e. performance bias) and inconsistency. We did not downgrade for imprecision, as the wide confidence interval is due to the inconsistency between study results. The certainty of the evidence for Analysis 4.3 was downgraded by three levels for very serious risk of bias (bias arising from the randomisation process and lack of blinding; i.e. performance bias) and very serious imprecision (small sample size, the confidence interval includes both a benefit and a harm).

#### Subgroup analysis

We considered duration of the intervention as a characteristic to analyse studies with short duration separately from studies with longer duration. Analysis 1.6 and Analysis 2.4 show an exploratory subgroup analysis based on duration. No differences were seen between these subgroups. Within the subgroups there was still considerable heterogeneity. We have no reason to assume that the duration of the intervention explains heterogeneity between studies, but we're very uncertain about the effect of duration of the intervention on stress symptoms.  

We considered compliance as a characteristic to analyse studies with poor compliance separately from studies with better compliance. However, we found that compliance was not reported in about 50% of the studies. We decided that subgroup analysis in which half of the studies could not be included would not be of added value and no conclusions can be drawn from such analyses. 

#### Sensitivity analysis

Most studies did not blind participants and therefore the overall certainty in the effect estimates is reduced. In order to provide an indication of the robustness of the overall conclusions we conducted the sensitivity analyses according to protocol for each comparison. 

##### 1. Focus one's attention on the experience of stress vs. no intervention

Removing low‐quality studies from the comparison of focusing one's attention on the experience of stress vs. no intervention on the short term left 31 studies. The SMD changed slightly from ‐0.37 (95% CI ‐0.52 to ‐0.23) to ‐0.49 (95% CI ‐0.67 to ‐0.31). For the same comparison for the medium term, removing low‐quality studies left eight studies. The SMD changed slightly from ‐0.43 (95% CI ‐0.71 to ‐0.14) to ‐0.41 (95% CI ‐0.65 to ‐0.17). The overall results and the direction of the effect for this comparison seem not to be affected by methodological quality of the included studies, and we considered the results of the analyses robust. For the same comparison for the long term, removing low‐quality studies left zero studies, so no sensitivity analysis was possible. 

##### 2. Focus one's attention away from the experience of stress vs. no intervention

Removing low‐quality studies from the comparison of focusing one's attention away from the experience of stress vs. no intervention on the short term left 24 studies. The SMD changed slightly from ‐0.55 (%95 CI ‐0.70 to ‐0.40) to ‐0.45 (95% CI ‐0.58 to ‐0.33). The overall results and the direction of the effect for this comparison seem not to be affected by methodological quality of the included studies, and we considered the results of the analyses robust. For the same comparison for the medium term, removing low‐quality studies left five studies, so no sensitivity analysis was possible. 

##### 3. Focus on work‐related risk factors on an individual level vs. no intervention

Removing low‐quality studies from the comparison of focusing one's attention on individual work‐related risk factors vs. no intervention on the short term left two studies, so no sensitivity analysis was possible. For the same comparison for the medium term, removing low‐quality studies left one study, so no sensitivity analysis was possible. For the same comparison for the long term, removing low‐quality studies left zero studies, so no sensitivity analysis was possible. 

##### 4. Combination of intervention types vs. no intervention

Removing low‐quality studies from the comparison of a combination of intervention types vs. no intervention in the short term left 13 studies in the comparison. The SMD changed slightly from ‐0.67 (95% CI ‐0.95 to ‐0.39) to ‐0.74 (95% CI ‐1.06 to ‐0.42). The overall results and the direction of the effect for this comparison seem not to be affected by methodological quality of the included studies, and we considered the results of the analyses robust. For the same comparison in the medium term, four studies were included and in the long term no studies were included precluding any analysis.

## Discussion

### Summary of main results

The primary objective of this review was to examine the effect of individual‐level stress management interventions on stress symptoms in healthcare workers. This review update includes an additional 89 studies, bringing the total number of studies to 117. Overall, the findings from the synthesis of randomised controlled trials (RCTs) indicate that there may be an effect on stress reduction in healthcare workers from individual‐level stress interventions, whether they focus one's attention **on** or **away** from the experience of stress. This effect may last up to a year after the end of the interventions. The evidence on the long‐term effect (more than a year after the end of the intervention) on stress symptoms for these two types of interventions is unclear.

In the short term, less than three months after the end of the intervention, a combination of individual‐level interventions may result in a reduction in stress symptoms. The evidence of effects thereafter or in the long term is inconclusive.

Only seven studies investigated interventions in which the focus is on work‐related risk factors, such as work demands. Due to this lack of evidence, we do not know if this type of intervention is effective. 

### Overall completeness and applicability of evidence

This systematic review includes the most recent evidence from studies published between 2013 and February 2022, which ensures that our findings are suitable for and applicable to current healthcare settings. 

The majority of the included studies were conducted in hospitals (94), the remaining 23 were conducted in other healthcare contexts (14 in mixed or other healthcare settings, five in medical emergency, four in residential care homes for the elderly or disabled). We believe that the results are generalisable to most healthcare situations, but they are most applicable to the hospital setting. Half of the studies (60) included nurses only, 23 physicians and 34 various or other healthcare staff. This is a more diverse population, compared to earlier Cochrane Reviews on the same topic (Ruotsalainen 2015), enhancing the applicability of our findings. 

About 64% of the studies were conducted in the Western industrialised world (North America and Europe) and 16% in Asia. The remaining 20% of the studies were spread over the Middle East, South America and Oceania and one study on two continents. Studies conducted in Africa are missing, just as in the former review (Ruotsalainen 2015). The findings are therefore not applicable to the large continent of Africa. 

The outcome measurements were diverse, focusing on outcomes such as burnout (such as the Maslach Burnout Inventory (MBI)) or on the experience of stress symptoms (List of Stress symptoms). The lack of clarity on the definition of occupational stress is reflected in this wide range of outcome measurements.

With regard to the interventions, we note that a minority of the included studies focused on the root cause of occupational stress: altering work‐related risk factors. One might expect this type of intervention to yield the most long‐term, sustainable changes, so it is a shortcoming that not more studies took this approach. Almost half (44%) of the studies focused on the experience of stress in itself and 36% on focusing away from stress. A smaller percentage (15%) of the interventions focused on a combination of the above approaches. Furthermore, studies do not really seem to distinguish whether their intervention program is aimed at the prevention of occupational stress or aimed at the treatment of (early) stress symptoms which might also contribute to a lack of clarity on the definition of (occupational) stress.

### Quality of the evidence

We assessed the methodological quality of the included RCTs using the Cochrane risk of bias tool (Higgins 2011). We assessed most included studies as having a high risk of bias arising from the randomisation process and lack of blinding; i.e. performance bias and to a lesser extent due to losses to follow‐up. The lack of blinding is problematic as the findings may be explained, at least in part, by a placebo effect. We tried to decrease the heterogeneity of the evidence generated by assessing intervention effects in four categories and with distinct follow‐up times (i.e. up to three months, three to 12 months, and more than one year after the intervention) and on distinct outcomes. We found some inconsistency. Inconsistency could also arise from the categorisation of interventions. The remaining variation within these categories could be due to dissimilar mechanisms of change. 

We downgraded the certainty of evidence by two levels for interventions focusing attention on the experience of stress until one‐year follow‐up by two levels for very serious risk of bias (bias arising from the randomisation process and lack of blinding; i.e. performance bias) in combination with some inconsistency and suspicion of publication bias. We downgraded the certainty of evidence by two levels for interventions focusing attention on the experience of stress with three levels for longer follow‐up periods due to very serious risk of bias and very serious imprecision (intervention could be harmful or beneficial). 

For interventions focusing attention away from the experience of stress until one‐year follow‐up, we downgraded the certainty of the evidence by two levels due to very serious risk of bias in combination with some inconsistency and suspicion of publication bias.

For interventions focusing on work‐related risk factors on an individual level, we downgraded three levels due to very serious risk of bias, imprecision and inconsistency.

For combined interventions, we downgraded the certainty of the evidence by two levels for the outcome until one‐year follow‐up and by three levels for longer follow‐up periods due to very serious risk of bias, inconsistency, imprecision, or suspicion of publication bias.

### Potential biases in the review process

Potential biases could be caused by missing studies with our search strategy and because study authors did not always present the necessary information, sometimes even after we contacted them. However, we assess the effect of these possible biases to be very low because our search strategy was very extensive and the high number of studies we were able to include in the comparisons. Also, we checked relevant references of the included studies. By explicitly operationalising the types of individual targeted interventions that were eligible in each type of intervention and by focusing on stress symptoms only, we reduced bias due to differences in interpretation between the author team. We further substantiated this by adding explicitly that the operationalisation of the types of individual targeted interventions and focus on the effect of stress symptoms only. 

We reduced bias due to differences in interpretation between the author team. Bias might have been introduced when multiple stress symptom questionnaires were measured other than the Perceived Stress Scale or the Maslach Burnout Inventory (MBI), and we had to decide which was "the best" to include in the meta‐analysis. Since, not all questionnaires have been validated very well this decision might sometimes be arbitrary and not based on high‐quality evidence (Shoman 2021). However, since all questionnaires have the same underlying construct i.e. measuring stress symptoms, we feel that the effect on the overall conclusion is small. Nonetheless, when more evidence on the psychometric properties of stress symptom questionnaires is available, these decisions might be reconsidered. We have made these decisions transparent by providing all stress symptom questionnaire that have been measured by the authors in the characteristic of included studies and providing with a footnote which stress symptom questionnaires was included in the meta‐analysis. 

Potential bias might be introduced by the categorisation of interventions into focusing on stress, focusing away from stress, work‐related, and combination as variation remains within each category. In the previous version of this review, the main categorisation was in person‐ and work‐directed interventions. In this update, we further specified the person‐directed interventions based on the ideas of Bamber 2006. By doing this, we tackled the difficulties encountered with the previous categorisation (Ruotsalainen 2015). Future studies should focus on unravelling underlying stress mechanisms. 

### Agreements and disagreements with other studies or reviews

Our review aimed to assess the effect of all individual‐level stress interventions for all types of healthcare workers. This approach provided us with the opportunity to group interventions according to a general working mechanism (i.e. interventions that draw one's attention on the experience of stress and interventions in which one's attention is drawn away from the experience of stress or interventions that focus on work‐related risk factors) rather than according to one specific intervention type. As such, we categorised mindfulness‐based interventions into different comparisons, depending on whether yoga and relaxation was the main goal or whether mindfulness was embedded in a more cognitive‐behavioural approach, such as mindfulness‐based stress reduction. 

Our review has a different conclusion than a systematic review focusing on hospital nurses (Jung 2021). That review looked at the effect of various mind‐body modalities on mental health and concluded that there was no difference in burnout symptoms between groups that received mindfulness as part of a stress‐reduction programme and those receiving no intervention. But one study comparing yoga to usual care did find a difference in burnout scores. Our review found that there may be an effect for interventions focusing one's attention away from the stress experience (such as yoga) as well as interventions focusing one's attention on the stress experience (mindfulness‐based stress reduction). Our review differs from the Jung 2021 review in that our objective was broader, including all healthcare workers and all stress‐related outcome measures. We reasoned that for individual‐level interventions, the intervention effect for different type of healthcare workers should be comparable. As a result, we were able to include more studies and calculate an effect estimate with more certainty. 

The Spinelli 2019 review focused on mindfulness only but also took the approach of looking across healthcare occupations. As a result, they were able to include 38 RCTs. Overall, they found moderate effects on stress outcomes and a small effect on burnout specifically. They reported that the included mindfulness interventions all appeared to significantly affect overall outcomes. This is more in line with our findings, although we did look at non‐mindfulness individual‐level interventions as well. Another review (Zhang 2021) focusing on physical relaxation (such as yoga and massage therapy) in all healthcare workers included 15 RCTs. Their conclusion was that these methods reduce occupational stress compared to no intervention control groups. This is in line with our finding that focusing one's attention away from stress results in stress reduction. Their network analysis revealed yoga as the best method within these types of interventions. 

Another Cochrane Review by Kunzler 2020 evaluated the evidence for resilience interventions in healthcare workers. As resilience building and stress reduction interventions often go hand in hand, their findings provide a valuable companion to our review. They concluded based on 17 RCTs that resilience training may lead to lower levels of stress, which was a secondary outcome in their review. Resilience training most often was based on mindfulness and cognitive‐behavioural therapy and was comparable to our category of interventions that draw one's attention to the experience of stress. However, their conclusion was based on very low‐certainty evidence, which is a problem of more reviews on healthcare workers on this topic, such as Clough 2017, while we graded the certainty of the evidence as low. 

Our review focuses on healthcare workers, however it is relevant to compare our findings to other reviews examining different occupations. In the Richardson 2008 review of 55 interventions as tested in 36 experimental studies, a significant medium to large effect on occupational stress was found, and the effect was significantly and consistently larger for cognitive behavioural interventions. In contrast, in our review we found that there may be an effect for cognitive behavioural interventions as for relaxation techniques. This might be explained by the occupation of healthcare workers, we focused on, and the type of stressors these employees face. Healthcare workers often have to deal with inevitable situations like death of a patient or telling patients about their permanent loss of quality of life. In these stressful situations that cannot be influenced (any more), the active coping and practice of functional responses which is at the heart of cognitive behavioural approaches, might not be the most suitable way of coping. A more passive way of coping such as relaxation or mediation (refocus, away from the stress) might be a better fit to these types of stressors and this might explain why we did not find a bigger effect of the cognitive behavioural approaches compared to the relaxation techniques. 

## Authors' conclusions

**Implications for practice**

Our review shows that there may be an effect on stress in healthcare workers from individual‐level stress interventions, whether they focus one's attention on or away from the experience of stress. This effect may last up to a year after the end of the intervention. A combination of interventions might be beneficial as well, at least in the short term. The long‐term effects, longer than a year after the intervention ended, of individual‐level stress interventions remain unknown. The same applies for interventions focussed on modifying work‐related risk factors. 

The estimates of the effects of individual‐level stress interventions may be biased because of a lack of blinding of the participants in the studies. The true effect of interventions in which one's attention is directed on or away from the experience of stress is likely to be close to the estimate of the effect, but there is a possibility that the effect is substantially different (e.g. due to placebo effect). The effect could be potentially smaller than our synthesis of the available evidence indicated. Our confidence in the effect of combinations of interventions is limited, and the true effect may be substantially different from the estimate of the effect. We have very little confidence in the effect of individual‐level interventions in which the focus is on work‐related risk factors. Based on the included studies we cannot indicate whether or not there is any effect, even though this approach is often considered to be the most impactful and sustainable way to eliminate stress in the workplace. These interventions tend to be complex because they require changes in how the work is organised, designed and managed, which is often beyond the scope of the individual employee (Nielsen 2010). Also, difficulties in adequately measuring the effects might explain why this kind of intervention does not live up to the expectations researchers have of them based on theories. 

Country‐specific policies and legislation can influence what types of interventions are implemented. In most countries, there is some legislation on health and safety at work, but the extent and quality varies between countries according to a report by the World Health Organization (Burton 2010). The minimum variant is protecting workers from injuries or illness, but more refined legislation is in place in many countries requiring aspects such as a risk assessment, and the implementation and monitoring of measures. However, examining whether such legislation is effective is beyond the scope of our review.

**Implications for research**

The findings of this review show the need for methodologically better designed and executed studies. Trials of this type are required as nearly all included studies suffered from lack of blinding of participants and personnel. We acknowledge the difficulty of blinding in stress reduction interventions. Nevertheless, in 14 studies attempts were made to blind participants to group assignment, thus showing that blinding is not impossible. Better design and execution of studies also include providing details on the randomisation process and study protocol or trial registration.

Furthermore, there is a need for more studies on interventions in which the focus is on work‐related risk factors both at the individual and organisational level. With more participants the optimal information size can be reached and conclusions can be drawn. 

We believe it would be helpful to investigate and identify unpublished data (potentially showing no effect or a harmful effect) of individual‐level stress management interventions. Large studies on this topic might also help resolve this small‐study issue. 

The long‐term effects of individual‐level stress management interventions are unknown due to the total absence of studies or paucity of data. Studies following the participants for more than a year after the intervention has ended are needed to be able to draw conclusions about the long‐term benefits, if any, on stress reduction of interventions aimed at reducing stress in healthcare workers. 

Designing interventions to reduce stress amongst high‐risk populations should be preferably based on working mechanisms or underlying biological or behavioural change models.

We found a preliminary indication for a higher standardised mean difference (SMD) when using a wait list control group compared to a non‐intervention control group, which has been corroborated by previous research (Faltinsen 2022). Further research is needed to determine how each control arm could affect the SMD. 

When studying the effect of an intervention focusing on the experience of stress compared to no intervention, wait list control group, placebo, or no stress‐reduction intervention, we recommend future research to have at least 116 participants per study arm at follow‐up. This calculation is based on the SMD of analysis 1.1 (α 0.05, power 80%, difference between two independent means) (Faul 2017). When studying the effect of an intervention focusing away from the experience of stress compared to no intervention, wait list control group, placebo, or no stress‐reduction intervention, we recommend future research to have at least 53 participants per study arm at follow‐up. Again, this calculation is based on the SMD of analysis 1.2 (α 0.05, power 80%, difference between two independent means) (Faul 2017). 

## What's new

**Table:** 

**Date**	**Event**	**Description**
22 May 2023	Amended	Updated acknowledgements to include specifics on NIHR Incentive Award.

## History

Protocol first published: Issue 2, 2000 Review first published: Issue 4, 2006

**Table:** 

**Date**	**Event**	**Description**
12 May 2023	New search has been performed	This review has been updated to include the results of a new search on February 2022. The previous review (Ruotsalainen 2015) has been split into this review on individual‐level interventions and a review on organization‐level interventions (Giga 2018). We used a replacement approach and used the previous review (Ruotsalainen 2015) as one source of studies.
12 May 2023	New citation required and conclusions have changed	In this update, we added 89 studies on top of the 28 related titles identified from the studies in the previous review making 117 total included studies. For this update, we categorized stress‐interventions and outcomes in another way, resulting in new GRADE assessments that are not one‐to‐one comparable to the previous version

## Notes

2023 update: this review update was split from the original title (Ruotsalainen 2015) and now only included individual‐level interventions and added a further 89 studies including 11,119 participants. We implemented several improvements and described these in the section "Differences between protocol and review". For example, we excluded stressors as an outcome. 

## Appendices

### Appendix 1. Search strategy

**Ovid MEDLINE(R)** and Epub Ahead of Print, In‐Process, In‐Data‐Review & Other Non‐Indexed Citations, Daily and Versions(R) <1946 to February 09, 2022>

1 exp Health Personnel/ or "health personnel".mp. or "health care personnel".mp. or "healthcare personnel".mp. or "health care worker*".mp. or "healthcare worker*".mp. or "health worker*".mp. or "health professional*".mp. or "health care professional*".mp. or "healthcare professional*".mp. or "medical care personnel".mp. or nurse?.mp. or physician?.mp. or anesthetist?.mp. or audiologist?.mp. or "dental staff".mp. or "dental personnel".mp. or dentist?.mp. or "medical staff".mp. or "nursing staff".mp. or "nursing personnel".mp. or nutritionist?.mp. or dietitian?.mp. or therapist?.mp. or pharmacist?.mp. or veterinarian?.mp. or "medical personnel".mp. or physiotherapist?.mp. 1469819

2 (care professional? or healthcare professional? or elderly care or nurse? or healthcare worker? or care worker? or physician? or doctor?).mp. 1085871

3 (hospital? or healthcare organi?ation or icu or picu or nursing homes or eol facilit* or "end of life facilit*").ab,kf,ti. 1349587

4 1 or 2 or 3 2647944

5 exp Stress, Psychological/ or "occupational stress".mp. or "occupational strain".mp. or "occupational burden".mp. or "work stress".mp. or "work strain?".mp. or "work burden?".mp. or "work‐related stress".mp. or "work‐related strain?".mp. or "work‐related burden?".mp. or "psychological load?".mp. 147646

6 ((health surveillance adj2 (employee? or worker?)) or (interview* adj5 (employee? or worker?))).ab,kf,ti. 3743

7 (prevent* adj2 (harm* or work or stress)).ab,kf,ti. 10691

8 (((job or work or occupation*) adj2 stress) or compassion fatigue or burnout or burn out or ((strain? or demand*) adj2 (work or job or profession?))).ab,kf,ti. 31680

9 (work abil* or work participa* or work functioning or "functioning at work").ab,kf,ti. 3326

10 ((mental or psych*) adj2 (stress or emotion*)).mp. 149113

11 (anxiety or depression or ((heart or coronary or cardio*) adj2 (disease? or disorder?))).mp. 1328494

12 (autonom* or bullying or bullied or cyberbullying).ab,hw,kf,ti. 202566

13 (decision latitude or decision authority or skill discretion or social support or effort reward).ab,kf,ti. 48057

14 organi?ational justice.ab,kf,ti. 388

15 5 or 6 or 7 or 8 or 9 or 10 or 11 or 12 or 13 or 14 1679887

16 4 and 15 246563

17 exp workplace/ or work*.mp. or job?.mp. or employee*.mp. or occupation*.mp. 2143173

18 (paid work or worker? or vocational or occupation* or presenteeism or employment or employee? or job? or work place or work related or work disabil* or work product* or work limit* or work instabili* or work performance or work capacit or work evaluat* or work direct* or working populat* or workplace).ab,kf,ti. 509144

19 17 or 18 2180713

20 16 and 19 56322

21 exp patients/ or patient*.mp. 7827241

22 20 not 21 28613

23 (randomized controlled trial or controlled clinical trial).pt. 648159

24 (randomized or placebo or randomly or trial or groups).ab. 3176534

25 23 or 24 3322226

26 exp animals/ not humans.sh. 4956807

27 25 not 26 2843767

28 22 and 27 4665

**Scopus**

( ( ( ( ( TITLE‐ABS‐KEY ( ( "health personnel" OR "health care personnel" OR "healthcare personnel" OR "health care worker*" OR "healthcare worker*" OR "health worker*" OR "health professional*" OR "health care professional*" OR "healthcare professional*" OR "medical care personnel" OR nurse? OR physician? OR anesthetist? OR audiologist? OR "dental staff" OR "dental personnel" OR dentist? OR "medical staff" OR "nursing staff" OR "nursing personnel" OR nutritionist? OR dietitian? OR therapist? OR pharmacist? OR veterinarian? OR "medical personnel" OR physiotherapist? ) ) ) OR ( TITLE‐ABS‐KEY ( ( care AND professional? OR healthcare AND professional? OR elderly AND care OR nurse? OR healthcare AND worker? OR care AND worker? OR physician? OR doctor? ) ) ) OR ( TITLE‐ABS‐KEY ( ( hospital? OR healthcare AND organi?ation OR icu OR picu OR nursing AND homes OR eol AND facilit* OR "end of life facilit*" ) ) ) ) AND ( ( TITLE‐ABS‐KEY ( ( stress OR "occupational stress" OR "occupational strain" OR "occupational burden" OR "work stress" OR "work strain?" OR "work burden?" OR "work‐related stress" OR "work‐related strain?" OR "work‐related burden?" OR "psychological load?" ) ) ) OR ( ( TITLE‐ABS‐KEY ( ( health AND surveillance ) AND ( employee? OR worker? ) OR interview* AND ( employee? OR worker? ) ) ) OR ( TITLE‐ABS‐KEY ( ( job OR work OR occupation* ) AND ( stress OR compassion AND fatigue OR burnout ) ) ) OR ( TITLE‐ABS‐KEY ( ( work AND abil* OR work AND participa* OR work AND functioning OR "functioning at work" ) ) ) OR ( TITLE‐ABS‐KEY ( ( autonom* OR bullying OR bullied OR cyberbullying ) ) ) OR ( TITLE‐ABS‐KEY ( ( bullying OR bullied OR cyberbullying ) ) ) OR ( TITLE‐ABS‐KEY ( ( decision AND latitude OR decision AND authority OR skill AND discretion OR effort AND reward OR organizational AND justice ) ) ) ) ) ) AND ( ( TITLE‐ABS‐KEY ( randomized AND controlled AND trial ) ) OR ( TITLE‐ABS‐KEY ( controlled AND clinical AND trial OR random* OR double‐blind OR single‐blind OR ( ( singl* OR doubl* OR trebl* OR tripl* ) AND ( mask* OR blind* ) ) ) ) ) ) AND NOT ( TITLE‐ABS‐KEY ( ( animal AND NOT human ) ) ) ) AND ( TITLE‐ABS‐KEY ( ( work* OR job* OR employee* OR occupation* ) ) )

1,619 results

**Cinahl **

Select / deselect all 

**Table:**

	Search ID#** **	**Search Terms **	**Actions **
	S9	s7 NOT s8	View Results (1,521) View Details Edit
	S8	TI patient*	View Results (727,918) View Details Edit
	S7	S3 AND S6	View Results (1,742) View Details Edit
	S6	AB ("randomized controlled trial*" OR "controlled clinical trial*" OR "controlled trial*" OR random* OR double‐blind OR "double blind") OR TI ("randomized controlled trial*" OR "controlled clinical trial*" OR "controlled trial*" OR random* OR double‐blind OR "double blind")	View Results (416,931) View Details Edit
	S5	S3 AND S4	View Results (7,386) View Details Edit
	S4	TX "randomized controlled trial*" OR "controlled clinical trial*" OR "controlled trial*" OR random* OR double‐blind OR "double blind" OR single‐blind OR "single blind" OR "clinical trial*" OR ( ( singl* OR doubl* OR trebl* OR tripl* ) AND (mask* OR blind* ) )	View Results (1,760,234) View Details Edit
	S3	S1 AND S2	View Results (22,854) View Details Edit
	S2	AB "health care personnel" OR "healthcare personnel" OR "health care worker*" OR "healthcare worker*" OR "health worker*" OR "health professional*" OR "health care professional*" OR "healthcare professional*" OR "medical care personnel" OR nurse? OR physician? OR anesthetist? OR audiologist? OR "dental staff" OR "dental personnel" OR dentist? OR "medical staff" OR "nursing staff" OR "nursing personnel" OR nutritionist? OR dietitian? OR therapist? OR pharmacist? OR veterinarian? OR "medical perso ...	View Results (1,026,684) View Details Edit
	S1	AB stress OR "occupational stress" OR "occupational strain" OR "occupational burden" OR "work stress" OR "work strain?" OR "work burden?" OR "work‐related stress" OR "work‐related strain?" OR "work‐related burden?" OR "psychological load?"	View Results (146,198) View Details Edit

**PsycInfo**

**Table:**

	Search ID#** **	**Search Terms **	**Actions **
	S9	s7 NOT s8	View Results (1,259) View Details Edit
	S8	TI patient*	View Results (183,016) View Details Edit
	S7	S3 AND S6	View Results (1,377) View Details Edit
	S6	AB ("randomized controlled trial*" OR "controlled clinical trial*" OR "controlled trial*" OR random* OR double‐blind OR "double blind") OR TI ("randomized controlled trial*" OR "controlled clinical trial*" OR "controlled trial*" OR random* OR double‐blind OR "double blind")	View Results (235,145) View Details Edit
	S5	S3 AND S4	View Results (1,515) View Details Edit
	S4	TX "randomized controlled trial*" OR "controlled clinical trial*" OR "controlled trial*" OR random* OR double‐blind OR "double blind" OR single‐blind OR "single blind" OR "clinical trial*" OR ( ( singl* OR doubl* OR trebl* OR tripl* ) AND (mask* OR blind* ) )	View Results (287,667) View Details Edit
	S3	S1 AND S2	View Results (20,128) View Details Edit
	S2	AB "health care personnel" OR "healthcare personnel" OR "health care worker*" OR "healthcare worker*" OR "health worker*" OR "health professional*" OR "health care professional*" OR "healthcare professional*" OR "medical care personnel" OR nurse? OR physician? OR anesthetist? OR audiologist? OR "dental staff" OR "dental personnel" OR dentist? OR "medical staff" OR "nursing staff" OR "nursing personnel" OR nutritionist? OR dietitian? OR therapist? OR pharmacist? OR veterinarian? OR "medical perso ...	View Results (323,949) View Details Edit
	S1	AB stress OR "occupational stress" OR "occupational strain" OR "occupational burden" OR "work stress" OR "work strain?" OR "work burden?" OR "work‐related stress" OR "work‐related strain?" OR "work‐related burden?" OR "psychological load?"	View Results (238,644) View Details Edit

**Cochrane Central Register of Controlled Trials**

Issue 2 of 12, February 2022

#276 (health personnel or "health care personnel" or "healthcare personnel" or "health care worker*" or "healthcare worker*" or "health worker*" or "health professional*" or "health care professional*" or "healthcare professional*" or "medical care personnel" or nurse? or physician? or anesthetist? or audiologist? or "dental staff" or "dental personnel" or dentist? or "medical staff" or "nursing staff" or "nursing personnel" or nutritionist? or dietitian? or therapist? or pharmacist? or veterinarian? or "medical personnel" or physiotherapist?):ti,ab,kw 106189

#277 care worker* or nurse* or physician* 87712

#278 #276 or #277 119949

#279 (stress or "occupational stress" or "occupational strain" or "occupational burden" or "work stress" or "work strain?" or "work burden?" or "work‐related stress" or "work‐related strain?" or "work‐related burden?" or "psychological load?"):ti,ab,kw 65145

#280 ((job or work or occupation*) AND (stress or compassion fatigue or burnout )):ti,ab,kw 5695

#281 ((work abil* or work participa* or work functioning or "functioning at work")):ti,ab,kw 21757

#282 (bullying or bullied or cyberbullying):ti,ab,kw 463

#283 ((decision latitude or decision authority or skill discretion or social support or effort reward)):ti,ab,kw 16728

#284 (organizational justice):ti,ab,kw 37

#285 #279 or #280 or #281 or #282 or #283 or #284 97280

#286 #278 and #285 14233

#287 (work* or job? or employee* or occupation*):ti,ab,kw 99224

#288 #286 and #287 8424

#289 (patient*):ti 388757

#290 #288 not #289 7248 limit to Pubmed, Embase and Cinahl results = 4571

**ClinicalTrial.gov**

CONDITION| stress or occupational stress or occupational strain or occupational burden or work stress or work strain or work burden or work‐related stress or work‐related strain or work‐related burden or psychological load AND OTHER TERMS|  health personnel or health care personnel or health care worker or healthcare worker or health worker or health professional or health care professional or healthcare professional or medical care personnel or nurse or physician or medical staff or nursing staff or nursing personnel or medical personnel

**World Health Organization International Clinical Trials Registry Platform**

Basic search: stress AND healthcare OR stress AND health worker OR stress AND nursing personnel OR stress AND medical personnel

## Data and analyses

**Comparison 1:** ‘Focus one’s attention on the experience of stress (thoughts, feelings, behaviour)’ vs No intervention/wait list/placebo/no stress‐reduction intervention (SMD)

Outcome or subgroup title	No. of studies	No. of participants	Statistical method	Effect size
1.1 Any symptoms of stress‐related outcome (follow‐up up to 3 months after the end of the intervention)	41	3645	Std. Mean Difference (IV, Random, 95% CI)	‐0.37 [‐0.52, ‐0.23]
1.1.1 No intervention	29	2434	Std. Mean Difference (IV, Random, 95% CI)	‐0.35 [‐0.53, ‐0.16]
1.1.2 Wait list	9	619	Std. Mean Difference (IV, Random, 95% CI)	‐0.52 [‐0.80, ‐0.25]
1.1.3 Placebo	1	436	Std. Mean Difference (IV, Random, 95% CI)	‐0.02 [‐0.21, 0.16]
1.1.4 No stress‐reduction intervention	2	156	Std. Mean Difference (IV, Random, 95% CI)	‐0.33 [‐1.19, 0.53]
1.2 Any symptoms of stress‐related outcome (follow‐up > 3 to 12 months after the end of the intervention)	19	1851	Std. Mean Difference (IV, Random, 95% CI)	‐0.43 [‐0.71, ‐0.14]
1.2.1 No intervention	11	1271	Std. Mean Difference (IV, Random, 95% CI)	‐0.46 [‐0.90, ‐0.02]
1.2.2 Wait list	6	480	Std. Mean Difference (IV, Random, 95% CI)	‐0.53 [‐0.83, ‐0.24]
1.2.3 No stress reduction intervention	2	100	Std. Mean Difference (IV, Random, 95% CI)	0.16 [‐0.23, 0.56]
1.3 Any symptoms of stress‐related outcome (follow‐up >12 months)	1		Mean Difference (IV, Random, 95% CI)	Totals not selected
1.4 Psychological symptoms: anxiety and depression (follow‐up up to 3 months after the end of the intervention)	8	742	Std. Mean Difference (IV, Random, 95% CI)	‐0.27 [‐0.58, 0.03]
1.4.1 No intervention	7	482	Std. Mean Difference (IV, Random, 95% CI)	‐0.28 [‐0.68, 0.11]
1.4.2 Wait list	1	260	Std. Mean Difference (IV, Random, 95% CI)	‐0.28 [‐0.53, ‐0.04]
1.5 Psychological symptoms: anxiety and depression (follow‐up > 3 to 12 months after the end of the intervention)	3		Std. Mean Difference (IV, Random, 95% CI)	Totals not selected
1.5.1 No intervention	3		Std. Mean Difference (IV, Random, 95% CI)	Totals not selected
1.6 Explanatory analysis ‐ subgroup (short vs long duration of intervention): any symptoms of stress‐related outcome (follow‐up up to 3 months after the end of the intervention)	41	3645	Std. Mean Difference (IV, Random, 95% CI)	‐0.37 [‐0.52, ‐0.23]
1.6.1 Short (< 12weeks)	29	2647	Std. Mean Difference (IV, Random, 95% CI)	‐0.32 [‐0.49, ‐0.15]
1.6.2 Long (≥ 12 weeks)	12	998	Std. Mean Difference (IV, Random, 95% CI)	‐0.50 [‐0.78, ‐0.22]

**Comparison 2:** ‘Focus one’s attention away from the experience of stress by means of relaxation, exercise or something else’ vs No intervention/wait list/placebo/no stress‐reduction intervention (SMD)

Outcome or subgroup title	No. of studies	No. of participants	Statistical method	Effect size
2.1 Any symptoms of stress‐related outcome (follow‐up up to 3 months after the end of the intervention)	35	2366	Std. Mean Difference (IV, Random, 95% CI)	‐0.55 [‐0.70, ‐0.40]
2.1.1 No intervention	22	1565	Std. Mean Difference (IV, Random, 95% CI)	‐0.48 [‐0.61, ‐0.35]
2.1.2 Wait list	8	429	Std. Mean Difference (IV, Random, 95% CI)	‐0.88 [‐1.53, ‐0.24]
2.1.3 Placebo	3	168	Std. Mean Difference (IV, Random, 95% CI)	‐0.43 [‐0.74, ‐0.12]
2.1.4 No stress‐reduction intervention	2	204	Std. Mean Difference (IV, Random, 95% CI)	‐0.27 [‐0.55, 0.00]
2.2 Any symptoms of stress‐related outcome (follow‐up > 3 to 12 months after the end of the intervention)	6	427	Std. Mean Difference (IV, Random, 95% CI)	‐0.41 [‐0.79, ‐0.03]
2.2.1 No intervention	4	312	Std. Mean Difference (IV, Random, 95% CI)	‐0.43 [‐0.98, 0.12]
2.2.2 Wait list	2	115	Std. Mean Difference (IV, Random, 95% CI)	‐0.40 [‐0.77, ‐0.03]
2.3 Psychological symptoms: anxiety and depression (follow‐up up to 3 months after the end of the intervention)	7	378	Std. Mean Difference (IV, Random, 95% CI)	‐1.07 [‐1.95, ‐0.19]
2.3.1 No intervention	2	127	Std. Mean Difference (IV, Random, 95% CI)	‐0.67 [‐1.60, 0.25]
2.3.2 Wait list	3	173	Std. Mean Difference (IV, Random, 95% CI)	‐1.89 [‐4.24, 0.46]
2.3.3 Placebo	2	78	Std. Mean Difference (IV, Random, 95% CI)	‐0.34 [‐0.79, 0.11]
2.4 Explanatory analysis ‐ subgroup (short vs long duration of intervention): any symptoms of stress‐related outcome (follow‐up up to 3 months after the end of the intervention)	35	2366	Std. Mean Difference (IV, Random, 95% CI)	‐0.55 [‐0.70, ‐0.40]
2.4.1 Short (<12 weeks)	33	2255	Std. Mean Difference (IV, Random, 95% CI)	‐0.52 [‐0.68, ‐0.37]
2.4.2 Long (≥ 12 weeks)	2	111	Std. Mean Difference (IV, Random, 95% CI)	‐0.96 [‐1.48, ‐0.43]

**Comparison 3:** ‘Focus on work‐related risk factors on an individual level such as work demands’ vs No intervention/no stress‐reduction intervention (SMD)

Outcome or subgroup title	No. of studies	No. of participants	Statistical method	Effect size
3.1 Any symptoms of stress‐related outcome (follow‐up up to 3 months after the end of the intervention)	3		Std. Mean Difference (IV, Random, 95% CI)	Totals not selected
3.1.1 No intervention	2		Std. Mean Difference (IV, Random, 95% CI)	Totals not selected
3.1.2 No stress‐reduction intervention	1		Std. Mean Difference (IV, Random, 95% CI)	Totals not selected
3.2 Any symptoms of stress‐related outcome (follow‐up > 3 to 12 months after the end of the intervention)	2		Std. Mean Difference (IV, Random, 95% CI)	Totals not selected
3.3 Any symptoms of stress‐related outcome (follow‐up >12 months)	1		Mean Difference (IV, Random, 95% CI)	Totals not selected
3.4 Psychological symptoms: anxiety and depression (follow‐up > 3 to 12 months after the end of the intervention)	1		Mean Difference (IV, Random, 95% CI)	Totals not selected

**Comparison 4:** ‘Combination of interventions’ vs No intervention/wait list/no stress‐reduction intervention (SMD)

Outcome or subgroup title	No. of studies	No. of participants	Statistical method	Effect size
4.1 Any symptoms of stress‐related outcome (follow‐up up to 3 months after the end of the intervention)	15	1003	Std. Mean Difference (IV, Random, 95% CI)	‐0.67 [‐0.95, ‐0.39]
4.1.1 No intervention	10	666	Std. Mean Difference (IV, Random, 95% CI)	‐0.71 [‐1.08, ‐0.34]
4.1.2 Wait list	4	270	Std. Mean Difference (IV, Random, 95% CI)	‐0.79 [‐1.21, ‐0.38]
4.1.3 No stress‐reduction intervention	1	67	Std. Mean Difference (IV, Random, 95% CI)	0.13 [‐0.35, 0.61]
4.2 Any symptoms of stress‐related outcome (follow‐up > 3 to 12 months after the end of the intervention)	6	574	Std. Mean Difference (IV, Random, 95% CI)	‐0.48 [‐0.95, ‐0.00]
4.2.1 No intervention	3	330	Std. Mean Difference (IV, Random, 95% CI)	‐0.70 [‐1.52, 0.12]
4.2.2 Wait list	2	177	Std. Mean Difference (IV, Random, 95% CI)	‐0.36 [‐0.65, ‐0.06]
4.2.3 No stress‐reduction intervention	1	67	Std. Mean Difference (IV, Random, 95% CI)	0.00 [‐0.48, 0.48]
4.3 Any symptoms of stress‐related outcome (follow‐up > 12 months)	1		Mean Difference (IV, Random, 95% CI)	Totals not selected
4.4 Psychological symptoms: anxiety and depression (follow‐up up to 3 months after the end of the intervention)	4		Std. Mean Difference (IV, Random, 95% CI)	Totals not selected
4.4.1 No intervention	3		Std. Mean Difference (IV, Random, 95% CI)	Totals not selected
4.4.2 Wait list	1		Std. Mean Difference (IV, Random, 95% CI)	Totals not selected
4.5 Psychological symptoms: anxiety and depression (follow‐up > 3 to 12 months after the end of the intervention)	1		Mean Difference (IV, Random, 95% CI)	Totals not selected
4.6 Psychological symptoms: anxiety and depression (follow‐up > 12 months after the end of the intervention)	1		Mean Difference (IV, Random, 95% CI)	Totals not selected

**Comparison 5:** Focus one’s attention on the experience of stress vs focus one’s attention away from the experience of stress

Outcome or subgroup title	No. of studies	No. of participants	Statistical method	Effect size
5.1 Any symptoms of stress‐related outcome (follow‐up up to 3 months)	3		Std. Mean Difference (IV, Random, 95% CI)	Totals not selected
5.2 Any symptoms of stress‐related outcome (follow‐up > 3 to 12 months after the end of the intervention)	2		Std. Mean Difference (IV, Random, 95% CI)	Totals not selected
5.3 Psychological symptoms: anxiety and depression (follow‐up up to 3 months)	1		Std. Mean Difference (IV, Random, 95% CI)	Totals not selected
5.4 Psychological symptoms: anxiety and depression (follow‐up > 3 to 12 months after the end of the intervention)	1	38	Std. Mean Difference (IV, Random, 95% CI)	0.24 [‐0.39, 0.88]

**Comparison 6:** ‘Combination of interventions’ vs focus one's attention on the experience of stress (SMD)

Outcome or subgroup title	No. of studies	No. of participants	Statistical method	Effect size
6.1 Any symptoms of stress‐related outcome (follow‐up up to 3 months)	1		Mean Difference (IV, Random, 95% CI)	Totals not selected
6.2 Any symptoms of stress‐related outcome (follow‐up > 3 to 12 months after the end of the intervention)	1		Mean Difference (IV, Random, 95% CI)	Totals not selected

## Characteristics of studies

### Characteristics of included studies [ordered by study ID]

**Table:** Alexander 2015

***Study characteristics***		
Methods	**Study design:** randomised controlled trial **Study grouping:** parallel group	
Participants	**Baseline characteristics** Yoga *Age (mean ± SD)*: NR *Sex (N (% female))*: NR *Sample size*: 20 *Years of experience (mean ± SD)*: NR Control (No intervention) *Age (mean ± SD)*: NR *Sex (N (% female))*: NR *Sample size*: 20 *Years of experience (mean ± SD)*: NR Overall *Age (mean ± SD)*: 46.38 ± 10.23 *Sex (N (% female))*: 39 (98%) *Sample size*: 40 *Years of experience (mean ± SD)*: 14.21 ± 11.02 **Included criteria:** no prior experience with yoga, willingness to complete eight weekly sessions and homework exercises, and willingness to be randomly assigned to the research or control group. **Excluded criteria:** serious illness or major orthopaedic diagnoses of the neck, back, pelvis, or lower extremities that could interfere with completion of the yoga intervention protocol. **Pretreatment:** NR **Compliance rate:** NR **Response rate:** NR **Type of healthcare worker:** exclusively nurses	
Interventions	**Intervention characteristics** Yoga *Type of the intervention*: Intervention type 2 ‐ to focus one’s attention away from the experience of stress *Description of the intervention*: In early yoga sessions, participants learned to become conscious of their breathing. Breathing is both a conscious and unconscious process and therefore gives conscious access to the autonomic nervous system. Inhalation stimulates the sympathetic nervous system, while exhalation stimulates the parasympathetic nervous system. When one inhales, heart rate increases and when one exhales, heart rate decreases. Practising mindful breathing allows individuals to calm the body and mind immediately, thereby decreasing stress or energising the nervous system if one feels fatigued or depressed (Burg & Michalak, 2011; Mason et al., 2013). Throughout the intervention, the instructor taught participants the basics of postural alignment, deep breathing, and monitoring the mind with simple meditations. Each session concluded with deep relaxation. Each participant received handouts for each session to provide further information and a visual reminder of the exercises, the basis for cultivating a home practice. As the series progressed, additional exercises, breathing practices, and meditations were added to expose participants to the wide range of movements that can work not only the skeletal muscles but also other body systems such as the internal organs, nervous system, circulation, and emotions. *The number of sessions*: eight *Duration of each session on average*: NR *Duration of the entire intervention*: eight weeks *Duration of the entire intervention short vs long*: Short *Intervention deliverer*: Experienced yoga instructor, who is an osteopathic physician in the local community *Intervention form*: Group Control (no intervention) *Type of the intervention*: NA *Description of the intervention*: NA *The number of sessions*: NA *Duration of each session on average*: NA *Duration of the entire intervention*: NA *Duration of the entire intervention short vs long*: NA *Intervention deliverer*: NA *Intervention form*: NA	
Outcomes	MBI **Outcome type**: ContinuousOutcome *Health Promoting Lifestyle Profile II (HPLP II)* **Outcome type**: ContinuousOutcome	
Identification	**Sponsorship source:** The author(s) disclosed receipt of the following financial support for the research, authorship, and/or publication of this article: This research was supported by the Research and Creative Activities Fund of Texas Christian University. **Country:** USA **Setting:** Hospital **Comments:** NR **Authors name:** Gina K. Alexander **Institution:** Texas Christian University, Harris College of Nursing and Health Sciences **Email:** g.alexander@tcu.edu **Address:** TCU Box 298620, Fort Worth, TX 76129, USA **Time period:** NR	
Notes	MBI‐EE included in analysis 2.1	
***Risk of bias***		
**Bias**	**Authors' judgement**	**Support for judgement**
Random sequence generation (selection bias)	Unclear risk	Questionnaire, and core study questionnaires. After individuals completed consent forms and baseline assessments, they were enroled in the study and randomized to the intervention (yoga) or usual care control group. A total of 54 individuals Sequence generation process not mentioned
Allocation concealment (selection bias)	Unclear risk	Insufficient information to understand whether intervention allocations could have been foreseen in advance of, during, enrolment.
Blinding of participants and personnel (performance bias) All outcomes	High risk	Participants were not blinded.
Blinding of outcome assessment (detection bias) All outcomes	High risk	Participants were not blinded whereas outcomes are self‐reported
Incomplete outcome data (attrition bias) All outcomes	Unclear risk	Not reported.
Selective reporting (reporting bias)	Unclear risk	No trial registration or study protocol reported in the study and we did not find one online. No indication of selective reporting.
Other bias	Unclear risk	Compliance rate and response rate not reported.

**Table:** Amutio 2015

***Study characteristics***		
Methods	**Study design:** randomised controlled trial **Study grouping:** parallel group	
Participants	**Baseline characteristics** Mindfulness training *Age in years (mean ± SD)*: NR *Sex (N (% female))*: NR *Sample size*: 21 *Years of experience (> 10 years)*: NR Control (wait list) *Age in years (mean ± SD)*: NR *Sex (N (% female))*: NR *Sample size*: 21 *Years of experience (> 10 years)*: NR Overall *Age in years (mean ± SD)*: 47.3 ± 9.4 *Sex (N (% female))*: 24 (57%) *Sample size*: 42 *Years of experience (> 10 years)*: 28 (67%) **Included criteria:** willingness to complete the questionnaires and commitment to adhere to the programmes' attendance and dedication requirements. **Excluded criteria:** were being in psychiatric or psychological treatment, or not being actively practising at the time of the study. **Pretreatment:** no initial differences between groups were found for the main variables of our study (mindfulness, F = 2.51, P = 0.12; burnout, F = 1.11, P = 0.30; and emotional exhaustion, F = 2.87, P = 0.10), including demographic or professional characteristics (P > 0.05) **Type of healthcare worker:** exclusively physicians **Response rate:** NR **Compliance rate:** regarding acceptability of the intervention to participants, the attendance rates for the two phases of the program were 88% for weekly sessions and 72% for monthly sessions	
Interventions	**Intervention characteristics** Mindfulness training *Type of the intervention*: Intervention type 1 ‐ to focus one’s attention on the experience of stress *Description of the intervention*: The sessions were taught following the standardised MBSR protocol. Twenty‐minute PowerPoint presentation of a particular topic related to the medical profession (e.g. dealing with suffering, interpersonal relationships). A 45‐minute mindfulness exercise (body‐scan, yoga stretches, and meditation—i.e. breathing, observing thoughts, walking meditation). A 60‐minute group reflection about the weekly topic and the experiences with the mindfulness practice. This included Krasner's narrative and appreciative enquiry exercises. Dedicated time to record HR and BP at the beginning and end of each session. Additionally, participants were asked to practice mindfulness exercises every day for a period of 45 minutes by means of a set of CDs distributed to them and containing the same exercises as the ones practised in the class sessions. They were also required to register the number of days practised per week and the length of each of the sessions in minutes by means of a record sheet specially designed for that purpose *The number of sessions*: 9 *Duration of each session on average*: 2.5 hours + 45 min homework *Duration of the entire intervention*: 8 weeks *Duration of the entire intervention short vs long*: Short *Intervention deliverer*: MBSR instructor who was trained by Kabat‐Zinn at the Stress Reduction Clinic in the University of Massachusetts (USA) *Intervention form*: Group + homework Control (wait list) *Type of the intervention*: wait list *Description of the intervention*: The wait list control group was told that a similar course would be offered again. *The number of sessions*: NA *Duration of each session on average*: NA *Duration of the entire intervention*: NA *Duration of the entire intervention short vs long*: NA *Intervention deliverer*: NA *Intervention form*: NA	
Outcomes	*Maslach Burnout Inventory ‐ Emotional Exhaustion* **Outcome type**: ContinuousOutcome *Maslach Burnout Inventory ‐ Depersonalisation* **Outcome type**: ContinuousOutcome *Maslach Burnout Inventory ‐ Personal accomplishment (lack of)* **Outcome type**: ContinuousOutcome	
Identification	**Sponsorship source:** The authors report the University of the Basque Country (UPV/EHU)provided funding for the materials and travel expenses. The Official Medical College of Biscay in Spain provided the physical setting to conduct the sessions. **Country:** Spain **Setting:** all participants were actively used in public (42.9%) or private (52.4%) practice. **Comments:** NR **Authors name:** Alberto Amutio, PhD; **Institution:** Department of Social Psychology and Methodology of the BehavioralSciences, Faculty of Psychology, University of the Basque Country (UPV/EHU), Spain **Email:** Alberto.amutio@ehu.eus **Address:** Avda Tolosa, 70, Donostia‐San Sebastian, Gipuzko **Time period:** NR	
Notes	MBI‐EE included in analysis 1.1	
***Risk of bias***		
**Bias**	**Authors' judgement**	**Support for judgement**
Random sequence generation (selection bias)	Low risk	Quote: "Participants in the experimental group (n = 21) were randomly selected using the statistical program SPSS 20.0. The remaining subjects were included in the control group (n = 21)."
Allocation concealment (selection bias)	Unclear risk	Judgement Comment: Unable to judge whether participants and/or investigators could possibly foresee assignment
Blinding of participants and personnel (performance bias) All outcomes	High risk	Quote: "Each participant in the experimental group committed to attending the sessions, doing the exercises assigned as home‐work, and answering the evaluation questions at the end of each of the phases of the study. The waitlist control group was told that a similar course would be offered again." Judgement Comment: Participants not blinded
Blinding of outcome assessment (detection bias) All outcomes	High risk	Participants were not blinded whereas outcomes are self‐reported
Incomplete outcome data (attrition bias) All outcomes	Unclear risk	not explicitly stated whether participants dropped out.
Selective reporting (reporting bias)	Unclear risk	No trial registration or no study protocol reported, nor did we find one online.
Other bias	Low risk	No indication of other sources of bias

**Table:** Aranda Ausern 2016

***Study characteristics***		
Methods	**Study design:** randomised controlled trial **Study grouping:** parallel group	
Participants	**Baseline characteristics** Control (no intervention) *Age in years (mean ± SD)*: 49.9 (8.7) *Sex (N (% female))*: 21 (95.5%) *Sample size*: 22 *Years of experience (mean ± SD)*: 24.0 (10.8) Mindfulness and self‐compassion program *Age in years (mean ± SD)*: 50.0 (7.9) *Sex (N (% female))*: 17 (73.9%) *Sample size*: 23 *Years of experience (mean ± SD)*: 24.0 (8.0) Overall *Age in years (mean ± SD)*: 4.9 (8.2) *Sex (N (% female))*: 38 (84.4%) *Sample size*: 45 *Years of experience (mean ± SD)*: 24.0 (9.3) **Included criteria:** informed consent, committing to completing the pre‐ and post‐intervention questionnaires, attending at least 75% of the sessions and practising mindfulness and self‐compassion for 45 minutes a day. **Excluded criteria:** having completed a mindfulness and/or compassion program in the previous 6 months; having a psychiatric illness that did not make participation in the study advisable. **Pretreatment:** they were not significant in any of the characteristics considered. **Type of healthcare worker:** various healthcare staff but 46.7% nurses and physicians 53.3% **Response rate:** NR **Compliance rate:** Intervention group 92% and control group 92%	
Interventions	**Intervention characteristics** Control (no intervention) *Type of the intervention*: NA *Description of the intervention*: NA *Number of sessions*: NA *Duration of each session*: NA *Duration of the entire intervention*: NA *Duration of the entire intervention short vs long*: NA *Intervention deliverer*: NA *Intervention form*: NA Mindfulness and self‐compassion program *Type of the intervention*: Intervention type 2 ‐ to focus one’s attention away from the experience of stress *Description of the intervention*: Each session dealt with a specific topic and included mindfulness and self‐compassion practices, with time for dialogue and exchange of experiences among the participants. Material was provided for the practices at home (manual of theoretical contents, audios and practice diaries). Learning how to become conscious of one's breathing. Throughout the intervention, the instructor taught participants the basics of postural alignment, deep breathing, and monitoring the mind with simple meditations. Each session concluded with deep relaxation. *Number of sessions*: 8 sessions *Duration of each session*: 2.5 hours each *Duration of the entire intervention*: 8 weeks *Duration of the entire intervention short vs long*: short *Intervention deliverer*: It was taught by an instructor with a master's degree in Mindfulness and trained in the MBSR and MSC programs. *Intervention form*: group	
Outcomes	*Perceived Stress Questionnaire (PSQ)* **Outcome type**: ContinuousOutcome **Reporting**: Fully reported **Direction**: Lower is better **Data value**: Endpoint	
Identification	**Sponsorship source:** This work has been partially financed by the Department of Health of the Government of Navarra, by obtaining the first prize in the II Contest of Ideas for Health Research in Primary Care. **Country:** Spain **Setting:** NR **Comments:** NR **Authors name:** Aranda Auserón **Institution:** Subdirección de Farmacia, Servicio Navarro de Salud‐Osasunbidea (SNS‐O), Pamplona, Spain **Email:** garandaa@navarra.es **Address:** NR	
Notes	PSS included in analysis 2.1	
***Risk of bias***		
**Bias**	**Authors' judgement**	**Support for judgement**
Random sequence generation (selection bias)	Low risk	The randomization of the groups was carried out by assigning correlative numbers to the 48 participants and selecting a total of 25 from a balloon with 48 numbered balls; these numbers were part of the intervention group, with the rest remaining in the control group.
Allocation concealment (selection bias)	Unclear risk	Insufficient information to understand whether intervention allocations could have been foreseen in advance of, during, enrolment.
Blinding of participants and personnel (performance bias) All outcomes	High risk	Participants not blinded.
Blinding of outcome assessment (detection bias) All outcomes	High risk	Participants not blinded whereas outcomes are self‐reported
Incomplete outcome data (attrition bias) All outcomes	Low risk	3 of the 48 (16%) randomised participants were lost to follow‐up, which is below our pre‐defined cut‐off point.
Selective reporting (reporting bias)	Unclear risk	No trial protocol or registration mentioned in the study nor did we find one online.
Other bias	Unclear risk	Low participation rate in the study (48 of 1281; 3.75%)

**Table:** Axisa 2019

***Study characteristics***		
Methods	**Study design:** randomised controlled trial **Study grouping:** parallel group	
Participants	**Baseline characteristics** Evaluation of a well‐being workshop *Age 25 to 29 30 to 34 35 to 44*: 12 (52) 5 (22) 6 (26) *Sex ((N) % female)*: 16 (70%) *Sample size*: 23 *Years of experience (mean ± SD)*: NR Control (no intervention) *Age 25 tp 29 30 to 34 35 to 44*: 16 (70) 4 (17) 3 (13) *Sex ((N) % female)*: 18 (78%) *Sample size*: 23 *Years of experience (mean ± SD)*: NR Overall *Age 25 to 29 30 to 34 35 to 44*: 28 (61) 9 (20) 9 (20) *Sex ((N) % female)*: 34 (74%) *Sample size*: 46 *Years of experience (mean ± SD)*: NR **Included criteria:** recruitment was restricted to physician trainees completing their RACP basic physician training in New South Wales (NSW) hospitals. **Excluded criteria:** NR **Pretreatment:** differences between groups at 3 and 6 months were assessed using linear regression models, with group as a covariate, and adjusted for the participants’ baseline value of the outcome measure. **Compliance rate:** not explicitly reported "Lack of control over work rosters, difficulty swapping shifts, being on call or studying for the RACP exams, were major factors influencing the intervention group participant attendance at workshops" **Response rate:** 88% **Type of healthcare worker:** exclusively physician trainees	
Interventions	**Intervention characteristics** Evaluation of a wellbeing workshop *Type of the intervention*: Intervention type 1 ‐ to focus one’s attention on the experience of stress *Description of the intervention*: following review of the international literature, a workshop was developed in consultation with local experts to promote health and wellbeing for physician trainees. The workshop objectives were to outline strategies for wellbeing and stress management and to encourage participants to apply these strategies in their own lives. The workshop incorporated case studies specifically developed for physician trainees, a holistic well‐being framework and group work activities to encourage discussion about approaches to work, life and self‐care. Some topics in the workshop included stressors relating to work–life balance, understanding well‐being and resilience, mindfulness, barriers to looking after well‐being, giving and receiving feedback and stress management strategies. *The number of sessions*: 1 *Duration of each session on average*: 4.5 hours including 1‐hour break *Duration of the entire intervention*: 4.5 hours *Duration of the entire intervention short vs long*: Short *Intervention deliverer*: Workshops were facilitated by specialist clinicians who received specific training to facilitate the workshops *Intervention form*: Group Control (no intervention) *Type of the intervention*: NA *Description of the intervention*: NA *The number of sessions*: NA *Duration of each session on average*: NA *Duration of the entire intervention*: NA *Duration of the entire intervention short vs long*: NA *Intervention deliverer*: NA *Intervention form*: NA	
Outcomes	*ProQOL ‐ Burnout* **Outcome type**: ContinuousOutcome *DASS ‐ stress* **Outcome type**: ContinuousOutcome *DASS ‐ Anxiety* **Outcome type**: ContinuousOutcome *DASS ‐ Depression* **Outcome type**: ContinuousOutcome	
Identification	**Sponsorship source:** The authors received no financial support for the research, authorship, and/or publication of this article **Country:** Australian **Setting:** Several hospitals **Comments:** NR **Authors name:** Carmen Axisa **Institution:** University of Technology Sydney, Sydney, NSW, and; PhD Candidate, Sydney Medical School, The University of Sydney, Sydney, NSW, Australia **Email:** carmen.axisa@uts.edu.au **Address:** University of Technology Sydney, Building 10, Level 7, 235 Jones St, Ultimo, Sydney, NSW 2007, Australia. **Time period:** 2014‐105	
Notes	DASS‐stress included in analysis 1.1 and 1.2 DASS‐Anxiety included in analysis 1.4 and 1.5	
***Risk of bias***		
**Bias**	**Authors' judgement**	**Support for judgement**
Random sequence generation (selection bias)	Low risk	Quote: "Participants were randomly assigned to the intervention or control group using a web‐based True Random Number Generator service."
Allocation concealment (selection bias)	Unclear risk	Unable to judge whether participants and/or investigators could possibly foresee assignment
Blinding of participants and personnel (performance bias) All outcomes	High risk	Participants were not blinded.
Blinding of outcome assessment (detection bias) All outcomes	High risk	Participants were not blinded whereas outcomes are self‐reported
Incomplete outcome data (attrition bias) All outcomes	High risk	46 of the 59 (78%) randomised participants included in the analyses. Not reported whether lost to follow‐up was at random.
Selective reporting (reporting bias)	Low risk	No trial registration or no study protocol reported. No indication of selective reporting.
Other bias	Unclear risk	Compliance difficult to assess.

**Table:** Bagheri 2019

***Study characteristics***		
Methods	**Study design:** randomised controlled trial **Study grouping:** parallel group	
Participants	**Baseline characteristics** Cognitive‐behavioural therapy + relaxation *Age in years (mean ± SD)*: NR *Sex (N (% female))*: NR *Sample size*: 30 *Years of experience (mean ± SD)*: NR Control (no intervention) *Age in years (mean ± SD)*: NR *Sex (N (% female))*: NR *Sample size*: 30 *Years of experience (mean ± SD)*: NR Overall *Age in years (mean ± SD)*: 33.21 ± 7.04 *Sex (N (% female))*: 52 (88%) *Sample size*: 60 *Years of experience (mean ± SD)*: NR **Included criteria:** nurses of both sexes, undergraduate and postgraduate education, all age groups, more than one year experience of clinical work, and working in different wards. **Excluded criteria:** having a chronic physical and psychological illness, taking drugs that affected the mental system, and loss of a first‐degree relative (father, mother, spouse or child) less than six months beforehand. It was also announced that one of the admission requirements was the principle of confinement to educational materials and issues raised by the group members in each session. **Pretreatment:** age and the variable burnout and its subscales before intervention were not significantly different in the two groups. **Compliance rate:** NR **Response rate:** NR **Type of healthcare worker:** various healthcare professionals including nurses 44 (75%), head nurses 10 (17%) and supervisors 5 (9%)	
Interventions	**Intervention characteristics** Cognitive‐behavioural therapy + relaxation *Type of the intervention*: Intervention type 1 ‐ to focus one’s attention on the experience of stress *Description of the intervention*: at the beginning of each session, the objectives of the meeting were discussed, followed by a summing up of the previous meeting. A few minutes were then allocated to examining the homework from the previous meeting. The total therapeutic goals of the sessions were as follows: Session One: provides participants with the information that physiological, cognitive and behavioural processes interact with each other, that emotions have cognitive, physiological and behavioural components, and that all or most emotional responses have cognitive components; Session Two: empowers participants to receive the preliminary thoughts between the event and the emotional response and to write them in three columns, (I) the activating event, (II) beliefs or thoughts, and (III) emotional outcomes; Session Three: participants recognise the most important aspects of the cognitive theories of depression, anxiety and anger, become familiar with the characteristics of happy thoughts and how they can be reached, identify important cognitive distortions and discover their ability to recognise them in their thoughts. Finally, they must recognise their potential resistance to cognitive therapy, and learn strategies to deal with this resistance; Session Four: participants become acquainted with the point that their thoughts, just like emotional outcomes, have behavioural consequences, and that also their behavioural consequences may be ineffective; Session Five: focus' on the nature of schemas (central beliefs, thoughts, inefficient attitudes), and the relationship between schemas and happy thoughts, as well as on downward arrows for identifying schemas; Session Six: participants accept the principle that beliefs are volatile. Dominant cultural beliefs change throughout human history, and individuals also change their beliefs over time; Session Seven: focus' on the understanding that beliefs can be evaluated based on a number of criteria, that beliefs can have different degrees of efficiency, and that individuals use a set of beliefs to organise their behaviours that are, to some extent, consistent and compatible with other beliefs held by others. Consistency with other beliefs and the beliefs of other people has an implicit implication on the correctness of that belief. Session Eight: participants can apply a logical analysis to their beliefs. Logical analysis is the strongest technique for challenging people’s beliefs. An important aspect of logical analysis is that it has multiple ways of challenging beliefs in itself. Therefore, in this way, through the challenge to their beliefs participants tend to conclude that their beliefs are true or false. Session Nine: participants can “oppose” their negative beliefs, and in Session Ten they can create a practical application for themselves that will encourage them to continue practising techniques and approaches they have learnt throughout the program, and provide a program for continuity and sustainability of alteration. In order to achieve the proposed therapeutic goals, cognitive behavioural methods are taught to them. *The number of sessions*: 10 *Duration of each session on average*: 1.5 to 2 hours *Duration of the entire intervention*: 2.5 months *Duration of the entire intervention short vs long*: Short *Intervention deliverer*: Clinical psychiatrist with a Master degree. *Intervention form*: Group, face‐to‐face Control (no intervention) *Type of the intervention*: NA *Description of the intervention*: Meanwhile, no psychological intervention was performed on the control group. *The number of sessions*: NA *Duration of each session on average*: NA *Duration of the entire intervention*: NA *Duration of the entire intervention short vs long*: NA *Intervention deliverer*: NA *Intervention form*: NA	
Outcomes	*Maslach Burnout Inventory ‐ Emotional Exhaustion* **Outcome type**: ContinuousOutcome *Maslach Burnout Inventory ‐ Depersonalisation* **Outcome type**: ContinuousOutcome *Maslach Burnout Inventory ‐ Personal accomplishment (lack of)* **Outcome type**: ContinuousOutcome	
Identification	**Sponsorship source:** NR **Country:** Iran **Setting:** Hospital **Comments:** NR **Authors name:** Bagheri T **Institution:** Burn Research Centre, Iran University of Medical Sciences, Tehran, **Email:** mahnoushmomeni@gmail.com, momeni.m@iums.ac.ir **Address:** Burn Research Centre, Motahari Hospital, Rashid Yasami St, Vali‐e‐asr Ave, Tehran, Iran **Time period:** 2014	
Notes	MBI‐EE included in analysis 1.1	
***Risk of bias***		
**Bias**	**Authors' judgement**	**Support for judgement**
Random sequence generation (selection bias)	Unclear risk	Quote: "They were assigned by the block randomization method to two groups of 30 subjects." Unclear how randomization took place.
Allocation concealment (selection bias)	Unclear risk	Quote: "Participants in the study included all nurses, head nurses and supervisors who met the inclusion criteria. They were assigned by the block randomization method to two groups of 30 subjects." Unable to judge whether participants or researchers could foresee the outcome of block randomization.
Blinding of participants and personnel (performance bias) All outcomes	High risk	Participants not blinded.
Blinding of outcome assessment (detection bias) All outcomes	High risk	Participants were not blinded whereas outcomes are self‐reported.
Incomplete outcome data (attrition bias) All outcomes	Unclear risk	Not described.
Selective reporting (reporting bias)	High risk	Quote: "their annual evaluation. Outcome measures: A demographic questionnaire was used to collect personal, social and occupational data including age, sex, marital status, educational level, work experience, overtime worked per month, work area, work shift, number of children and economic status. Burnout in all participants in the study was determined by the Maslach Burnout Questionnaire. Maslach Burnout Questionnaire (MBQ). The Maslach Burnout Questionnaire has 22 items which measure burnout in the three dimensions of emotional exhaustion (9 questions), personality depersonalization (5 questions), and individual performance (8 questions). In order to determine the total burnout score, questions 1, 2, 3, 5, 6, 9, 10, 12, 13, 14, 15, 19, 21 and 22 are considered (+) and questions 4, 7, 8, 11, 16, 17, 18 and 20 (‐), and then aggregated. Results for burnout frequency will be 35 to 84 (high), ‐15 to 34 (average), ‐16 to ‐66 (low) and for burnout severity 40 to 98 (high), ‐18 to 39 (average) and ‐19 to ‐77 (low). The validity of the questionnaire was verified by Maslach and Jackson and its reliability was calculated through Cronbach’s alpha, which was reported between ‐0.60 and 0.08. In Sedghi’s research, the reliability was determined to be 0.78. 20 The Cronbach’s alpha was reported as 0.8 in the present study. Data collection. In the second stage, the nurses in the intervention group received group cognitive therapy. In the third stage, immediately after and one month after training completion, the burnout level of all participants in the study was determined and evaluated by the Maslach burnout inventory. Data analysis. Data were extracted" In the trial register it is mentioned that the primary outcome is the Job Stress Questionnaire and the General Health questionnaire a secondary outcome while those have not been reported. https://en.irct.ir/trial/8633
Other bias	Unclear risk	The response rate nor the compliance rate have been reported.

**Table:** Barattucci 2019

***Study characteristics***		
Methods	**Study design:** randomised controlled trial **Study grouping:** parallel group	
Participants	**Baseline characteristics** Mindfulness‐Based IARA Model® *Age in years (mean ± SD)*: 44.1 ± 9.5 *Sex (N (% female))*: NR *Sample size*: 295 *Years of experience (mean ± SD)*: 10.8 ± 10.3 Control (no intervention) *Age in years (mean ± SD)*: 45.2 ± 10.3 *Sex (N (% female))*: NR *Sample size*: 202 *Years of experience (mean ± SD)*: 10.2 ± 9.7 Overall *Age in years (mean ± SD)*: 40.4 ± 11.0 *Sex (N (% female))*: 284 (57%) *Sample size*: 497 *Years of experience (mean ± SD)*: 11.0 ± 10.7 **Included criteria:** NR **Excluded criteria:** NR **Pretreatment:** χ2 analyses revealed no differences between groups for any of the demographic and work characteristics: Gender, age education, marital state, and organisational seniority. t‐test analyses highlighted that training and control groups were almost overlapping at baseline on outcome measures. **Compliance rate:** NR **Response rate:** 98% **Type of healthcare worker:** various healthcare workers including doctors, nurses, and healthcare assistants	
Interventions	**Intervention characteristics** Mindfulness‐Based IARA Model® *Type of the intervention*: Intervention type 1 ‐ to focus one’s attention on the experience of stress *Description of the intervention*: In the first meeting, after a general IARA introduction and an introductory presentation from participants and trainers (all IARA trainers followed a specific qualifying course and were a psychologist and a neuroscientist, a psychologist and nurse, or psychologist and a director of nursing service depending on the training meeting; further information in Table 1), each HCP was invited to present him/herself and share some experiences belonging to daily work life in order to create a group climate. After this, the counselling principles were taught also using the role‐play techniques. In the second meeting both oval and star diagrams belonging to transpersonal psycho‐synthesis were explained. Role‐play was also used in this meeting session, improving HCP awareness by reflecting on three psychological concepts such as acceptance, listening, unconditional positive acceptance of oneself and others. Finally, during this meeting, a SWOT analysis (i.e. Strengths, Weaknesses, Opportunities and Threats analysis) was presented and explained. In particular, HCPs were invited to pay particular attention to the strength and opportunity elements included in the SWOT. The third meeting involved education on emotions. In particular, the session involved a deeper exploration of the recognition of primary emotions (astonishment, disgust, fear, anger, joy/happiness, sadness; shame was also considered) and techniques to particularly regulate anger and fear. Moreover, a basic mindfulness exercise was proposed. The attention to breathing and to the emerging thoughts as a first step to improve the awareness of mental activity and to stay in the present moment. During the final meeting, specific guided imagery IARA exercises were explained and demonstrated. Finally, HCPs shared their impressions of the training and some proposals for implementing the IARAin their wards. *The number of sessions*: 4 *Duration of each session on average*: 2 *Duration of the entire intervention*: NR *Duration of the entire intervention short vs long*: Short *Intervention deliverer*: all IARA trainers followed a specific qualifying course and were a psychologist and a neuroscientist, a psychologist and nurse, or psychologist and a director of nursing service depending on the training meeting. *Intervention form*: Group Control (no intervention) *Type of the intervention*: NA *Description of the intervention*: NA *The number of sessions*: NA *Duration of each session on average*: NA *Duration of the entire intervention*: NA *Duration of the entire intervention short vs long*: NA *Intervention deliverer*: NA *Intervention form*: NA	
Outcomes	*Zung Self‐Rating Anxiety Scale (SAS)* **Outcome type**: ContinuousOutcome *Perceived Stress Scale (PSS)* **Outcome type**: ContinuousOutcome	
Identification	**Sponsorship source:** this research received no external funding. **Country:** Italy **Setting:** public hospitals **Comments:** NR **Authors name:** Massimiliano Barattucci **Institution:** eCampus University, 22060 Novedrate, Italy **Email:** massimiliano.barattucci@uniecampus.it **Address:** 22060 Novedrate, Italy **Time period:** 2018‐2019	
Notes	Author M.Barattucci kindly provided clarification on table 5. PSS included in analysis 1.2	
***Risk of bias***		
**Bias**	**Authors' judgement**	**Support for judgement**
Random sequence generation (selection bias)	Unclear risk	Quote: "All HCPs belonging to these wards—doctors, nurses, and healthcare assistants—were randomly assigned to a control group (N = 301) or to an IARA training program (N = 301)." Sequence generation process insufficiently described
Allocation concealment (selection bias)	Unclear risk	Quote: "Material and Methods The research was conﬁgured as a randomized pre‐post evaluation with a comparison group, which included the completion of a questionnaire at the beginning (T0) and at the end of the training (T1). Baseline assessment was managed in November 2018, while Follow‐up in May 2019. All procedures performed in present study were in accordance with the 1964 Helsinki Declaration and its later amendments or comparable ethical standards. All participants gave their signed consent to participate in the study. The study was approved by the local ethics committee. From diﬀerent Italian public hospitals, 602 HCP volunteer participants were recruited. The research involved many wards, such as oncology, general medicine, neurology, general surgery, gastroenterology, orthopedics, traumatology, urology, otolaryngology, pulmonology, and home care professionals. All HCPs belonging to these wards—doctors, nurses, and healthcare assistants—were randomly assigned to a control group (N = 301) or to an IARA training program (N = 301). Overall,</b> 497 workers participated in ﬁlling" Insufficient information to understand whether intervention allocations could have been foreseen in advance of, during, enrolment.
Blinding of participants and personnel (performance bias) All outcomes	High risk	Participants were not blinded.
Blinding of outcome assessment (detection bias) All outcomes	High risk	Participants were not blinded whereas outcomes are self‐reported.
Incomplete outcome data (attrition bias) All outcomes	High risk	Quote: "and of 202 HCPs for the control group (response rate = 68%; Figure 1)." High lost to follow‐up in the control group, reasons not provided. Unclear whether this was a random.
Selective reporting (reporting bias)	Unclear risk	No trial registration or study protocol reported nor did we find one online.
Other bias	Unclear risk	Compliance not reported.

**Table:** Barbosa 2015

***Study characteristics***		
Methods	**‐Study design:** cluster‐randomised controlled trial **Study grouping:** parallel group	
Participants	**Baseline characteristics** Psychoeducational intervention *Age in years (mean ± SD)*: 43.4 ± 10.0 *Sex (N (% female))*: 27 (100%) *Sample size*: 27 *Years of experience (mean ± SD)*: 9.84 ± 4.9 Education‐only (active control) *Age in years (mean ± SD)*: 45.9 ± 8.0 *Sex (N (% female))*: 31 (100%) *Sample size*: 31 *Years of experience (mean ± SD)*: 9.4 ± 2.5 Overall *Age in years (mean ± SD)*: 44.72 ± 9.0 *Sex (N (% female))*: 58 (100%) *Sample size*: 58 *Years of experience (mean ± SD)*: 9.6 ± 3.7 **Included criteria:** to be included in the study, DCWs had to be employed for at least two months (so adjustments to the residents and facility had been achieved) and provide morning personal care (i.e. period of time between 7AM and 12AM that involved activities related to bathing, grooming, dressing and toileting) to people with a diagnosis of moderate to severe dementia. **Excluded criteria:** temporary DCWs and trainees were excluded as it was not possible to ensure their participation until the end of the study. **Pretreatment:** None of the measured socio‐demographic variables were statistically significantly different at baseline. At baseline, there were no significant differences between the groups in perceived stress, burnout, or job satisfaction. **Compliance rate:** NR **Response rate:** 100% **Type of healthcare worker:** Exclusively direct care workers (DCWs)	
Interventions	Psychoeducational intervention *Type of the intervention*: Intervention type 4 ‐ Combination of two or more of the above *Description of the intervention: the supportive component aimed to provide DCWs with coping strategies to manage work‐related stress and prevent burnout (e.g. time‐management, assertiveness, and problem‐solving). At the end of each supportive component, relaxation techniques, stretching, and strengthening exercises were practised. * *The number of sessions*: 8 *Duration of each session on average*: 90 min *Duration of the entire intervention*: 8 weeks *Duration of the entire intervention short vs long*: short *Intervention deliverer*: by a gerontologist and a physical therapist with training and experience in PCC approaches and psycho‐educational groups *Intervention form*: group Education‐only (active control) *Type of the intervention*: education‐only *Description of the intervention: the control facilities received an education‐only intervention.The coordination, length, order, and content of the sessions were the same as the educational component of the PE intervention. It was the absence of the supportive component that distinguished both interventions. Each participant was assisted during morning care by the same professionals who helped DCWs to deliver a more PCC and clarified doubts that emerged from sessions.* *The number of sessions*: 8 *Duration of each session on average*: 90 min *Duration of the entire intervention*: 8 weeks *Duration of the entire intervention short vs long*: short *Intervention deliverer*: by a gerontologist and a physical therapist with training and experience in PCC approaches and psycho‐educational groups *Intervention form*: Group	
Outcomes	*The Perceived Stress Scale (PSS)* **Outcome type**: ContinuousOutcome *Maslach Burnout Inventory ‐ Emotional exhaustion* **Outcome type**: ContinuousOutcome *Maslach Burnout Inventory ‐ Depersonalisation* **Outcome type**: ContinuousOutcome *Maslach Burnout Inventory ‐ Personal accomplishment* **Outcome type**: ContinuousOutcome	
Identification	**Sponsorship source:** This work was supported by grants (grant numbers SFRH/BD/72460/2010 and RIPD/CIF/109464/2009) from the Foundation for Science and Technology (FCT) **Country:** Portugal **Setting:** 4 aged‐care facilities **Comments:** NR **Authors name:** Ana Barbosa **Institution:** Department of Health Sciences, University of Aveiro, Campus Universita ´rio de Santiago, **Email:** anabarbosa@ua.pt **Address:** Agra do Crastoedifıcio 30, Aveiro, Portugal. **Time period:** NR	
Notes	PSS included in analysis 7.1 and 7.2	
***Risk of bias***		
**Bias**	**Authors' judgement**	**Support for judgement**
Random sequence generation (selection bias)	Low risk	All four facilities agreed to participate and were randomly allocated to the experimental group—PE intervention—or control group— education‐only intervention, using a random number generator.
Allocation concealment (selection bias)	Low risk	After recruitment, the facilities within each pair were randomly assigned to the experimental group–PE intervention—or control group— education‐only intervention—using a random number generator. This decision was supported by the fact that education has become the most widely used approach with DCWs. Randomization occurred at the facility level because of possible contamination. No indication of allocation concealment
Blinding of participants and personnel (performance bias) All outcomes	Low risk	Participants were blinded to the experimental or the control group.
Blinding of outcome assessment (detection bias) All outcomes	Low risk	Researchers were not blinded to the intervention or assessments, however outcomes were PROs.
Incomplete outcome data (attrition bias) All outcomes	Low risk	Lost to follow‐up not at random (due to sick leave, dismissal, vacation) but relatively small (13%)
Selective reporting (reporting bias)	Unclear risk	No trial registration or study protocol reported, nor did we find one online. No indication of selective reporting.
Other bias	Unclear risk	Unit of analysis error (i.e. when a study ignored the clustering of the data in their analysis). Compliance not reported.

**Table:** Behnammoghadam 2019

***Study characteristics***		
Methods	**Study design:** randomised controlled trial **Study grouping:** parallel group	
Participants	**Baseline characteristics** Eye movement desensitisation and reprocessing (EMDR) *Age in years (mean ± SD)*: 30.8 ± 5.5 *Sex (N (% female))*: NR *Sample size*: 25 *Years of experience (mean ± SD)*: 7.5 ± 4.9 Control (no intervention) *Age in years (mean ± SD)*: 31.5 ± 6.4 *Sex (N (% female))*: NR *Sample size*: 25 *Years of experience (mean ± SD)*: 8.6 ±5.6 Overall *Age in years (mean ± SD)*: 31.1 ± 5.9 *Sex (N (% female))*: NR *Sample size*: 50 *Years of experience (mean ± SD)*: 5.2 ± 8 **Included criteria:** employed as one of the technician classes (rescuer, basic, middle, or senior technician) at pre‐hospital medical emergency services, age range 18–55 years, working at pre‐hospital medical emergency services as their main job, not employed in administrative departments or communication centre of pre‐hospital medical emergency services, no drug addiction, no hearing or vision impairment, and concurrently, getting scores above 19 in the Alken stress scale, and provided written informed consent to participate in the study. **Excluded criteria:** no motivation to cooperate, intolerance to the treatment, absent for more than one therapeutic session, imprecise completion of data collection instruments, transfer or death of the technician. **Pretreatment:** there were no significant differences between the two groups for age, years of experience and stress at baseline. **Compliance rate:** not able to assess as participants absent for more than one therapeutic session were excluded **Response rate:** not able to assess **Type of healthcare worker:** exclusively emergency medical technicians	
Interventions	**Intervention characteristics** Eye movement densensitization and reprocessing (EMDR) *Type of the intervention*: Intervention type 1 ‐ to focus one’s attention on the experience of stress *Description of the intervention*: Subjects in the intervention group received EMDR training in five consecutive 45–90 sessions, as a research intervention based on the approved protocol, and according to the following eight stages: 1. History taking and treatment design: while communicating with the subject, the history was taken and the treatment process was designed. 2. Preparation: by describing the process, the subject was being prepared for EMDR implementation. 3. Evaluation: the subjects were asked to recall the problematic distressing events and measure their subjective units of disturbance using an 11‐degreemental disorder scale from 0 to 10. Zero means a lack of mental discomfort, and 10 means the maximum mental discomfort. Then, in a test to assess the validity of cognitions as one of the other pre‐test scales, they were asked to express their positive beliefs and rank it from 1 to 7 in a seven‐point scale. One means completely false and 7 means perfectly true. Then, after applying this technique, both of the scales were re‐evaluated. 4. Desensitisation: according to Shapiro, the inventor of this technique, at this stage the subject is asked to imagine the most prominent part of an annoying scene, focusing on the negative recognition of the scene that the subject previously described in a brief sentence expressing a harmful event such as “I am guilty“ or “it is really terrible or disturbing to me“to concentrate on emotions and physical states related to tension and anxiety, and then, after determining which part of the body is affected by the anxiety, the subject is instructed to follow the rapid movements of the therapist’s finger just about 30 cm away from his eyes, from right to left and vice versa, across his field of vision. This movement involves two rounds of trips to the sides within 1 second, which is considered a cycle, and each 24–24 cycles constitute a set. After each set, the researcher asks the subjects to stop imagining the scene, lean back into the back of their seat, and breathe deeply. Then, the level of mental discomfort and cognitive validity were ranked, evaluated, and recorded. This process, based on the need and motivation of the subjects, was continued until the level of mental discomfort reached 0 or minimum. 5. Implementation: the subjects were asked to recall the positive phrases and then repeat the eye movement process. 6. Physical scan: subjects were asked to focus on that part of the body that had difficulty during stress or anxiety, and evaluate the problem. 7. Completion: this step was to ensure the stability of the subjects at the end of the session. 8. Re‐evaluation: subjects again completed subjective units of disturbance and VOC scale. *The number of sessions*: 5 *Duration of each session on average*: NR *Duration of the entire intervention*: 5 days *Duration of the entire intervention short vs long*: Short *Intervention deliverer*: NR *Intervention form*: Individual Control (no intervention) *Type of the intervention*: NA *Description of the intervention*: NA *The number of sessions*: NA *Duration of each session on average*: NA *Duration of the entire intervention*: NA *Duration of the entire intervention short vs long*: NA *Intervention deliverer*: NA *Intervention form*: NA	
Outcomes	*Elkin stress symptoms scale* **Outcome type**: ContinuousOutcome	
Identification	**Sponsorship source:** NR **Country:** Iran **Setting:** Medical Emergency Services, urban and road stations **Comments:** NR **Authors name:** Sharif Shahini **Institution:** University of Medical Sciences, Next to Imam Sajad Hospital **Email:** sharif.shahini@yahoo.com **Address:** PO Box: 2591994 Yasuj, Iran **Time period:** 2017	
Notes	Elkin stress symptoms scale included in analysis 1.1	
***Risk of bias***		
**Bias**	**Authors' judgement**	**Support for judgement**
Random sequence generation (selection bias)	Low risk	Quote: "A number was assigned to the subjects who concurrently got a score of above 19 in the SSS, and by matching that number with the block random allocation list, the technicians were assigned to the intervention or control group, and the process was continued until the completion of the estimated sample size."
Allocation concealment (selection bias)	Unclear risk	Quote: "25 people for each group. During random block assignment, the order of the participants in the intervention and control groups was determined as follows. By multiplying the number of study groups (two groups) by 2, the number of samples per block was calculated as 4; then, by calculating the factorial of each block sample size (4!=4×3×2×1=24), the number of blocks generated from all possible orders was obtained as 24; since the number of people in each block was 4 and the estimated sample size was 50 based on the following description, by matching 13 random numbers generated by Sample Randomizer with the mentioned block numbers, the order of 50 research subjects was determined, numbers 1–50 were allocated to the subject and control groups, and the random allocation list was edited." Difficult to judge whether participants and/or investigators could possibly foresee assignment
Blinding of participants and personnel (performance bias) All outcomes	High risk	Participants were not blinded.
Blinding of outcome assessment (detection bias) All outcomes	High risk	Participants were not blinded whereas outcomes are self‐reported.
Incomplete outcome data (attrition bias) All outcomes	Low risk	No loss to follow‐up
Selective reporting (reporting bias)	Low risk	Quote: "Iranian Clinical Trial Registry Website (code: IRCT20180102038191N1)." Trial registration in which the stress scale is not specified. No indication of selective reporting.
Other bias	Unclear risk	Not able to assess compliance/as participants absent for more than one therapeutic session were excluded.

**Table:** Bernburg 2019

***Study characteristics***		
Methods	**Study design:** randomised controlled trial **Study grouping:** parallel group	
Participants	**Baseline characteristics** Mental Health Promotion Intervention *Age (mean ± SD)*: 31.3 ± 2.5 *Sex (N (% female))*: 36 (82%) *Sample size*: 44 *Years of experience (mean ± SD)*: 8.7 ± 2.1 Control (wait list) *Age (mean ± SD)*: 32.8 ± 2.1 *Sex (N (% female))*: 33 (79%) *Sample size*: 42 *Years of experience (mean ± SD)*: 9.4 ± 2.5 Overall *Age (mean ± SD)*: NR *Sex (N (% female))*: 69 (80%) *Sample size*: 86 *Years of experience (mean ± SD)*: NR **Included criteria:** formal inclusion criteria were: (1) employment as a full time working nurse in a psychiatric hospital department, (2) time to take part in the study over the whole time period, (3) written consent to finish the surveys (baseline and three follow‐ups) **Excluded criteria:** not being on sick‐leave (Due to sickness absence, six nurses were excluded) **Pretreatment:** we found no significant differences between intervention and WCG with regard to gender, age, and working experience. Not recorded whether groups differed at baseline on the primary outcome perceived stress. **Compliance rate:** NR **Response rate:** not able to assess **Type of healthcare worker:** exclusively nurses working in psychiatry	
Interventions	**Intervention characteristics** Mental Health Promotion Intervention *Type of the intervention*: Intervention type 4 ‐ Combination of two or more. *Description of the intervention*: The training modules were designed on basis and values of i.e. mindfulness and acceptance training, cognitive behavioural training and solution focused group work (Wise, Hersh, & Gibson, 2012). All training modules involved theoretical input, watching videos, oral group discussions, experimental exercises, and home assignments. The WCG received no training but answered all surveys included in the study. Content of the training modules ‐ Unit module on Introduction: opening, psycho‐educational information and discussion on the topic, working as a nurse. Unit Module on work‐related problems and strategies to solve problems in the working context of nurses in Psychiatry. Unit Module on relaxation techniques, emotion regulation techniques, cognitive strategies, acceptance and tolerance of emotions and effective self‐support. Unit Module on conflict management at work: conflict types and conflict handling in the hospital setting. Unit Module on planning for the future: Looking for supervision and feedback on one’s own job performance. Unit Module on communication for nurses: how to improve communication with patients, colleagues and supervisors in the hospital setting. Unit Module on organisational hospital culture: i.e. how to report mistakes to colleagues and supervisors and dealing with mistakes. Unit Module on social support: how to use social support during work, how to handle difficult work situations. Unit Overall training evaluation by the participating nurses *The number of sessions*: 12 *Duration of each session on average*: 1.5‐2 hours *Duration of the entire intervention*: 12 weeks *Duration of the entire intervention short vs long*: Long *Intervention deliverer*: Two certified instructors performed the training and were registered and accredited as psychotherapists. They had sufficient qualifications in cognitive behavioural therapy and systemic/solution focused brief therapy in group settings *Intervention form*: Group, face to face Control (wait list) *Type of the intervention*: NA *Description of the intervention*: The WCG received no training but answered all surveys included in the study. *The number of sessions*: NA *Duration of each session on average*: NA *Duration of the entire intervention*: NA *Duration of the entire intervention short vs long*: NA *Intervention deliverer*: NA *Intervention form*: NA	
Outcomes	*Perceived Stress Questionnaire* **Outcome type**: ContinuousOutcome	
Identification	**Sponsorship source:** NR **Country:** Germany **Setting:** Psychiatric Hospital **Comments:** NR **Authors name:** Monika Bernburg **Institution:** Institute of Occupational Medicine, Social Medicine and Environmental Medicine, Goethe‐University, Frankfurt am Main, Germany **Email:** mache@uke.de **Address:** CONTACT Stefanie Maches. Institute for Occupational and Maritime Medicine (ZfAM), University Medical Center Hamburg‐Eppendorf, Seewartenstrasse 10, 20459 Hamburg, Germany **Time period:** NR	
Notes	PSQ included in analysis 4.1 and 4.2	
***Risk of bias***		
**Bias**	**Authors' judgement**	**Support for judgement**
Random sequence generation (selection bias)	Low risk	Quote: "generated algorithm." Quote: "participate in this intervention study. Afterwards, these nurses were randomised into two study groups through a computer‐generated algorithm."
Allocation concealment (selection bias)	Unclear risk	Quote: "The researchers invited 140 nurses via email and/or direct communication to participate in the intervention. In sum, 86 nurses confirmed and gave their consent to participate in this intervention study. Afterwards, these nurses were randomized into two study groups through a computer‐generated algorithm." Insufficient information to understand whether intervention allocations could have been foreseen in advance of, during, enrolment.
Blinding of participants and personnel (performance bias) All outcomes	High risk	Quote: "So in the end, 44 nurses were included in the intervention group (IG) and 42 nurses took part in waitlist control group (CG)." Participants were not blinded.
Blinding of outcome assessment (detection bias) All outcomes	High risk	Participants were not blinded whereas outcomes are self‐reported.
Incomplete outcome data (attrition bias) All outcomes	Unclear risk	Attrition was not reported.
Selective reporting (reporting bias)	Unclear risk	No trial registration or study protocol reported, nor did we find one online
Other bias	Unclear risk	Compliance rate not reported and response rate not able to assess. Not recorded whether groups differed at baseline on the primary outcome perceived stress.

**Table:** Bernburg 2020

***Study characteristics***		
Methods	**Study design:** randomised controlled trial **Study grouping:** parallel group	
Participants	**Baseline characteristics** Psychosocial competence training combined with cognitive behavioural and solution‐focused counselling *Age (mean ± SD)*: 23.1 ± 2.5 *Sex (N (% female))*: 36 (78%) *Sample size*: 47 *Years of experience (mean ± SD)*: 1.3 ± 1.1 Control (no intervention) *Age (mean ± SD)*: 23.6 ± 2.4 *Sex (N (% female))*: 33 (71%) *Sample size*: 47 *Years of experience (mean ± SD)*: 1.2 ± 1.3 Overall *Age (mean ± SD)*: 23 ± 2.5 *Sex (N (% female))*: 69 (73%) *Sample size*: 94 *Years of experience (mean ± SD)*: 1.1 ± 1.3 **Included criteria:** Inclusion criteria were: (1) regular access to the Internet, (2) full‐time employment in a clinic, (3) a maximum of two years of work experience (junior nurse), (4) availability and willingness to participate during the 36 weeks, (5) agree to complete the questionnaires, (6) no previous knowledge or experience with mental health promotion training. **Excluded criteria:** NR **Pretreatment:** Baseline data on socio‐demographic differences indicated only small, insignificant differences between intervention and control group (P > 0.05). Not recorded whether groups differed at baseline on the primary outcome perceived stress. **Compliance rate:** NR **Response rate:** difficult to assess as figure 1 is mentioned but not included in the article. **Type of healthcare worker:** exclusively junior nurses	
Interventions	**Intervention characteristics** Psychosocial competence training combined with cognitive behavioural and solution‐focused counselling *Type of the intervention*: Intervention type 4 ‐ Combination of two or more of the above *Description of the intervention*: Training sessions included theoretical input, watching videos, oral group discussions, experiential exercises, and home assignments. Module: Psycho‐educational information. During the first session, the nurses received psycho‐educational information about stress based on Lazarus’ transactional model of stress and basic information on stress. This session was designed to prepare nurses with general knowledge of coping methods (emotion‐focused and problem‐focused coping strategies) and more specific coping strategies for work‐related problematic situations. Subsequently, the nurses identified personal goals, work‐related stressors, and motivation for training participation. Module on problem‐solving techniques. In sessions 2 and 3, the nurses worked on problem‐solving techniques. This module was based on solution‐focused therapy. The participants learnt a systematic six‐step problem‐solving method that can be applied to an individual’s problems. Typical scenarios for work‐related stress in hospitals were presented. Module on techniques of emotion regulation ‐ The nurses worked on modules for emotion control and emotion regulation. Techniques include muscle and breath relaxation, acceptance and tolerance of emotions, and effective self‐support. The techniques were presented using examples of emotional reactions related to the typical work context. Module for planning for the future ‐ The participants were asked to develop a plan for the future. They were also asked to strengthen something important in their lives and to imagine life after completion of the training modules. *The number of sessions*: 12 *Duration of each session on average*: 1.5 hours *Duration of the entire intervention*: 12 weeks *Duration of the entire intervention short vs long*: Long *Intervention deliverer*: Two qualified psychotherapists conducted the training sessions. Both psychotherapists were registered and accredited as psychotherapists. They had training in cognitive behavioural therapy, systemic therapy, and solution‐focused brief therapy *Intervention form*: Group, face‐to‐face Control (no intervention) *Type of the intervention*: NA *Description of the intervention*: The CG group received no intervention on mental health and did not undertake anything comparable to the intervention, e.g. any other training in psychosocial skills, counselling or therapy *The number of sessions*: NA *Duration of each session on average*: NA *Duration of the entire intervention*: NA *Duration of the entire intervention short vs long*: NA *Intervention deliverer*: NA *Intervention form*: NA	
Outcomes	*Perceived Stress Questionnaire (PSQ)* **Outcome type**: ContinuousOutcome *Maslach Burnout Inventory ‐ Emotional Exhaustion* **Outcome type**: ContinuousOutcome	
Identification	**Sponsorship source:** NR **Country:** Germany **Setting:** Hospitals **Comments:** NA **Authors name:** Monika Bernburg **Institution:** Institute of Occupational Medicine, Social Medicine and Environmental Medicine, Goethe University, Germany **Email:** s.mache@uke.de **Address:** Address for correspondence: Stefanie Mache, Institute for Occupational Medicine and Maritime Medicine (ZfAM), University Medical Center Hamburg‐Eppendorf, Martinistraße 52, 20251Hamburg, Germany. **Time period:** NR	
Notes	MBI‐EE included in analysis 4.1 and 4.2	
***Risk of bias***		
**Bias**	**Authors' judgement**	**Support for judgement**
Random sequence generation (selection bias)	Low risk	Quote: "After answering the baseline questionnaire (t0) participants were randomized with the ratio 50%: 50% to the two study groups (IG or CG). The randomization process was accomplished with a computer generated list of numbers."
Allocation concealment (selection bias)	Low risk	Quote: "This list was created by an independent research assistant; another assistant was blinded to the list, securing covered distribution to research conditions."
Blinding of participants and personnel (performance bias) All outcomes	High risk	Quote: "The CG group was asked to complete all surveys. The CG group received no intervention on mental health and did not undertake anything comparable to the intervention, e.g. any other training in psychosocial skills, counselling or therapy." Participants were not blinded.
Blinding of outcome assessment (detection bias) All outcomes	High risk	Participants were not blinded whereas outcomes are self‐reported.
Incomplete outcome data (attrition bias) All outcomes	Low risk	Quote: "Dropout attrition: There was an overall drop‐out rate (from randomization to analysis) of 12%. 10 participants decided to terminate the study (reasons included illness, non‐appearance of participants) and did not answer the questionnaires. Overall, four of the 94 participants at T1, 6/94 of participants at T2 and 4/94 of participants at T3 did not provide all follow‐up data on the results. The participants who did not provide all follow‐up data did not differ in any meaningful way, either in the primary outcome or in other baseline outcomes (P > 0.05), from those who provided data. " Low loss to follow‐up and follow‐up seems to be at random.
Selective reporting (reporting bias)	Unclear risk	No trial registration or no study protocol reported, nor did we find one online
Other bias	Unclear risk	Compliance rate not reported and response rate difficult to assess. Not recorded whether groups differed at baseline on the primary outcome perceived stress.

**Table:** Brazier 2022

***Study characteristics***		
Methods	**Study design:** randomised controlled trial **Study grouping:** parallel group	
Participants	**Baseline characteristics** Dear Doctor *Age (mean ± SD)*: NR *Sex (N (% female))*: NR *Sample size*: 74 *Years of experience (mean ± SD)*: NR Control (no intervention) *Age (mean ± SD)*: NR *Sex (N (% female))*: NR *Sample size*: 79 *Years of experience (mean ± SD)*: NR Overall *Age (mean ± SD)*: NR *Sex (N (% female))*: NR *Sample size*: 153 *Years of experience (mean ± SD)*: NR **Included criteria:** trainees were invited to participate if they were registered as being in Core Training Year 2 (CT2), or Speciality Training Years 3 or 4 (ST3 or ST4) of the UK training programme at the time of recruitment. **Excluded criteria:** no exclusion criteria were applied. **Pretreatment:** randomisation was well‐balanced in the final sample: participants in the two trial groups did not differ in terms of profile or covariates. **Compliance rate:** 1 of the 139 opted out (99%) **Response rate:** 18% **Type of healthcare worker:** exclusively trainee anaesthetists	
Interventions	**Intervention characteristics** Dear Doctor *Type of the intervention*: Intervention type 4 ‐ Combination of two or more *Description of the intervention*: The intervention consisted of 22 text messages, including an introduction message with details of how to opt out of receiving the messages, sent fortnightly for approximately 10 months. The programme was named‘Dear Doctor’ and the messages appeared to come from this name on recipients’ mobile phones. The intervention content was informed by factors associated with burnout in the existing literature (summarised in the online Supporting Information Appendix S1, Table S1) and by factors identified by UK trainee anaesthetists in a preceding interview study (online Supporting Information Appendix S1, Table S2). As contributors to burnout may vary across medical specialities, the interview study ensured that the intervention content was tailored to the target population. The intervention combined this tailored approach with evidenced practices from the behavioural science and well‐being literature. Messages drew on 11 evidence‐based themes, with some messages drawing on more than one theme. The 11 themes could be grouped into six higher level categories: gratitude; self‐efficacy; connection to purpose; social support; support resources (including mindfulness and self‐compassion); and planning prompts. *The number of sessions*: 22 text messages *Duration of each session on average*: NA *Duration of the entire intervention*: 10 months *Duration of the entire intervention short vs long*: Long *Intervention deliverer*: NA *Intervention form*: Individual text messages Control (no intervention) *Type of the intervention*: NA *Description of the intervention*: NA *The number of sessions*: NA *Duration of each session on average*: NA *Duration of the entire intervention*: NA *Duration of the entire intervention short vs long*: NA *Intervention deliverer*: NA *Intervention form*: NA	
Outcomes	*Copenhagen Burnout Inventory ‐ work‐related subscale* **Outcome type**: ContinuousOutcome	
Identification	**Sponsorship source:** The study was funded by the National Institute for Health Research Imperial Patient Safety Translational Research Centre and registered (ISRCTN11418903) **Country:** UK **Setting:** Royal College of Anaesthetists **Comments:** NR **Authors name:** A Brazier **Institution:** National Institute for Health Research Imperial Patient Safety Translational Research Centre, Faculty of Medicine, Imperial College London, London, UK **Email:** a.brazier19@imperial.ac.uk **Address:** NR **Time period:** 2019‐2020	
Notes	CBI included in analysis 4.1	
***Risk of bias***		
**Bias**	**Authors' judgement**	**Support for judgement**
Random sequence generation (selection bias)	Low risk	Quote: "Participants were randomly assigned to intervention or control conditions with a 1:1 allocation ratio using a random number generator (Stata â, StataCorp, College Station, TX, USA). Randomisation was stratiﬁed by training year and across ﬁve broad training regions. "
Allocation concealment (selection bias)	Unclear risk	Insufficient information to understand whether intervention allocations could have been foreseen in advance of, during, enrolment.
Blinding of participants and personnel (performance bias) All outcomes	High risk	Quote: "Participants were not blinded to their condition assignment: intervention group participants received the intervention; control participants did not." Participants were not blinded.
Blinding of outcome assessment (detection bias) All outcomes	High risk	Participants were not blinded whereas outcomes are self‐reported.
Incomplete outcome data (attrition bias) All outcomes	Unclear risk	Quote: "Despite the high attrition, there were no signiﬁcant differences in participant characteristics (v 2 s < 3.80, ps > 0.15) or baseline outcomes measures (ts < 0.81, ps > 0.42) between those who did and did not complete the ﬁnal survey (see also online Supporting Information Table S4)." High attrition, reasons other than did not complete survey not given. Lost to follow‐up appears to be at random.
Selective reporting (reporting bias)	Unclear risk	Quote: "The following exploratory ﬁndings should be regarded as indicative as they were not pre‐speciﬁed in the trial registry or protocol (the relevant outcomes were added after the start of the trial)." Trial registration number not provided, Difficult to assess whether there is selective outcome reporting.
Other bias	Unclear risk	Quote: "The RCoA identiﬁed and invited 1549 trainees to participate via email. Of the 647 (response rate 42%) who completed the baseline survey, 274 trainees (18% of the original cohort) consented to participate and remained in the trial throughout the trial period." Quote: "Female participants (v 2 = 8.07, p = 0.018), those with lower burnout (t = ‐2.42, p = 0.016), higher ‘meaningful’ score (t = 3.29, P = 0.001) and ‘valued’ score (t = 2.97, p = 0.003) were more likely to sign up for the trial after completing the baseline survey." Low selective response rate.

**Table:** Brennan 2006

***Study characteristics***		
Methods	RCT, USA	
Participants	Nurses with at least 6 months full‐time bedside nursing in a hospital setting. Those who regularly receive massage therapy on their own as well as anyone with medical reasons for not being able to have chair massage were excluded.	
Interventions	1) Experimental: massage: application to the back, neck, shoulders, arms and hands. Techniques used were effleurage, petrissage, friction, vibration and compression. One 30‐minute session per person over 4 days 2) Control: 10‐minute self‐directed break	
Outcomes	The Perceived Stress Scale	
Identification		
Notes	PSS included in analysis 2.1	
***Risk of bias***		
**Bias**	**Authors' judgement**	**Support for judgement**
Random sequence generation (selection bias)	Unclear risk	Not reported.
Allocation concealment (selection bias)	Low risk	"Sample size was 82 participants, randomly assigned to the massage group or the control group per a randomization schedule developed by a biostatistician who worked for the hospital but was not on the study team" (p. 337)
Blinding of participants and personnel (performance bias) All outcomes	High risk	Participants were not blinded.
Blinding of outcome assessment (detection bias) All outcomes	High risk	Participants were not blinded whereas outcomes are self‐reported.
Incomplete outcome data (attrition bias) All outcomes	High risk	"A total of 60 follow‐up surveys were completed, a 73% return rate" (p.339)
Selective reporting (reporting bias)	Low risk	There was only one outcome measured and reported.
Other bias	Unclear risk	We did not find any indications of other sources of bias.

**Table:** CezardaCosta 2019

***Study characteristics***		
Methods	**Study design:** randomised controlled trial **Study grouping:** parallel group	
Participants	**Baseline characteristics** Stretching exercise *Age (mean ± SD)*: 35.5 ± 9.5 *Sex (N (% female))*: NR *Sample size*: 20 *Years of experience (up to 3 years, 4‐7 years, ≥ 8 years)*: 20 (100%), 0 (0%), 0 (0%) Control (no intervention) *Age (mean ± SD)*: 37.8 ± 8.9 *Sex (N (% female))*: NR *Sample size*: 19 *Years of experience (up to 3 years, 4‐7 years, ≥ 8 years)*: 19 (100%), 0 (0%), 0 (0%) Overall *Age (mean ± SD)*: NR *Sex (N (% female))*: NR *Sample size*: 39 *Years of experience (up to 3 years, 4‐7 years, ≥ 8 years)*: 39 (100%), 0 (0%), 0 (0%) **Included criteria:** to participate in the research, NPs could not present any medical impediment to performing physical exercises and not participating in any kind of physical activity oriented during the research. Those individuals who were absent from classes for three consecutive sessions for any reason were excluded. **Excluded criteria:** NR **Pretreatment:** NR **Compliance rate:** the frequency of the students was recorded in all classes. The participant who missed three or more consecutive classes was excluded from the investigation; however, the only person excluded from the study was even allowed participating in the classes **Response rate:** NR **Type of healthcare worker:** exclusively nurses	
Interventions	**Intervention characteristics** Stretching exercise *Type of the intervention*: Intervention type 2 ‐ to focus one’s attention away from the experience of stress *Description of the intervention*: The members of the EG participated in MS classes for eight weeks, with 40‐minute sessions containing active and static stretching exercises under the supervision and guidance of a Physical Education teacher. The classes were offered for three days a week and each member attended at least two days a week. The frequency of the students was recorded in all classes. The participant who missed three or more consecutive classes was excluded from the investigation; however, the only person excluded from the study was actually allowed to participate in the classes. In each session, eight exercises with four sets of 30 seconds and 30 seconds intervals were given, as recommended by the American College of Sports Medicine (2013). Active and static stretching exercises were directed to the body segments in general. The body segment was slowly moved up to a certain range of motion with slight tension (muscle discomfort), remaining in the position *The number of sessions*: 16‐24 *Duration of each session on average*: 40 min *Duration of the entire intervention*: 8 weeks *Duration of the entire intervention short vs long*: Short *Intervention deliverer*: a Physical Education teacher *Intervention form*: Group, face‐to‐face Control (no intervention) *Type of the intervention*: NA *Description of the intervention*: NA *The number of sessions*: NA *Duration of each session on average*: NA *Duration of the entire intervention*: NA *Duration of the entire intervention short vs long*: NA *Intervention deliverer*: NA *Intervention form*: NA	
Outcomes	*Occupational Stress Scale (OSS)* **Outcome type**: ContinuousOutcome	
Identification	**Sponsorship source:** Fundação Carlos Chagas Filho de Amparo à Pesquisa do Estado do Rio de Janeiro (FAPERJ **Country:** Brasil **Setting:** The State Institute of the Brain Paulo Niemeyer (IEC). **Comments:** NR **Authors name:** Flávia Porto **Institution:** Instituto de Educação Física e Desportos, Universidade do Estado do Rio de Janeiro **Email:** laviaporto30@gmail.com **Address:** Rua São Francisco Xavier, 524, Sala 9122F, Maracanã, CEP 20550‐900, Rio de Janeiro, RJ, Brasil **Time period:** NR	
Notes	OSS included in analysis 2.1	
***Risk of bias***		
**Bias**	**Authors' judgement**	**Support for judgement**
Random sequence generation (selection bias)	Low risk	Quote: "They were chosen from a list of random numbers generated in software (Microsoft Excel 2010, São Paulo, Brazil)."
Allocation concealment (selection bias)	Unclear risk	Quote: "The volunteers were randomly assigned to the experimental group (EG: n = 20, 35.5 ± 9.5 years old, 69.9 ± 13.7 kg and 1.62±0.5m) and the control group (CG: n = 19, 37.8±8.9 years old, 81.8±15.4 kg and 1.68±0.9m). They were chosen from a list of random numbers generated in software (Microsoft Excel 2010, São Paulo, Brazil). The CG was submitted to the same evaluation as the EG. However, they did not participate in the classes of muscle stretching (MS). There was no blinding of participants and evaluators; however, it was considered that it did not influence the outcome of the study." Difficult to judge whether participants and/or investigators could possibly foresee assignment
Blinding of participants and personnel (performance bias) All outcomes	High risk	Quote: "There was no blinding of participants and evaluators; however, it was considered that it did not influence the outcome of the study." Participants were not blinded.
Blinding of outcome assessment (detection bias) All outcomes	High risk	Quote: "(MS). There was no blinding of participants and evaluators; however, it was considered that it did not influence the outcome of the study." Participants were not blinded whereas outcomes are self‐reported.
Incomplete outcome data (attrition bias) All outcomes	Low risk	Quote: "The participant who missed three or more consecutive classes was excluded from the investigation; however, the only person excluded from the study was even allowed participating in the classes."
Selective reporting (reporting bias)	Low risk	Trial registration. No indication of selective reporting.
Other bias	Unclear risk	Response rate not reported.

**Table:** Chen 2015

***Study characteristics***		
Methods	**Study design:** randomised controlled trial **Study grouping:** parallel group	
Participants	**Baseline characteristics** Aromatherapy *Age (mean ± SD)*: 33.1 ± 6.8 *Sex (N (% female))*: 53 (100%) *Sample size*: 53 *Years of experience (mean ± SD)*: 8.0 ± 6.0 Control (no intervention) *Age (mean ± SD)*: 33.3 ± 6.5 *Sex (N (% female))*: 57 (100%) *Sample size*: 57 *Years of experience (mean ± SD)*: 8.4 ± 6.1 Overall *Age (mean ± SD)*: 33.2 (NR) *Sex (N (% female))*: 110 (100%) *Sample size*: 110 *Years of experience (mean ± SD)*: 8.2 (NR) **Included criteria:** female nursing staff, participants were chosen by applying the following criteria: displaying more symptoms of stress than the average of 4.6, scheduled to work over seven consecutive days, ages and working years. The criteria possessed by the experimental group included: able to communicate with researchers, willing to participate in the project, not allergic to lavender, not suffering from any form of liver or kidney dysfunction, and with normal olfactory functions. **Excluded criteria:** NR **Pretreatment:** comparisons of control variables (ages, work experiences, working years, levels of education and promotion potentials) between the experimental and the control group showed no significant statistical distribution variance. Differences in the number of the stress symptoms on the pre‐test day between the experimental group and the control group did not have a statistical significance. **Compliance rate:** NR **Response rate:** not able to assess. Purposive sampling. **Type of healthcare worker:** exclusively female nurses.	
Interventions	**Intervention characteristics** Aromatherapy *Type of the intervention*: Intervention type 2 ‐ to focus one’s attention away from the experience of stress *Description of the intervention*: nurses in the experimental group wore bottles of 3% lavender oil hung in front of their right chests. On the first day of the study, the nurses in the experimental group began to wear the lavender oil bottle at the start of their shifts, and wore the necklaces at all times while working for their next four working days. Additionally, the nurses were required to rate their job stress‐related symptoms before the end of their shift every day for one pre‐test day and four posttest days. *The number of sessions*: NA *Duration of each session on average*: NA *Duration of the entire intervention*: 4 days *Duration of the entire intervention short vs long*: Short *Intervention deliverer*: NA *Intervention form*: Individual Control (no intervention) *Type of the intervention*: Placebo *Description of the intervention*: But the nurses in the control group wore bottles without lavender oil. Concurrently, the nurses in the control group were still required to write down their job stress‐related symptoms before the end of their shifts on the pretest day and each of the four posttest days. *The number of sessions*: NA *Duration of each session on average*: NA *Duration of the entire intervention*: NA *Duration of the entire intervention short vs long*: NA *Intervention deliverer*: NA *Intervention form*: NA	
Outcomes	*Number of Job stress‐related symptoms* **Outcome type**: ContinuousOutcome	
Identification	**Sponsorship source:** NR **Country:** Taiwan **Setting:** Teaching hospital **Comments:** NR **Authors name:** Li Fang **Institution:** Department of Nursing, Meiho University, **Email:** fangli72@yahoo.edu.tw **Address:** 23 Pingguang Road, Neipu Shiang, Pingtung County 912, Taiwan. **Time period:** NR	
Notes		
***Risk of bias***		
**Bias**	**Authors' judgement**	**Support for judgement**
Random sequence generation (selection bias)	Unclear risk	Quote: "Subsequently, the 110 nurses were randomly separated into two groups, one experimental group of 53 and one control group of 57."
Allocation concealment (selection bias)	Unclear risk	Difficult to judge whether participants and/or investigators could possibly foresee assignment, but the outcomes are not likely to be influenced.
Blinding of participants and personnel (performance bias) All outcomes	Low risk	Quote: "Participants of the control group pinned small empty bottles on their clothes on the right chest. Some participants of the control group might note that the small bottle would not decrease stress because it had no odour. When participants in the experimental group received small bottles containing lavender oil and experienced the smells, they might know there was something in the bottle that might make differences." Placebo control group.
Blinding of outcome assessment (detection bias) All outcomes	Low risk	Participants were blinded and outcomes are PROs.
Incomplete outcome data (attrition bias) All outcomes	Unclear risk	Not mentioned explicitly, but it appears that there is no loss to follow‐up.
Selective reporting (reporting bias)	Unclear risk	No trial registration or study protocol reported, nor did we find one online No indication of selective reporting.
Other bias	Unclear risk	Compliance rate not reported. Response rate not able to assess.‘Loosely’ validated outcome measure.

**Table:** Cheng 2015

***Study characteristics***		
Methods	**Study design:** randomised controlled trial **Study grouping:** parallel group	
Participants	**Baseline characteristics** Gratitude *Age (N (% 21‐25, 26‐30, 31‐35, ≥ 36))*: 9 (27%), 12 (35%), 9 (27%), 4 (12%) *Sex (N (% female))*: 16 (47%) *Sample size*: 34 *Years of experience (mean ± SD)*: 8.1 ± 4.8 Hassle *Age (N (% 21‐25, 26‐30, 31‐35, ≥ 36))*: 11 (32%), 14 (41%), 8 (24%), 1 (3%) *Sex (N (% female))*: 20 (59%) *Sample size*: 34 *Years of experience (mean ± SD)*: 5.7 ± 3.9 Control (no intervention) *Age (N (% 21‐25, 26‐30, 31‐35, ≥ 36))*: 1 (3%), 14 (41%), 12 (35%), 7 (21%) *Sex (N (% female))*: 20 (59%) *Sample size*: 34 *Years of experience (mean ± SD)*: 4.4 ± 3.7 Overall *Age (N (% 21‐25, 26‐30, 31‐35, ≥ 36))*: 21 (21%), 40 (39%), 29 (28%), 12 (12%) *Sex (N (% female))*: 56 (55%) *Sample size*: 102 *Years of experience (mean ± SD)*: NR **Included criteria:** full‐time Chinese professional workers **Excluded criteria:** exclusion criterion was scheduled long leave in the next 4 months. **Pretreatment:** age, education and years of experience differed statistically significant at baseline. **Compliance rate:** the compliance rate was excellent, with 99% of the diary days having valid returns. There were no significant differences between the two groups on number of diaries completed, as well as total number of events reported in the 4‐week period. **Response rate:** 82% **Type of healthcare worker:** various health care professionals including physicians (33%), nurses (55%) and physical/occupational therapists (12%).	
Interventions	**Intervention characteristics** Gratitude *Type of the intervention*: 1. Intervention type 1 ‐ to focus one’s attention on the experience of stress *Description of the intervention*: In both the gratitude and the hassle condition, participants wrote diaries about work‐related events twice a week for four consecutive weeks. The gratitude condition received this instruction: Different things happen at work every day. Some are minor, some are important. Whether minor or important, sometimes you feel thankful that these events have happened to you. For example, you may feel thankful that your colleague swapped work schedules with you, or helped you in some way that made your job easier In both conditions, participants were instructed to write at least one such event on the diary in either Chinese or English, whichever language was more comfortable to them. Events were sequentially numbered by the participant who provided them, and each event was typically described briefly, as in a summary rather than lengthy narratives *The number of sessions*: 8 *Duration of each session on average*: NA *Duration of the entire intervention*: 4 weeks *Duration of the entire intervention short vs long*: Short *Intervention deliverer*: NA *Intervention form*: Individual Hassle *Type of the intervention*: 1. Intervention type 1 ‐ to focus one’s attention on the experience of stress *Description of the intervention*: In both the gratitude and the hassle condition, participants wrote diaries about work‐related events twice a week for four consecutive weeks. Instructions for the hassle condition were as follows: Different things happen at work every day. Some are minor, some are important. Whether minor or important, sometimes you feel annoyed or even angry that these things actually happened to you. For example, you might feel annoyed because a patient’s relative made a complaint about you, or because the work was exhausting. In both conditions, participants were instructed to write at least one such event on the diary in either Chinese or English, whichever language was more comfortable to them. Events were sequentially numbered by the participant who provided them, and each event was typically described briefly, as in a summary rather than lengthy narratives *The number of sessions*: 8 *Duration of each session on average*: NA *Duration of the entire intervention*: 4 weeks *Duration of the entire intervention short vs long*: Short *Intervention deliverer*: NA *Intervention form*: Individual Control (no intervention) *Type of the intervention*: NA *Description of the intervention*: Control participants were not asked to do anything. *The number of sessions*: NA *Duration of each session on average*: NA *Duration of the entire intervention*: NA *Duration of the entire intervention short vs long*: NA *Intervention deliverer*: NA *Intervention form*: NA	
Outcomes	*Perceived Stress Scale* **Outcome type**: ContinuousOutcome *Center for Epidemiologic Studies‐Depression Scale (CES‐D)* **Outcome type**: ContinuousOutcome	
Identification	**Sponsorship source:** This study was supported by Research Grants Council of Hong Kong Strategic Public Policy Research Grant No. HKIEd1001‐SPPR‐08 awarded to Sheung‐Tak Cheng. **Country:** Hong Kong **Setting:** 5 public hospitals **Comments:** NR **Authors name:** Sheung‐Tak Cheng **Institution:** Department of Health and Physical Education, Hong Kong **Email:** takcheng@ied.edu.hk **Address:** Institute of Education, 10 Lo Ping Road, Tai Po, N.T. **Time period:** NR	
Notes	PSS included in analysis 1.1	
***Risk of bias***		
**Bias**	**Authors' judgement**	**Support for judgement**
Random sequence generation (selection bias)	Low risk	Quote: "control. Method Design and Randomization This was a double‐blind randomized controlled trial with follow‐up to 3 months posttreatment. Participants were randomly assigned into one of three experimental conditions— gratitude, hassle, and nil‐treatment control. 3 Block‐restricted randomization was performed by the second author using a true random number generator to create groups of" Quote: "generator to create groups of equal size (n = 34 per group). Participants were told that this was a study about the well‐being of health care workers, without further details about the research objective or hypothesis. Data collection consisted purely of self‐ administered questionnaires. The research assistants were blind to experimental assignment and were not involved in obtaining diaries or answers to the questionnaires. Participants were debriefed at the conclusion of the study."
Allocation concealment (selection bias)	Low risk	No indication of selection bias
Blinding of participants and personnel (performance bias) All outcomes	Unclear risk	Quote: "All questionnaires and diaries were filled out by the participants themselves, who were told not to disclose details of the experiment to others." Although this was a double‐blind randomised‐controlled trial it is questionable whether the blinding was effective as participants randomised to the gratitude or hassle group were asked to keep a diary and could have informed colleagues.
Blinding of outcome assessment (detection bias) All outcomes	Unclear risk	Participants were blinded to group assignment and outcomes are PROs. It is questionable whether the blinding was effective as participants randomised to the gratitude or hassle group were asked to keep a diary and could have informed colleagues.
Incomplete outcome data (attrition bias) All outcomes	Low risk	Quote: "One hassle and two gratitude participants did not have data at the 3‐month follow‐up (see Figure 1). Little’s (1988) missing completely at random test yielded a nonsignificant result, 2 (6) 7.14, p.308. Therefore, the data were missing completely at random, and all available data could be used.
Selective reporting (reporting bias)	Low risk	Quote: "The trial protocol is available from the first author." No public trial protocol nor trial registration. No identification of selective outcome reporting.
Other bias	Low risk	Quote: "Although age, years of experience, and education were significantly different among groups at baseline, and were related to perceived stress, only the latter two were included as covariates, as age and years of experience were highly correlated (r .89, p.001) and experience was theoretically more closely related to stress." Quote: "total of 125 practitioners were approached, and 102 physicians, nurses, physiotherapists, and occupational therapists provided consent to participate (success rate 82%)." Quote: "Owing partly to reminders sent by the research team, the compliance rate was excellent, with 99% of the diary days having valid returns. There were no significant differences between the two groups on number of diaries completed"

**Table:** Chesak 2020

***Study characteristics***		
Methods	**Study design:** randomised controlled trial **Study grouping:** parallel group	
Participants	**Baseline characteristics** Authentic Connections Groups *Age (mean ± SD)*: NR *Sex (N (% female))*: NR *Sample size*: 18 *Years of experience (mean ± SD)*: NR Control (no intervention) *Age (mean ± SD)*: NR *Sex (N (% female))*: NR *Sample size*: 18 *Years of experience (mean ± SD)*: NR Overall *Age (mean ± SD)*: NR *Sex (N (% female))*: NR *Sample size*: 36 *Years of experience (mean ± SD)*: NR **Included criteria:** inclusion criteria included being: (a) a nursing education specialist or clinical nurse specialist, and (b) a mother to at least one child or adult child. **Excluded criteria:** exclusion criteria included: (a) being actively suicidal or (b) meeting criteria for psychoses. **Pretreatment:** NR **Compliance rate:** 1/18 allocated to the intervention group dropped out. Session attendance rates among the intervention group averaged 92% across the study (not including the participant who dropped out) **Response rate:** NR **Type of healthcare worker:** exclusively nurses	
Interventions	**Intervention characteristics** Authentic Connections Groups *Type of the intervention*: Intervention type 1 ‐ to focus one’s attention on the experience of stress *Description of the intervention*: The intervention sessions included facilitated discussions cantered on acknowledging and addressing the many stressors that professional mothers who are raising children face. The facilitators intentionally create a warm, accepting, and empathic environment that allowed participants to feel seen and heard for who they are at their core. The sessions were participatory in nature and de‐signed to be insight‐oriented in an effort to unobtrusively and respectfully guide participants towards discovering optimal solutions to shared work, parenting, and personal life issues. Examples of discussion topics include minimising rumination, assertiveness and mentorship at work, feelings of shame and self‐doubt, limit‐setting with children, and dealing with their pain. Participants were guided in methods to both tend to themselves and develop a support system, or “go‐to committee,” to pro‐vide them with ongoing support both during and after the sessions concluded *The number of sessions*: 12 *Duration of each session on average*: 1 hour *Duration of the entire intervention*: 12 weeks *Duration of the entire intervention short vs long*: Long *Intervention deliverer*: Researchers both of whom attended mentored training to prepare them to lead the intervention. The interventionists themselves are not just highly skilled professionals but also mothers themselves. Thus, each one of them resonated strongly, at a personal as well as professional level, with the issues to be addressed in the group sessions. *Intervention form*: Group of six participants Control (no intervention) *Type of the intervention*: no intervention *Description of the intervention*: participants in the control group were provided 1 hour per week of protected time reserved on their online work calendars for 12 weeks *The number of sessions*: 12 *Duration of each session on average*: 1 hour *Duration of the entire intervention*: 12 weeks *Duration of the entire intervention short vs long*: NR *Intervention deliverer*: NR *Intervention form*: NR	
Outcomes	*The Perceived Stress Scale* **Outcome type**: ContinuousOutcome *Maslach Burnout Inventory ‐ Emotional Exhaustion* **Outcome type**: ContinuousOutcome *Maslach Burnout Inventory ‐ Personal accomplishment (lack of)* **Outcome type**: ContinuousOutcome *Self‐Rating Depression Scale ‐ Depression* **Outcome type**: ContinuousOutcome *Self‐Rating Depression Scale ‐ Anxiety* **Outcome type**: ContinuousOutcome	
Identification	**Sponsorship source:** Funding Sources: Elizabeth C. Bonner Endowment Fund; Authentic Connections **Country:** USA **Setting:** Hospital **Comments:** NA **Authors name:** Sherry S. Chesak **Institution:** Department of Nursing, Division of Nursing Research, Mayo Clinic **Email:** chesak.sherry@mayo.edu **Address:** 200 First St. SW, Rochester, MN 55905 **Time period:** NR	
Notes	PSS included in analysis 1.1 and 1.2 Self‐Rating Depression Scale ‐ Depression included in analysis 1.4 and 1.5	
***Risk of bias***		
**Bias**	**Authors' judgement**	**Support for judgement**
Random sequence generation (selection bias)	Unclear risk	Quote: "Participants who agreed and consented were randomly assigned to the intervention group (n = 18) or control group (n = 18)." Sequence generation process not mentioned
Allocation concealment (selection bias)	Unclear risk	Quote: "Participants who agreed and consented were randomly assigned to the intervention group (n = 18) or control group (n = 18). " Unable to judge whether participants and/or investigators could possibly foresee assignment
Blinding of participants and personnel (performance bias) All outcomes	High risk	Participants were not blinded
Blinding of outcome assessment (detection bias) All outcomes	High risk	Participants were not blinded whereas outcomes are self‐reported.
Incomplete outcome data (attrition bias) All outcomes	High risk	13/15 of the 18 participants (72%/83%) allocated to the intervention group completed respectively post‐intervention and 3‐month follow‐up assessment. 17/14 of the 18 participants (78%/94%) allocated to the intervention group completed respectively post‐intervention and 3‐month follow‐up assessment. Reasons not provided. Not mentioned whether lost to follow‐up at random.
Selective reporting (reporting bias)	Unclear risk	No trial registration or study protocol reported, nor did we find one online
Other bias	Unclear risk	Not able to assess response rate. Authors mention that they have corrected for baseline differences. However, the baseline characteristics have not been reported.

**Table:** Cho 2021

***Study characteristics***		
Methods	**Study design:** randomised controlled trial **Study grouping:** parallel group	
Participants	**Baseline characteristics** Meridian acupressure *Age (mean ± SD)*: 26.2 ± 2.4 *Sex (N (% female))*: 26 (90%) *Sample size*: 29 *Years of experience (< 1, 1 – < 3, 3– < 5, ≥ 5)*: 9 (31%), 5 (17%), 7 (24%), 8 (28%) Control (wait list) *Age (mean ± SD)*: 26.8 ± 2.7 *Sex (N (% female))*: 29 (97%) *Sample size*: 30 *Years of experience (< 1, 1 – < 3, 3 – < 5, ≥ 5)*: 3 (10%), 5 (17%), 11 (37%), 11 (37%) Overall *Age (mean ± SD)*: 26.5 ± 2.5 *Sex (N (% female))*: 55 (93%) *Sample size* : 59 *Years of experience (< 1, 1 – < 3, 3 – < 5, ≥ 5)*: NR **Included criteria:** the inclusion criteria for participants were daytime shift work nurses who voluntarily agreed to participate, without cognitive disorder, with clear consciousness, ability to communicate in verbal and non‐verbal language, and ability to understand the objectives of the study. Three participants who reported a poor state of health state without a doctor’s diagnosis and prescription were included. **Excluded criteria:** the exclusion criteria were persons diagnosed with acute or chronic illness by a doctor, those who have taken a prescription with skin lesions at the intervention site, and pregnant and lactating women. **Pretreatment:** the analysis of the homogeneity between the intervention and control groups showed that they were homogeneous, with a significance level of P < 0.05 **Compliance rate:** NR **Response rate:** NR convenience sample **Type of healthcare worker:** exclusively nurses	
Interventions	**Intervention characteristics** Meridian acupressure *Type of the intervention*: Intervention type 2 ‐ to focus one’s attention away from the experience of stress *Description of the intervention*: Firstly, the researcher trimmed the nails and washed the hands immediately before the intervention in order to prevent skin irritation. The intervention was performed at the nurse station in order to maintain the privacy of the participants. A smartphone with a stopwatch function was prepared for 10‐s finger‐pressure per Meridian acupressure point and 5‐s pause. The participants were instructed to sit comfortably on a stationary chair and were informed in advance that acupressure was to be applied on 6 Meridian points for approximately 15 min. The researcher delivering the intervention had completed a special training course as an acupressure therapist in an acupressure research institute in SouthKorea. This researcher has been educating and serving the community for many years. The participants were encouraged to express any discomfort during the Meridian acupressure at any time, and relaxation of mind and body was induced. The participants were allowed to have a rest for 15 min while relaxing comfortably after the intervention. *The number of sessions*: 3 *Duration of each session on average*: 15 min *Duration of the entire intervention*: 3 days *Duration of the entire intervention short vs long*: Short *Intervention deliverer*: Trained researcher *Intervention form*: Individual, face‐to‐face Control (wait list) *Type of the intervention*: NA *Description of the intervention*: NA *The number of sessions*: NA *Duration of each session on average*: NA *Duration of the entire intervention*: NA *Duration of the entire intervention short vs long*: NA *Intervention deliverer*: NA *Intervention form*: NA	
Outcomes	*The stress scale ‐ psychological stress* **Outcome type**: ContinuousOutcome *The stress scale ‐ physical stress* **Outcome type**: ContinuousOutcome *The State Anxiety Inventory (SAI)* **Outcome type**: ContinuousOutcome *The stress scale ‐ total* **Outcome type**: ContinuousOutcome	
Identification	**Sponsorship source:** The author(s) received no financial support for the research, authorship, and/or publication of this article **Country:** Korea **Setting:** Hospital **Comments:** NR **Authors name:** Youngmi Cho **Institution:** Department of Nursing, Sun Moon University **Email:** choyoung23@yahoo.com **Address:** Chungcheongnam‐do, Asan‐si 31460 Korea **Time period:** 2018	
Notes	The stress scale included in analysis 2.1 STAI included in analysis 2.3	
***Risk of bias***		
**Bias**	**Authors' judgement**	**Support for judgement**
Random sequence generation (selection bias)	Low risk	Quote: "The participants were recruited through convenience sampling. During the coin toss, heads meant the subject became a participant in the intervention group in this study."
Allocation concealment (selection bias)	Unclear risk	Quote: "The participants were recruited through convenience sampling. During the coin toss, heads meant the subject became a participant in the intervention group in this study. " Insufficient information to understand whether intervention allocations could have been foreseen in advance of, during, enrolment.
Blinding of participants and personnel (performance bias) All outcomes	High risk	Participants were not blinded
Blinding of outcome assessment (detection bias) All outcomes	High risk	Participants were not blinded whereas outcomes are self‐reported.
Incomplete outcome data (attrition bias) All outcomes	Low risk	No loss to follow‐up
Selective reporting (reporting bias)	Unclear risk	No trial registration or no study protocol reported, nor did we find one.
Other bias	Unclear risk	Compliance and response rate not recorded.

**Table:** Cohen‐Katz 2005

***Study characteristics***		
Methods	RCT, USA	
Participants	25 nurses, pastoral care, respiratory therapy and social work personnel	
Interventions	1) Experimental: mindfulness‐based stress reduction programme: 8‐week program with approximately 2.5 hours teaching per week and homework practice with audiotapes for six days a week. Group sessions included teaching on topics such as communication skills, stress reactivity and self‐compassion and experiential exercises to help participants integrate these concepts. 2) Control: no intervention	
Outcomes	MBI, Brief Symptom Inventory	
Identification	We kindly received data from the author.	
Notes	MBI‐EE included in analysis 2.1	
***Risk of bias***		
**Bias**	**Authors' judgement**	**Support for judgement**
Random sequence generation (selection bias)	Unclear risk	"Participants were then randomly assigned to the treatment group or the wait‐list control group." (p.27)
Allocation concealment (selection bias)	Unclear risk	Not reported.
Blinding of participants and personnel (performance bias) All outcomes	High risk	Participants were not blinded.
Blinding of outcome assessment (detection bias) All outcomes	High risk	Participants were not blinded whereas outcomes are self‐reported.
Incomplete outcome data (attrition bias) All outcomes	Low risk	2/14 (14%) in the treatment group did not return completed inventories and were not taken into consideration in the analyses, which is below our pre‐defined cut‐off value.
Selective reporting (reporting bias)	Low risk	All outcomes reported.
Other bias	Unclear risk	We did not find any indications of other sources of bias.

**Table:** Concilio 2021

***Study characteristics***		
Methods	**Study design:** randomised controlled trial **Study grouping:** parallel group	
Participants	**Baseline characteristics** Text messaging (Social Support Behavioral Code) *Age (range in years)*: NR *Sex (N (% female))*: NR *Sample size*: 11 *Years of experience (mean ± SD)*: NA Control (text messaging ‐ medical facts) *Age (range in years)*: NR *Sex (N (% female))*: NR *Sample size*: 11 *Years of experience (mean ± SD)*: NA Overall *Age (range in years)*: 21 to 30 *Sex (N (% female))*: 20 (95%) *Sample size*: 22 *Years of experience (mean ± SD)*: NA **Included criteria:** eligibility criteria were: (a) NLGNs employed as RNs for the first time, (b) ages 19 to 37 years (millennials and post‐millennials) as the majority joining the workforce (NursingLicensure.org, 2020), (c) proficient in English, (d) working in an acute care facility as an RN during the first year of hire, (e) had a working personal smartphone, (f) had the ability to send and receive text messages, (g) had an active and working personal email account, (h) were willing to participate for 6 weeks, (i) completed survey instruments at baseline, week 3, and week 6, (j) agreed to not use or carry their smartphone while performing direct patient care, and (k) assumed any data charges for text messages **Excluded criteria:** exclusion criteria were NLGNs who had worked as an RN on another floor or at another organisation **Pretreatment:** NR **Compliance rate:** **Response rate:** 100% **Type of healthcare worker:** exclusively nurses who were in their first year of hire.	
Interventions	** Intervention characteristics** Text messaging (Social Support Behavioral Code) *Type of the intervention*: 3. Intervention type 3 ‐ to focus on work‐related risk factors on an individual level. *Description of the intervention*: Week 1: Emotional support March 23: Monday: quote: “Speak with someone this week who understands or knows you well.”Week 3: Esteem support April 6: Monday: quote: “Always do your best so you can be proud that you gave it your best shot." Week 5: Network support April 20: Monday: quote: “Connect with others who share your specialty and with other newly licenced nurses: https://nurse.org/orgs.shtml to find your nursing organization to find your nursing organization". *The number of sessions*: 24 unique text messages *Duration of each session on average*: NA *Duration of the entire intervention*: 6 weeks *Duration of the entire intervention short vs long*: Short *Intervention deliverer* : NR *Intervention form*: Text messages Control (text messaging ‐ medical facts) *Type of the intervention*: Control *Description of the intervention*: Week 1 Monday: “DVT usually affects the deep veins of the legs” (MedlinePlus.gov, 2017) Week 3Monday: “An ostomy is surgery to create an opening (stoma) from an area inside the body to the outside. It treats certain diseases of the digestive or urinary systems. It can be permanent when an organ must be removed” (MedlinePlus.gov, 2017) Week 5 Monday: “If you have diabetes, your blood glucose, or blood sugar, levels are too high. Over time, this can damage the covering on your nerves or the blood vessels that bring oxygen to your nerves. Damaged nerves may stop sending messages or may send messages slowly or at the wrong times” (MedlinePlus.gov, 2017). *The number of sessions*: 24 unique text messages *Duration of each session on average*: NA *Duration of the entire intervention*: 6 weeks *Duration of the entire intervention short vs long*: Short *Intervention deliverer*: NR *Intervention form*: Text messages	
Outcomes	*Perceived Sense of Stress (PSS)* **Outcome type**: Continuous Outcome	
Identification	**Sponsorship source:** NR **Country:** USA **Setting:** two urban health care systems located in western Pennsylvania and southern California. **Comments:** NR **Authors name:** Lisa Concil **Institution:** Lecturer and Clinical Instructor, San Diego State University **Email:** 10006 Maya Linda Road, #5207, San Diego, CA 92126 **Address:** lconcilio@sdsu.edu **Time period:** NR	
Notes	PSS included in analysis 3.1	
***Risk of bias***		
**Bias**	**Authors' judgement**	**Support for judgement**
Random sequence generation (selection bias)	Low risk	Quote: "Randomization was conducted using an online random sequence generator (Random.org, 2019)."
Allocation concealment (selection bias)	Unclear risk	Quote: "A $20 gift card was sent to participants who completed the study requirements. Additionally, the first four participants who completed the study at the end of week 6 were awarded another $50 gift card." We assume that consecutive nurses were randomised. Difficult to judge whether nurses or the investigator could possibly foresee assignment.
Blinding of participants and personnel (performance bias) All outcomes	Unclear risk	Unclear whether participants were blinded. The control group received 'placebo' text messages.
Blinding of outcome assessment (detection bias) All outcomes	Unclear risk	Unclear whether participants were blinded whereas outcomes were self‐reported. The control group received 'placebo' text messages.
Incomplete outcome data (attrition bias) All outcomes	Low risk	Attrition rate is low. (21/22 were included in the analysis). Reason provided. Not reported whether the participant that was lost to follow‐up differed from the other participants.
Selective reporting (reporting bias)	Unclear risk	No trial registration or no study protocol reported, nor did we find one online. No indication of selective reporting.
Other bias	Unclear risk	Not reported whether there were any baseline differences between groups on stress, gender, age.

**Table:** Copeland 2021

***Study characteristics***		
Methods	**Study design:** randomised controlled trial **Study grouping:** parallel group	
Participants	**Baseline characteristics** Meditation *Age (mean ± SD)*: NR *Sex (N (% female))*: 4 (100%) *Sample size*: 4 *Years of experience (mean ± SD)*: NR Journal *Age (mean ± SD)*: NR *Sex (N (% female))*: 4 (100%) *Sample size*: 4 *Years of experience (mean ± SD)*: NR Outside *Age (mean ± SD)*: NR *Sex (N (% female))*: 5 (100%) *Sample size*: 5 *Years of experience (mean ± SD)*: NR Gratitude *Age (mean ± SD)*: NR *Sex (N (% female))*: 5 (100%) *Sample size*: 5 *Years of experience (mean ± SD)*: NR Control (no intervention) *Age (mean ± SD)*: NR *Sex (N (% female))*: 2 (100%) *Sample size*: 2 *Years of experience (mean ± SD)*: NR Overall *Age (mean ± SD)*: 44.4 ± 11.4 *Sex (N (% female))*: 20 (100%) *Sample size*: 20 *Years of experience (mean ± SD)*: 2.24 ± 0.88 **Included criteria:** Full and part‐time nurses and nurse aides working any shift at a suburban, 225 bed, Level 1 trauma centre were eligible to participate. **Excluded criteria:** NR **Pretreatment:** NR **Compliance rate:** 3 of the 18 nurses assigned to an intervention group did not complete the six‐week intervention (17%) **Response rate:** NR **Type of healthcare worker:** exclusively nurses	
Interventions	**Intervention characteristics** Meditation *Type of the intervention*: Intervention type 2 ‐ to focus one’s attention away from the experience of stress *Description of the intervention*: Participants were asked to meditate for approximately five minutes at work every day they worked during the six‐week period. Meditation was described as a way to enhance mindfulness and become present in the moment. Participants were assured there is no right or wrong way to meditate, it is normal for the mind to wander during meditation, and the most important thing is that they take the time to do it. Participants were asked to download a meditation app on their smartphone (i.e. Simple Habit). The Simple Habit app, for example, allows users to identify how much time they have (five minutes), where they are (work), and what they would like to emphasise (stress, frustration, energy, focus, procrastination). They are then guided through a meditation suited for the choices selected. Participants not wanting to download an app were shown how to search for five‐minute meditations on the web. Participants were instructed to use any quiet, private space available to them (conference room, compassion room, staff lounge) and to turn off any work phones or pagers during this time. Each participant was given a record keeping log to record the date worked, location of meditation, identification of which meditation was done, and length of time spent meditating. *The number of sessions*: every day they worked during the six‐week period *Duration of each session on average*: 5 minutes *Duration of the entire intervention*: 6 weeks *Duration of the entire intervention short vs long*: Short *Intervention deliverer*: NA *Intervention form*: Individual, at work Journal *Type of the intervention*: Intervention type 2 ‐ to focus one’s attention away from the experience of stress *Description of the intervention*: Participants were asked to thank three people and compliment three additional people at work every day they worked during the six‐week period. It was explained that the act of complimenting or thanking another person can be intrinsically rewarding and motivating. It can also increase a sense of connection/relationship between people. Participants were told that they could compliment and thank any person (colleague, visitor, and patient) they encounter during their work and that they should communicate a positive message. Each participant was given a record‐keeping log to record the date worked and positions, not names, of the people they thanked and complimented. *The number of sessions*: every day they worked during the six‐week period *Duration of each session on average*: 5 minutes *Duration of the entire intervention*: 6 weeks *Duration of the entire intervention short vs long*: Short *Intervention deliverer*: NA *Intervention form*: Individual, at work Outside *Type of the intervention*: Intervention type 2 ‐ to focus one’s attention away from the experience of stress *Description of the intervention*: Participants were asked to take a break outdoors at work every day they worked during the six‐week period. Participants were asked to spend a minimum of five minutes outdoors. Being outdoors was described as an opportunity to disconnect themselves from their work and to recharge themselves. Participants were told they could engage in activity (walking a path) or sit quietly (in the healing garden), but they were to turn off personal phones and work phones/pagers during this time. As the intent is to disconnect and refocus, participants were also asked to limit interaction with others during this time and instead focus on what they could hear, see, or smell around them. Each participant was given a record‐keeping log to record the date worked, location they went to outdoors, and time spent outside. *The number of sessions*: every day they worked during the six‐week period *Duration of each session on average*: 5 minutes *Duration of the entire intervention*: 6 weeks *Duration of the entire intervention short vs long*: Short *Intervention deliverer*: NA *Intervention form*: Individual, at work outside Gratitude *Type of the intervention*: Intervention type 2 ‐ to focus one’s attention away from the experience of stress *Description of the intervention*: Participants were asked to journal for a minimum of five minutes at work every day they worked during the six‐week period. Journaling was described as an opportunity to reflect on their experiences during their work shift and also as an opportunity to take the perspective of “the other”. The journaling could take any form the participant wished. Small three‐ring notebooks were provided with the following prompts glued to the inside cover, although they were not required to be used: how would the patient/visitor/colleague/observer describe this situation; the best thing that happened today was; what I would have done differently if I could; this was unexpected and here’s what I did; the situation that touched me the most today was; would you believe this happened; or the way I got through that was. Participants were asked to date each journal entry to keep track of how often they journaled during the six‐week period. *The number of sessions*: every day they worked during the six‐week period *Duration of each session on average*: 5 minutes *Duration of the entire intervention*: 6 weeks *Duration of the entire intervention short vs long*: Short *Intervention deliverer*: NA *Intervention form*: Individual, at work Control (no intervention) *Type of the intervention*: NA *Description of the intervention*: These participants were asked not to change anything in their work practise for six weeks. *The number of sessions*: NA *Duration of each session on average*: NA *Duration of the entire intervention*: NA *Duration of the entire intervention short vs long*: NA *Intervention deliverer*: NA *Intervention form*: NA	
Outcomes	*ProQOL ‐ Burnout* **Outcome type**: ContinuousOutcome *ProQOL ‐ Compassion Satisfaction* **Outcome type**: ContinuousOutcome *ProQOL ‐ Secondary Trauma* **Outcome type**: ContinuousOutcome	
Identification	**Sponsorship source:** The author(s) received no financial support for the research, authorship, and/or publication of this article. **Country:** United States of America **Setting:** 1 trauma center **Comments:** NR **Authors name:** Darcy Copeland **Institution:** College of Natural and Health Sciences, University of Northern Colorado **Email:** darcy.copeland@unco.edu **Address:** Gunter Hall 3080, Campus Box 125, Greeley, CO 80639, USA **Time period:** NR	
Notes	Pro_QOL_BO included in analysis 2.1.Intervention groups combined to create a single pair‐wise comparison	
***Risk of bias***		
**Bias**	**Authors' judgement**	**Support for judgement**
Random sequence generation (selection bias)	Low risk	Quote: "As participants were enrolled, they were randomized, using a list of random numbers generated from an online random number generator, into one of five groups: meditation, outside, gratitude, journal, or control."
Allocation concealment (selection bias)	Unclear risk	Quote: "Upon receipt of IRB approval, nurses and nurse aids were invited to participate via organizational email. As interested participants contacted the PI, they were screened for eligibility and a time was arranged to complete informed written consent procedures, the pre‐intervention assessment, and receive intervention instructions. As participants were enroled, they were randomized, using a list of random numbers generated from an online random number generator, into one of five groups: meditation, outside, gratitude, journal, or control." As a list of random numbers was used it is assumed that the person who randomised patients could foresee allocation it is however unclear who performed randomisation.
Blinding of participants and personnel (performance bias) All outcomes	High risk	Participants were not asked to change any other practise while at work; participants assigned to one intervention were not expressly discouraged from engaging in the other interventions. Participants were not blinded.
Blinding of outcome assessment (detection bias) All outcomes	High risk	Participants were not blinded whereas outcomes were self‐reported
Incomplete outcome data (attrition bias) All outcomes	Low risk	2 of the 22 participants were not included in the analysis (9%). Reasons were provided. Participants who did not complete the study were more often male however loss to follow‐up is below our pre‐defined cut‐off point.
Selective reporting (reporting bias)	Unclear risk	No trial registration or no study protocol reported, nor did we find one.
Other bias	Unclear risk	Not recorded whether there were differences at baseline between groups.

**Table:** Dahlgren 2022

***Study characteristics***		
Methods	**Study design:** randomised controlled trial **Study grouping:** parallel group	
Participants	**Baseline characteristics** Proactive recovery programme *Age (mean ± SD)*: 27.5 ± 5.3 *Sex (N (% female))*: (NR) 85% *Sample size*: 99 *Years of experience (mean ± SD)*: 2.8 ± 2.1 Control (no intervention) *Age (mean ± SD)*: 27.0 ± 5.1 *Sex (N (% female))*: (NR) 91% *Sample size*: 108 *Years of experience (mean ± SD)*: 3.3 ± 2.7 Overall *Age (mean ± SD)*: NR *Sex (N (% female))*: NR *Sample size*: 207 *Years of experience (mean ± SD)*: NR **Included criteria:** RNs with less than 12 months’ work experience were eligible to participate. **Excluded criteria:** NR **Pretreatment:** no significant differences were observed between the two groups at baseline for any of the background variables or any of the outcome measures at baseline. **Compliance rate:** 36 of the 99 (36%) participants randomised to the intervention group participated in two of the three sessions. **Response rate:** 45% (207/461) **Type of healthcare worker:** exclusively nurses	
Interventions	**Intervention characteristics** Proactive recovery programme *Type of the intervention*: Intervention type 2 ‐ to focus one’s attention away from the experience of stress *Description of the intervention*: The sessions had three main focus': (1) unwinding from stress, including detach‐ment from thoughts of work during free time; (2) supporting sleep in relation to homoeostatic and circadian processes; and (3) handling fatigue and increasing recovery behaviours (see table1). Psycho‐educative elements were interspersed with group discussions and exercises. Participants were encouraged to reflect on their habitual behaviours connected to sleep and recovery and possible alternatives. Between sessions, the participants were encouraged to try strategies or behaviour changes of their choice, with the aim of enhancing sleep and recovery. During the second and third sessions, participants reflected on the experience of trying new strategies. All participants received written material covering the content of each session, as well as online access to an adapted version of a bio‐mathematical model (ArturNurse). ArturNurse evaluated fatigue risk levels based on their work schedules25 and provided suggestions of strategies from the programme on how to optimise sleep in relation to different shifts. *The number of sessions*: 3 *Duration of each session on average*: 2.5 hours *Duration of the entire intervention*: 4 weeks *Duration of the entire intervention short vs long*: Short *Intervention deliverer*: Authors (Bachelor of applied psychology) *Intervention form*: Group, face‐to‐face Control (no intervention) *Type of the intervention*: NA *Description of the intervention*: NA *The number of sessions*: NA *Duration of each session on average*: NA *Duration of the entire intervention*: NA *Duration of the entire intervention short vs long*: NA *Intervention deliverer*: NA *Intervention form*: NA	
Outcomes	*Perceived Stress Scale (PSS)* **Outcome type**: Continuous Outcome *Shirom‐Melamed Burn‐out Questionnaire (SMBQ) ‐ Global* **Outcome type**: ContinuousOutcome	
Identification	**Sponsorship source:** This study was funded by AFA Försäkring (150024) **Country:** Sweden **Setting:** Eight Swedish hospitals **Comments:** NR **Authors name:** Anna Dahlgren **Institution:** Department of Clinical Neuroscience, Karolinska Institute, Stockholm, Solna, Sweden **Email:** anna.dahlgren@ki.s **Address:** NR **Time period:** 2017‐2018	
Notes	PSS included in analysis 2.1 and 2.2	
***Risk of bias***		
**Bias**	**Authors' judgement**	**Support for judgement**
Random sequence generation (selection bias)	High risk	Excel generator for random allocation to groups was used by the research team. Based on a previous feasibility study, adjustments to the process of random group allocation were made if many nurses from the same ward were initially allocated to one group. Twenty adjustments were also made for participants who were randomised to the intervention group but knew that they could not attend the group sessions. They were moved to the control group and replaced by a random participant from the control group.
Allocation concealment (selection bias)	High risk	Quote: "parallel randomised control trial was designed to include 100 participants in each group (intervention and wait list control) to detect moderate effect sizes (Cohen’s d = 0.5) resulting in a power of 0.94. Excel generator for random allocation to groups was used by the research team. Based on a previous feasibility study, adjustments to the process of random group allocation were made if many nurses from the same ward were initially allocated to one group. Twenty adjustments were also made for participants who were randomised to the intervention group but knew that they could not attend the group sessions. They were moved to the control group and replaced by a random participant from the control group. Adjustments were made for 24 participants. Masking was not applicable. After the follow‐up measure the control group received the intervention."
Blinding of participants and personnel (performance bias) All outcomes	High risk	'Masking was not possible'. Participants were not blinded.
Blinding of outcome assessment (detection bias) All outcomes	High risk	Participants were not blinded whereas outcomes are self‐reported.
Incomplete outcome data (attrition bias) All outcomes	High risk	130 of the 207 randomised participants (63%) completed 6 months follow‐up. Reasons not provided as well as whether it was at random.
Selective reporting (reporting bias)	High risk	Trial registration (https://clinicaltrials.gov/ct2/show/NCT04246736). In the trial registration many other stress outcomes were mentioned, which were not mentioned in this study, i.e. Change in stress and energy (subjective measures) [Time Frame: In order to detect change measures were made at baseline and four weeks after the intervention.] Stress‐Energy rating questionnaire: Minimum score 0, maximum score 5. Higher scores indicate more stress and more energy. Change in stress symptoms (subjective measures) [Time Frame: In order to detect change measures were made at baseline and four weeks after the intervention.] Self‐ratings of stress symptoms/absence of stress symptoms (single items): "Tense"; "Irritated"; "Exhausted"; "Hard to disconnect from thoughts of work during spare time"; "Emotional burden"; "Relaxed/calm" on a scale ranging from 1 = not at all, and 5 = very much. Measured every day during seven days at baseline and seven days at follow‐up. Change in diurnal levels of stress (subjective measures) [Time Frame: In order to detect change measures were made at baseline and four weeks after the intervention.] Self‐rated stress scale for repeated measurement, measured one a nine‐graded scale with values ranging from 1 = very low stress to 9 = very high stress. Measured every third hour during wake time seven days at baseline and seven days at follow‐up. Change in stress (objective measures) [Time Frame: In order to detect change measures were made at baseline and four weeks after the intervention.] Hair cortisol based on 2 cm segments (pg/mg)
Other bias	Unclear risk	Judgement Comment: Low response rate 207/461 (45%)

**Table:** deSouza 2021

***Study characteristics***		
Methods	**Study design:** randomised controlled trial **Study grouping:** parallel group	
Participants	**Baseline characteristics** Massage *Age (mean ± SD)*: NR *Sex (N (% female))*: 30 (100%) *Sample size* : 30 *Years of experience (up to 3 years, 4 to 6 years, over 6 years)*: 5 (17%), 14 (47%), 11 (37%) Control (no intervention) *Age (mean ± SD)*: NR *Sex (N (% female))*: 30 (100%) *Sample size* : 30 *Years of experience (up to 3 years, 4 to 6 years, over 6 years)*: 12 (40%), 13 (43%), 5 (17%) Overall *Age (mean ± SD)*: 34.2 ± 5.3 *Sex (N (% female))*: 60 (100%) *Sample size*: 60 *Years of experience (up to 3 years, 4 to 6 years, over 6 years)*: NR **Included criteria:** The inclusion criteria were the following: woman age 20 to 45; working in day‐time shift (morning, afternoon, 12‐hour shift), LSS score ≥ 40, time working in the hospital at least one year, a 30‐day interval between returning from vacation or medical level. **Excluded criteria:** The exclusion criteria were: smokers, hypertensive individuals, use of glucocorticoids, beta‐blockers, psychoactive drugs in the last three months, pregnant, or hysterectomised woman, use of integrative practises (acupuncture, massage, herbal therapy, Reiki, or floral therapy amongst others) for at least two months. **Pretreatment:** the groups were homogenous regarding the qualitative variables used to describe the population ‐ age, BMI, LSS, and BPI ‐ except for the pain interference component in enjoyment of life (P = 0.037). **Compliance rate:** two of the 30 missed a massage session (6.7%) **Response rate:** 60 of the 91 eligible patients participated (66%) **Type of healthcare worker:** exclusively nurses	
Interventions	**Intervention Characteristics** Massage *Type of the intervention*: Intervention type 2 ‐ to focus one’s attention away from the experience of stress *Description of the intervention*: Chair massage sessions performed after the work shift or at lunchtime in a room especially designated for this purpose *The number of sessions*: 6 *Duration of each session on average*: 15 minutes *Duration of the entire intervention*: 3 weeks *Duration of the entire intervention short vs long*: Short *Intervention deliverer*: Therapist *Intervention form*: Individual Control (no intervention) *Type of the intervention*: NA *Description of the intervention*: The control group received no treatment. *The number of sessions*: NA *Duration of each session on average*: NA *Duration of the entire intervention*: NA *Duration of the entire intervention short vs long*: NA *Intervention deliverer*: NA *Intervention form*: NA	
Outcomes	*The List of Stress Symptoms (LSS)* **Outcome type**: ContinuousOutcome	
Identification	**Sponsorship source:** The study was funded by the Research Support Foundation of the State São Paulo (Process No 2017/19, 645‐2) **Country:** Brazil **Setting:** Two teaching cancer hospitals **Comments:** NR **Authors name:** Borges de Souza **Institution:** Nursing course of the Santa Casa de São Paulo school of medicine sciences **Email:** Talita.Paverni@fcmsantacasasp.edu.br **Address:** Rua dr. Cesario Motta 3r, 61, 01221‐020 **Time period:** 2017‐2018	
Notes	LSS included in analysis 2.1	
***Risk of bias***		
**Bias**	**Authors' judgement**	**Support for judgement**
Random sequence generation (selection bias)	Low risk	The volunteers who met the inclusion criteria were numbered for the two‐group simple randomization (http://www.randomizer.org). After this, one of the researchers created a sequential numerical list: the first participant was randomly chosen and the rest were included in the list as they met the eligibility criteria and according to the groups of the randomized list.
Allocation concealment (selection bias)	Unclear risk	The volunteers who met the inclusion criteria were numbered for the two‐group simple randomization (http://www.randomizer.org). After this, one of the researchers created a sequential numerical list: the first participant was randomly chosen and the rest were included in the list as they met the eligibility criteria and according to the groups of the randomized list. Insufficient information to understand whether intervention allocations could have been foreseen in advance of, during, enrolment.
Blinding of participants and personnel (performance bias) All outcomes	Low risk	Participants were blinded.
Blinding of outcome assessment (detection bias) All outcomes	Low risk	The study was blinded to researchers, participants and statistician.
Incomplete outcome data (attrition bias) All outcomes	Unclear risk	12 of the initial 60 participants discontinued the study (20%), reasons provided but not whether they differed with responders. After that another 12 participants was randomized and included in the analysis. The baseline characteristics are provided for 30 participants, but it is unclear which participants are included here.
Selective reporting (reporting bias)	Low risk	In the trial protocol, the authors mention that salivary cortisol is, alongside the LSS, the primary outcome. Salivary not reported in this article. https://ensaiosclinicos.gov.br/rg/RBR-3bjjf4 but is not one our outcome measures so no selective reporting on outcomes that we are interested in.
Other bias	Low risk	Judgement Comment: No indication of other sources of bias.

**Table:** Dincer 2021

***Study characteristics***		
Methods	**Study design:** randomised controlled trial **Study grouping:** parallel group	
Participants	**Baseline characteristics** Emotional Freedom Techniques *Age (mean ± SD)*: 33.5 ± 9.8 *Sex (N (% female))*: 32 (91%) *Sample size*: 35 *Years of experience (mean ± SD)*: NR Control (wait list) *Age (mean ± SD)*: 33.4 ± 9.6 *Sex (N (% female))*: 32 (87%) *Sample size*: 37 *Years of experience (mean ± SD)*: NR Overall *Age (mean ± SD)*: 33.5 ± 9.6 *Sex (N (% female))*: 64 (89%) *Sample size*: 72 *Years of experience (mean ± SD)*: NR **Included criteria:** nurses caring for COVID‐19 patients. Inclusion criteria were: a) not having any psychiatric diagnoses, b) not taking any courses about coping with anxiety and stress, and c) volunteering to participate in the study **Excluded criteria:** NR **Pretreatment:** no statistically significant pre‐intervention differences were found between the groups on demographic variables. The pre‐test stress level, anxiety level and the burn‐out score did not differ significantly between the groups. **Compliance rate:** five of the 40 participants did not attend the EFT sessions (13%) **Response rate:** 100% **Type of healthcare worker:** exclusively nurses caring for COVID‐19 patients	
Interventions	**Intervention characteristics** Emotional Freedom Techniques *Type of the intervention*: Intervention type 2 ‐ to focus one’s attention away from the experience of stress *Description of the intervention*: Each 5‐person group began by having the participants complete the pre‐test SUD, the STAI‐I, and the burnout scale via SurveyMonkey. EFT was applied to each group of nurses in a single session of approximately 20 min. At the end of the session, participants again completed the post‐test SUD, the STAI‐I, and the burn‐out scale The EFT session began by presenting the participants with a picture of the acupressure points (Fig. 2) and showing them how to gently tap on them using their index and middle fingers. After this demonstration, the participants followed the basic steps of an EFT session, following the researcher’s example: Identify an anxiety‐evoking issue and determine the SUD level. Creating a personal acceptance and reminder statement in the general form of "I accept myself despite this.........." Tapping seven times on each acupressure point shown in Fig. 2. After tapping these points, the affirmation/reminder statement is repeated. A sequence of physical movements and vocalisations called“The Nine Gamut Procedure” is carried out. Steps 3 and 4 are repeated. Another SUD rating is given. *The number of sessions*: 1 *Duration of each session on average*: 20 min *Duration of the entire intervention*: 20 min *Duration of the entire intervention short vs long*: Short *Intervention deliverer*: First author certified in Emotional Freedom Techniques *Intervention form*: Online group Control (wait list) *Type of the intervention*: control *Description of the intervention*: participants in the control group were asked to stay comfortable in a calm and tranquil environment for the next 15 min. *The number of sessions*: 1 *Duration of each session on average*: 15 min *Duration of the entire intervention*: 15 min *Duration of the entire intervention short vs long*: short *Intervention deliverer*: NR *Intervention form*: NR	
Outcomes	*State Anxiety Scale* **Outcome type**: Continuous Outcome *The Burnout Scale* **Outcome type**: Continuous Outcome *Subjective Units of Distress Scale* **Outcome type**: Continuous Outcome	
Identification	**Sponsorship source:** This research was not funded. **Country:** Turkey **Setting:** A university hospital **Comments:** NR **Authors name:** Berna Dincer **Institution:** Department of Internal Medicine Nursing, Faculty of Health Science, Istanbul Medeniyet University, Istanbul, Turkey **Email:** berna.dincer@medeniyet.edu.tr **Address:** 38, Tıbbiye Street, Istanbul, Uskudar 34668, Istanbul, Turkey **Time period:** 2020	
Notes	Subjective Units of Distress Scale included in analysis 2.1 STAI included in analysis 2.3	
***Risk of bias***		
**Bias**	**Authors' judgement**	**Support for judgement**
Random sequence generation (selection bias)	Low risk	Eighty nurses who met the inclusion criteria were assigned to groups using an online random number generator.
Allocation concealment (selection bias)	Unclear risk	Insufficient information to understand whether intervention allocations could have been foreseen in advance of, during, enrolment.
Blinding of participants and personnel (performance bias) All outcomes	High risk	Quote: "After completing the Descriptive Characteristics Form online, a time for the meeting was determined in collaboration with the participants in each subgroup. They were also asked to stay comfortable in as calm and tranquil an environment as possible during the session. The EFT treatment was provided by the ﬁrst author, who was certiﬁed in EFT. Each 5‐person group began by having the participants complete the pre‐test SUD, the STAI‐I, and the burnout scale via SurveyMonkey." Participants were not blinded. Baseline questionnaire filled in after randomisation.
Blinding of outcome assessment (detection bias) All outcomes	High risk	Quote: "The analysis was conducted by a researcher who was blind to group assignment." Participants were not blinded whereas outcomes were self‐reported.
Incomplete outcome data (attrition bias) All outcomes	Low risk	10% lost‐to‐follow‐up however unknown whether this was at random however loss to follow‐up is below our pre‐defined cut‐off point.
Selective reporting (reporting bias)	High risk	No intention to tread analysis. Participants randomised to the intervention group that did not attend the EFT session (n = 5) were excluded.
Other bias	Low risk	No indication of other sources of bias

**Table:** Duchemin 2015

***Study characteristics***		
Methods	**Study design:** randomised controlled trial **Study grouping:** parallel group	
Participants	**Baseline Characteristics** Mindfulness‐based intervention *Age (mean ± SD)*: NR *Sex (N (% female))*: NR *Sample size*: NR *Years of experience (mean ± SD)*: NR Control (wait list) *Age (mean ± SD)*: NR *Sex (N (% female))*: NR *Sample size*: NR *Years of experience (mean ± SD)*: NR Overall *Age (mean ± SD)*: 44.2 (NR) *Sex (N (% female))*: 28 (88%) *Sample size*: 32 *Years of experience (mean ± SD)*: 14.5 (NR) **Included criteria:** participants were personnel, 18 or older, from the surgical intensive care unit (SICU) of a large academic medical center. Eligibility criteria included any personnel working in the SICU and having contact with the patients or their families. **Excluded criteria:** individuals practising mindfulness, yoga, or exercising more than 30 minutes a day were excluded, as were individuals with third trimester pregnancy or a history of recent surgery if it limited ability to perform the gentle yoga movement **Pretreatment:** there were no significant differences between the two groups for age (P = 0.9496, t = 0.0638), years of experience (P = 0.9485, t = 0.06512), or years working in the SICU (P = 0.8702, t = 0.1648). **Compliance rate:** NR **Response rate:** not able to assess as the exact number of eligible individuals is not reported. More than 200 individuals working in the SICU were eligible to participate and were informed about the study through flyers and information provided at staff meetings. Thirty‐two individuals were interested in participating and all were eligible to participate because of minimal exclusion criteria to reflect real workplace conditions. **Type of healthcare worker:** various healthcare staff but 69% nurses	
Interventions	**Intervention characteristics** Mindfulness‐based intervention *Type of the intervention*: Intervention type 2 ‐ to focus one’s attention away from the experience of stress *Description of the intervention*: The 8‐week group MBI combines a didactic introduction/discussion and a combination of mindfulness and yoga practises with music at each session16. The intervention was delivered by M Klatt, a trained mindfulness and certified yoga instructor, who developed the MBI to be pragmatically performed in a work setting. The protocol combines elements of mindfulness meditation, yoga movements, and relaxation through music. All sessions last 1 hour except for week five sessions that lasts 2 hours and includes mindful eating. After introduction of the weekly theme/prompt, the participants are led through a body scan, gentle stretching, yoga, progressive relaxation, and/or an eating meditation (for the two‐hour session), and then into formal meditation. Each week a different topic is highlighted. The music is standardized to be the same background music in each session, and in the background of each meditation practise contained on CDs, which were provided to participants to facilitate daily practise. The intervention is 8 weeks in length, paralleling the mindfulness‐based‐stress‐reduction (MBSR) traditional program, with shortening of the group session duration for the setting. Participants are asked to perform 20‐minute daily individual practises if possible. The group stress‐reduction sessions were delivered at the workplace during work hours. Work coverage was assured for the participants during the time of the group sessions and assessments *The number of sessions*: 8 *Duration of each session on average*: 1 hour except for week five sessions that lasted two hours. Participants are asked to perform 20‐minute daily individual practises if possible. *Duration of the entire intervention*: 8 weeks *Duration of the entire intervention short vs long*: short *Intervention deliverer*: Trained mindfulness and certified yoga instructor *Intervention form*: Group, face‐to‐face, at work during work hours Control (wait list) *Type of the intervention*: NA *Description of the intervention*: Participants randomly assigned to the control wait‐list group received the mindfulness sessions after the first group had finished their eight‐week intervention and after completion of the second set of assessments *The number of sessions*: NA *Duration of each session on average*: NA *Duration of the entire intervention*: NA *Duration of the entire intervention short vs long*: NA *Intervention deliverer*: NA *Intervention form*: NA	
Outcomes	*DASS ‐ stress* **Outcome type**: Continuous Outcome *Perceived Stress Scale* **Outcome type**: Continuous Outcome *Maslach Burnout Inventory ‐ emotional exhaustion* **Outcome type**: Continuous Outcome *Maslach Burnout Inventory ‐ depersonalization* **Outcome type**: ContinuousOutcome *Maslach Burnout Inventory ‐ Personal accomplishment* **Outcome type**: Continuous Outcome *Professional Quality of Life (ProQOF)* **Outcome type**: Continuous Outcome	
Identification	**Sponsorship source:** Funding: Funded in part by the OSU Harding Behavioral Health Stress, Trauma and Resilience program **Country:** Unites States **Setting:** A large academic medical center **Comments:** NR **Authors name:** Anne‐Marie Duchemin **Institution:** Department of Psychiatry, The Ohio State University, **Email:** anne‐marie.duchemin@osumc.edu **Address:** 1670 Upham Drive, Columbus, OH 43210, USA. **Time period:** NR	
Notes	Not able to include in analysis due to missing data.	
***Risk of bias***		
**Bias**	**Authors' judgement**	**Support for judgement**
Random sequence generation (selection bias)	Low risk	Quote: "Eligible participants were randomized 1:1 using Graphpad software to intervention group or waiting list control group, with stratification by gender and type of work. Assessments were performed for all"
Allocation concealment (selection bias)	Unclear risk	Quote: "Eligible participants were randomized 1:1 using Graphpad software to intervention group or waiting list control group, with stratification by gender and type of work. Assessments were performed for all participants," Difficult to judge whether participants and/or investigators could possibly foresee assignment.
Blinding of participants and personnel (performance bias) All outcomes	High risk	Participants were not blinded.
Blinding of outcome assessment (detection bias) All outcomes	High risk	Quote: "Questionnaires and samples were coded." Participants were not blinded whereas outcomes are self‐reported.
Incomplete outcome data (attrition bias) All outcomes	Low risk	Quote: "There was no drop‐out and all participants completed the 2 sets of assessments."
Selective reporting (reporting bias)	Unclear risk	No trial registration or study protocol reported, nor did we find one online
Other bias	Unclear risk	Quote: "was considered statistically significant. RESULTS More than 200 individuals working in the SICU were eligible to participate and were informed about the study through flyers and information provided at staff meetings. Thirty‐two individuals were interested in participating and all were eligible to participate due to minimal exclusion criteria to reflect real workplace conditions. Participants (n = 32) were" Not able to assess the response rate as the exact number of eligible individuals is not reported. Compliance not reported.

**Table:** Dunne 2019

***Study characteristics***		
Methods	**Study design:** randomised controlled trial **Study grouping:** parallel group	
Participants	**Baseline characteristics** Attention‐based training (ABT) *Age in years*: NR *Sex (% female)*: NR *Sample size*: 29 *Years of experience*: NR Control (wait list) *Age in years*: NR *Sex (% female)*: NR *Sample size*: 29 *Years of experience*: NR Overall *Age in years*: NR *Sex (% female)*: NR *Sample size*: 58 *Years of experience*: NR **Included criteria:** a current staff member of the emergency department of St. James’ Hospital; preference to participate in the study and to be over 18 years of age **Excluded criteria:** alcohol or substance abuse within the past 6 months; more than four consecutive classes of meditation or other mind–body practises (including yoga and Tai‐chi) in the past two years; a diagnosis of schizophrenia; currently using (at time of enrolment) anti‐psychotic medication or recently started on anti‐depressant meditation (less than three months at the time of enrolment). Participants on a stable dose of anti‐depressant medication (for more than three months) were permitted but advised to consult with their general practitioner or psychiatrist prior to enrolment. **Pretreatment:** NR **Type of healthcare worker:** exclusively emergency department healthcare worker **Response rate:** 100% **Compliance rate:** 16 of the 29 followed at least two sessions > 55%	
Interventions	**Intervention characteristics** Attention‐based training (ABT) *Type of the intervention*: Intervention type 2 ‐ to focus one’s attention away from the experience of stress *Description of the intervention*: Each session consisted of ABT practise and discussion on the importance of focused attention and the meaning of healthcare. ABT practise involved repeatedly focusing one’s attention on a chosen non‐English phrase (maranatha). *The number of sessions*: 4 *Duration of each session on average*: Two 20‐minute sessions over seven days (280 min in total). *Duration of the entire intervention*: seven weeks *Duration of the entire intervention short vs long*: Short *Intervention deliverer*: Education specialist, meditation expert and a healthcare professional. *Intervention form*: Group ‐ face‐to‐face Control (wait list) *Type of the intervention*: Wait‐list control *Description of the intervention*: NA *The number of sessions*: NA *Duration of each session on average*: NA *Duration of the entire intervention*: NA *Duration of the entire intervention short vs long*: NA *Intervention deliverer*: NA *Intervention form*: NA	
Outcomes	*Maslach Burnout Inventory ‐ Emotional Exhaustion* **Outcome type**: Continuous Outcome *Maslach Burnout Inventory ‐ Depersonalisation* **Outcome type**: Continuous Outcome *Maslach Burnout Inventory ‐ Personal accomplishment* **Outcome type**: Continuous Outcome *DASS ‐ stress* **Outcome type**: Continuous Outcome *DASS ‐ anxiety* **Outcome type**: Continuous Outcome	
Identification	**Sponsorship source:** NR **Country:** Ireland **Setting:** Hospital **Comments:** NR **Authors name:** PJ Dunne **Institution:** Trinity Translational Medicine Institute, Trinity College **Email:** padraicdunne@rcsi.com **Address:** Dublin D08 W9RT, Ireland **Time period:** NR	
Notes	The authors kindly referred to the supplementary file. MBI‐EE included in analysis 2.1 DASS anxiety included in analysis 2.3	
***Risk of bias***		
**Bias**	**Authors' judgement**	**Support for judgement**
Random sequence generation (selection bias)	Low risk	Quote: "Emergency MDT participants were stratiﬁed by role and gender and allocated to intervention or no‐treatment control group using an online randomization tool [17]. Volunteers"
Allocation concealment (selection bias)	Unclear risk	Quote: "Emergency MDT participants were stratiﬁed by role and gender and allocated to intervention or no‐treatment control group using an online randomization tool [17]." Insufficient information to understand whether intervention allocations could have been foreseen in advance of, during, enrolment.
Blinding of participants and personnel (performance bias) All outcomes	High risk	Participants were not blinded.
Blinding of outcome assessment (detection bias) All outcomes	High risk	Participants were not blinded whereas outcomes are self‐reported
Incomplete outcome data (attrition bias) All outcomes	Low risk	Quote: "There was no signiﬁcant difference between study completers and withdrawers." 42 of the 58 participants were analysed. Response rate 72%. However, missing at random according to the authors.
Selective reporting (reporting bias)	Unclear risk	The authors report a trial registration, nor did we find one online
Other bias	Unclear risk	Unclear whether participants differ on baseline characteristics.

**Table:** Dyrbye 2016

***Study characteristics***		
Methods	**Study design:** randomised controlled trial **Study grouping:** parallel group	
Participants	**Baseline characteristics** Intervention *Age in years (%) 31‐40, 41‐50, 51‐60, > 60*: 34 (25.2%) 41 (30.4%) 40 (39.6%) 20 (14.8%) *Sex (N (% female))*: 48 (35.6%) *Sample size*: 145 *Years of experience (%) < 5, 5‐10, 11‐20, 21‐30, > 30*: 28 (20.7%), 32 (23.7%), 43 (31.9%), 25 (18.5%), 7 (5.2%) Control (no intervention) *Age in years (%) 31‐40, 41‐50, 51‐60, > 60*: 43 (31.4%), 44 (32.1%), 34 (24.8%), 6 (11.7%) *Sex (N (% female))*: 40 (29.2%) *Sample size*: 145 *Years of experience (%) < 5, 5‐10, 11‐20, 21‐30, > 30*: 3 (16.8%), 37 (27.0%), 49 (35.8%), 19 (13.9%), 9 (6.6%) Overall *Age in years (%) 31‐40, 41‐50, 51‐60, > 60*: NR *Sex (N (% female))*: NR *Sample size*: 290 *Years of experience (%) < 5, 5‐10, 11‐20, 21‐30, > 30*: NR **Included criteria:** NR **Excluded criteria:** NR **Pretreatment:** Reported socio demographic baseline characteristics of participants randomised to the intervention group were similar to socio demographic baseline characteristics of participants randomised to the control group **Compliance rate:** We could not determine if participants in the intervention arm actually completed their chosen weekly micro‐tasks **Response rate:** NR **Type of healthcare worker:** physicians from various disciplines	
Interventions	**Intervention characteristics** Intervention *Type of intervention*: Intervention type 1: to focus one’s attention on the experience of stress *Description of the intervention*: Menu of five to six self‐directed micro‐tasks and were asked to select and complete one task of their choosing weekly and intentionally designed to cultivate professional satisfaction and well‐being in one of six domains: Promote meaning in work and job satisfaction, Foster teamwork and social support at work, Nurture personal relationships and work‐life balance, Recognise and build on personal strengths (courage, honesty, patience, wisdom, humanity, justice, and transcendence), Encourage effective problem‐solving, and Promote positive emotions *The number of sessions*: six tasks *Duration of each session on average*: five to seven minutes *Duration of the entire intervention:* 10 weeks *Duration of the entire intervention short vs long*: Short *Intervention deliverer*: NR *Intervention form*: Individual (digital) Control (no intervention) *Type of intervention*: No intervention *Description of the intervention*: NA *The number of sessions*: NA *Duration of each session on average*: NA *Duration of the entire intervention*: NA *Duration of the entire intervention short vs long*: NA *Intervention deliverer*: NA *Intervention form*: NA	
Outcomes	*Maslach Burnout Inventory ‐ Emotional exhaustion* **Outcome type**: Continuous Outcome *Maslach Burnout Inventory ‐ Personal accomplishment (lack of)* **Outcome type**: Continuous Outcome *Maslach Burnout Inventory ‐ Depersonalisation* **Outcome type**: Continuous Outcome	
Identification	**Sponsorship source:** NR **Country:** USA **Setting:** Mayo Clinic Departments of Medicine in Minnesota and Arizona and Mayo Clinic Department of Surgery in Minnesota **Comments:** NR **Authors name:** Dyrbye LN, West CP, Richards ML, Ross HJ, Satele D, Shanafelt TS **Institution:** Mayo Clinic **Email:** Dyrbye.liselotte@mayo.edu **Address:** 200 Second Street SW, Rochester, Minnesota, 55905, United States **Time period:** NR	
Notes	MBI‐EE included in analysis 1.1	
***Risk of bias***		
**Bias**	**Authors' judgement**	**Support for judgement**
Random sequence generation (selection bias)	Low risk	Quote: "Participants were randomized to an intervention group or a control group using a computer‐generated algorithm. Randomization was stratiﬁed by speciality (Internal Medicine or Surgery), campus (Rochester or Arizona), and baseline response to the single item, “The work I do is meaningful to me” (from the Empowerment at Work Scale (Spreitzer, 1995)). All participants were asked to complete baseline and end‐of‐study (three month) survey. For both surveys consented participants received an e‐mailed cover letter with a link to a web‐based survey."
Allocation concealment (selection bias)	Unclear risk	Insufficient information to understand whether intervention allocations could have been foreseen in advance of, during, enrolment.
Blinding of participants and personnel (performance bias) All outcomes	High risk	Participants were not blinded.
Blinding of outcome assessment (detection bias) All outcomes	High risk	Participants were not blinded whereas outcomes are self‐reported.
Incomplete outcome data (attrition bias) All outcomes	Low risk	Over 90% of participants in each arm completed both the baseline and end‐of‐study surveys.
Selective reporting (reporting bias)	Unclear risk	No protocol registration, nor did we find one online.
Other bias	Unclear risk	Response rate not reported. Quote: We could not determine if participants in the intervention arm actually completed their chosen weekly micro‐tasks

**Table:** Dyrbye 2019

***Study characteristics***		
Methods	**Study design:** randomised controlled trial **Study grouping:** parallel group	
Participants	**Baseline characteristics** Coaching *Age (31‐40, 41‐50, 51‐60, > 60)*: 7/44 (15.9), 25/44 (56.8), 12/44 (27.3), 0/44 (0) *Sex (N (% female))*: 20 (46%) *Sample size*: 44 *Years of experience (mean ± SD)*: 15.8 (7.2) Control (wait list) *Age (31‐40, 41‐50, 51‐60, > 60)*: 7/42 (16.7), 20/42 (47.6), 12/42 (28.6), 3/42 (7.1) *Sex (N (% female))*: 28 (64%) *Sample size*: 44 *Years of experience (mean ± SD)*: 15.7 (8.3) Overall *Age (31‐40, 41‐50, 51‐60, > 60)*: NR *Sex (N (% female))*: NR *Sample size*: NR *Years of experience (mean ± SD)*: NR **Included criteria:** Individuals who had been in practice for 5 to 30 years were eligible **Excluded criteria:** NR **Pretreatment:** no group differences reported **Type of healthcare worker:** exclusively physician **Response rate:** 12% **Compliance rate:** Participants randomised to the intervention group had a mean of 5.5 coaching sessions (range, 0‐6 coaching sessions).	
Interventions	**Intervention characteristics** Coaching *Type of the intervention*: Intervention type 1 ‐ to focus one’s attention on the experience of stress *Description of the intervention*: All coaching sessions were performed by telephone. The initial coaching session focused on creating the relationship, assessing needs, identifying values, setting goals, and creating an action plan. Subsequent sessions followed the same general structure: (1) check in, debrief strategic action the participant had taken since the last session, manage progress, and re‐view accountability; (2) plan and set goals; (3) design actions to incorporate into daily life; (4) commit to next steps; and (5) checkout and summarise. The topics individuals could request coaching on were unscripted and individualised. Coaches made brief notes of the topics discussed. Participants randomised to the intervention group were expected to see the same number of patients as their colleagues who were not in the intervention group. Participants who scheduled their coaching during their clinical time were expected to make up the patient visits by seeing additional patients at other times (e.g. adding extra patients before or after their standard clinical time on other days). Example: optimising meaning in work; aligning values and priorities with work‐related tasks; ensuring work activities align with the aspects of work perceived as most meaningful; reconsidering nonclinical roles, integrating personal and professional life; sharing tasks with partner; meeting needs of ageing parents; reducing work‐home conflicts; building social support and community at work; strategies to network with colleagues; taking breaks at work with colleagues; building peer relationships; addressing stressful relationships with colleagues; improving work efficiency; steps to increase efficiency with email and other tasks; delegating tasks, setting boundaries with patients, collaborating with colleagues, and obtaining additional EHR training; addressing workload; prioritising and saying “no”; avoiding ove‐scheduling; setting expectations; setting goals; establishing roles and responsibilities; building leadership skills; building teams; changing management; influencing leaders; challenging conversations; pursuing hobbies and recreation; finding time and discovering interests; engaging in self‐care; strategising to get exercise; eating healthy, attending to medical needs; strengthening relationships outside of work; proactively scheduling social events with friends; spending more time with family; showing appreciation towards others; being grateful. *The number of sessions*: 6 *Duration of each session on average*: 30 minutes *Duration of the entire intervention*: 5 months *Duration of the entire intervention short vs long*: Long *Intervention deliverer*: Credentialed professional coaches *Intervention form*: Individual by telephone Control (wait list) *Type of the intervention*: Wait list *Description of the intervention*: Participants randomised to the control group received no intervention during the 5 months of the study but were provided with access to Bluepoint coaches for an equivalent number of coaching contact hours (3.5 hours) during the 5 months after the conclusion of the active study interval. *The number of sessions*: NA *Duration of each session on average*: NA *Duration of the entire intervention*: NA *Duration of the entire intervention short vs long*: NA *Intervention deliverer*: NA *Intervention form*: NA	
Outcomes	*Maslach Burnout Inventory ‐ Emotional exhaustion* **Outcome type**: ContinuousOutcome *Maslach Burnout Inventory ‐ Depersonalization* **Outcome type**: ContinuousOutcome *Maslach Burnout Inventory ‐ Personal accomplishment* **Outcome type**: ContinuousOutcome	
Identification	**Sponsorship source:** Funding for this study was provided by the Mayo Clinic Department ofMedicine Program on Physician Well‐Being and thePhysician Foundation **Country:** USA **Setting:** Mixed healthcare settings including the department of medicine, family medicine and pediatric **Comments:** NR **Authors name:** Liselotte N.Dyrbye **Institution:** Department of Medicine, Program on Physician Well‐Being, Mayo Clinic **Email:** dyrbye.liselotte@mayo.edu **Address:** 200 First StSW, Rochester, MN 55905 **Time period:** 2017‐2018	
Notes	We kindly received the mean and SD of the MBI‐EE for both groups from author C. West. MBI‐EE included in analysis 1.1	
***Risk of bias***		
**Bias**	**Authors' judgement**	**Support for judgement**
Random sequence generation (selection bias)	Low risk	Quote: "We used a computer‐generated dynamic allocation algorithm to randomize participants into a coaching group and a control group. Randomization"
Allocation concealment (selection bias)	Unclear risk	Quote: "We used a computer‐generated dynamic allocation algorithm to randomize participants into a coaching group and a control group. Randomization was stratified by years in practice, work site (Arizona, Florida, Minnesota, or Mayo Clinic Health System), and primary care (family medicine, general pediatrics, or general internal medicine) vs sub‐specialty practice." Insufficient information to understand whether intervention allocations could have been foreseen in advance of, during, enrolment.
Blinding of participants and personnel (performance bias) All outcomes	High risk	Participants were not blinded
Blinding of outcome assessment (detection bias) All outcomes	High risk	Participants were not blinded whereas outcomes are self‐reported.
Incomplete outcome data (attrition bias) All outcomes	Low risk	82 of the 88 completed follow‐up >93%
Selective reporting (reporting bias)	Low risk	The authors report a trial register. For the outcomes that we are interested in, there is no selective outcome reporting.
Other bias	Unclear risk	Low participation rate (88 of the 764 eligible physicians participated)

**Table:** ElKhamali 2018

***Study characteristics***		
Methods	**Study design:** randomised controlled trial **Study grouping:** parallel group	
Participants	**Baseline characteristics** Multimodel intervention *Age n(%) < 30, 31 to 40, > 41*: 49 (49%), 45 (45%), 7 (7%) *Sex (N (% female))*: 61 (60%) *Sample size*: 101 *Years of experience (mean ± SD)*: NR Control (no intervention) *Age n(%) < 30, 31 to 40, > 41*: 46 (47%), 43 (44%), 8 (8%) *Sex (N (% female))*: 54 (56%) *Sample size* : 97 *Years of experience (mean ± SD)*: NR Overall *Age n(%) < 30, 31 to 40, > 41*: NR *Sex (N (% female))*: NR *Sample size*: NR *Years of experience (mean ± SD)*: NR **Included criteria:** (1) actively working in an adult ICU, (2) held a registered nurse license, and (3) had at least 6 months’ work experience in the current ICU. **Excluded criteria:** (1) current placement outside ICU, (2) on maternity or sick leave, (3) planning to leave ICU, or (4) already completed the simulation intervention prior to the beginning of the trial. **Pretreatment:** the only major between‐group difference was in marital status (46% were single in the intervention group vs 62% in the control group). **Compliance rate:** 100% **Response rate:** 100% **Type of healthcare worker:** nurses	
Interventions	**Intervention characteristics** Multi‐model intervention *Type of the intervention*: Intervention type 4. Combination of intervention type 1 to focus one’s attention on the experience of stress and 3 to focus on work‐related risk factors on an individual level. *Description of the intervention*: The intervention was intended to reduce job strain prevalence by improving the ability of ICU nurses to cope with stressful situations and cope with some stressors related to work organisation or working conditions. *The number of sessions*: five *Duration of each session on average*: five whole working days *Duration of the entire intervention*: two weeks *Duration of the entire intervention short vs long*: short *Intervention deliverer*: physicians and qualified nurses *Intervention form*: Group Control (no intervention) *Type of the intervention*: NA *Description of the intervention*: NA *The number of sessions*: NA *Duration of each session on average*: NA *Duration of the entire intervention*: NA *Duration of the entire intervention short vs long*: NA *Intervention deliverer*: NA *Intervention form*: NA	
Outcomes	*Job stress questionnaire (JSQ) ‐ psychological demand* **Outcome type**: ContinuousOutcome *Job stress questionnaire (JSQ) ‐ decision latitude* **Outcome type**: Continuous Outcome *Job stress questionnaire (JSQ) ‐ Social support* **Outcome type**: Continuous Outcome *The Copenhagen Psychosocial Questionnaire ‐ (COPSOQ) ‐ stress* **Outcome type**: Continuous Outcome *The Copenhagen Psychosocial Questionnaire ‐ (COPSOQ) ‐ burnout* **Outcome type**: Continuous Outcome	
Identification	**Sponsorship source:** NR **Country:** France **Setting:** Hospital **Comments:** NR **Authors name:** Radia El Khamali **Institution:** Assistance Publique‐Hôpitaux de Marseille, Hôpital Nord, Réanimation des Détresses Respiratoires et des Infections Sévères, Marseille, Franc **Email:** laurent.papazian@ap‐hm.fr **Address:** Laurent Papazian, MD, PhD, Médecine Intensive‐Réanimation, Hôpital Nord, Chemin des Bourrely, 13015 Marseille, France **Time period:** 2016‐2019	
Notes	COPSOQ stress included in analysis 4.2	
***Risk of bias***		
**Bias**	**Authors' judgement**	**Support for judgement**
Random sequence generation (selection bias)	Low risk	Briefly, ICU nurses meeting the inclusion criteria were selected by lots drawn by the clinical research unit at the Assistance Publique‐Hôpitaux de Marseille, which was not involved with the ICU. At each planned session, the chief nurse provided the clinical research unit with a list of nurses to participate in the program (each nurse chose an identification number). The clinical research unit selected 2, 4, 6, 8, 10, or 12 nurses to participate in the trial. Half of the selected nurses (1, 2, 3, 4, 5, or 6) were randomized to the intervention group and the other half were randomized to the control group.
Allocation concealment (selection bias)	Unclear risk	Participants were randomly assigned using a computer‐generated randomization list (allocation ratio of 1:1) and a permuted block design (block size range, 4‐8). Participants from 1 to 3 ICUs were randomized to 1 of 2 equal‐sized groups: (1) the 5‐day intervention simulation training group or (2) the control group (nurses did not participate in simulation training but answered questionnaires). Insufficient information to understand whether intervention allocations could have been foreseen in advance of, during, enrolment.
Blinding of participants and personnel (performance bias) All outcomes	High risk	Participants were not blinded.
Blinding of outcome assessment (detection bias) All outcomes	High risk	Participants were not blinded whereas outcomes are self‐reported.
Incomplete outcome data (attrition bias) All outcomes	Low risk	Almost no loss to follow‐up, those lost reasons were reported
Selective reporting (reporting bias)	High risk	Registration protocol: https://clinicaltrials.gov/ct2/show/NCT02672072 Did not report on the outcomes of Maslach Burnout inventory
Other bias	Low risk	No indication for other sources of bias.

**Table:** Emani 2020

***Study characteristics***		
Methods	**Study design:** randomised controlled trial **Study grouping:** parallel group	
Participants	**Baseline characteristics** Chromotherapy‐based intervention *Age (mean ± SD)*: NR *Sex (N (% female))*: 40 (100%) *Sample size*: 40 *Years of experience < 15 years and > 15 years (mean ± SD)*: < 25 (nr). > 15 (nr) Control (no intervention) *Age (mean ± SD)*: NR *Sex (N (% female))*: 40 (100%) *Sample size*: 40 *Years of experience < 15 years and > 15 years (mean ± SD)*: < 24 (nr). > 16 (nr) Overall *Age (mean ± SD)*: NR *Sex (N (% female))*: 80 *Sample size*: 80 *Years of experience < 15 years and > 15 years (mean ± SD)*: NR **Included criteria:** inclusion criteria: interested in attending chromotherapy educational and consultation sessions at least three times per month for a period of 3 months, age range between 25 and 45 years **Excluded criteria:** exclusion criteria included existing comorbid clinical conditions that could have any effect on fatigue such as depression, thyroid disease or severe infection, surgery operation from three months ago, or participants who were suffering from malnutrition and iron deficiency anaemia, and participants who were unable for other reasons to continue their participation in this research. **Pretreatment:** there were no significant differences between the two groups for age, years of experience, or years working **Compliance rate:** of 96 nurses 80 participated in all sessions > 83% **Response rate:** NR **Type of healthcare worker:** nurses	
Interventions	**Intervention characteristics** Chromotherapy‐based intervention *Type of the intervention*: Intervention type 2 ‐ to focus one’s attention away from the experience of stress *Description of the intervention* : The interventions for experimental ICU included three parts; (A) changing colour of decoration in experimental ICU, such as installation of colour panels in ICU ward, (B) providing a mobile cover and a pencil case in yellow, orange, and green colours based on the principles of chromotherapy, for each of experimental group nurses, and (C) in addition, three educational sessions on chromotherapy were held and then participants received individualised consulting sessions weekly on chromotherapy applying in their personal life and in their own home for 3 months. *The number of sessions*: 3 times per month *Duration of each session on average*: NR *Duration of the entire intervention*: 3 months *Duration of the entire intervention short vs long*: short *Intervention deliverer*: NR *Intervention form*: Group and individual Control (no intervention) *Type of the intervention*: NA *Description of the intervention*: NA *The number of sessions*: NA *Duration of each session on average*: NA *Duration of the entire intervention*: NA *Duration of the entire intervention short vs long*: NA *Intervention deliverer*: NA *Intervention form*: NA	
Outcomes	*Professional Quality of Life (ProQol) ‐ Compassion satisfaction* **Outcome type**: ContinuousOutcome *Profesional Quality of Life (ProQol) ‐ Burn out* **Outcome type**: ContinuousOutcome *Professional Quality of Life (ProQOL) ‐ Secondary traumatic stress* **Outcome type**: ContinuousOutcome	
Identification	**Sponsorship source:** NR **Country:** Iran **Setting:** Hospital **Comments:** NR **Authors name:** Roghiye Emani **Institution:** Nursing and Midwifery Faculty of Urmia University of Medical Sciences, Urmia, Iran; **Email:** haleh_ghavami@yahoo.co.uk **Address:** Nursing and Midwifery Faculty of Urmia University of Medical Sciences, Pardis–e‐Nazlou, 11 km of Nazlou Road, Urmia, Iran **Time period:** NR	
Notes	PRO‐QOl_BO included in analysis 2.1	
***Risk of bias***		
**Bias**	**Authors' judgement**	**Support for judgement**
Random sequence generation (selection bias)	Low risk	Quote: "A total of 96 ICU nurses assessed for eligibility; among them 80 nurses (according to inclusion and exclusion criteria of the study) were randomized to the experimental group or to the control group." "For randomization in this study, an independent researcher‐made random allocation cards using computer‐generated the random numbers. The allocator kept the original random allocation sequences in an inaccessible third place and worked with a copy. "
Allocation concealment (selection bias)	Low risk	Quote: "For randomization in this study, an independent researcher‐made random allocation cards using computer‐generated the random numbers. The allocator kept the original random allocation sequences in an inaccessible third place and worked with a copy."
Blinding of participants and personnel (performance bias) All outcomes	High risk	Participants were not blinded.
Blinding of outcome assessment (detection bias) All outcomes	High risk	Participants were not blinded whereas outcomes are self‐reported.
Incomplete outcome data (attrition bias) All outcomes	Unclear risk	Lost to follow‐up not reported.
Selective reporting (reporting bias)	Low risk	IRCT registration number: IRCT2017101431588N3. No indication of selective reporting.
Other bias	Unclear risk	Response rate not able to assess. Compliance not reported.

**Table:** Errazuriz 2022

***Study characteristics***		
Methods	**Study design:** randomised controlled trial **Study grouping:** parallel group	
Participants	**Baseline characteristics** Mindfulness‐based stress reduction (MSBR) *Age (mean ± SD)*: 40.9 ± 12 *Sex (N (% female))*: 34 (97%) *Sample size*: 35 *Years of experience (mean ± SD)*: 16.8 ± 11 Psychoeducational stress management (SMC) *Age (mean ± SD)*: 40.1 ± 14 *Sex (N (% female))*: 33 (97%) *Sample size*: 34 *Years of experience (mean ± SD)*: nr ± (11) Wait list *Age (mean ± SD)*: 39.6 ± 11 *Sex (N (% female))*: 36 (100%) *Sample size*: 36 *Years of experience (mean ± SD)*: 14 ± 10 Overall *Age (mean ± SD)*: NR *Sex (N (% female))*: NR *Sample size*: NR *Years of experience (mean ± SD)*: NR **Included criteria:** (i) non‐physician healthcare workers; (ii) aged ≥ 18 years; (iii) with a permanent work contract; and (iv) in direct contact with patients. **Excluded criteria:** participants were excluded if they reported suicidal ideation or problematic alcohol consumption at enrolment as measured in items 8 and 11 of the 45‐item Outcome Questionnaire **Pretreatment:** the three groups did not differ significantly in any of the collected baseline characteristics, except for levels of ’rewards’ at work and scores in the mindfulness ‘describing’ facet **Compliance rate:** NR **Response rate:** NR **Type of healthcare worker:** various healthcare workers	
Interventions	**Intervention characteristics** Mindfulness‐based stress reduction (MSBR) *Type of the intervention*: Intervention type 2 ‐ to focus one’s attention away from the experience of stress *Description of the intervention*: Mindfulness‐based stress reduction (MBSR) courses teach individuals to observe thoughts, emotions, and situations non‐judgementally and non‐reactively via exercises, including meditation *The number of sessions*: 8 *Duration of each session on average*: 2 hours *Duration of the entire intervention*: 8 weeks *Duration of the entire intervention short vs long*: short *Intervention deliverer*: Clinical psychologist *Intervention form*: Group Psychoeducational stress management (SMC) *Type of the intervention*: Intervention type 1 ‐ to focus one’s attention on the experience of stress *Description of the intervention*: The training was aimed to develop efficient coping strategies through lectures addressing work‐related stress and wellbeing, interpersonal support, and experiential activities. *The number of sessions*: 8 *Duration of each session on average*: 2 hours *Duration of the entire intervention*: 8 weeks *Duration of the entire intervention short vs long*: short *Intervention deliverer*: Clinical psychologist *Intervention form*: Group Wait list *Type of the intervention*: NA *Description of the intervention*: NA *The number of sessions*: NA *Duration of each session on average*: NA *Duration of the entire intervention*: NA *Duration of the entire intervention short vs long*: NA *Intervention deliverer*: NA *Intervention form*: NA	
Outcomes	*General Health Questionnaire (GHQ‐12)* **Outcome type**: ContinuousOutcome *45‐item Outcome Questionnaire (OQ‐45)* **Outcome type**: ContinuousOutcome *Perceived Stress Scale (PSS)* **Outcome type**: ContinuousOutcome	
Identification	**Sponsorship source:** NR **Country:** Chile **Setting:** Mixed healthcare settings including: a tertiary hospital, a teaching hospital and an outpatient complex. **Comments:** NR **Authors name:** Antonia Errazuriz **Institution:** Department of Psychiatry, School of Medicine, Pontificia Universidad Catolica de Chile **Email:** anerrazuriz@uc.cl **Address:** School of Medicine, Pontificia Universidad Catolica de Chile, Diagonal Paraguay 362, Santiago, 8330077, Chile **Time period:** NR	
Notes	We kindly received the mean and SD of the primary outcome from author A. Errazuriz. PSS included in analysis 1.1 and 1.2 and 2.1 and 2.2 and 5.1 and 5.2 and 6.1 and 6.2	
***Risk of bias***		
**Bias**	**Authors' judgement**	**Support for judgement**
Random sequence generation (selection bias)	Low risk	Quote: "Participants were randomized into three groups (1:1:1 ratio) using computer‐generated random numbers, stratified by work position."
Allocation concealment (selection bias)	Unclear risk	Quote: "Allocation was executed by ordering subjects according to the random number within strata and assigning the subjects within each stratum to groups 1, 2, and 3, consecutively, until exhausting the number of subjects within each stratum." Insufficient information to understand whether intervention allocations could have been foreseen in advance of, during, enrolment.
Blinding of participants and personnel (performance bias) All outcomes	High risk	Participants were not blinded.
Blinding of outcome assessment (detection bias) All outcomes	High risk	Participants were not blinded whereas outcomes are self‐reported.
Incomplete outcome data (attrition bias) All outcomes	High risk	52 of the 105 (50%) randomised participants were included in the analysis. Reasons not provided. Not reported whether lost to follow‐up at random.
Selective reporting (reporting bias)	High risk	Trial registration: ISRCTN12039804. Did not report on Maslach Burnout Inventory and number of sick leaves in the previous three months
Other bias	Unclear risk	Compliance rate and response rate not reported.

**Table:** Ewers 2002

***Study characteristics***		
Methods	RCT, UK	
Participants	20 forensic mental health nurses	
Interventions	1) Experimental: psychosocial Intervention Training: 20 days of training with the aim to improve nurses' knowledge about serious mental illness and attitude towards patients and thus decrease subjective burnout. Training duration 6 months. The training helps clinicians to conceptualise their patients' problems within a more empathic framework and trains them in the skills to intervene effectively. Thus, self‐efficacy may increase and jobs may be perceived as more rewarding. 2) Control: no intervention	
Outcomes	MBI directly after training	
Identification		
Notes	MBI‐EE included in analysis 3.1	
***Risk of bias***		
**Bias**	**Authors' judgement**	**Support for judgement**
Random sequence generation (selection bias)	Unclear risk	"The 20 staff who volunteered for the PSI training were randomly allocated to either the experimental PSI training group (n = 10) or a waiting list control group (n=10). The sample was stratified by ward, sex and day/night duty, thus subjects in each group represented all grades of staff and all wards." (p. 473)
Allocation concealment (selection bias)	Unclear risk	Not reported.
Blinding of participants and personnel (performance bias) All outcomes	High risk	Participants were not blinded.
Blinding of outcome assessment (detection bias) All outcomes	High risk	Participants were not blinded whereas outcomes are self‐reported.
Incomplete outcome data (attrition bias) All outcomes	Unclear risk	Presumably all participants completed all measurements as no data reported on dropouts.
Selective reporting (reporting bias)	Low risk	All outcomes reported.
Other bias	Unclear risk	We did not find any indications of other sources of bias.

**Table:** Fendel 2021

***Study characteristics***		
Methods	**Study design:** randomised controlled trial **Study grouping:** parallel group	
Participants	**Baseline characteristics** Mindfulness based program (MBP) *Age (mean ± SD)*: 31 ± 3.4 *Sex (N (% female))*: 49 (64%) *Sample size*: 76 *Years of experience (mean ± SD)*: 3.2 ± 1.7 Control (no intervention) *Age (mean ± SD)*: 31 ± 3.5 *Sex (N (% female))*: 47 (66%) *Sample size*: 71 *Years of experience (mean ± SD)*: 2.8 ± 1.6 Overall *Age (mean ± SD)*: 31 ± 3.4 *Sex (N (% female))*: 96 (65%) *Sample size*: 147 *Years of experience (mean ± SD)*: 3 ± 1.7 **Included criteria:** Physicians younger than 45, with an ongoing position as a resident physician at base‐line, and minimum employment of 40% **Excluded criteria:** NR **Pretreatment:** at baseline, there were no statistically significant differences between groups with regard to demo‐graphics, meditation experience, distress and quality of care outcomes except for a difference in how attentive the resident physicians were, as judged by their colleagues **Compliance rate:** 198 eligible 147 participated in at least 50% of the intervention > 74% **Response rate:** 150 of the 181 eligible physicians participated (83%) **Type of healthcare worker:** resident physician	
Interventions	**Intervention characteristics** Mindfulness‐based program (MBP) *Type of the intervention*: Intervention type 1 ‐ to focus one’s attention on the experience of stress *Description of the intervention*: moment‐to‐moment awareness, cultivated by paying attention to the present moment, as non‐judgementally and open‐heartedly as possible We based the program on the validated MBSR program [45] and tailored it to resident physicians’ particular needs and circumstances. The tailoring process, program content and feasibility findings have been described elsewhere [40, 42]. Importantly, as proposed in the literature, we introduced mindfulness as a practise of self‐care, in order to promote personal well‐being, meaning and professional fulfilment rather than as a means to foster stress resistance *The number of sessions*: eight *Duration of each session on average*: 135 min *Duration of the entire intervention*: four months *Duration of the entire intervention short vs long*: long *Intervention deliverer*: three psychiatrist *Intervention form*: Group Control (no intervention) *Type of the intervention*: NA *Description of the intervention*: NA *The number of sessions*: NA *Duration of each session on average*: NA *Duration of the entire intervention*: NA *Duration of the entire intervention short vs long*: NA *Intervention deliverer*: NA *Intervention form*: NA	
Outcomes	*Copenhagen Burnout Inventory (CBI)‐ burnout* **Outcome type**: Continuous Outcome *Perceived Stress Scale (PSS)* **Outcome type**: Continuous Outcome *General Health Questionnaire‐12 (GHQ‐12) Mental distress* **Outcome type**: Continuous Outcome *Patient Health Questionnaire (PHQ4) ‐ depression* **Outcome type**: Continuous Outcome *Patient Health Questionnaire (PHQ4) ‐ anxiety* **Outcome type**: Continuous Outcome	
Identification	**Sponsorship source:** NR **Country:** Germany **Setting:** University of Freiburg **Comments:** NR **Authors name:** Johannes Fendel **Institution:** Department of Occupational and Consumer Psychology, Institute of Psychology, University of Freiburg, Freiburg, Germany **Email:** NR **Address:** Department of Occupational and Consumer Psychology, Institute of Psychology, University of Freiburg, Freiburg, Germany **Time period:** 2018‐2020	
Notes	PSS included in analysis 1.1 and 1.2 PHQ included in analysis 1.4 and 1.5	
***Risk of bias***		
**Bias**	**Authors' judgement**	**Support for judgement**
Random sequence generation (selection bias)	Low risk	Quote: "email from the study team. We used minimisation to allocate participants into one of two groups using the software Qminim [64]. Through this approach, we minimised the imbalance between the groups with regard to gender (male, female) and baseline levels of personal burnout (CBI cut‐off values 0– 37.4 = low, 37.5–62.4 = medium, 62.5–100 = high burnout) [65]. We applied a weighted random".
Allocation concealment (selection bias)	Low risk	Quote: "A researcher with no contact with participants carried out minimisation and group assignment after the completion of baseline assessments."
Blinding of participants and personnel (performance bias) All outcomes	High risk	Quote: "Due to the nature of the interventions, participants and trainers were aware of the allocated arm. To minimise bias, self‐report measures were administered online."
Blinding of outcome assessment (detection bias) All outcomes	High risk	Participants were not blinded whereas outcomes are self‐reported.
Incomplete outcome data (attrition bias) All outcomes	Low risk	A low drop‐out rate of 18% at follow‐up for the primary outcome. 98% included in the intention to treat analysis.
Selective reporting (reporting bias)	Low risk	Trial registration DRKS00014015. No indication of selective reporting on stress/burnout assessment.
Other bias	Low risk	No indication of other sources of bias

**Table:** Finnema 2005

***Study characteristics***		
Methods	RCT, the Netherlands	
Participants	99 nursing assistants	
Interventions	1) Experimental: Integrated emotion‐oriented care: Basic training course of two days with an intermediary period of two weeks for homework (for all staff members on intervention wards) addressing staff members' own experience, phases of ego‐experience of the demented residents and the application of (non‐)verbal empathic skills. Advanced course of seven days spread out over seven to eight months for five people from each intervention ward and an Adviser course of 10 days over nine months for one person from each intervention ward. 2) Control: Training and support in giving usual care	
Outcomes	The Organization and Stress Scale	
Identification		
Notes	OSS included in analysis 3.1	
***Risk of bias***		
**Bias**	**Authors' judgement**	**Support for judgement**
Random sequence generation (selection bias)	Unclear risk	"A pretest‐posttest control group design with matched groups (randomized clinical trial) was used" (p. 331)
Allocation concealment (selection bias)	Unclear risk	Not reported.
Blinding of participants and personnel (performance bias) All outcomes	High risk	Participants were not blinded
Blinding of outcome assessment (detection bias) All outcomes	High risk	Participants were not blinded whereas outcomes are self‐reported.
Incomplete outcome data (attrition bias) All outcomes	High risk	"During the experimental period 25 nursing assistants dropped out due to: illness (11), pregnancy (2), and transfer (9). In three cases questionnaires were missing. Data analysis was carried out on 99 'complete' cases. Drop‐out did not differ between the groups..." (p. 333)
Selective reporting (reporting bias)	High risk	For nursing assistants results consist of covariance analyses that were not prespecified.
Other bias	Low risk	We did not identify any indications of other sources of bias.

**Table:** Fiol DeRoque 2021

***Study characteristics***		
Methods	**Study design:** randomised controlled trial **Study grouping:** parallel group	
Participants	**Baseline characteristics** Mindfulness‐based mHealth intervention (PsyCovidApp Group) *Age (mean ± SD)*: 42 ± 11 *Sex (N (% female))*: 210 (85%) *Sample size*: 248 *Years of experience (mean ± SD)*: NR Control (App) group *Age (mean ± SD)*: 40 ± 9.6 *Sex (N (% female))*: 191 (82%) *Sample size*: 234 *Years of experience (mean ± SD)*: NR Overall *Age (mean ± SD)*: 41.4 ± 10.4 *Sex (N (% female))*: 401 (83%) *Sample size*: 482 *Years of experience (mean ± SD)*: NR **Included criteria:** healthcare workers from any medical speciality (pneumology, internal medicine, emergency, primary care, etc) and role (physicians, nurses, nurse assistants, etc) with access to a smartphone. We included health care workers who had provided direct, face‐to‐face health care to patients with a diagnosis of infection with COVID‐19. **Excluded criteria:** healthcare workers who were not able to download and activate the app used to deliver the intervention during the next 10 days following the baseline assessment. **Pretreatment:** reported socio demographic baseline characteristics of participants randomised to the intervention group were similar to socio demographic baseline characteristics of participants randomised to the control group **Compliance rate:** 684 healthcare workers of which 482 participated in at least 50% of the intervention 482 > 74% **Response rate:** 482 of the 525 (92%) eligible participants participated **Type of healthcare worker:** various HCWs	
Interventions	**Intervention characteristics** Mindfulness‐based mHealth intervention (PsyCovidApp Group) *Type of the intervention*: Intervention type 1 ‐ to focus one’s attention on the experience of stress *Description of the intervention*: The self‐managed psycho‐educational intervention, based on cognitive‐behavioural therapy and mindfulness approaches, included written and audiovisual content targeting four areas: emotional skills, healthy lifestyle behaviour, work stress and burnout, and social support. Additionally, the intervention included daily prompts (notifications) that included brief questionnaires to monitor mental health status, followed by short messages offering tailored information and resources based on the participants ´responses. *The number of sessions*: NR *Duration of each session on average*: NR *Duration of the entire intervention*: two weeks *Duration of the entire intervention short vs long*: short *Intervention deliverer*: An App developed by group of psychologists, psychiatrists and experts in healthy lifestyle promotion (all co‐authors of the study) *Intervention form*: Individual Control (App) group *Type of the intervention*: Control App *Description of the intervention*: Brief written information about the mental health care of health care workers during the COVID‐19 pandemic *The number of sessions*: NA *Duration of each session on average*: NA *Duration of the entire intervention*: two weeks *Duration of the entire intervention short vs long*: short *Intervention deliverer*: An App developed by group of psychologists, psychiatrists and experts in healthy lifestyle promotion (all co‐authors of the study) *Intervention form*: Individual	
Outcomes	*DASS ‐ overall* **Outcome type**: Continuous Outcome *Maslach Burnout Inventory ‐ Emotional exhaustion* **Outcome type**: Continuous Outcome *Maslach Burnout Inventory ‐ Personal accomplishment* **Outcome type**: Continuous Outcome *Maslach Burnout Inventory ‐ Depersonalisation* **Outcome type**: Continuous Outcome	
Identification	**Sponsorship source:** NR **Country:** Spain **Setting:** Hospital **Comments:** NR **Authors name:** Maria Antònia Fiol‐DeRoque **Institution:** Health Research Institute of the Balearic Islands, Palma de Mallorca, Spain **Email:** mariajesus.serranoripoll@ssib.es **Address:** Maria Jesús Serrano‐Ripoll, PhD Health Research Institute of the Balearic Islands Edificio S, Hospital Universitario Son Espases Carretera de Valldemossa Palma de Mallorca, 07120 Spain **Time period:** 2020	
Notes	MBI‐EE included in analysis 1.1	
***Risk of bias***		
**Bias**	**Authors' judgement**	**Support for judgement**
Random sequence generation (selection bias)	Low risk	Quote: "Participants were randomly assigned (1:1) to receive the PsyCovidApp intervention or the control app over two weeks by a designated researcher" Using a computer‐generated sequence of random numbers create by Internet relay chat.
Allocation concealment (selection bias)	Low risk	Randomization done by a designated researcher who was not involved in data collection or analysis.
Blinding of participants and personnel (performance bias) All outcomes	Low risk	Health care workers were blinded to group allocation (as both groups received an app)
Blinding of outcome assessment (detection bias) All outcomes	Low risk	Quote: "Participants, the outcome data collectors and trial statisticians were unaware of the treatment allocation."
Incomplete outcome data (attrition bias) All outcomes	Low risk	Loss to follow‐up less than 20%. 436 of the 482 randomized participants had complete two‐week outcome data.
Selective reporting (reporting bias)	Low risk	https://clinicaltrials.gov/ct2/show/NCT04393818 No differences between study and registration of the protocol
Other bias	Low risk	No indication of other source of bias

**Table:** Foji 2020

***Study characteristics***		
Methods	**Study design:** randomised controlled trial **Study grouping:** parallel group	
Participants	**Baseline characteristics** Emotional intelligence training *Age (mean ± SD)*: NR *Sex (N (% female))*: 52 (84%) *Sample size*: 62 *Years of experience (mean ± SD)*: NR Control (no intervention) *Age (mean ± SD)*: NR *Sex (N (% female))*: 60 (82%) *Sample size*: 73 *Years of experience (mean ± SD)*: NR Overall *Age (mean ± SD)*: NR *Sex (N (% female))*: 112 (83%) *Sample size*: 135 *Years of experience (mean ± SD)*: NR **Included criteria:** NR **Excluded criteria:** people who have not already received any training on the topic of research. If a person has already been trained, the results of the study will be affected whether the score obtained from the study is present or not? Do not use anti‐anxiety and tranquilizers during the study period. Drug use could interfere with the outcome (due to sleepiness and lack of consciousness) (either at the training stage or the completion stage of the response). No night shift before the night before the tests. Fatigue caused by night shift could interfere with completing the questionnaire or understanding the training sessions. [4] To commit to attend all or more than half of the meetings. Pregnant nurses were excluded or nurses with underlying diseases (blood pressure, diabetes, etc.) were excluded. **Pretreatment:** NR **Compliance rate:** NR **Response rate:** NR **Type of healthcare worker:** exclusively nurses	
Interventions	**Intervention characteristics** Workplace Health Promotion Program *Type of the intervention*: Intervention type 1 ‐ to focus one’s attention on the experience of stress *Description of the intervention*: The training program in this study is based on a training package of emotional intelligence,[9] implemented during 6 sessions of 2 h, 2 days a week. The related experts did training in two repetitive periods (two 6‑session courses). The content of the program for each session was as follows: group and members’ referrals with each other, familiarity with the method of work, learning and discussing emotional intelligence and its components, understanding the concept of emotional self‑regulation, expressing emotions, attachments and ways of changing perceptions. *The number of sessions*: 6 *Duration of each session on average*: 2 hours *Duration of the entire intervention*: 3 weeks *Duration of the entire intervention short vs long*: Short *Intervention deliverer*: Experts *Intervention form*: Group, face‐to‐face Control (no intervention) *Type of the intervention*: NA *Description of the intervention*: NA *The number of sessions*: NA *Duration of each session on average*: NA *Duration of the entire intervention*: NA *Duration of the entire intervention short vs long*: NA *Intervention deliverer*: NA *Intervention form*: NA	
Outcomes	*General Health Questionnaire (GHQ)* **Outcome type**: ContinuousOutcome	
Identification	**Sponsorship source:** NR **Country:** Iran **Setting:** Hospital **Comments:** NR **Authors name:** Dr. Razieh Khosrorad **Institution:** Department of Health Education, School of Health, Sabzevar University of Medical Sciences, Sabzevar, Iran. Educational Neuroscience Research Center, Sabzevar University of Medical Sciences, Sabzevar, Iran. **Email:** rkhosrorad@yahoo. com **Address:** NR **Time period:** NR	
Notes	GHQ included in analysis 1.1	
***Risk of bias***		
**Bias**	**Authors' judgement**	**Support for judgement**
Random sequence generation (selection bias)	Low risk	Target population of this study consist of nurses in units of Mohammad Vasei, Shahid Beheshti, and Shahidan Mobini Hospitals in Sabzevar, randomly divided into two groups and a sample of 135 people were randomly selected based on the list of sample group names and random number table
Allocation concealment (selection bias)	Unclear risk	Not reported.
Blinding of participants and personnel (performance bias) All outcomes	High risk	Participants were not blinded.
Blinding of outcome assessment (detection bias) All outcomes	High risk	Participants were not blinded whereas outcomes are self‐reported.
Incomplete outcome data (attrition bias) All outcomes	Unclear risk	Not reported
Selective reporting (reporting bias)	Unclear risk	Inaccessible plan number 94098 with the ethics code of IR.MEDSAB.REC.1394.51. No indication of selective reporting.
Other bias	Unclear risk	Response rate and compliance rate not reported.

**Table:** Frogeli 2020

***Study characteristics***		
Methods	**Study design:** randomised controlled trial **Study grouping:** parallel group	
Participants	**Baseline characteristics** Engagement in proactive behaviours *Age (mean (min‐max)*: 27. 8 (22‐54) *Sex (N % female)*: 106 (82%) *Sample size*: 130 *Years of experience (mean ± SD)*: NR Control (care as usual) *Age (mean (min‐max)*: 27. 2 (21–52) *Sex (N % female)*: 97 (89%) *Sample size*: 109 *Years of experience (mean ± SD)*: NR Overall *Age (mean (min‐max)*: NR *Sex (N % female)*: 203 (85%) *Sample size*: 239 *Years of experience (mean ± SD)*: NR **Included criteria:** eligible participants were newly graduated nurses who worked at any clinical department of Uppsala University Hospital and participated in the transition‐to‐practice program **Excluded criteria:** NR **Pretreatment:** no group differences reported **Type of healthcare worker:** exclusively nurses **Response rate:** 86% **Compliance rate:** 95%	
Interventions	**Intervention characteristics** Engagement in proactive behaviours *Description of the intervention*: Session 1: The model of the intervention Presentation/discussion about the newcomer experience, stress, and stress‐related ill health Presentation/discussion of the socialisation processes role clarity, task mastery, and social acceptance and their association with experiences of stress when transitioning into a new profession. Discussion about obstacles to engaging in proactive behaviours, with a focus on emotional experiences and fatigue Increase engagement in leisure activities. Discussion about strategies to recover energy with a focus on sleep, physical exercise, social interactions, and personal interests. Homework assignment. Individual exercise with the goal of increasing engagement in one specific recovery‐promoting leisure activity per week based on the principles of approach behaviours and action planning. Session 2: Follow‐up on session 1. Repetition of session 1. Discussion of experiences of trying to increase engagement in leisure activities (homework assignment from session 1). Reduce engagement in avoidance behaviours. Discussion of common fears experienced as a newcomer and the effect of fears on behaviours, with a focus on avoidance of proactive behaviours and effects on the socialisation processes and management of challenges. Homework assignment Individual exercise with the goal of increasing engagement in one specific recovery‐promoting leisure activity per week based on the principles of approach behaviours and action planning Individual exercise with the goal of reducing avoidance behaviours/increasing engagement in proactive behaviours based on principles of systematic exposure and action planning. Session 3: Follow‐up on sessions 1 and 2. Repetition of Session 1 and 2. Discussion of experiences of trying to increase engagement in leisure activities as well as decrease avoidance behaviours/increase engagement in proactive behaviours (homework assignment from Session 2). Summary of intervention Key take‐home messages. *The number of sessions*: 3 *Duration of each session on average*: three hours *Duration of the entire intervention*: nine hours *Duration of the entire intervention short vs long*: short *Intervention deliverer*: first author, psychologist *Intervention form*: group *Type of the intervention*: Intervention type 1 ‐ to focus one’s attention on the experience of stress Control (care as usual) *Description of the intervention*: The control intervention consisted of the ordinary content of the transition‐to‐practice program. The total time of activities was the same for the control intervention as for the experimental intervention. The purpose of the control intervention was to facilitate the professional adjustment of the new RNs. The sessions focused on subjects such as patient care (e.g. nutrition, wound treatment), communication skills, team management, and the role, rights, and responsibilities of nurses. The control intervention was managed by the clinical training centre at the hospital where the study took place *The number of sessions*: three *Duration of each session on average*: three hours *Duration of the entire intervention*: nine hours *Duration of the entire intervention short vs long*: short *Intervention deliverer*: NR *Intervention form*: Group *Type of the intervention*: Intervention type 1 ‐ to focus one’s attention on the experience of stress	
Outcomes	*Items from the Stress and Energy Questionnaire* **Outcome type**: Continuous Outcome	
Identification	**Sponsorship source:** Funding: This study was supported by AFA insurance [Grant no 140007] **Country:** Sweden **Setting:** University hospital **Comments:** NR **Authors name:** Elin Frögéli **Institution:** Department of Clinical Neuroscience, Karolinska Institute **Email:** elin.frogeli@ki.se **Address:** NR **Time period:** 2016‐2017	
Notes	Included in analysis 1.1	
***Risk of bias***		
**Bias**	**Authors' judgement**	**Support for judgement**
Random sequence generation (selection bias)	Unclear risk	Quote: "The participants were randomized to one of two groups of equal size at a 1:1 ratio, stratified by clinical ward. Specifically, person 1 that was registered for the program was allocated to group 1, person 2 was allocated to group 2, person 3 to group 3, and so on. However, if person 1 and person 3 came from the same clinical ward, person 3 was placed in group 2. Person 4 was then placed in group 1 and person 5 in group 2, and so on. The purpose of this design was to avoid having too many nurses from the same clinical ward being placed in the same study group, as this would cause problems of staffing on the clinical wards." Sequence generation process not mentioned.
Allocation concealment (selection bias)	High risk	Participants and/or investigators enroling participants could possibly foresee assignments and thus introduce selection bias.
Blinding of participants and personnel (performance bias) All outcomes	High risk	Quote: "Finally, no measures were taken to assure that there was no diffusion of information between the groups, which may have affected the results." Participants were not blinded.
Blinding of outcome assessment (detection bias) All outcomes	High risk	Participants were not blinded whereas outcomes are self‐reported.
Incomplete outcome data (attrition bias) All outcomes	Unclear risk	Quote: "Attrition Analysis: We investigated differences in the study variables at baseline between participants who responded at follow‐up and those who did not. These analyses revealed no differences." Randomized 239 ‐> lost to follow‐up ‐> 55 = 23% but no differences between responders and not responders. However, these analyses have not been reported.
Selective reporting (reporting bias)	Unclear risk	No mention of a protocol, nor did we find one
Other bias	Unclear risk	Quote: "The reliability of some of the measures in the present trial was questionable, which may have limited the ability to properly assess the outcomes." ‘Loosely’ validated outcome measure

**Table:** Ghawadra 2020

***Study characteristics***		
Methods	**Study design:** randomised controlled trial **Study grouping:** parallel group	
Participants	**Baseline characteristics** Mindfulness‐based training *Age <25, 26‐30, > 31 (n(%))*: 42 (49%), 19 (47%), 42 (65%) *Sex (N (% female))*: 112 (53%) *Sample size*: 118 *Years of experience < 5, 6‐10, > 11 (n(%))*: 57 (50%), 31 (47%), 30 (70%) Control (no intervention) *Age < 25, 26‐30, > 31 (n(%))*: 44 (51%), 20 (53%), 23 (35%) *Sex (N (% female))*: 101 (47%) *Sample size*: 106 *Years of experience < 5, 6‐10, > 11 (n(%))*: 58 (50%), 35 (53%), 13 (30%) Overall *Age < 25, 26‐30, > 31 (n(%))*: NR *Sex (N (% female))*: NR *Sample size*: 224 *Years of experience < 5, 6‐10, > 11 (n(%))*: NR **Included criteria:** nurses who work in wards who had mild to moderate levels of stress and depression (according to DASS‐21) in an earlier cross‐sectional survey. **Excluded criteria:** nurses who work in the outpatient clinic, or nursing managers, due to the different types of patient care, roles and responsibilities. Nurses who have a history of mental illness (n = 3) were excluded in the first study. The nurses who had severe and extremely severe levels of SAD (according to DASS‐21). They were advised to seek professional help at the psychiatric/psychology clinic in the hospital. **Pretreatment:** reported socio‐demographic baseline characteristics of participants randomised to the intervention group were similar to socio‐demographic baseline characteristics of participants randomized to the control group **Compliance rate:** the drop‐out rate was high, especially for the website intervention (48.3%). **Response rate:** it seems that all eligible participants actually participated **Type of healthcare worker:** nurses	
Interventions	**Intervention characteristics** Mindfulness‐based training *Type of the intervention*: Intervention type 1 ‐ to focus one's attention on the experience of stress *Description of the intervention*: The MBT intervention in this study was MINDFULGym, consisted of ABC of stress, introduction to mindfulness, mindful body stretching, mindful breathing, NOW‐ing the present moment, paying attention to wellness, loving kindness practice *The number of sessions*: NR *Duration of each session on average*: 2 hours workshop, 4 weeks of self‐practice *Duration of the entire intervention*: 4 weeks *Duration of the entire intervention short vs long*: short *Intervention deliverer*: Author *Intervention form*: Individual and group Control (no intervention) *Type of the intervention*: NA *Description of the intervention*: NA *The number of sessions*: NA *Duration of each session on average*: NA *Duration of the entire intervention*: NA *Duration of the entire intervention short vs long*: NA *Intervention deliverer*: NA *Intervention form*: NA	
Outcomes	*DASS ‐ Depression* **Outcome type**: ContinuousOutcome *DASS ‐ Anxiety* **Outcome type**: ContinuousOutcome *DASS ‐ stress* **Outcome type**: ContinuousOutcome	
Identification	**Sponsorship source:** NR **Country:** Malaysia **Setting:** Hospital **Comments:** NR **Authors name:** Sajed Faisal Ghawadra **Institution:** Department of Nursing Science, Faculty of Medicine, University of Malaya, Kuala Lumpur, Malaysi **Email:** katlim@um.edu.my **Address:** Khatijah Lim Abdullah, Department of Nursing Science, Faculty of Medicine, University of Malaya, 506030 Kuala Lumpur, Malaysia and Fakultas Keperawatan Universitas Airlangga, Surabaya, Indonesia **Time period:** NR	
Notes	Not able to include in analysis due to missing data.	
***Risk of bias***		
**Bias**	**Authors' judgement**	**Support for judgement**
Random sequence generation (selection bias)	Unclear risk	Quote: "The participants were randomly assigned to the intervention and control groups using stratified blocked randomization." Sequence generation process insufficiently described.
Allocation concealment (selection bias)	Unclear risk	Unable to judge whether participants and/or investigators could possibly foresee assignment
Blinding of participants and personnel (performance bias) All outcomes	High risk	Participants were not blinded
Blinding of outcome assessment (detection bias) All outcomes	High risk	Participants were not blinded whereas outcomes are self‐reported
Incomplete outcome data (attrition bias) All outcomes	Low risk	25 of the 249 (11%) participants were not analysed. Reasons provided. Not mentioned whether lost to follow‐up was at random however loss to follow‐up is below our pre‐defined cut‐off point.
Selective reporting (reporting bias)	Unclear risk	Trial registration, nor did we find one online
Other bias	Low risk	Low compliance but the per protocol analysis did not differ from the intention to treat analysis. Per‐protocol (PP) analysis was performed for (n = 136) participants who completed all the intervention (workshop and website), and for those who completed the three‐point questionnaires; the intervention and control group in PP analysis were n = 37 and 99, respectively. The results of the PP using GEE were similar to ITT, which strengthens the validity of the results

**Table:** Gollwitzer 2018

***Study characteristics***		
Methods	**Study design:** randomised controlled trial **Study grouping:** parallel group	
Participants	**Baseline Characteristics** Mental contrasting with implementation intentions‐ MCII *Age (mean ± SD)*: NR *Sex (N (% female))*: NR *Sample size*: 41 *Years of experience (mean ± SD)*: NR Mental contrasting with implementation intentions + further intervention groups ‐ IIMCII *Age (mean ± SD)*: NR *Sex (N (% female))*: NR *Sample size*: 41 *Years of experience (mean ± SD)*: NR Control *Age (mean ± SD)*: NR *Sex (N (% female))*: NR *Sample size*: 47 *Years of experience (mean ± SD)*: NR Overall *Age (mean ± SD)*: 40 ± 10.2 *Sex (N (% female))*: 86 (82%) *Sample size*: 129 *Years of experience (mean ± SD)*: 17.6 (NR) **Included criteria:** NR **Excluded criteria:** NR **Pretreatment:** There were no significant differences between the two groups for age, years of experience, or years working **Compliance rate:** NR **Response rate:** of 251 eligible participants 129 participated > 51% **Type of healthcare worker:** various healthcare workers	
Interventions	**Intervention characteristics** Mental contrasting with implementation intentions ‐ MCII *Type of the intervention*: Intervention type 1 ‐ to focus one’s attention on the experience of stress *Description of the intervention*: Engage in a mental exercise that (1) required specifying a wish related to reducing stress, (2) identifying and imagining its most desired positive outcome, (3) detecting and imagining the obstacle that holds them back, and (4) coming up with an if‐then plan on how to overcome it. *The number of sessions*: 3 *Duration of each session on average*: NR *Duration of the entire intervention*: 3 weeks *Duration of the entire intervention short vs long*: short *Intervention deliverer*: NR *Intervention form*: individual Mental contrasting with implementation intentions + further intervention groups ‐ IIMCII *Type of the intervention*: Intervention type 1 ‐ to focus one’s attention on the experience of stress *Description of the intervention*: Engage in a mental exercise that (1) required specifying a wish related to reducing stress, (2) identifying and imagining its most desired positive outcome, (3) detecting and imagining the obstacle that holds them back, and (4) coming up with an if‐then plan on how to overcome it. (5) plan where and when to perform MCII. *The number of sessions*: 3 *Duration of each session on average*: NR *Duration of the entire intervention*: 3 weeks *Duration of the entire intervention short vs long*: short *Intervention deliverer*: NR *Intervention form*: individual Control (no intervention) *Type of the intervention*: NA *Description of the intervention*: NA *The number of sessions*: NA *Duration of each session on average*: NA *Duration of the entire intervention*: NA *Duration of the entire intervention short vs long*: NA *Intervention deliverer*: NA *Intervention form*: NA	
Outcomes	*Perceived Stress Questionnaire‐ 20‐ PSQ‐20* **Outcome type**: ContinuousOutcome	
Identification	**Sponsorship source:** NR **Country:** Germany **Setting:** Various health institutions **Comments:** NR **Authors name:** Peter M Gollwitzer **Institution:** Department of Psychology, New York University, New York, NY, United States **Email:** gabriele.oettingen@nyu.edu **Address:** NR **Time period:** NR	
Notes	PSQ included in analysis 1.1. Intervention groups combined to create a single pair‐wise comparison	
***Risk of bias***		
**Bias**	**Authors' judgement**	**Support for judgement**
Random sequence generation (selection bias)	Low risk	Quote: "The message also contained the email address of the experimenter whom the nurses should contact if they wanted to register for the study. Those who registered (N = 251 nurses) were contacted in return by the experimenter (again via email) and given access to the study website that had been created by using the soscisurvey.de data collection service. Participants who entered the website (N = 129) were randomly assigned to the three conditions of the study (MCII = 41, and IIMCII = 41, Control = 47)"
Allocation concealment (selection bias)	Unclear risk	Not mentioned.
Blinding of participants and personnel (performance bias) All outcomes	High risk	Participants were not blinded.
Blinding of outcome assessment (detection bias) All outcomes	High risk	Participants were not blinded whereas outcomes are self‐reported.
Incomplete outcome data (attrition bias) All outcomes	Low risk	105 of the 129 (81%) randomized participants answered the final questionnaire. Reasons not provided nor whether missing was at random.
Selective reporting (reporting bias)	Unclear risk	No registration, nor did we find one.
Other bias	Unclear risk	Data on participants' adherence to the MCII instructions and the frequency and context of participants using MCII is not described.

**Table:** Grabbe 2020

***Study characteristics***		
Methods	**Study design:** randomised controlled trial **Study grouping:** parallel group	
Participants	**Baseline characteristics** Community Resiliency Model *Age (mean ± SD)*: 45.3 ± 13 *Sex (N (% female))*: 99 (100%) *Sample size*: 99 *Years of experience (mean ± SD)*: 16 ± 14 Control (Nutrition) *Age (mean ± SD)*: 45.9 ± 13 *Sex (N (% female))*: 97 (100%) *Sample size*: 97 *Years of experience (mean ± SD)*: 19.2 ± 13 Overall *Age (mean ± SD)*: 45.3 ± 13 *Sex (N (% female))*: 196 (100%) *Sample size*: 196 *Years of experience (mean ± SD)*: 17.7 ± 13 **Included criteria:** NR **Excluded criteria:** NR **Pretreatment:** no significant differences were noted between the two randomised groups by age, years in nursing, or on any of the base‐line measures. **Compliance rate:** 59 of the 99 (60%) participants allocated to the intervention group did not receive the intervention **Response rate:** of the 1600 invited nurses, 196 completed baseline and were randomised (12%) **Type of healthcare worker:** nurses	
Interventions	**Intervention characteristics** Community Resiliency Model *Type of the intervention*: Intervention type 1 ‐ to focus one’s attention on the experience of stress *Description of the intervention*: psycho‐education/sensory awareness skills training *The number of sessions*: 1 *Duration of each session on average*: 3 hours *Duration of the entire intervention*: 3 hours *Duration of the entire intervention short vs long*: short *Intervention deliverer*: the authors *Intervention form*: individual in group form Control (Nutrition) *Type of the intervention*: control *Description of the intervention*: class on nutrition/healthy eating *The number of sessions*: 1 *Duration of each session on average*: 3 hours *Duration of the entire intervention*: 3 hours *Duration of the entire intervention short vs long*: short *Intervention deliverer*: the authors *Intervention form*: individual in group form	
Outcomes	*Secondary traumatic stress (STSS)* **Outcome type**: ContinuousOutcome *Copenhagen Burnout Inventory (CBI)* **Outcome type**: ContinuousOutcome	
Identification	**Sponsorship source:** NR **Country:** USA **Setting:** Hospital **Comments:** NR **Authors name:** Linda Grabbe **Institution:** Nell Hodgson Woodruff School of Nursing, Emory University, Atlanta, GA **Email:** lgrabbe@emory.edu **Address:** Corresponding author: Linda Grabbe, Nell Hodgson Woodruff School of Nursing, Emory University, 1520 Clifton Rd, Atlanta, GA30322 **Time period:** 2017‐2018	
Notes	CBI included in analysis 1.1 and 1.2	
***Risk of bias***		
**Bias**	**Authors' judgement**	**Support for judgement**
Random sequence generation (selection bias)	Unclear risk	Quote: "These participants were then randomly placed in either the intervention or control group. The"
Allocation concealment (selection bias)	Unclear risk	Difficult to judge whether participants and/or investigators could possible foresee assignment. However, it is assumed that randomization was performed in one go and that participants and/or investigators could not foresee assignment.
Blinding of participants and personnel (performance bias) All outcomes	High risk	Participants were not blinded.
Blinding of outcome assessment (detection bias) All outcomes	High risk	Participants were not blinded whereas outcomes are self‐reported.
Incomplete outcome data (attrition bias) All outcomes	High risk	Not reported whether lost to follow‐up was at random. 40% lost.
Selective reporting (reporting bias)	Unclear risk	No protocol registration, nor did we find one.
Other bias	Unclear risk	Low response and compliance rate.

**Table:** Gunasingam 2015

***Study characteristics***		
Methods	**Study design:** randomised controlled trial **Study grouping:** parallel group	
Participants	**Baseline Characteristics** Debriefing intervention *Age (n) 20‐24, 25‐30, >30*: 4, 9, 0 *Sex (N (% female))*: 5 (28%) *Sample size*: 13 *Years of experience (mean ± SD)*: NR Control (no intervention) *Age (n) 20‐24, 25‐30, >30*: 10, 6, 0 *Sex (N (% female))*: 8 (56%) *Sample size*: 18 *Years of experience (mean ± SD)*: NR Overall *Age (n) 20‐24, 25‐30, >30*: NR *Sex (N (% female))*: NR *Sample size*: 31 *Years of experience (mean ± SD)*: NR **Included criteria:** the sample of interns invited to participate were those who were based at the teaching hospital during term 3 of 2011 **Excluded criteria:** interns who were seconded to other hospitals were excluded **Pretreatment:** reported socio‐demographic baseline characteristics of participants randomised to the intervention group were similar to socio‐demographic baseline characteristics of participants randomised to the control group. **Compliance rate:** attendance at the debriefing sessions was not always 100%, leading to the potential argument that those who were regularly in attendance were experiencing more or less stress. **Response rate:** 31 of 52 invited interns entered this study (60%). **Type of healthcare worker:** physicians	
Interventions	**Intervention characteristics** Debriefing intervention *Type of the intervention*: Intervention type 1 ‐ to focus one’s attention on the experience of stress *Description of the intervention*: Debriefing, in the context of chronic work‐related emotional and interpersonal stressors, can be described as an opportunity to meet with peers and have a facilitated discussion with a senior and trusted health professional. It essentially involves peer support, feedback, mentoring and problem‐solving. Increased support and feedback has been shown to reduce work‐related psychological stress *The number of sessions*: four sessions *Duration of each session on average*: 1 hour *Duration of the entire intervention*: NR *Duration of the entire intervention short vs long*: short *Intervention deliverer*: senior health professionals *Intervention form*: Individual in group form Control (no intervention) *Type of the intervention*: NA *Description of the intervention*: NA *The number of sessions*: NA *Duration of each session on average*: NA *Duration of the entire intervention*: NA *Duration of the entire intervention short vs long*: NA *Intervention deliverer*: NA *Intervention form*: NA	
Outcomes	*Maslach Burnout Inventory ‐ Emotional Exhaustion* **Outcome type**: ContinuousOutcome *Maslach Burnout Inventory ‐ Cynicism* **Outcome type**: ContinuousOutcome *Maslach Burnout Inventory ‐ Professional efficacy* **Outcome type**: ContinuousOutcome	
Identification	**Sponsorship source:** NR **Country:** Australia **Setting:** Hospital **Comments:** NR **Authors name:** Nishmi Gunasingam **Institution:** Royal Prince Alfred Hospital, Camperdown, New South Wales, Australia **Email:** nishmi@gmail.com **Address:** Dr Nishmi Gunasingam, Medical Training andAdministration Unit, RoyalPrince Alfred Hospital, Missenden Road, Camperdown, NSW 2050 **Time period:** 2011	
Notes	MBI‐EE included in analysis 1.1	
***Risk of bias***		
**Bias**	**Authors' judgement**	**Support for judgement**
Random sequence generation (selection bias)	Low risk	Quote: "Participants were allocated a unique identifying number to maintain anonymity. A computer generated randomisation code"
Allocation concealment (selection bias)	Unclear risk	Quote: "allocated participants to the debrieﬁng intervention or control group." Difficult to judge whether participants and/or investigators could possible foresee assignment. However, it is assumed that randomization was performed in one go and that participants and/or investigators could not foresee assignment.
Blinding of participants and personnel (performance bias) All outcomes	High risk	Participants were not blinded.
Blinding of outcome assessment (detection bias) All outcomes	High risk	Participants were not blinded whereas outcomes are self‐reported.
Incomplete outcome data (attrition bias) All outcomes	Low risk	No loss to follow‐up.
Selective reporting (reporting bias)	Unclear risk	No trial registration or no study protocol reported, nor did we find one online
Other bias	Unclear risk	Difficult to assess compliance rate ‐> Attendance at the debriefing sessions was not always 100%, leading to the potential argument that those who were regularly in attendance were experiencing more or less stress.

**Table:** Gärtner 2013

***Study characteristics***		
Methods	Cluster‐RCT, the Netherlands	
Participants	Nurses on wards of an academic hospital were screened for work and health problems: Experimental: 29 wards, 591 participants of which 151 screened positive. Control: 28 wards, 561 participants of which 161 screened positive. Experimental: 17%, Control 22% men, > 45 years age ‐ Experimental 51% Control 46%, > 10 years of experience ‐ Experimental 51% Control 41%	
Interventions	1) Experimental 1: all who screened positive were referred to Occupational Health Physician (OHP). Participants who were screened as positive were invited for a face‐to‐face preventive consultation with their occupational physician. The consultation was voluntary, and workers could reschedule or cancel it if they wished. Supervisors were not informed about the screening results or about the invitation for and content of the preventive consultation of any employee. The 7‐step protocol for OHPs closely followed occupational physicians’ care as usual for consultations initiated by the employee in contrast to the compulsory consultation in the context of absenteeism. Occupational physicians received 3 hours of training from the researchers on the use of the protocol. (CBT) 2) Experimental 2: participants received personalised feedback on their screening results immediately after filling out the baseline questionnaire, both onscreen and in an e‐mail. The personalised feedback was followed by an invitation for a tailored offer of self‐help EMH interventions, on the basis of an algorithm based on the specific symptoms and the work‐relatedness of the symptoms. participants were mostly offered a choice of 2 to 3 EMH interventions to leave room for personal preferences. Participants who screened negative on all mental health complaints were invited to follow an EMH intervention aimed at enhancing and retaining their mental fitness. The EMH interventions are self‐help interventions on the Internet aimed at reducing specific mental health complaints or enhancing well‐being. The interventions are mainly based on the principles of cognitive behavioural therapy and combine a variety of aspects, e.g. providing information and advice, weekly assignments, the option of keeping a diary and a forum to get in contact with others who have similar complaints. The EMH interventions were developed as stand‐alone interventions by the Trimbos Institute (CBT). 2) Control: waiting list: In the control arm. Participants filled out the baseline questionnaire; however, results of the screening‐questionnaires were not to be reported back to participants, and no further interventions were advised at baseline. As compensation, participants in the control arm received their personal screening results together with a tailored choice for a self‐help EMH intervention six months after baseline.	
Outcomes	Gartner 2013: the study's primary outcome was help‐seeking behaviour; we used secondary outcomes: distress from the Dutch 4DKL, anxiety and depression from Brief Symptom Inventory Ketelaar 2013: the study used work‐functioning as the primary outcome: we used the distress part of the Dutch 4DKL as stress outcome; anxiety and depression were also measured but not reported Bolier 2014: Brief Symptom Inventory (BSI) ‐ Anxiety and Brief Symptom Inventory (BSI) ‐ depression Noben 2014: cost‐effectiveness	
Identification		
Notes	We got the following data for the distress scale of the 4DKL at 6 months follow‐up for the group who screened positive from author K Nieuwenhuijsen: Experimental: N = 86 6.24 ± 6.52 Control: N = 116 6.82 ± 6.57 We got the following data from author S. Ketelaar: Distress measure with 4DKL at 6 months follow‐up for the group who screened positive: Experimental: N = 52 6.06 ± 6.54; Control: N = 116 6.82 ± 6.5.7 4DKL included in analysis 1.2 Intervention groups combined to create a single pair‐wise comparison. BSI‐depression included in analysis 1.4 Experimental 2 vs control 4DKL included in analysis 5.2 Experimental 1 vs Experimental 2	
***Risk of bias***		
**Bias**	**Authors' judgement**	**Support for judgement**
Random sequence generation (selection bias)	Low risk	" Randomization was performed at the ward level (n = 86). Randomization sequences with a block size of three were generated with Nquery Advisor (Statistical Solutions, Ltd, Cork, Ireland) by one researcher (K.N.) who was not involved in the recruitment"
Allocation concealment (selection bias)	Unclear risk	Not blinded
Blinding of participants and personnel (performance bias) All outcomes	High risk	Participants were not blinded.
Blinding of outcome assessment (detection bias) All outcomes	High risk	Participants were not blinded whereas outcomes are self‐reported.
Incomplete outcome data (attrition bias) All outcomes	High risk	At 3 months lost to follow‐up: Experimental 37% / Control 30%; at 6 months Experimental 46% / Control 34%
Selective reporting (reporting bias)	Low risk	Gartner 2013: All outcomes reported that were announced in protocol Ketelaar 2013: Anxiety and Depression were not reported in Ketelaar 2013 but in Bolier 2014
Other bias	Unclear risk	Compliance very low: 34% of those invited visited their OHP

**Table:** Günüsen 2010

***Study characteristics***		
Methods	RCT, Turkey	
Participants	Quote: "All of the nurses (n = 227) were invited to complete the Maslach Burnout Inventory (MBI) developed by Maslach & Jackson (1981). Those who completed the questionnaire and received a score on emotional exhaustion higher than the median score for all nurses were invited to participate in the burnout reduction intervention." (p. 487) 108 nurses were randomised to one of three conditions.	
Interventions	1) Coping training (N = 36) Quote: "The group that received coping training consisted of two groups, each group consisting of 18 people. In the first week, the concept of stress was explained to the nurses, and coping methods used by the nurses in stressful conditions were discussed. In the second session, basic communication skills on the stress level were discussed. In the third session, cognitive coping methods were presented theoretically. In the fourth session, cognitive distortions found among nurses and methods for coping with these distortions were discussed. In the fifth session, the problem‐solving method was theoretically explained to the nurses. In the sixth session, stressful situations that the nurses encountered were discussed and resolved by means of the problem‐solving method. In the seventh session, problems that the nurses had difficulty coping with were discussed by utilizing the skills learnt during the course of the programme." (p. 488) 2) Support group (N = 36) "...the support group consisted of three groups, each group consisting of 12 people. The nurses talked about the most frequently encountered stressors in the workplace and expressed their feelings towards their jobs. At the beginning of each session, the nurses expressed their feelings related to difficult situations at the workplace. Then, a problem chosen by the nurses was attempted to be solved by using reflective cycle steps. Researchers provided information when needed. Possible solution methods were discussed in the groups, and the nurses were advised to use these methods in their daily lives. The nurses shared their difficult and favourable times and also exchanged recommendations with each other." (p. 488) 3) Control: No intervention (N = 36)	
Outcomes	MBI	
Identification		
Notes	MBI‐EE included in analysis 1.1 and 1.2. Intervention groups combined to create a single pair‐wise comparison.	
***Risk of bias***		
**Bias**	**Authors' judgement**	**Support for judgement**
Random sequence generation (selection bias)	Low risk	"Random allocation was concealed by using a system of sequentially numbered, opaque, sealed envelopes containing the computer‐generated random allocation, which had been drawn up by a statistician. During the randomization, the researchers and the participants did not know the groups to which they would be allocated." (p. 487)
Allocation concealment (selection bias)	Low risk	See above
Blinding of participants and personnel (performance bias) All outcomes	High risk	"No blinding was applied to the participants and the researchers." (p. 487)
Blinding of outcome assessment (detection bias) All outcomes	High risk	Participants were not blinded whereas outcomes are self‐reported.
Incomplete outcome data (attrition bias) All outcomes	Low risk	"Intention‐to‐treat analysis was used because of sample loss." (p. 487)
Selective reporting (reporting bias)	Low risk	The authors only measured and adequately reported results of the MBI.
Other bias	Unclear risk	We did not find any indications of other sources of bias.

**Table:** Hersch 2016

***Study characteristics***		
Methods	**Study design:** randomised controlled trial **Study grouping:** parallel group	
Participants	**Baseline Characteristics** Web‐based BREATHE *Age categories 22‐26, 27‐31, 32‐36, 37‐41, 42‐46, 47‐51, 52‐56, 57‐61, 62‐66 (n(%))*: 7 (13%), 7 (13%), 5 (10%), 7(13%), 2 (4%), 9 (17%), 5 (10%), 8 (15%), 2 (4%) *Sex (N (% female))*: 48 (92%) *Sample size*: 52 *Years of experience (mean ± SD)*: NR Control (no intervention) *Age categories 22‐26, 27‐31, 32‐36, 37‐41, 42‐46, 47‐51, 52‐56, 57‐61, 62‐66 (n(%))*: 15 (29%), 2 (4%), 7 (13%), 3 (6%), 4 (8%), 5 (10%), 11 (21%), 5 (10%), 0 (0) *Sex (N (% female))*: 43 (83%) *Sample size*: 52 *Years of experience (mean ± SD)*: NR Overall *Age categories 22‐26, 27‐31, 32‐36, 37‐41, 42‐46, 47‐51, 52‐56, 57‐61, 62‐66 (n(%))*: NR *Sex (N (% female))*: NR *Sample size*: 104 *Years of experience (mean ± SD)*: NR **Included criteria:** Nurses had to be 21 years of age or older and work at one of the participating hospitals **Excluded criteria:** NR **Pretreatment:** reported socio‐demographic baseline characteristics of participants randomised to the intervention group were similar to socio‐demographic baseline characteristics of participants randomised to the control group. **Compliance rate:** the majority of program group participants logged into the program 1 to 3 times. Ten participants in the experimental group never logged into the program. The average number of logins for those who logged in at least once was 2.5. The average amount of time spent in the BREATHE program was 43 minutes. **Response rate:** of 117 eligible participants 105 participated > 88% **Type of healthcare worker:** nurses	
Interventions	**Intervention characteristics** Web‐based BREATHE *Type of the intervention*: Intervention type 4 combination to focus one’s attention on the experience of stress & to focus on work‐related risk factors on an individual level. *Description of the intervention*: The intervention consisted of seven parts: Welcome and Introduction; Assess Your Stress; Identify Stressors, manage Stress; Avoid Negative Coping; and Your Mental Health. The Manager’s Role includes additional information for nurse managers on identifying workplace stressors and reducing stress through positive management practices. *The number of sessions*: as often as the target‐group wanted *Duration of each session on average*: as long as the target‐group wanted *Duration of the entire intervention*: 3 months *Duration of the entire intervention short vs long*: short *Intervention deliverer*: NR *Intervention form*: Individual (digital) Control (no intervention) *Type of the intervention*: NA *Description of the intervention*: NA *The number of sessions*: NA *Duration of each session on average*: NA *Duration of the entire intervention*: NA *Duration of the entire intervention short vs long*: NA *Intervention deliverer*: NA *Intervention form*: NA	
Outcomes	*Nursing Stress Scale* **Outcome type**: ContinuousOutcome *Symptoms of distress* **Outcome type**: ContinuousOutcome	
Identification	**Sponsorship source:** NR **Country:** USA **Setting:** Hospital **Comments:** NR **Authors name:** Rebekah K. Hersch **Institution:** ISA Associates, Inc. **Email:** rhersch@isagroup.com **Address:** SA Associates, Inc., 201 North Union Street, Suite 330, Alexandria, Virginia **Time period:** NR	
Notes	Symptoms of distress included in analysis 4.1	
***Risk of bias***		
**Bias**	**Authors' judgement**	**Support for judgement**
Random sequence generation (selection bias)	Low risk	Quote: "Randomization was conducted using a block randomized design with blocks of 4 and 6. The 0 and 1 within each block were random and the order of the group of 4 and the group of 6 was random. Randomization occurred after each participant completed the pretest question‐ naire. The online questionnaire site was checked every day to determine who completed the pretest each day and individuals were assigned to the next condition on the randomization table as they completed the questionnaire."
Allocation concealment (selection bias)	Unclear risk	Quote: "Once randomization was complete, participants were notiﬁed of the condition to which they were assigned (no blinding procedures were employed) and were informed of next steps; experimental group participants were sent a link to the BREATHE program along with a randomly generated username and password and instructions for using the program." Insufficient information to understand whether intervention allocations could have been foreseen in advance of, during, enrolment.
Blinding of participants and personnel (performance bias) All outcomes	High risk	Participants were not blinded.
Blinding of outcome assessment (detection bias) All outcomes	High risk	Participants were not blinded whereas outcomes are self‐reported.
Incomplete outcome data (attrition bias) All outcomes	Low risk	14 of the 104 randomised participants were lost to follow‐up (1 control group vs 13 intervention group). Missing data were imputated. Missing not at random. We found that the following participants were less likely to respond to the posttest measure: those who reported greater number of days in which they had five or more drinks on the same occasion at pretest, those who reported more drinks per day at pretest, and those who had lower scores on the understanding of depression and anxiety measure at pretest
Selective reporting (reporting bias)	Unclear risk	No trial registration or no study protocol reported, nor did we find one online
Other bias	Low risk	No indication of other sources of bias

**Table:** Hilcove 2021

***Study characteristics***		
Methods	**Study design:** randomised controlled trial **Study grouping:** Parallel group	
Participants	**Baseline characteristics** Mindfulness‐based Yoga *Age (mean ± SD)*: 42 ± NR *Sex (N (% female))*: 38 (95%) *Sample size*: 41 *Years of experience (mean ± SD)*: NR Control (no intervention) *Age (mean ± SD)*: 42 ± nr *Sex (N (% female))*: 35 (95%) *Sample size*: 37 *Years of experience (mean ± SD)*: NR Overall *Age (mean ± SD)* : 42 ± 12.1 *Sex (N (% female))*: NR *Sample size*: 78 *Years of experience (mean ± SD)*: NR **Included criteria:** employees who provided direct patient care (including but not limited to nurses, nursing assistants, therapists, physicians, and social workers), older than 18 years. **Excluded criteria:** the presence of joint or muscle problems that limited mobility (e.g. advanced arthritis, herniated disk, or past injuries that prevent painless or safe movement), having routinely practised yoga or any other MB intervention in the past 6 months, or currently on medication that might interact with the results of salivary cortisol measures, including prednisone, cortisone, or steroid‐based medicine. **Pretreatment:** reported socio‐demographic baseline characteristics of participants randomised to the intervention group were similar to socio‐demographic baseline characteristics of participants randomised to the control group. **Compliance rate:** there was 98.7% attendance across all sessions for those in the MB yoga intervention group. **Response rate:** NR **Type of healthcare worker:** Nurses & other healthcare professionals	
Interventions	**Intervention characteristics** Mindfulness‐based Yoga *Type of the intervention*: Intervention type 2 ‐ to focus one’s attention away from the experience of stress *Description of the intervention*: Based on a combination of Hatha and Raja Yoga practices. The MB yoga intervention was a beginner level program, starting with seated centering, brief teaching about yoga focused attention on the breath, and yogic breath practice (complete yogic breath and alternate nostril breathing). *The number of sessions*: 6 weeks *Duration of each session on average*: NR *Duration of the entire intervention*: 6 weeks *Duration of the entire intervention short vs long*: Short *Intervention deliverer*: NR *Intervention form*: NR Control (no intervention) *Type of the intervention*: NA *Description of the intervention*: NA *The number of sessions*: NA *Duration of each session on average:* NA *Duration of the entire intervention*: NA *Duration of the entire intervention short vs long*: NA *Intervention deliverer*: NA *Intervention form*: NA	
Outcomes	*Maslach Burnout Inventory ‐ Emotional Exhaustion* **Outcome type**: ContinuousOutcome *The Perceived Stress Scale (PSS)* **Outcome type**: ContinuousOutcome	
Identification	**Sponsorship source:** NR **Country:** USA **Setting:** Hospital **Comments:** NR **Authors name:** Kelly Hilcove **Institution:** Honor Health Scottsdale Shea Medical Center **Email:** Kelly@KellyHilcove.com **Address:** BSN, RN, HNB‐BC, Board Certified Holistic Nurse, Honor Health Scottsdale Shea Medical Center, 9003 East Shea Boulevard, Scottsdale, AZ 85261, **Time period:** NR	
Notes	PSS included in analysis 2.1	
***Risk of bias***		
**Bias**	**Authors' judgement**	**Support for judgement**
Random sequence generation (selection bias)	Low risk	Quote: "Once identiﬁed as eligible, participants signed consent, completed subjective assessments, and were randomly assigned to the intervention or control group using a computerized randomization tool."
Allocation concealment (selection bias)	Unclear risk	Difficult to judge whether participants and/or investigators could possibly foresee assignment. However, it is assumed that randomization was performed in one go and that participants and/or investigators could not foresee the assignment.
Blinding of participants and personnel (performance bias) All outcomes	High risk	Participants were not blinded.
Blinding of outcome assessment (detection bias) All outcomes	High risk	Participants were not blinded whereas outcomes are self‐reported.
Incomplete outcome data (attrition bias) All outcomes	Low risk	Quote: "Two members of the control group were not able to participate in collection of post‐intervention data, due to personal time constraints, yielding an attrition rate of 2.5%."
Selective reporting (reporting bias)	Unclear risk	No trial registration or no study protocol reported, nor did we fine one online
Other bias	Unclear risk	Response rate not reported.

**Table:** Ho 2021

***Study characteristics***		
Methods	**Study design:** randomised controlled trial **Study grouping:** parallel group	
Participants	**Baseline characteristics** Mindful‐Compassion Art‐based Therapy (MCAT) *Age (mean ± SD)*: 44 ± 11.5 *Sex (N (% female))*: 22 (76%) *Sample size*: 29 *Years of experience range (N (%)) 1‐5 years, 6‐10 years, 10 years or above*: 20 (69%), 7 (24%), 2 (7%) Waitlist‐control *Age (mean ± SD)*: 45 ± 10.4 *Sex (N (% female))*: 20 (74%) *Sample size*: 27 *Years of experience range (N (%)) 1‐5 years, 6‐10 years, 10 years or above*: 19 (70%), 6 (22%), 2 (7%) Overall *Age (mean ± SD)*: NR *Sex (N (% female))*: NR *Sample size*: 56 *Years of experience range (N (%)) 1‐5 years, 6‐10 years, 10 years or above*: NR **Included criteria:** inclusion criteria included healthcare workers (i.e. physicians, nurse, medical social workers, and allied health professionals) whose primary job was caring for terminally ill patients, 21 years old and above, and fluent in both written and spoken English **Excluded criteria:** exclusion criteria included the inability to provide informed consent or major depression (or other mental health conditions) or both. **Pretreatment:** reported socio‐demographic baseline characteristics of participants randomised to the intervention group were similar to socio‐demographic baseline characteristics of participants randomised to the control group. **Compliance rate:** NR **Response rate:** NR **Type of healthcare worker:** physicians, nurse, medical social workers, and allied health professionals	
Interventions	**Intervention characteristics** Mindful‐Compassion Art‐based Therapy (MCAT) *Type of the intervention*: Intervention type 2 ‐ to focus one’s attention away from the experience of stress *Description of the intervention*: Mindfulness meditation with the expressive power of art‐based therapy to support and enhance the psycho‐socio‐emotional health of healthcare worker. Each MCAT session covered a unique topic that aims to promote understanding, acceptance, and compassion for self and others to cultivate psychological resilience and shared meaning. *The number of sessions*: 6 *Duration of each session on average*: 3 hours *Duration of the entire intervention*: 6 weeks *Duration of the entire intervention short vs long*: short *Intervention deliverer*: MCAT therapists including one accredited art therapist and one clinical researcher trained in mindfulness‐based stress reduction *Intervention form*: Group (face‐to‐face) Waitlist‐control *Type of the intervention*: Waitlist *Description of the intervention*: NA *The number of sessions*: NA *Duration of each session on average*: NA *Duration of the entire intervention*: NA *Duration of the entire intervention short vs long*: NA *Intervention deliverer*: NA *Intervention form*: NA	
Outcomes	*Maslach Burnout inventory general survey‐ burn‐out* **Outcome type**: ContinuousOutcome *Maslach Burnout inventory general survey‐ exhaustion* **Outcome type**: ContinuousOutcome *Maslach Burnout Inventory ‐ cynicism* **Outcome type**: ContinuousOutcome *Maslach Burnout Inventory ‐ professional efficacy* **Outcome type**: ContinuousOutcome	
Identification	**Sponsorship source:** NR **Country:** Singapore **Setting:** Hospice **Comments:** NR **Authors name:** Andy Hau Yan Ho **Institution:** Action Research for Community Health Laboratory, Psychology Programme, School of Social Sciences, Nanyang Technological University, Singapore, Singapore **Email:** andyhyho@ntu.edu.sg **Address:** NR **Time period:** NR	
Notes	MBI‐EE included in analysis 2.1	
***Risk of bias***		
**Bias**	**Authors' judgement**	**Support for judgement**
Random sequence generation (selection bias)	Low risk	Quote: "Simple randomization for each recruitment round was conducted by using an allocation sequence based on a computer‐generated list of random numbers. Specifically, a random number sequence ranging from 1 to 18 or 20 (depending on the number of participants recruited in each recruitment round) was generated via Research Randomizer (Urbaniak and Plous, 2019). Thereafter, each participant was randomly assigned a unique number from the sequence.
Allocation concealment (selection bias)	Unclear risk	See above. Difficult to judge whether participants and/or investigators could possibly foresee assignment.
Blinding of participants and personnel (performance bias) All outcomes	High risk	Participants were not blinded.
Blinding of outcome assessment (detection bias) All outcomes	High risk	Participants were not blinded whereas outcomes are self‐reported.
Incomplete outcome data (attrition bias) All outcomes	Low risk	No attrition throughout the entire research period.
Selective reporting (reporting bias)	Low risk	Trial registration NCT03440606. No indication of selective outcome reporting.
Other bias	Unclear risk	Response rate and compliance rate not reported.

**Table:** Huang 2020

***Study characteristics***		
Methods	**Study design:** randomised controlled trial **Study grouping:** parallel group	
Participants	**Baseline characteristics** Balint group intervention *Age 18‐25 26‐30 31‐40 (n)*: 18‐25 (15%) 26‐30 (39%) 31‐40 (22%) *Sex (N (% female))*: 55 (NR) *Sample size*: 70 *Years of experience (mean ± SD)*: NR Control (no intervention) *Age 18‐25, 26‐30, 31‐40 (n)*: 18‐25 (14%) 26‐30 (40%) 31‐40 (21%) *Sex (N (% female))*: 56 (NR) *Sample size*: 76 *Years of experience (mean ± SD)*: NR Overall *Age 18‐25 26‐30 31‐40 (n)*: NR *Sex (N (% female))*: NR *Sample size*: 146 *Years of experience (mean ± SD)*: NR **Included criteria:** The inclusion criteria encompassed (1) working in an ICU a licensed nurse for at least one year and working in hospitals with at least 1000 beds and 100 ICU nurses. **Excluded criteria:** the exclusion criteria were: (1) Participants who have neuropsychiatric disorders; (2) Participants in pregnancy or lactation; (3) Participants who incomplete or invalid questionnaire. **Pretreatment:** reported socio‐demographic baseline characteristics of participants randomised to the intervention group were similar to socio‐demographic baseline characteristics of participants randomised to the control group. **Compliance rate:** 100% **Response rate:** 100% **Type of healthcare worker:** nurses	
Interventions	**Intervention characteristics** Balint group intervention *Type of the intervention*: Intervention type 1 ‐ to focus one’s attention on the experience of stress *Description of the intervention*: Balint group training, including the case reports and group discussions, attempt to throw light on the doctor‐patient relationship *The number of sessions*: 8 *Duration of each session on average*: 1.5 hours *Duration of the entire intervention*: 8 *Duration of the entire intervention short vs long*: Short *Intervention deliverer*: Senior Balint trainers *Intervention form*: Group (face‐to‐face) Control (no intervention) *Type of the intervention*: NA *Description of the intervention*: NA *The number of sessions*: NA *Duration of each session on average*: NA *Duration of the entire intervention*: NA *Duration of the entire intervention short vs long*: NA *Intervention deliverer*: NA *Intervention form*: NA	
Outcomes	*Maslach Burnout Inventory ‐ Emotional Exhaustion* **Outcome type**: ContinuousOutcome *Maslach Burnout Inventory ‐ Depersonalisation* **Outcome type**: ContinuousOutcome *Maslach Burnout Inventory ‐ Personal accomplishment* **Outcome type**: ContinuousOutcome	
Identification	**Sponsorship source:** NR **Country:** China **Setting:** Hospital **Comments:** NR **Authors name:** Huigen Huang **Institution:** Guangdong Provincial People’s Hospital, Guangdong Academy of Medical Sciences, Guangzhou, Guangdong, China **Email:** gdpphhuanghuigen@163.com **Address:** No. 102 Zhongshan Er Road, Guangdong Provincial People’s Hospital, Guangdong Academy of Medical Sciences, Guangzhou, Guangdong, 510080, China. **Time period:** 2016	
Notes	MBI‐EE included in analysis 1.1	
***Risk of bias***		
**Bias**	**Authors' judgement**	**Support for judgement**
Random sequence generation (selection bias)	Low risk	Quote: "under the leadership of Guangdong General Hospital from May 2016 to November 2016. The participants were selected through random sampling first, then they were divided into two groups (i.e. the intervention group and the control group) with a random number generator."
Allocation concealment (selection bias)	Unclear risk	Then they were divided into two groups (i.e. the intervention group and the control group) with a random number generator Insufficient information to understand whether intervention allocations could have been foreseen in advance of, during, enrolment.
Blinding of participants and personnel (performance bias) All outcomes	High risk	Participants were not blinded.
Blinding of participants and personnel (performance bias) All outcomes	High risk	Participants were not blinded.
Blinding of outcome assessment (detection bias) All outcomes	High risk	Participants were not blinded whereas outcomes are self‐reported.
Incomplete outcome data (attrition bias) All outcomes	Low risk	6 of the 152 randomised participants withdrew (4%). Reasons provided. Missing not at random however loss to follow‐up is below our pre‐defined cut‐off point.
Selective reporting (reporting bias)	Unclear risk	No trial registration or no study protocol reported, nor did we find one online.
Other bias	Low risk	No indication of other sources of bias

**Table:** Huang 2020a

***Study characteristics***		
Methods	**Study design:** randomised controlled trial **Study grouping:** parallel group	
Participants	**Baseline characteristics** Balint intervention *Age (mean ± SD)*: 24 ± 0.90 *Sex (N (% female))*: 12 (67%) *Sample size*: 18 *Years of experience (mean ± SD)*: NR Wait‐list control group *Age (mean ± SD)*: 23 ± 0.92 *Sex (N (% female))*: 13 (72%) *Sample size*: 18 *Years of experience (mean ± SD)*: NR Overall *Age (mean ± SD)*: NR *Sex (N (% female))*: NR *Sample size*: 36 *Years of experience (mean ± SD)*: NR **Included criteria:** NR **Excluded criteria:** NR **Pretreatment:** there were no significant differences between the two groups for age and gender **Compliance rate:** 100% **Response rate:** all residents invited voluntarily participated (100% response rate). **Type of healthcare worker:** residents	
Interventions	**Intervention characteristics** Balint intervention *Type of the intervention*: Intervention type 1 ‐ to focus one’s attention on the experience of stress *Description of the intervention*: Each participant in the discussion groups could volunteer to report his/her case. The group leader facilitated the entire Balint session. Discussions were largely case‐focused and highlighted the emotions and attitudes aroused by participants from the presentation. *The number of sessions*: 10 sessions *Duration of each session on average*: 1 hour *Duration of the entire intervention*: 6 months *Duration of the entire intervention short vs long*: Long *Intervention deliverer*: Group leaders formally trained and qualified by the “Asia‐link Program” *Intervention form*: Group (face‐2‐face) Wait‐list control group *Type of the intervention*: Wait‐list control *Description of the intervention*: NA *The number of sessions*: NA *Duration of each session on average*: NA *Duration of the entire intervention*: NA *Duration of the entire intervention short vs long*: NA *Intervention deliverer* : NA *Intervention form*: NA	
Outcomes	*Maslach Burnout Inventory human services survey ‐ Emotional Exhaustion* **Outcome type**: ContinuousOutcome *Maslach Burnout Inventory human services survey ‐ Depersonalisation* **Outcome type**: ContinuousOutcome *Maslach Burnout Inventory human services survey ‐ Personal accomplishment* **Outcome type**: ContinuousOutcome	
Identification	**Sponsorship source:** NR **Country:** China **Setting:** Hospital **Comments:** NR **Authors name:** Lei Huang **Institution:** Department of Psychiatry, Tongji Hospital, Tongji University School of Medicine, Shanghai, China, Medical Education Division, Tongji Hospital, Tongji University School of Medicine, Shanghai, China **Email:** wuwy@tongji.edu.cn **Address:** NR **Time period:** 2016	
Notes	MBI‐EE Included in analysis 1.2	
***Risk of bias***		
**Bias**	**Authors' judgement**	**Support for judgement**
Random sequence generation (selection bias)	Unclear risk	Quote: "Following consent and the completion of the ﬁrst round of assessment completion, the 36 residents were randomly assigned to the intervention (n = 18) or the control group (n = 18)." Sequence generation process insufficiently described
Allocation concealment (selection bias)	Unclear risk	Difficult to judge whether participants and/or investigators could possible foresee assignment. However, it is assumed that randomization was performed in one go and that participants and/or investigators could not foresee assignment.
Blinding of participants and personnel (performance bias) All outcomes	High risk	Participants were not blinded.
Blinding of outcome assessment (detection bias) All outcomes	High risk	Participants were not blinded whereas outcomes are self‐reported.
Incomplete outcome data (attrition bias) All outcomes	Low risk	No loss to follow‐up.
Selective reporting (reporting bias)	Low risk	No trial registration or no study protocol reported. No indication of selective reporting.
Other bias	Low risk	No indication of other sources of bias reported.

**Table:** Janzarik 2022

***Study characteristics***		
Methods	**Study design:** randomised controlled trial **Study grouping:** parallel group	
Participants	**Baseline characteristics** Intervention (resilience) *Age (mean ± SD)*: 47.4 ± 10.8 *Sex (N (% female))*: 35 (92%) *Sample size*: 38 *Years of experience (mean ± SD)*: NR Control (no intervention) *Age (mean ± SD)*: 46.5 ± 10.4 *Sex (N (% female))*: 31 (91%) *Sample size*: 34 *Years of experience (mean ± SD)*: NR Overall *Age (mean ± SD)*: NR *Sex (N (% female))*: NR *Sample size*: 72 *Years of experience (mean ± SD)*: NR **Included criteria:** NR **Excluded criteria:** NR **Pretreatment:** there were no significant differences between intervention and control group regarding age, gender, marital status, weekly working hours, and stressor load before the intervention. **Compliance rate:** NR **Response rate:** NR **Type of healthcare worker:** nurses	
Interventions	**Intervention characteristics** Intervention (resilience) *Type of the intervention*: Intervention type 4 ‐ Combination of to focus one’s attention on the experience of stress & to focus one’s attention away from the experience of stress *Description of the intervention*: The training included therapy elements from cognitive behavioural therapy and psychodynamic psychotherapy. Additionally, mindfulness and imagination exercises were included. The aim of the intervention was to provide participants with new skills to help them cope better with individual stressors. *The number of sessions*: 8 *Duration of each session on average*: 120 minutes *Duration of the entire intervention*: 8 weeks *Duration of the entire intervention short vs long*: Short *Intervention deliverer*: Psychologist *Intervention form*: Group Control (no intervention) *Type of the intervention*: NA *Description of the intervention*: NA *The number of sessions*: NA *Duration of each session on average*: NA *Duration of the entire intervention*: NA *Duration of the entire intervention short vs long*: NA *Intervention deliverer*: NA *Intervention form*: NA	
Outcomes	*General Health Questionnaire‐28‐ (GHQ‐28)* **Outcome type**: ContinuousOutcome *Mainz Inventory of Microstressors (MIMI)* **Outcome type**: ContinuousOutcome *Perceived Stress Scale (PSS)* **Outcome type**: ContinuousOutcome	
Identification	**Sponsorship source:** NR **Country:** Germany **Setting:** Hospital **Comments:** NR **Authors name:** Gesche Janzarik **Institution:** Leibniz Institute for Resilience Research **Email:** pt.janzarik@gmail.com **Address:** Leibniz Institute for Resilience Research (LIR) Mainz, 55122 Mainz, Germany **Time period:** NR	
Notes	PSS included in analysis 4.1 and 4.2	
***Risk of bias***		
**Bias**	**Authors' judgement**	**Support for judgement**
Random sequence generation (selection bias)	Unclear risk	Quote: "This randomised controlled trial included three assessment points: pre‐test, post‐test, and three follow‐up measurements at three, six, and nine months." Not reported how randomisation took place (e.g. software)
Allocation concealment (selection bias)	Unclear risk	Difficult to judge whether participants and/or investigators could possible foresee assignment. However, it is assumed that randomization was performed in one go and that participants and/or investigators could not foresee assignment.
Blinding of participants and personnel (performance bias) All outcomes	High risk	Participants were not blinded.
Blinding of outcome assessment (detection bias) All outcomes	High risk	Participants were not blinded whereas outcomes are self‐reported.
Incomplete outcome data (attrition bias) All outcomes	High risk	58 of the 72 (80%) randomised participants responded to the latest follow‐up time included in this review. Reasons provided. Not mentioned whether missing was at random.
Selective reporting (reporting bias)	Unclear risk	No trial registration or no study protocol reported, nor did we find one online.
Other bias	Unclear risk	Compliance rate and response rate not reported.

**Table:** Jensen 2006

***Study characteristics***		
Methods	Cluster‐RCT, Denmark	
Participants	210 eldercare workers	
Interventions	1) Experimental 1: Stress Management Intervention: The SMI was developed to address the work stress in health care with particular attention to prevention of burnout and development of strategies for stress management. Training occurred over 20 weeks, with group sessions every 2 weeks, and each session lasting 2 hours. Between sessions, the participants were given assignments concerning implementation of the programme in daily practice. 2) Experimental 2: Transfer Technique Intervention: The TTI was based on the Stockholm training concept, which aims to reduce the biomechanical load on the back, minimise work in asymmetric postures, and prevent sudden unexpected loads. Training in the TTI arm was a combination of practical classroom education (24 hours for each worker) and instruction at the work site. There were 11 instructors who belonged to the 7 groups in the TTI arm, with 1 to 2 persons in each group who received 30 hours of education during the initial phase of the study. 3) Control: Reference Programme consisting of lessons of the participants' own choice in matters unrelated to the intervention programmes but of the same duration as the active intervention lessons (e.g. on skin care, proper treatment of a person with diabetes, etc.)	
Outcomes	MBI (results not reported in article but obtained directly from author)	
Identification	MBI‐EE included in analysis 1.2	
Notes		
***Risk of bias***		
**Bias**	**Authors' judgement**	**Support for judgement**
Random sequence generation (selection bias)	Unclear risk	"Randomization was performed at group level because the intervention programs were meant to involve the employee as a group during education and implementation. The assignment to the different intervention programs was balanced to secure representation of all 3 programs in each of the wards." (p.1762)
Allocation concealment (selection bias)	Unclear risk	Not reported.
Blinding of participants and personnel (performance bias) All outcomes	High risk	Participants not blinded.
Blinding of outcome assessment (detection bias) All outcomes	High risk	Participants were not blinded whereas outcomes are self‐reported.
Incomplete outcome data (attrition bias) All outcomes	High risk	Quote: "Altogether, 163 members of the source population (79%) participated in both baseline and follow‐up investigation, and completed at least 2 sets of diaries during the study period. The proportion of dropouts from baseline to follow‐up did not differ significantly across intervention groups. We observed no differences in age and number of years occupied in health care and mean intensity of LBP during the past year between participants who remained in the study and participants who dropped out." (p.1762).
Selective reporting (reporting bias)	High risk	Results data for the MBI, Setterlind's Stress Scores and rating of social support were not reported because they were not statistically significantly different between groups. "...[N]o significant changes were found in either of the intervention arms in ... the Maslach Burnout Inventory, the Setterlind stress scores, or the rating of social support (data not shown)" (p. 1765)
Other bias	Low risk	We did not find any indications of other sources of bias.

**Table:** Kavurmaci 2022

***Study characteristics***		
Methods	**Study design:** randomised controlled trial **Study grouping:** parallel group	
Participants	**Baseline characteristics** Yoga *Age (mean ± SD)*: 39.4 ± 9.4 *Sex (N (% female))*: NR *Sample size*: 33 *Years of experience (mean ± SD)*: NR Control (no intervention) *Age (mean ± SD)*: 37.9 ± 8.9 *Sex (N (% female))*: NR *Sample size*: 34 *Years of experience (mean ± SD)*: NR Overall *Age (mean ± SD)*: NR *Sex (N (% female))*: NR *Sample size*: 67 *Years of experience (mean ± SD)*: NR **Included criteria:** not participating in yoga and similar regular exercise program during the research, not having any health problems that will prevent yoga, to agree to participate in the study. **Excluded criteria:** NR **Pretreatment:** reported sociodemographic baseline characteristics of participants randomised to the intervention group were similar to sociodemographic baseline characteristics of participants randomised to the control group. **Compliance rate:** two of the 35 participants in the experimental group excluded from the study because they did not regularly participate in yoga practice. **Response rate:** Of 80 participants 67 participated > 84% **Type of healthcare worker:** nurses	
Interventions	**Intervention characteristics** Yoga *Type of the intervention*: Intervention type 2 ‐ to focus one’s attention away from the experience of stress *Description of the intervention*: In yoga practice, asanas, breathing exercises, relaxation and meditation techniques were applied. Yoga practice consisted of standing breathing exercise, sitting breathing exercise, lying breathing exercise, sitting relaxation (meditation) and deep relaxation. *The number of sessions*: two times per week *Duration of each session on average*: 60 to 90 minutes *Duration of the entire intervention*: 8 weeks *Duration of the entire intervention short vs long*: short *Intervention deliverer*: Researcher who is a certified yoga instructor *Intervention form*: Individual on a group‐level Control (no intervention) *Type of the intervention*: NA *Description of the intervention*: NA *The number of sessions*: NA *Duration of each session on average*: NA *Duration of the entire intervention*: NA *Duration of the entire intervention short vs long*: NA *Intervention deliverer*: NA *Intervention form*: NA	
Outcomes	*Maslach Burnout Inventory ‐ Emotional Exhaustion* **Outcome type**: ContinuousOutcome *Maslach Burnout Inventory ‐ Depersonalisation* **Outcome type**: ContinuousOutcome *Maslach Burnout Inventory ‐ Personal accomplishment* **Outcome type**: ContinuousOutcome	
Identification	**Sponsorship source:** NR **Country:** Turkey **Setting:** Nursing faculty **Comments:** NR **Authors name:** Mehtap Kavurmaci **Institution:** Department of Internal Medicine Nursing, Atatürk University, Erzurum, Turkey **Email:** m.curcani@hotmail.com **Address:** Mehtap Kavurmaci, PhD, Department of InternalMedicine Nursing, Atatürk University, Erzurum25080, Turkey **Time period:** 2019	
Notes	MBI‐EE included in analysis 2.1	
***Risk of bias***		
**Bias**	**Authors' judgement**	**Support for judgement**
Random sequence generation (selection bias)	Low risk	Quote: "The individuals were selected for the sample by the probability sampling method of simple random sampling. These individuals were listed for the simple random sampling method, and it was select 70 individuals from the table of random numbers including 35 in the experiment group and 35 in the control group."
Allocation concealment (selection bias)	Unclear risk	Insufficient information to understand whether intervention allocations could have been foreseen in advance of, during, enrolment.
Blinding of participants and personnel (performance bias) All outcomes	High risk	Participants were not blinded.
Blinding of outcome assessment (detection bias) All outcomes	High risk	Participants were not blinded whereas outcomes are self‐reported.
Incomplete outcome data (attrition bias) All outcomes	Low risk	Two of the participants in the experimental group were excluded from the study because they did not regularly participate in yoga practice. One of the participants in the control group was excluded from the study because he did not complete his final test. Unknown whether non‐completers differed from completers however loss to follow‐up is below our pre‐defined cut‐off point.
Selective reporting (reporting bias)	Unclear risk	No trial registration or no study protocol reported, nor did we find one online.
Other bias	Low risk	No indication of other sources of bias.

**Table:** Kesselheim 2020

***Study characteristics***		
Methods	**Study design:** cluster‐randomised controlled trial **Study grouping:** parallel group	
Participants	**Baseline characteristics** Novel training (intervention group) *Age (N (%)) 26‐30, 31‐35, 36‐40, 41‐50*: 17 (29%), 38 (64%), 4 (7%), 0 *Sex (N (% female))*: 45 (76%) *Sample size*: 59 *Years of experience (mean ± SD)*: NR Usual training (control group) *Age (N (%)) 26‐30, 31‐35, 36‐40, 41‐50*: 12 (29%), 27 (66%), 1 (2%), 1 (2%) *Sex (N (% female))*: 31 (76%) *Sample size*: 41 *Years of experience (mean ± SD)*: NR Overall *Age (N (%)) 26‐30, 31‐35, 36‐40, 41‐50*: NR *Sex (N (% female))*: NR *Sample size*: 100 *Years of experience (mean ± SD)*: NR **Included criteria:** NR **Excluded criteria:** NR **Pretreatment:** there were no significant differences between the UT and intervention arms with respect to age, gender, or additional professional degrees. At baseline, pretest data reveal that UT and intervention groups did not differ significantly in their scores on the PHOSAH, MBI, PPOS, or Empowerment at Work Scale (Table 2). Mean scores on the PHOSAH, the primary outcome measure, were 7.4 (SD 4.2) for fellows in the UT group and 8.2 (SD 3.3) for the intervention group (P = 0.35). However, baseline performance on the PHOSAH was somewhat lower than previously published, with mean score of 9 (SD 3.4) .15 Fellows in both groups had similar levels of satisfaction with their fellowship training in several domains of humanism and professionalism (Table 3). **Compliance rate:** NR **Response rate:** NR **Type of healthcare worker:** paediatric haematology‐oncology fellows	
Interventions	**Intervention characteristics** Novel training (intervention group) *Type of the intervention*: Intervention type 1 ‐ to focus one’s attention on the experience of stress *Description of the intervention*: The intervention arm of this study futilises a novel, 4‐module, case‐based curriculum which aims to foster PHO fellows’ reflection on the feelings, challenges, and conflicts arising in the care of children and families affected by cancer or blood disorders. *The number of sessions*: NR *Duration of each session on average*: NR *Duration of the entire intervention*: NR *Duration of the entire intervention short vs long*: short *Intervention deliverer*: faculty facilitator *Intervention form*: group Usual training (control group) *Type of the intervention*: NR *Description of the intervention*: NR *The number of sessions*: NR *Duration of each session on average*: NR *Duration of the entire intervention*: NR *Duration of the entire intervention short vs long*: NR *Intervention deliverer*: NR *Intervention form*: NR	
Outcomes	*Maslach Burnout Inventory ‐ Emotional Exhaustion* **Outcome type**: ContinuousOutcome *Maslach Burnout Inventory ‐ Depersonalisation* **Outcome type**: ContinuousOutcome *Maslach Burnout Inventory ‐ Personal accomplishment (lack of)* **Outcome type**: ContinuousOutcome	
Identification	**Sponsorship source:** NR **Country:** USA **Setting:** Hospital **Comments:** NR **Authors name:** Jennifer Kesselheim **Institution:** Department of Pediatric Oncology, Dana‐Farber/Boston Children’s Cancer and Blood Disorders Center, Boston, Massachusettes **Email:** Jennifer_kesselheim@dfci.harvard.edu **Address:** Jennifer Kesselheim, Dana‐Farber/Boston Children’s Cancer and Blood Disorders Center, Boston, Massachusettes 450 Brookline Avenue, D1107, Boston, MA02215 **Time period:** 2016‐2017	
Notes	MBI‐EE included in analysis 1.1	
***Risk of bias***		
**Bias**	**Authors' judgement**	**Support for judgement**
Random sequence generation (selection bias)	Unclear risk	Quote: Program directors committed to study participation sought local approval from their Institutional Review Board after which their program was randomized to either the intervention or UT arm of the study. Sequence generation process insufficiently described.
Allocation concealment (selection bias)	Unclear risk	Unable to judge whether participants and/or investigators could possible foresee assignment.
Blinding of participants and personnel (performance bias) All outcomes	High risk	Participants were not blinded.
Blinding of outcome assessment (detection bias) All outcomes	High risk	Participants were not blinded whereas outcomes are self‐reported.
Incomplete outcome data (attrition bias) All outcomes	Low risk	89 of the 100 randomised participants included in the analysis (89%). Reasons not described nor whether missing was at random. However loss to follow‐up is below our pre‐defined cut‐off point.
Selective reporting (reporting bias)	Unclear risk	No trial registration or no study protocol reported, nor did we fine one online
Other bias	Unclear risk	Unit of analysis error (i.e. when a study ignored the clustering of the data in their analysis). Compliance not reported.

**Table:** Kharatzadeh 2020

***Study characteristics***		
Methods	**Study design:** randomised controlled trial **Study grouping:** parallel group	
Participants	**Baseline characteristics** Emotion regulation training *Age (mean ± SD)*: NR *Sex (N (% female))*: NR *Sample size*: 30 *Years of experience (mean ± SD)*: NR Control (no intervention) *Age (mean ± SD)*: NR *Sex (N (% female))*: NR *Sample size*: 30 *Years of experience (mean ± SD)*: NR Overall *Age (mean ± SD)*: NR *Sex (N (% female))*: NR *Sample size*: 60 *Years of experience (mean ± SD)*: NR **Included criteria:** employment in intensive or critical care units, no previous participation in an ERT program and not currently taking psychotropic medication or other unprescribed substances. **Excluded criteria:** NR **Pretreatment:** at baseline, an independent sample t‐test showed no significant difference between the two groups in terms of age and working hours per month. The two groups also did not differ in sex, nor marital status. There were no between‐group differences in CERQ, DASS‐21, and ProQoL‐5 subscale scores at baseline. The statistical analysis controlled for baseline scores for CERQ, DASS‐21 and ProQoL‐5 scores as confounders. **Compliance rate:** four of the 30 (13%) participants randomised to the intervention group missed more than 2 sessions **Response rate:** nine of the 71 (13%) eligible participants did not want to participate **Type of healthcare worker:** nurses	
Interventions	**Intervention characteristics** Emotion regulation training *Type of the intervention*: Intervention type 1 ‐ to focus one’s attention on the experience of stress *Description of the intervention*: The ERT program was based on Gross'(1998) emotion regulation model, which identifies five points in the emotion generative process. The five points are situation selection, situation modification, attentional deployment, cognitive change,and response modification *The number of sessions*: six sessions *Duration of each session on average*: 2 hours *Duration of the entire intervention*: NR *Duration of the entire intervention short vs long*: short *Intervention deliverer*: Clinical psychologist *Intervention form*: NR Control (no intervention) *Type of the intervention*: NA *Description of the intervention*: NA *The number of sessions*: NA *Duration of each session on average*: NA *Duration of the entire intervention*: NA *Duration of the entire intervention short vs long*: NA *Intervention deliverer*: NA *Intervention form*: NA	
Outcomes	*Professional Quality of Life (ProQol) ‐ Burn out* **Outcome type**: ContinuousOutcome *DASS ‐ depression* **Outcome type**: ContinuousOutcome *DASS ‐ Anxiety* **Outcome type**: ContinuousOutcome *DASS‐ Stress* **Outcome type**: ContinuousOutcome	
Identification	**Sponsorship source:** NR **Country:** Iran **Setting:** Hospital **Comments:** NR **Authors name:** Hamid Kharatzadeh BSc, MSc, PhD Candidate **Institution:** Department of Clinical Psychology, Faculty ofHuman Sciences, Shahed University, Tehran, Iran **Email:** m_alavi@nm.mui.ac.ir **Address:** Mousa Alavi, Nursing and Midwifery CareResearch Center, Faculty of Nursing andMidwifery, Isfahan University of MedicalSciences, Isfahan, Iran **Time period:** 2018‐2019	
Notes	DASS‐stress included in analysis 1.1 DASS ‐ depression included in analysis 1.4	
***Risk of bias***		
**Bias**	**Authors' judgement**	**Support for judgement**
Random sequence generation (selection bias)	Low risk	Quote: "randomly allocated to either the treatment group" Using a computer‐based randomization allocation
Allocation concealment (selection bias)	Unclear risk	Quote: "using a computer‐based randomiza‐ tion allocation (refer to the study CONSORT diagram, Figure 1)." Insufficient information to understand whether intervention allocations could have been foreseen in advance of, during, enrolment.
Blinding of participants and personnel (performance bias) All outcomes	High risk	Participants were not blinded.
Blinding of outcome assessment (detection bias) All outcomes	High risk	Participants were not blinded whereas outcomes are self‐reported.
Incomplete outcome data (attrition bias) All outcomes	Low risk	Follow‐up lost less then 20% (53 of the 60 randomised participants included in the analysis).
Selective reporting (reporting bias)	Low risk	IRCT20171005036572N3. No indication of selective outcome reporting
Other bias	Low risk	No indication of other sources of bias

**Table:** Kim 2016

***Study characteristics***		
Methods	**Study design:** randomised controlled trial **Study grouping:** parallel group	
Participants	**Baseline Characteristics** Repetitive transcranial magnetic stimulation (rTMS) *Age (mean ± SD)*: 28 ± 3 *Sex (N (% female))*: 12 (50%) *Sample size*: 12 *Years of experience (mean ± SD)*: NR Control (placebo) *Age (mean ± SD)*: 32 ± 7 *Sex (N (% female))*: 12 (50%) *Sample size*: 12 *Years of experience (mean ± SD)*: NR Overall *Age (mean ± SD)*: NR *Sex (N (% female))*: 28 (100%) *Sample size*: 24 *Years of experience (mean ± SD)*: NR **Included criteria:** NR **Excluded criteria:** individuals with 1) past or current diagnosis of any axis I psychiatric disorder based on the Structured Clinical Interview for DSM‐IV‐TR Axis I Disorders, Patient Edition (SCID‐I/P),19 2) severe medical illness, 3) organic mental disorder, seizure disorder, or mental retardation, 4) pregnancy, 5) current psychotropic medication use, 6) surgical treatment of intracranial lesions, or 7) a magnetic substance in their brain or orbital area. **Pretreatment:** at baseline, there were no significant differences between the intervention group and the control group with regard to age, duration of employment, working hours per week, marital status, occupation, socio‐economic status, all **Compliance rate:** among the 28 enroled participants, data from four participants were dropped because they did not complete the TMS sessions or QEEG assessment: one participant from the active‐TMS group and one participant from the sham‐TMS group discontinued the TMS sessions due to headache and one participant from the active‐TMS group and one participant from the sham‐TMS group missed their QEEG appointments without giving notification. Ultimately, 24 participants completed all TMS sessions and QEEG assessments **Response rate:** NR **Type of healthcare worker:** various HCWs	
Interventions	**Intervention characteristics** Repetitive transcranial magnetic stimulation (rTMS) *Type of the intervention*: Intervention type 2 ‐ to focus one’s attention away from the experience of stress *Description of the intervention* : Repetitive transcranial magnetic stimulation (rTMS) is a therapeutic technique for applying stimulation to the cerebral cortex in a non‐invasive manner *The number of sessions*: 12 *Duration of each session on average*: NR *Duration of the entire intervention*: 4 weeks *Duration of the entire intervention short vs long*: short *Intervention deliverer*: NR *Intervention form*: individual Control (sham TMS) *Type of the intervention*: NA *Description of the intervention*: NA *The number of sessions*: NA *Duration of each session on average*: NA *Duration of the entire intervention*: NA *Duration of the entire intervention short vs long*: NA *Intervention deliverer*: NA *Intervention form*: NA	
Outcomes	*Psychological strain* **Outcome type**: ContinuousOutcome *Beck’s depression inventory* **Outcome type**: ContinuousOutcome *Beck’s anxiety inventory* **Outcome type**: ContinuousOutcome	
Identification	**Sponsorship source:** NR **Country:** South‐Korea **Setting:** Hospital **Comments:** NR **Authors name:** Young In Kim **Institution:** Department of Psychiatry, Chung‐Ang University Hospital, Seoul, Republic of Korea **Email:** sunmikim706@gmail.com **Address:** Sun Mi Kim, MD, PhD Department of Psychiatry, Chung‐Ang University Hospital, 102 Heukseok‐ro, Dongjak‐gu, Seoul 06973, Republic of Korea Tel: +82‐2‐6299‐1519, Fax: +82‐2‐6298‐1508 **Time period:** NR	
Notes	PSY included in analysis 2.1 Beck’s depression inventory included in analysis 2.3	
***Risk of bias***		
**Bias**	**Authors' judgement**	**Support for judgement**
Random sequence generation (selection bias)	Unclear risk	Quote: "Participants were randomly divided into two groups: the active‐TMS group and the sham‐TMS group. " Sequence generation process insufficiently described
Allocation concealment (selection bias)	Unclear risk	Difficult to judge whether participants and/or investigators could possibly foresee assignment
Blinding of participants and personnel (performance bias) All outcomes	Low risk	Participants were randomly divided into two groups: the active‐TMS group and the sham‐TMS group.
Blinding of outcome assessment (detection bias) All outcomes	Low risk	Participants were blinded and outcomes are self‐reported.
Incomplete outcome data (attrition bias) All outcomes	Low risk	No intention to treat analysis. Among the 28 enroled participants, data from four participants were dropped because they did not complete the TMS sessions or QEEG assessment (14%): one participant from the active‐TMS group and one participant from the sham‐TMS group discontinued the TMS sessions due to headache and one participant from the active‐TMS group and one participant from the sham‐TMS group missed their QEEG appointments without giving notification. Ultimately, 24 participants completed all TMS sessions and QEEG assessments. Missing not at random however below our pre‐defined cut‐off value.
Selective reporting (reporting bias)	Unclear risk	No trial registration or no study protocol reported, nor did we find one online. No indication of selective reporting.
Other bias	Unclear risk	Not able to assess response rate.

**Table:** Kline 2020

***Study characteristics***		
Methods	**Study design:** randomised controlled trial **Study grouping:** parallel group	
Participants	**Baseline characteristics** Dog *Age (mean ± SD)*: 31 ± 7.1 *Sex (N (% female))*: 20(50) *Sample size*: 43 *Years of experience (mean ± SD)*: NR Coloring *Age (mean ± SD)*: 32 ± 7.3 *Sex (N (% female))*: 20(50) *Sample size*: 39 *Years of experience (mean ± SD)*: NR Control (no intervention) *Age (mean ± SD)*: 33 ± 7.2 *Sex (N (% female))*: 18(49) *Sample size*: 40 *Years of experience (mean ± SD)*: NR Overall *Age (mean ± SD)*: NR *Sex (N (% female))*: NR *Sample size*: 122 *Years of experience (mean ± SD)*: NR **Included criteria:** emergency care providers, including nurses, residents, and physicians on duty in the ED of Eskenazi hospital **Excluded criteria:** provider statement of dislike, allergy, fear or other reason to not interact with a therapy dog, and prior enrolment **Pretreatment:** no significant differences in age and gender between the two experimental groups and the control group. Authors did not report any other results. **Compliance rate:** 127 eligible and 122 participated in at least 50% of the intervention thus > 96% **Response rate:** NR **Type of healthcare worker:** nurse, physician resident	
Interventions	**Intervention characteristics** Dog *Type of the intervention*: Intervention type 2 ‐ to focus one’s attention away from the experience of stress *Description of the intervention*: Being in a room with a dog, participants were allowed to touch the dog *The number of sessions*: 1 *Duration of each session on average*: 5 minutes *Duration of the entire intervention*: beginning of the shift until the end of the shift *Duration of the entire intervention short vs long*: short *Intervention deliverer*: NR *Intervention form*: individual Colouring *Type of the intervention*: Intervention type 2 ‐ to focus one’s attention away from the experience of stress *Description of the intervention*: Colouring mandalas in a room *The number of sessions*: 1 *Duration of each session on average*: 5 minutes *Duration of the entire intervention*: beginning of the shift until the end of the shift *Duration of the entire intervention short vs long*: short *Intervention deliverer*: NR *Intervention form*: individual Control (no intervention) *Type of the intervention*: NA *Description of the intervention*: NA *The number of sessions*: NA *Duration of each session on average*: NA *Duration of the entire intervention*: NA *Duration of the entire intervention short vs long*: NA *Intervention deliverer*: NA *Intervention form*:	
Outcomes	*Perceived Stress Scale (PSS)* **Outcome type**: ContinuousOutcome *Stress (VAS)* **Outcome type**: ContinuousOutcome	
Identification	**Sponsorship source:** NR **Country:** USA **Setting:** Hospital **Comments:** NR **Authors name:** Jeffrey A Kline **Institution:** Indiana University School of Medicine, Indianapolis **Email:** jefkline@iupui.edu **Address:** Indiana University School of Medicine, Indianapolis **Time period:** 2018	
Notes	Author J Kline kindly provided the mean and SD of the primary outcome for the intervention groups. Modified PSS included in analysis 2.1. Intervention groups combined to create a single pair‐wise comparison	
***Risk of bias***		
**Bias**	**Authors' judgement**	**Support for judgement**
Random sequence generation (selection bias)	Unclear risk	Quote: "were randomized by preprinted random sequence to receive either exposure to a therapy dog or to coloring a mandala."
Allocation concealment (selection bias)	Unclear risk	Insufficient information to understand whether intervention allocations could have been foreseen in advance of, during, enrolment.
Blinding of participants and personnel (performance bias) All outcomes	High risk	Participants were not blinded.
Blinding of outcome assessment (detection bias) All outcomes	High risk	Participants were not blinded whereas outcomes are self‐reported.
Incomplete outcome data (attrition bias) All outcomes	Low risk	No loss to follow‐up
Selective reporting (reporting bias)	Low risk	Trial registration NCT03628820. No selective outcome reporting.
Other bias	Unclear risk	Response rate not reported.

**Table:** Kurebayashi 2012

***Study characteristics***		
Methods	RCT, Brazil	
Participants	75 nurses at the Teaching Hospital of the University of São Paulo. Quote: "In order to define the sample of participants, the authors used the Stress Inventory or Stress Symptoms List – SSL. This instrument was applied to all subjects who agreed to participate in the study (N = 109); however, only subjects who achieved mean (29 to 60 points), high (61 to 120 points) or very high (>120 points) scores were included in the sample; 75 of them completed the study. As for the distribution of the participants, 22 subjects were placed in the Control Group, 27 in the Needles Group and 26 in the Seeds Group." (p. 88) Quote: "The inclusion criteria were: belonging to the nursing team; voluntary participation in the study with availability to attend the sessions; obtaining a minimum SSL score at mean, high and very high stress level; not being pregnant. The authors excluded from the sample all the subjects who went on vacation or medical leave after the beginning of the study; did not show up to the session or gave up due to adverse effects, and those who had low SSL score." (p. 88)	
Interventions	1) Experimental 1: Auriculotherapy (form of acupuncture performed on the ears) with needles (n = 27) 2) Experimental 2: Auriculotherapy with seeds (n = 26) 3) Control: No intervention(n = 22) "The intervention groups received eight sessions (one session a week), with duration of 5 to 10 minutes each session, on the Shenmen, Kidney and Brainstem points. The first two points have calmative properties and the kidney point has energetic function. After the location of the reactive points with a point locator, the ear auricle was hygienized with cotton and ethyl alcohol 70% and, then, semi‐permanent needles were applied or seeds were fixed with adhesive plaster, according to the intervention group. In the group of auriculo therapy with seeds, mustard seeds were used, and the participants were instructed to stimulate them three times a day, for 15 times, with moderate pressure. The volunteers were instructed to remove the needles or seeds 24 hours before the session and, in case there was any discomfort, itching or signs of allergy, they should remove them before that." (p. 88)	
Outcomes	Stress Symptoms List	
Identification		
Notes	SSl included in analysis 2.1. Intervention groups combined to create a single pair‐wise comparison.	
***Risk of bias***		
**Bias**	**Authors' judgement**	**Support for judgement**
Random sequence generation (selection bias)	Unclear risk	"This randomized controlled clinical experiment was performed with three groups..." (p. 88)
Allocation concealment (selection bias)	Unclear risk	Not reported
Blinding of participants and personnel (performance bias) All outcomes	High risk	Participants were not blinded.
Blinding of outcome assessment (detection bias) All outcomes	High risk	Participants were not blinded whereas outcomes are self‐reported.
Incomplete outcome data (attrition bias) All outcomes	High risk	"...there was a loss of 34 subjects during the study. Seven professionals went on vacation after the beginning of the study and two on medical leave; 12 missed the session because they had forgotten it, due to traffic problems or the difficulty to reschedule it and seven did not show up for the first session. One participant gave up due to adverse effects, in this case, nightmares, and five exclusions were due to low score (1), not belonging to the nursing team (3), and not filling out properly the questionnaires (1)." (p. 89) The authors do not report how the dropouts were distributed among the study groups.
Selective reporting (reporting bias)	High risk	The authors present data separately for participants who had high SSL scores to begin with in table 2 but not at all for participants with a moderate SSL score.
Other bias	Low risk	We did not find any indications of other sources of bias.

**Table:** Kurebayashi 2014

***Study characteristics***		
Methods	**Study design:** randomised controlled trial **Study grouping:** parallel group	
Participants	**Baseline characteristics** Auriculotherapy (protocol group) *Age (mean ± SD)*: NR *Sex (N (% female))*: NR *Sample size*: 58 *Years of experience (mean ± SD)*: NR Auriculotherapy (no protocol group) *Age (mean ± SD)*: NR *Sex (N (% female))*: NR *Sample size*: 59 *Years of experience (mean ± SD)*: NR Control (no intervention) *Age (mean ± SD)*: NR *Sex (N (% female))*: NR *Sample size*: 58 *Years of experience (mean ± SD)*: NR Overall *Age (mean ± SD)*: 34.0 ± 7.9 *Sex (N (% female))*: NR *Sample size* : 175 *Years of experience (mean ± SD)*: NR **Included criteria:** voluntary participation in the study with the availability of time to attend the sessions, and obtaining a score in the SSL indicating a medium or high level of stress. **Excluded criteria:** nephrolithiasis with surgical indication (the Kidney point can stimulate the expulsion of stones), performing another energy therapy, taking anxiolytic or anti‐depressant medication, or being pregnant. **Pretreatment:** NR **Compliance rate:** NR **Response rate:** 175 of the 213 (82%) eligible nurses participated **Type of healthcare worker:** nurses	
Interventions	**Intervention characteristics** Auriculotherapy (protocol group) *Type of the intervention*: Intervention type 2 ‐ to focus one’s attention away from the experience of stress *Description of the intervention*: The Shen Men, Brainstem, Kidney, Liver, Liver Yang 1 and 2 points were used. The Shen Men and Brainstem points have calming properties, the Kidney point has an energy function, and the Liver Yang 1 and 2 points have the function of containing the rise of liver yang. *The number of sessions*: 12 sessions *Duration of each session on average*: 5‐10 minutes *Duration of the entire intervention*: 30 days *Duration of the entire intervention short vs long*: short *Intervention deliverer*: acupuncturists nurses and an acupuncturist psychologist *Intervention form*: Individual Auriculotherapy (no protocol group) *Type of the intervention*: Intervention type 2 ‐ to focus one’s attention away from the experience of stress *Description of the intervention*: The Shen Men, Brainstem, Kidney, Liver, Liver Yang 1 and 2 points were used. The Shen Men and Brainstem points have calming properties, the Kidney point has an energy function, and the Liver Yang 1 and 2 points have the function of containing the rise of liver yang. The points were chosen dependent on the reported symptoms at each consultation, according to the Traditional Chinese Medicine diagnoses found. *The number of sessions*: 12 sessions *Duration of each session on average*: 5 to 10 minutes *Duration of the entire intervention*: 30 days *Duration of the entire intervention short vs long*: short *Intervention deliverer*: acupuncturists nurses and an acupuncturist psychologist *Intervention form*: Individual Control (no intervention) *Type of the intervention*: NA *Description of the intervention*: NA *The number of sessions*: NA *Duration of each session on average*: NA *Duration of the entire intervention*: NA *Duration of the entire intervention short vs long*: NA *Intervention deliverer*: NA *Intervention form*: NA	
Outcomes	*Vasconcelos’ Stress Symptoms List (SSL)* **Outcome type**: ContinuousOutcome	
Identification	**Sponsorship source:** NR **Country:** Brasil **Setting:** Hospital **Comments:** NR **Authors name:** Leonice Fumiko Sato Kurebayashi **Institution:** Doctoral student, Escola de Enfermagem, Universidade de São Paulo, São Paulo, SP, Brazil. **Email:** juliaps@usp.br **Address:** Maria Júlia Paes da Silva Universidade de São Paulo. Escola de Enfermagem Av. Dr. Enéas de Carvalho Aguiar, 409Bairro: Cerqueira César CEP: 05409‐000, São Paulo, SP, Brasil **Time period:** 2011	
Notes	SSL included in analysis 2.1. Intervention groups combined to create a single pair‐wise comparison	
***Risk of bias***		
**Bias**	**Authors' judgement**	**Support for judgement**
Random sequence generation (selection bias)	Low risk	Quote: "using numbers randomly generated by the site www.randomizer.org"
Allocation concealment (selection bias)	Unclear risk	Difficult to judge whether participants and/or investigators could possibly foresee assignment. However, it is assumed that randomisation was performed in one go and that participants and/or investigators could not foresee assignment.
Blinding of participants and personnel (performance bias) All outcomes	High risk	Participants were not blinded.
Blinding of outcome assessment (detection bias) All outcomes	High risk	Participants were not blinded whereas outcomes are self‐reported.
Incomplete outcome data (attrition bias) All outcomes	Low risk	No loss to follow‐up.
Selective reporting (reporting bias)	Low risk	Protocol registration checked, no selective reporting.
Other bias	Unclear risk	'Loosely' validated outcome measure. Compliance rate not reported.

**Table:** Leao 2017

***Study characteristics***		
Methods	**Study design:** randomised controlled trial **Study grouping:** parallel group	
Participants	**Baseline characteristics** Mono‐sensory *Age (mean ± SD)*: 36.4 ± 8.2 *Sex (N (% female))*: NR *Sample size*: 25 *Years of experience (mean ± SD)*: NR Bisensory *Age (mean ± SD)*: 41.0 ± 9.2 *Sex (N (% female))*: NR *Sample size*: 26 *Years of experience (mean ± SD)*: NR Multisensory *Age (mean ± SD)*: 35.5 ± 8.9 *Sex (N (% female))*: NR *Sample size*: 23 *Years of experience (mean ± SD)*: NR Control (no intervention) *Age (mean ± SD)*: 38.2 ± 10.2 *Sex (N (% female))*: NR *Sample size*: 19 *Years of experience (mean ± SD)*: NR Overall *Age (mean ± SD)*: NR *Sex (N (% female))*: NR *Sample size*: 98 *Years of experience (mean ± SD)*: NR **Included criteria:** female health professionals aged between 18 and 60 years old who worked in healthcare or healthcare‐related areas **Excluded criteria:** relevant dermatological findings, women who worked at night or alternating shifts (due to the known chronobiological changes that could affect the outcomes in this study), as well as women who were pregnant or lactating. **Pretreatment:** reported socio‐demographic baseline characteristics of participants randomised to the intervention group were similar to socio‐demographic baseline characteristics of participants randomised to the control group. **Compliance rate:** we were surprised by the lack of time and availability of participants to take part in the study. Although many participants were interested in participating during the recruitment stage, they showed poor compliance and did not attend subsequent evaluations, especially those scheduled for 15 days and 30 days following the study (follow‐up). **Response rate:** of the 374 eligible participants 123 were randomised (33%) **Type of healthcare worker:** nurses	
Interventions	**Intervention characteristics** Mono‐sensory *Type of the intervention*: Intervention type 2 ‐ to focus one’s attention away from the experience of stress *Description of the intervention*: The proposed intervention consisted of daily body moisturizing *The number of sessions*: NR *Duration of each session on average*: NR *Duration of the entire intervention*: 30 days *Duration of the entire intervention short vs long*: short *Intervention deliverer*: NR *Intervention form*: Individual Bisensory *Type of the intervention*: Intervention type 2 ‐ to focus one’s attention away from the experience of stress *Description of the intervention*: The proposed intervention consisted of daily body moisturising *The number of sessions*: NR *Duration of each session on average*: NR *Duration of the entire intervention*: 30 days *Duration of the entire intervention short vs long*: short *Intervention deliverer*: NR *Intervention form*: Individual Multisensory *Type of the intervention*: Intervention type 2 ‐ to focus one’s attention away from the experience of stress *Description of the intervention*: The proposed intervention consisted of daily body moisturising *The number of sessions*: NR *Duration of each session on average*: NR *Duration of the entire intervention*: 30 days *Duration of the entire intervention short vs long*: short *Intervention deliverer*: NR *Intervention form*: Individual Control (no intervention) *Type of the intervention*: NA *Description of the intervention*: NA *The number of sessions*: NA *Duration of each session on average*: NA *Duration of the entire intervention*: NA *Duration of the entire intervention short vs long*: NA *Intervention deliverer*: NA *Intervention form*: NA	
Outcomes	*The ISS Inventory of Symptoms of Stress* **Outcome type**: DichotomousOutcome	
Identification	**Sponsorship source:** Hospital Isrealita Albert Einstein **Country:** Brasil **Setting:** Private hospital **Comments:** NR **Authors name:** Eliseth Ribeiro Leão **Institution:** Research Institute, Hospital Israelita Albert Einstein. São Paulo, Brazil **Email:** eliseth.leao@einstein.b **Address:** NR **Time period:** 2014	
Notes	Dichotomous outcome measure that we could not enter into the meta‐analysis	
***Risk of bias***		
**Bias**	**Authors' judgement**	**Support for judgement**
Random sequence generation (selection bias)	Low risk	Quote: "signed an informed consent form. Then, we randomized participants and conducted a pilot test with seven volunteers to evaluate any necessary adjustments to the study (1 control participant, and two participants for each of the intervention groups). Participants were randomized using opaque, sealed and numbered envelopes and then ran‐ domly selected using a computer program with numbers generated by the Research Randomizer1 in order to distribute participants among the four arms of the study:
Allocation concealment (selection bias)	Unclear risk	Participants were randomized using opaque, sealed and numbered envelopes and then randomly selected using a computer program with numbers generated by the Research Randomizer in order to distribute participants among the four arms of the study. The enrolment occurred continuously from July to October, 2014. The follow‐up of each participant was always performed 30 days after the end of the intervention. The study was conducted at a single center. Data collection was completed in December 2014. Difficult to judge whether participants and/or investigators could possibly foresee assignment.
Blinding of participants and personnel (performance bias) All outcomes	High risk	Participants were not blinded.
Blinding of outcome assessment (detection bias) All outcomes	High risk	Participants were not blinded whereas outcomes were self‐report.
Incomplete outcome data (attrition bias) All outcomes	High risk	30 of the randomised participants were not included in the analysis (30/123 24%). Reasons provided. Not reported whether missing at random.
Selective reporting (reporting bias)	Low risk	Trial registration NCT02406755. No indication of selective reporting.
Other bias	Unclear risk	251/374 (67%) did not want to participate. Low compliance ‐> We were surprised by the lack of time and availability of participants to take part in the study. Although a large number of participants were interested in participating during the recruitment stage, they showed poor compliance and did not attend subsequent evaluations, especially those scheduled for 15 days and 30 days following the study (follow‐up).

**Table:** Lebares 2021

***Study characteristics***		
Methods	**Study design:** randomised controlled trial **Study grouping:** parallel group	
Participants	**Baseline Characteristics** Enhanced stress resilience training‐1 (ESRT‐1) *Age (mean ± SD)*: 27 ± 2 *Sex (N (% female))*: 14 (64%) *Sample size*: 22 *Years of experience (mean ± SD)*: NR Control‐1 (no intervention) *Age (mean ± SD)*: 29 ± 2.8 *Sex (N (% female))*: 6 (18%) *Sample size*: 18 *Years of experience (mean ± SD)*: NR Enhanced stress resilience training‐2 (ESRT‐2) *Age (mean ± SD)*: 29 ± 2.4 *Sex (N (% female))*: 11 (50%) *Sample size*: 22 *Years of experience (mean ± SD)*: NR Control‐2 (no intervention) *Age (mean ± SD)*: 29 ± 2.2 *Sex (N (% female))*: 9 (43%) *Sample size*: 21 *Years of experience (mean ± SD)*: NR Overall *Age (mean ± SD)*: NR *Sex (N (% female))*: NR *Sample size*: NR *Years of experience (mean ± SD)*: NR **Included criteria:** NR **Excluded criteria:** NR **Pretreatment:** reported socio‐demographic baseline characteristics of participants randomised to the intervention group were similar to socio‐demographic baseline characteristics of participants randomised to the control group **Compliance rate:** 89 assigned to intervention 50% actually participated **Response rate:** NR **Type of healthcare worker:** post‐graduate residents and surgeons	
Interventions	**Intervention characteristics** Enhanced stress resilience training‐1 (ESRT‐1) *Type of the intervention*: 2. Intervention type 2 ‐ to focus one’s attention away from the experience of stress *Description of the intervention*: Focused on the development of mindfulness meditation skills using culturally acceptable language. *The number of sessions*: 8 *Duration of each session on average*: 2 hours *Duration of the entire intervention*: 8 *Duration of the entire intervention short vs long*: Short *Intervention deliverer*: MSBR instructor with > 10 years experience *Intervention form*: NR Control‐1 (no intervention) *Type of the intervention*: NA *Description of the intervention*: NA *The number of sessions*: NA *Duration of each session on average*: NA *Duration of the entire intervention*: NA *Duration of the entire intervention short vs long*: NA *Intervention deliverer*: NA *Intervention form*: NA Enhanced stress resilience training‐2 (ESRT‐2) *Type of the intervention*: 2. Intervention type 2 ‐ to focus one’s attention away from the experience of stress *Description of the intervention*: focused on the development of mindfulness meditation skills using culturally acceptable language, explicitly applied to surgery, hospital‐based work, and challenges of maintaining well‐being during demanding training. *The number of sessions*: 6 *Duration of each session on average*: 1.5 hours *Duration of the entire intervention*: 6 *Duration of the entire intervention short vs long*: Short *Intervention deliverer*: MSBR instructor with > 10 years experience *Intervention form*: NR Control‐2 (no intervention) *Type of the intervention*: NA *Description of the intervention*: NA *The number of sessions*: NA *Duration of each session on average*: NA *Duration of the entire intervention*: NA *Duration of the entire intervention short vs long*: NA *Intervention deliverer*: NA *Intervention form*: NA	
Outcomes	*Cohen's Perceived Stress Scale (PSS‐10)* **Outcome type**: ContinuousOutcome *Maslach Burnout Inventory ‐ emotional exhaustion* **Outcome type**: ContinuousOutcome *Maslach Burnout Inventory ‐ Depersonalisation* **Outcome type**: ContinuousOutcome	
Identification	**Sponsorship source:** NR **Country:** USA **Setting:** Hospital **Comments:** NR **Authors name:** Carter C. Lebares **Institution:** Department of Surgery, University of California, San Francisco **Email:** carter.Lebares@ucsf.edu **Address:** NR **Time period:** 2016‐2017	
Notes	PSS included in analysis 2.1 and 2.2	
***Risk of bias***		
**Bias**	**Authors' judgement**	**Support for judgement**
Random sequence generation (selection bias)	Low risk	Quote: "In 2016 and 2017, we conducted a partially‐blinded, pilot, parallel group randomized trial (NCT#03141190) with 1:1 allocation of PGY‐1 residents to ESRT‐1 (n ¼ 23) versus active Control‐1 (n ¼ 21). ESRT‐1 participants (100% surgical) were" Computer‐generated randomization
Allocation concealment (selection bias)	Unclear risk	One male control mistakenly attended the first week of ESRT‐2 and was allowed to continue in the intervention arm
Blinding of participants and personnel (performance bias) All outcomes	Unclear risk	Although participants were blinded to assignment and asked not to discuss class content between arms, communication was certainly possible and should be considered in evaluating our results.
Blinding of outcome assessment (detection bias) All outcomes	Unclear risk	Although participants were blinded to the assignment and asked not to discuss class content between arms, communication was certainly possible and should be considered in evaluating our results. Outcomes are self‐report.
Incomplete outcome data (attrition bias) All outcomes	Low risk	Total absences were minimal (10%) across all trials, populations, and conditions; 80% were attributable to scheduled vacations or emergent patient situations. The remainder were attributable to over‐sleeping. Attrition was limited to two female participants (both nonsurgical), who dropped from ESRT‐2 (intervention arm) due to disinterest in performing home practice and feeling ‘‘overloaded’’ by additional obligations.Participants who did not complete the study felt more overloaded however loss to follow‐up is below our pre‐defined cut‐off point.
Selective reporting (reporting bias)	Low risk	Trial registration NCT03141190NCT03518359. No indication of selective reporting.
Other bias	Low risk	No indication of other sources of bias.

**Table:** Lee 1994

***Study characteristics***		
Methods	Randomised controlled trial, Taiwan	
Participants	60 hospital nurses suffering from either: insomnia, headache or gastrointestinal discomfort.	
Interventions	1) Experimental: assertiveness training: six 2‐hour sessions on Monday, Wednesday and Friday at 2pm to 4pm on two consecutive weeks. The contents of sessions included the concept of beliefs and negative self‐statements, building a positive belief system, applying assertion to clinical settings and developing group and self‐reinforcement support systems. 2) Control: Traditional in‐service programme about computer applications in nursing.	
Outcomes	Perceived Stress Scale, Rathus Assertiveness Schedule	
Identification		
Notes	PSS included in analysis 1.1	
***Risk of bias***		
**Bias**	**Authors' judgement**	**Support for judgement**
Random sequence generation (selection bias)	Unclear risk	"Subjects were randomly assigned to one of two treatments: assertiveness training (AT) or alternate treatment control (ATC), which served as a control and contained updated knowledge of new computer technology for in patient settings." (p. 419)
Allocation concealment (selection bias)	Unclear risk	"Subjects admitted to the study agreed to random treatment assignment and a 2‐month commitment to the study. However, the subjects did not know whether they would receive treatment or control procedures during that time." (p. 425) Difficult to judge whether participants and/or investigators could possibly foresee assignment.
Blinding of participants and personnel (performance bias) All outcomes	High risk	Participants not blinded.
Blinding of outcome assessment (detection bias) All outcomes	High risk	Participants were not blinded whereas outcomes are self‐reported.
Incomplete outcome data (attrition bias) All outcomes	Low risk	"Of the respondents who initially chose to participate, three did not complete the study and were not included in the data analysis due to their failure to attend all sessions, failure to complete the questionnaire, or decision to leave hospital employment." (p. 425) Loss to follow‐up is below our review pre‐defined cut‐off point (three of the 60 (0.05%))
Selective reporting (reporting bias)	Low risk	All outcomes reported.
Other bias	Low risk	We did not find any indications of other sources of bias.

**Table:** Lee 2020

***Study characteristics***		
Methods	**Study design:** randomised controlled trial **Study grouping:** parallel group	
Participants	**Baseline characteristics** Psychotherapy *Age in years (mean ± SD)*: NR *Sex (N (% female))*: 37 (71.1%) *Sample size*: 52 *Years of experience (mean ± SD)*: NR Control (no intervention) *Age in years (mean ± SD)*: NR *Sex (N (% female))*: 41 (68.3%) *Sample size*: 60 *Years of experience (mean ± SD)*: NR Overall *Age in years (mean ± SD)*: NR *Sex (N (% female))*: 78 (69.6%) *Sample size*: 112 *Years of experience (mean ± SD)*: NR **Included criteria:** the participant must be a practising physician of the primary healthcare system in Almaty (Kazakhstan), he/she should have at least one year of work experience; he/she must provide signed informed consent. **Excluded criteria:** incomplete filling out of questionnaires, refusal to participate in the study, lack of informed consent, dismissal from work. **Pretreatment:** according to the results, there was no statistically significant difference between age, gender, marital status and speciality of doctors in the two groups. **Type of healthcare worker:** exclusively doctors involved in primary healthcare **Response rate:** 93% **Compliance rate:** 23%	
Interventions	**Intervention characteristics** Psychotherapy *Type of the intervention*: Intervention type 1 ‐ to focus one’s attention on the experience of stress *Description of the intervention*: Short‐term psychotherapy based on the coping strategy (Asimov method): 1st step – differentiation and self‐knowledge; 2nd step – awareness of the state through induced images; 3rd step – awareness of the state through spontaneous images with closed eyes; 4th step – awareness of the state through spontaneous images with open eyes; 5th step – environmentally friendly behaviour. *The number of sessions*: 9 to 12 times a month *Duration of each session on average*: 50 minutes *Duration of the entire intervention*: 4 weeks *Duration of the entire intervention short vs long*: Short *Intervention deliverer*: NR *Intervention form*: Individual, video communication Control (no intervention) *Type of the intervention*: NA *Description of the intervention*: NA *The number of sessions*: NA *Duration of each session on average*: NA *Duration of the entire intervention*: NA *Duration of the entire intervention short vs long*: NA *Intervention deliverer*: NA *Intervention form*: NA	
Outcomes	*Maslach Burnout Inventory ‐ Emotional Exhaustion* **Outcome type**: ContinuousOutcome *Maslach Burnout Inventory ‐ Depersonalisation* **Outcome type**: ContinuousOutcome *Maslach Burnout Inventory ‐ Personal accomplishment (lack of)* **Outcome type**: ContinuousOutcome	
Identification	**Sponsorship source:** NR **Country:** Republic of Kazakhstan **Setting:** Two out‐patient‐clinics **Comments:** NR **Authors name:** Sergey Lee **Institution:** S.D. Asfendiyarov Kazakh National Medical University **Email:** lee.s.kaznmu@mail.ru **Address:** Tole Bi Street 94, Almaty, 050000, Kazakhstan **Time period:** 2019	
Notes	MBI‐EE included in analysis 1.2	
***Risk of bias***		
**Bias**	**Authors' judgement**	**Support for judgement**
Random sequence generation (selection bias)	Low risk	Participants were assigned with an individual number, after which they were randomized using online random tools (experimental and control groups into two groups):
Allocation concealment (selection bias)	Unclear risk	Insufficient information to understand whether intervention allocations could have been foreseen in advance of, during, enrolment.
Blinding of participants and personnel (performance bias) All outcomes	High risk	Participants were not blinded.
Blinding of outcome assessment (detection bias) All outcomes	High risk	Participants were not blinded whereas outcomes are self‐reported.
Incomplete outcome data (attrition bias) All outcomes	High risk	112 of the 243 randomised participants included in the analyses. Not reported whether lost to follow‐up was at random.
Selective reporting (reporting bias)	Unclear risk	No trial protocol reported, nor did we find one online.
Other bias	Low risk	No indication of other bias.

**Table:** Lee 2021

***Study characteristics***		
Methods	**Study design:** randomised controlled trial **Study grouping:** parallel group	
Participants	**Baseline characteristics** Auricular acupressure (experimental) *Age in years (N (% 20‐29), N (% 30‐39), N (% 40‐49))*: 7 (25%), 13 (46%), 8 (29%) *Sex (N (% female))*: 26 (93%) *Sample size*: 28 *Years of experience (N (% < 10), N (% 10‐19), N (% 20‐30))*: 7 (25%), 17 (61%), 4 (14%) Control (placebo acupressure) *Age in years (N (% 20‐29), N (% 30‐39), N (% 40‐49))*: 4 (15%), 16 (62%), 6 (23%) *Sex (N (% female))*: 25 (96%) *Sample size*: 26 *Years of experience (N (% < 10), N (% 10‐19), N (% 20‐30))*: 10 (39%), 12 (46%), 4 (15%) Overall *Age in years (N (% 20‐29), N (% 30‐39), N (% 40‐49))*: 11 (20%), 29 (54%), 14 (26%) *Sex (N (% female))*: 51 (94%) *Sample size* : 54 *Years of experience (N (% <10), N (% 10‐19), N (% 20‐30))*: 17 (31%) 29 (54%), 8 (15%) **Included criteria:** Outpatient nurses who were over 20 years of age and working in a medical institution with no experience of receiving auricular acupressure. **Excluded criteria:** Those who were receiving complementary or alternative therapies; those taking antidepressants, anticonvulsants, or sleeping pills; and those with skin integrity problems were excluded from the study. **Pretreatment:** No statistically significant differences emerged between the two groups on stress, anxiety, depression, and physiological index **Type of healthcare worker:** Exclusively outpatient nurses **Response rate:** 100% **Compliance rate:** 90%	
Interventions	**Intervention characteristics** Auricular acupressure (experimental) *Type of the intervention*: Intervention type 2 ‐ to focus one’s attention away from the experience of stress *Description of the intervention*: The researcher applied auricular acupressure using vaccaria seeds to one ear of each participant. The experimental group received auricular acupressure on points related to stress, anxiety, and depression (Shenmen, heart, occiput, anterior lobe). *The number of sessions*: 5 *Duration of each session on average*: 5 days (4 times a day) *Duration of the entire intervention*: 5 weeks *Duration of the entire intervention short vs long*: short *Intervention deliverer*: The researcher *Intervention form*: Individual, face‐to‐face Control (placebo acupressure) *Type of the intervention*: NA *Description of the intervention*: The researcher applied auricular acupressure using vaccaria seeds to one ear of each participant. The placebo group received auricular acupressure on points unrelated to stress, anxiety, and depression (wrist, hips, elbow, shoulder). *The number of sessions*: 5 *Duration of each session on average*: 5 days (four times a day) *Duration of the entire intervention*: 5 weeks *Duration of the entire intervention short vs long*: short *Intervention deliverer*: The researcher *Intervention form*: Individual, face‐to‐face	
Outcomes	*Perceived Stress Scale (PSS)* **Outcome type**: ContinuousOutcome **Reporting**: Fully reported *Beck Depression Inventory‐II (BDI‐II)* **Outcome type**: ContinuousOutcome **Reporting**: Fully reported *State‐Trait Anxiety Inventory (STAI)* **Outcome type**: ContinuousOutcome **Reporting**: Fully reported	
Identification	**Sponsorship source:** NR **Country:** South Korea **Setting:** A medical institution **Comments:** NR **Authors name:** Hyojung Park **Institution:** College of Nursing, Ewha Womans University **Email:** hyojungp@ewha.ac.kr **Address:** 52, Ewhayeodae‐gil, Seodaemun‐gu, Seoul 03760, South Korea **Time period:** June 2018 to August 2018	
Notes	PSS included in analysis 2.1 Beck Depression Inventory‐II (BDI‐II) included in analysis 2.3	
***Risk of bias***		
**Bias**	**Authors' judgement**	**Support for judgement**
Random sequence generation (selection bias)	Unclear risk	Quote: "Participants for the experimental group and the control group were selected through randomization.
Allocation concealment (selection bias)	Unclear risk	Insufficient information to understand whether intervention allocations could have been foreseen in advance of, during, enrolment.
Blinding of participants and personnel (performance bias) All outcomes	Low risk	Participants were blinded.
Blinding of outcome assessment (detection bias) All outcomes	Low risk	Participants were blinded and outcomes are self‐reported
Incomplete outcome data (attrition bias) All outcomes	Low risk	54 of the 60 randomised participants included in the analyses.
Selective reporting (reporting bias)	Unclear risk	No trial registration, nor did we find one online.
Other bias	Low risk	No indication of other risk of bias.

**Table:** Lin 2015

***Study characteristics***		
Methods	**Study design:** randomised controlled trial **Study grouping:** parallel group	
Participants	**Baseline characteristics** Yoga *Age in years (mean ± SD)*: 32.1 ± 7.5 *Sex (N (% female))*: 26 (86.7%) *Sample size*: 30 *Years of experience (mean ± SD)*: NR Control (no intervention) *Age in years (mean ± SD)*: 29.8 ± 6.9 *Sex (N (% female))*: 22 (73.3%) *Sample size*: 30 *Years of experience (mean ± SD)*: NR Overall *Age in years (mean ± SD)*: NR *Sex (N (% female))*: 48 (80%) *Sample size*: 60 *Years of experience (mean ± SD)*: NR **Included criteria:** the inclusion criteria consisted of mental health professionals who were not involved in a formal exercise program and who were willing to participate in this study. **Excluded criteria:** exclusion criteria included pain due to injuries to shoulders, waist, or lower back, and musculoskeletal diseases such as muscle strains, that made participants unsuitable to participate in this study. **Pretreatment:** the demographic characteristics of the two groups, including gender, marital status, religious reference, educational status, job title, and age, showed no significant differences. The total scores of pretest stress adaptation between yoga and control groups did not reach statistical significance. The total scores of pretest work‐related stress between the two groups reached statistical significance. Thus, we took the total scores of pretest work‐related stress as a covariate to control for possible confounding. **Type of healthcare worker:** mental health professionals in a teaching hospital, but 38% non‐medical nor nursing staff **Response rate:** 80% **Compliance rate:** 100%	
Interventions	**Intervention characteristics** Yoga *Type of the intervention*: Intervention type 2 ‐ to focus one’s attention away from the experience of stress *Description of the intervention*: The yoga class regularly began with slower warm‐up exercises: Abdominal breathing, cooling breath, and bellows breath, followed by forced abdominal breathing, meditation, and bodily stretching positions. *The number of sessions*: 12 *Duration of each session on average*: 60 minutes *Duration of the entire intervention*: 12 weeks *Duration of the entire intervention short vs long*: Long *Intervention deliverer*: The fidelity of the intervention was monitored and directed by two qualified teachers. *Intervention form*: NR Control (no intervention) *Type of the intervention*: NA *Description of the intervention*: A free tea time during which they watched television and did not exercise. *The number of sessions*: 12 *Duration of each session on average*: 60 minutes *Duration of the entire intervention*: 12 weeks *Duration of the entire intervention short vs long*: Long *Intervention deliverer*: NA *Intervention form*: NA	
Outcomes	*Work‐related stress scale* **Outcome type**: ContinuousOutcome **Reporting**: Fully reported *Stress adaptation scale* **Outcome type**: ContinuousOutcome **Reporting**: Fully reported	
Identification	**Sponsorship source:** Funding for this study was provided by a grant RA12042 from Changhua Show Chwan Memorial Hospital and a grant MOST‐103‐2314‐B‐166‐003 from the Minister of Science in Taiwan **Country:** Taiwan **Setting:** A teaching hospital **Comments:** NR **Authors name:** Shu‐Hui Yeh **Institution:** Central Taiwan University of Science and Technology–Nursing **Email:** yehshuhui@gmail.com ; 107514@ctust.edu.tw **Address:** No.666 Buzih Road, Beitun District, Taichung City 40601, Taiwan **Time period:** NR	
Notes	Stress adaptation scale (higher is better) included in analysis 2.1	
***Risk of bias***		
**Bias**	**Authors' judgement**	**Support for judgement**
Random sequence generation (selection bias)	Low risk	This study was a single‐blind, parallel‐arm randomized controlled trial in which the analyzer was unaware of which group was the experimental or control group. The intervention consisted of a series of weekly, 60‐minute yoga classes over a 12‐week period (Figure 1). Those who were assigned to the control group participated in a free tea time during which they watched television and did not exercise. The participants each signed an informed consent prior to enroling in the study. Then, the participants signed the informed consent form and were randomly assigned to yoga or control groups by drawing lots. There were 30 participants each in the yoga and control groups. It was expected that the two groups were homogeneous through drawing lots of random allocation.
Allocation concealment (selection bias)	High risk	Then, the participants signed the informed consent form and were randomly assigned to yoga or control groups by drawing lots. There were 30 participants each in the yoga and control groups. It was expected that the two groups were homogeneous through drawing lots of random allocation.
Blinding of participants and personnel (performance bias) All outcomes	High risk	Participants not blinded.
Blinding of outcome assessment (detection bias) All outcomes	High risk	Participants were not blinded whereas outcomes are self‐reported.
Incomplete outcome data (attrition bias) All outcomes	Low risk	No loss to follow‐up.
Selective reporting (reporting bias)	Unclear risk	No trial registration, nor did we find one online.
Other bias	Unclear risk	Statistically significant baseline differences on stress.

**Table:** Lin 2019

***Study characteristics***		
Methods	**Study design:** randomised controlled trial **Study grouping:** parallel group	
Participants	**Baseline characteristics** Mindfulness‐based group intervention *Age in years (mean ± SD)*: 32.9 ± 7.5 *Sex (N (% female))*: 43 (97.7%) *Sample size*: 44 *Years of experience (mean ± SD)*: 11.8 ± 7.5 Control (wait list) *Age in years (mean ± SD)*: 30.2 ± 6.1 *Sex (N (% female))*: 41 (89.1%) *Sample size*: 46 *Years of experience (mean ± SD)*: 9.3 ± 5.6 Overall *Age in years (mean ± SD)*: NR *Sex (N (% female))*: 84 (93.3%) *Sample size*: 90 *Years of experience (mean ± SD)*: NR **Included criteria:** being employed as a full‐time nurse **Excluded criteria:** (a) being a student nurse, (b) suffering from serious somatic disease, (c) taking mood‐regulating drugs, (d) having suffered a major traumatic event in the past 6 months, and (e) having participated in mindfulness training previously. **Pretreatment:** no significant differences were observed between the two groups for any of the demographic characteristics. **Type of healthcare worker:** exclusively nurses **Response rate:** NR **Compliance rate:** 82%	
Interventions	**Intervention characteristics** Mindfulness‐based group intervention *Type of the intervention*: Intervention type 1 ‐ to focus one’s attention on the experience of stress *Description of the intervention*: The program was a mindfulness‐based group intervention generally based on the principles and exercises of MBSR (Kabat‐Zinn, 1990) and MBCT (Teasdale et al., 2000). *The number of sessions*: 8 *Duration of each session on average*: 2‐hour weekly group sessions and 20 minutes of formal mindfulness practice at home daily *Duration of the entire intervention*: 8 weeks *Duration of the entire intervention short vs long*: Short *Intervention deliverer* : a researcher who has been practising mindfulness for two years and attended several MBSR courses, retreats, and other training activities related to mindfulness and meditation. *Intervention form*: Combination (group and individual), face‐to‐face and at home Control (wait list) *Type of the intervention*: NA *Description of the intervention*: NA *The number of sessions*: NA *Duration of each session on average*: NA *Duration of the entire intervention*: NA *Duration of the entire intervention short vs long*: NA *Intervention deliverer*: NA *Intervention form*: NA	
Outcomes	*Perceived Stress Scale (PSS)* **Outcome type**: ContinuousOutcome	
Identification	**Sponsorship source:** This study was supported by a grant from the General Program of Science and Technology Plan for Health Care in Dongguan City of Guangdong Province. The funder played no role in the study design, data collection, data analysis, manuscript preparation, or decision to publish the report. **Country:** China **Setting:** Two general hospitals **Comments:** NR **Authors name:** Guoping He **Institution:** Xiangya Nursing School of Central South University **Email:** lily453125836@126.com **Address:** No. 172, Tongzipo Road, Yuelu District, Changsha, Hunan 410013, China **Time period:** 2017	
Notes	PSS included in analysis 1.1 and 1.2	
***Risk of bias***		
**Bias**	**Authors' judgement**	**Support for judgement**
Random sequence generation (selection bias)	Low risk	Quote: "utilized a randomized controlled design.Eligible participants were randomized 1:1 using a computer‐generated random number table to the intervention group or the wait‐list control group."
Allocation concealment (selection bias)	Unclear risk	Insufficient information to understand whether intervention allocations could have been foreseen in advance of, during, enrolment.
Blinding of participants and personnel (performance bias) All outcomes	High risk	Participants were not blinded.
Blinding of outcome assessment (detection bias) All outcomes	High risk	Participants were not blinded whereas outcomes are self‐reported.
Incomplete outcome data (attrition bias) All outcomes	Low risk	No loss to follow‐up.
Selective reporting (reporting bias)	Unclear risk	No trial registration, nor did we find one online.
Other bias	Unclear risk	Response rate not reported.

**Table:** Luthar 2017

***Study characteristics***		
Methods	**Study design:** randomised controlled trial **Study grouping:** parallel group	
Participants	**Baseline characteristics** Authentic Connections Groups *Age in years (mean ± SD)*: 38.8 ± 6.1 *Sex (N (% female))*: 21 (100%) *Sample size*: 21 *Years of experience (mean ± SD)*: NR Control (no intervention) *Age in years (mean ± SD)*: 39.4 ± 4.8 *Sex (N (% female))*: 19 (100%) *Sample size*: 19 *Years of experience (mean ± SD)*: NR Overall *Age in years (mean ± SD)*: NR *Sex (N (% female))*: 40 (100%) *Sample size*: 40 *Years of experience (mean ± SD)*: NR **Included criteria:** inclusion required at least one child 18 years of age or younger. **Excluded criteria:** NR **Pretreatment:** other than the difference in proportion of NP/PAs and physicians, the intervention and control groups did not differ in demographics, baseline adjustment or cortisol levels. **Type of healthcare worker:** female (physicians, PhD’s in clinical practice, NPs, and PAs) **Response rate:** NR **Compliance rate:** 100%	
Interventions	**Intervention characteristics** Authentic Connections Groups *Type of the intervention*: Intervention type 4 ‐ combination of interventions *Description of the intervention*: The central goal underlying this intervention was to facilitate authentic, supportive relationships among mothers. ACG meetings were based in respect, empathy, and empowerment. Although there were clear topics and exercises, sessions were non‐didactic in nature, based in guided discussions and role plays. *The number of sessions*: 12 *Duration of each session on average*: 1 hour *Duration of the entire intervention*: 12 weeks *Duration of the entire intervention short vs long*: long *Intervention deliverer*: A female psychiatrist (J.E.), a skilled female group facilitator trained in the manualized procedures. *Intervention form*: Group Control (no intervention) *Type of the intervention*: NA *Description of the intervention*: Protected time to use as they chose *The number of sessions*: 12 *Duration of each session on average*: 1 hour *Duration of the entire intervention*: 12 weeks *Duration of the entire intervention short vs long*: long *Intervention deliverer*: NA *Intervention form*: NA	
Outcomes	*Maslach Burnout Inventory ‐ Emotional Exhaustion* **Outcome type**: ContinuousOutcome *Maslach Burnout Inventory ‐ Depersonalisation* **Outcome type**: ContinuousOutcome *Maslach Burnout Inventory ‐ Personal accomplishment* **Outcome type**: ContinuousOutcome *Beck Depression Inventory* **Outcome type**: ContinuousOutcome	
Identification	**Sponsorship source:** This study was supported by a Seed fund from Arizona State University to Luthar. Mayo Clinic funded and supported medical‐care professionals’ time to participate in study activities **Country:** United States **Setting:** Mayo Clinic **Comments:** NR **Authors name:** Suniya S. Luthar **Institution:** Department of Psychology, Arizona State University, Tempe, Arizona **Email:** Suniya.Luthar@asu.edu **Address:** 950 S. McAllister Drive, Tempe, AZ85281 **Time period:** February to November 2015	
Notes	MBI‐EE included in analysis 4.1 Beck Depression scale included in analysis 4.4	
***Risk of bias***		
**Bias**	**Authors' judgement**	**Support for judgement**
Random sequence generation (selection bias)	Unclear risk	Quote: "Participants were assigned randomly to the ACG intervention group (n ¼ 21) or to the control group (n ¼ 19)"
Allocation concealment (selection bias)	Unclear risk	Not mentioned.
Blinding of participants and personnel (performance bias) All outcomes	Low risk	Quote: "With blinded random assignment,"
Blinding of outcome assessment (detection bias) All outcomes	Low risk	Participants were blinded and outcomes self‐reported.
Incomplete outcome data (attrition bias) All outcomes	Low risk	Quote: "On psychological measures, one participant was missing data on parenting stress at follow‐up. On biological measures, pregnancies and maternity leaves precluded draws from one woman throughout, and from two at the follow‐up. An additional two could not schedule times to provide samples at follow‐up, and two were statistical outliers and removed from the analysis (> 2 SD from the mean)."
Selective reporting (reporting bias)	Unclear risk	No trial registration nor did we find one online.
Other bias	Unclear risk	Response rate not reported.

**Table:** Mache 2015

***Study characteristics***		
Methods	**Study design:** randomised controlled trial **Study grouping:** parallel group	
Participants	**Baseline characteristics** Psychosocial Resiliency Training *Age in years (mean ± SD)*: NR *Sex (N (% female))*: 26 (62%) *Sample size*: 42 *Years of experience (mean ± SD)*: NR Control (no intervention) *Age in years (mean ± SD)*: NR *Sex (N (% female))*: 25 (59%) *Sample size*: 43 *Years of experience (mean ± SD)*: NR Overall *Age in years (mean ± SD)*: 28 *Sex (N (% female))*: 51 (60%) *Sample size*: 85 *Years of experience (mean ± SD)*: NR **Included criteria:** inclusion criteria were (1) employment as a hospital doctor, (2) working at least full time, (3) work‐ing experience of less than a year, (4) being able and willing to participate, and (5) agreement to complete a survey at least two times. **Excluded criteria:** NR **Pretreatment:** baseline data on gender, age, and perceived health indicate only small, insignificant differences between the control and the intervention group. **Type of healthcare worker:** exclusively junior physicians **Response rate:** NR **Compliance rate:** 89%	
Interventions	**Intervention characteristics** Psychosocial Resiliency Training *Type of the intervention*: Intervention type 1 ‐ to focus one’s attention on the experience of stress *Description of the intervention*: psychosocial resilience training combined with cognitive behavioural and solution‐focused counselling in a group. The main focus was on coping strategies, support between the participants, and solutions and goals for the future. *The number of sessions*: 12 *Duration of each session on average*: 2 hours *Duration of the entire intervention*: 12 weeks *Duration of the entire intervention short vs long*: long *Intervention deliverer*: Two psychologists delivered the intervention. Both of them were familiar with cognitive behavioural and solution‐focused work in group settings *Intervention form*: Group and individual; face‐to‐face and at home Control (no intervention) *Type of the intervention*: NA *Description of the intervention*: NA *The number of sessions*: NA *Duration of each session on average*: NA *Duration of the entire intervention*: NA *Duration of the entire intervention short vs long*: NA *Intervention deliverer*: NA *Intervention form*: NA	
Outcomes	*Perceived Stress Questionnaire (PSQ)* **Outcome type**: ContinuousOutcome	
Identification	**Sponsorship source:** NR **Country:** Germany **Setting:** Hospital: several clinic departments specialising in different medical specialities (e.g., internal medicine, paediatrics, neurology, and gynaecology). **Comments:** NR **Authors name:** Stefanie Mache **Institution:** Institute for Occupational and Maritime Medicine (ZfAM), University Medical Center Hamburg‐Eppendorf **Email:** s.mache@uke.de **Address:** Seewartenstrasse 10, 20459 Hamburg, Germany **Time period:** February to August 2014	
Notes	PSQ included in analysis 1.1, 1.2	
***Risk of bias***		
**Bias**	**Authors' judgement**	**Support for judgement**
Random sequence generation (selection bias)	High risk	Quote: "physicians were randomized into an intervention and control group. Of"
Allocation concealment (selection bias)	Unclear risk	Not mentioned.
Blinding of participants and personnel (performance bias) All outcomes	High risk	Participants were not blinded.
Blinding of outcome assessment (detection bias) All outcomes	High risk	Participants were not blinded whereas outcomes are self‐reported.
Incomplete outcome data (attrition bias) All outcomes	Low risk	No loss to follow‐up.
Selective reporting (reporting bias)	Unclear risk	No trial registration, nor did we find one online.
Other bias	Unclear risk	Response rate not reported.

**Table:** Mache 2016

***Study characteristics***		
Methods	**Study design:** randomised controlled trial **Study grouping:** parallel group	
Participants	**Baseline characteristics** Self‐care health intervention programme *Age in years (mean ± SD)*: NR *Sex (N (% female))*: 27 (72%) *Sample size*: 37 *Years of experience (mean ± SD)*: NR Control (no intervention) *Age in years (mean ± SD)*: NR *Sex (N (% female))*: 24 (69%) *Sample size*: 35 *Years of experience (mean ± SD)*: NR Overall *Age in years (mean ± SD)*: 33 ± 2.3 *Sex (N (% female))*: 51 (71%) *Sample size*: 72 *Years of experience (mean ± SD)*: NR **Included criteria:** study participation requires positive inclusion criteria: (1) employment as a psychiatrist in a psychiatric department, (2) working full time, (3) being able and willing to take part in the study, (4) agreement to complete a survey at least three times. **Excluded criteria:** NR **Pretreatment:** only small, insignificant differences between intervention and control group have been found in baseline data on gender, age and working experience. Statistically significant positive advance was found for perceived stress, resilience and self‐efficacy in the intervention group. **Type of healthcare worker:** exclusively physicians **Response rate:** 51% **Compliance rate:** 95%	
Interventions	**Intervention characteristics** Self‐care health intervention programme *Type of the intervention*: Intervention type 1 ‐ to focus one’s attention on the experience of stress *Description of the intervention*: The focus was on actual working situations and problems, coping strategies and support between colleagues and future goals. The training sessions included psycho‐education (theoretical input, watching videos, oral group discussions, self‐awareness with experimental exercises and home assignments). *The number of sessions*: 12 *Duration of each session on average*: 1.5 hours *Duration of the entire intervention*: 12 weeks *Duration of the entire intervention short vs long*: long *Intervention deliverer*: Two psychotherapists performed the self‐care training. Both psychotherapists are registered and accredited as psychotherapists and clinical supervisors. They had qualifications in cognitive behavioural therapy, systemic therapy and solution‐focused brief therapy in individual and group settings. *Intervention form*: The intervention was performed off duty. Group and individual, face‐to‐face and at home. Control (no intervention) *Type of the intervention*: NA *Description of the intervention*: NA *The number of sessions*: NA *Duration of each session on average*: NA *Duration of the entire intervention*: NA *Duration of the entire intervention short vs long*: NA *Intervention deliverer*: NA *Intervention form*: NA	
Outcomes	*Perceived Stress Questionnaire (PSQ)* **Outcome type**: ContinuousOutcome	
Identification	**Sponsorship source:** NR **Country:** Germany **Setting:** Twelve hospital departments in the North of Germany specialising in Psychiatry Medicine **Comments:** NR **Authors name:** Stefanie Mache **Institution:** Institute for Occupational and Maritime Medicine (ZfAM), University Medical Center Hamburg‐Eppendorf **Email:** s.mache@uke.de **Address:** Seewartenstrasse 10, 20459 Hamburg, Germany **Time period:** NR	
Notes	PSQ included in analysis 1.1	
***Risk of bias***		
**Bias**	**Authors' judgement**	**Support for judgement**
Random sequence generation (selection bias)	Low risk	Quote: "study. These physicians were randomised into two groups through a computer‐generated algorithm."
Allocation concealment (selection bias)	Unclear risk	Quote: "The surveys were conducted by using a secure web‐based survey sys tem, via links within e‐mail messages."
Blinding of participants and personnel (performance bias) All outcomes	High risk	Participants not blinded.
Blinding of outcome assessment (detection bias) All outcomes	High risk	Participants were not blinded whereas outcomes are self‐reported.
Incomplete outcome data (attrition bias) All outcomes	Low risk	Quote: "76 participants; four needed to be excluded due to health reasons (sickness absence)." Loss to follow‐up below our review's pre‐defined cut‐off value.
Selective reporting (reporting bias)	Unclear risk	No trial registration, nor did we find one online.
Other bias	Low risk	No indication of other bias.

**Table:** Mache 2017

***Study characteristics***		
Methods	**Study design:** randomised controlled trial **Study grouping:** parallel group	
Participants	**Baseline characteristics** Psychosocial competency training program *Age in years (mean ± SD)*: 28.1 ± 2.3 *Sex (N (% female))*: 23 (58%) *Sample size*: 39 *Years of experience (mean ± SD)*: 1.2 ± 1.4 Control (wait list) *Age in years (mean ± SD)*: 27.6 ± 2.4 *Sex (N (% female))*: 25 (60%) *Sample size*: 41 *Years of experience (mean ± SD)*: 1.1 ± 1.3 Overall *Age in years (mean ± SD)*: 28 ± 2.3 *Sex (N (% female))*: 48 (60%) *Sample size*: 80 *Years of experience (mean ± SD)*: NR **Included criteria:** (1) regular access to the Internet, (2) working full time in the hospital, and (3) working experience of max. Two years (4) being able and willing to participate for the next 36 weeks, (5) agreement to complete the questionnaires, (6) no prior knowledge of or experience with a mental health promotion training. **Excluded criteria:** NR **Pretreatment:** baseline data on socio‐demographic differences indicated only small, insignificant differences between IG and CG (P > 0.05). **Type of healthcare worker:** junior physicians working in clinic departments of oncology and haematology medicine **Response rate:** 66% **Compliance rate:** 100%	
Interventions	**Intervention characteristics** Psychosocial competency training program *Type of the intervention*: Intervention type 1 ‐ to focus one’s attention on the experience of stress *Description of the intervention*: The intervention was based on Lazarus transactional model of stress, including 2 strategies of coping with stressors: problem‐and emotion‐oriented coping. The intervention contained elements of (1) the CBT approach and (2) the solution‐focused approach. Training sessions involved theoretical input, watching videos, oral group discussions, experiential exercises, and home assignments. *The number of sessions*: 12 *Duration of each session on average*: 1.5 hours *Duration of the entire intervention*: 12 weeks *Duration of the entire intervention short vs long*: long *Intervention deliverer*: Two qualified psychotherapists performed all training sessions. Both psychotherapists were registered and accredited as psychotherapists. They had sufficient qualifications and training in cognitive behavioural therapy (CBT), systemic therapy, and solution focused brief therapy. *Intervention form*: Combination of group and individual, face‐to‐face and at home. Control (wait list) *Type of the intervention*: NA *Description of the intervention*: NA *The number of sessions*: NA *Duration of each session on average*: NA *Duration of the entire intervention*: NA *Duration of the entire intervention short vs long*: NA *Intervention deliverer*: NA *Intervention form*: NA	
Outcomes	*Perceived Stress Questionnaire (PSQ)* **Outcome type**: ContinuousOutcome *Maslach Burnout Inventory ‐ Emotional Exhaustion* **Outcome type**: ContinuousOutcome	
Identification	**Sponsorship source:** NR **Country:** Germany **Setting:** Oncology and hematology hospital departments **Comments:** NR **Authors name:** Stefanie Mache **Institution:** Institute for Occupational and Maritime Medicine (ZfAM), University Medical Center Hamburg‐Eppendorf **Email:** s.mache@uke.de **Address:** NR **Time period:** NR	
Notes	MBI‐EE included in analysis 1.1, 1.2	
***Risk of bias***		
**Bias**	**Authors' judgement**	**Support for judgement**
Random sequence generation (selection bias)	Low risk	Quote: "participants were randomized with the ratio 50%:50% to the 2 study groups (IG or CG). The randomization was performed with a computer‐generated list of numbers."
Allocation concealment (selection bias)	Low risk	Quote: "This list was created by an independent assistant; the other assistant was blinded to the list, securing covered distribution to research conditions."
Blinding of participants and personnel (performance bias) All outcomes	High risk	Participants were not blinded.
Blinding of outcome assessment (detection bias) All outcomes	High risk	Participants were not blinded whereas outcomes are self‐reported.
Incomplete outcome data (attrition bias) All outcomes	Low risk	Quote: "There was a total dropout rate (from randomization to analyses) of 10%. Eleven participants decided not to finish the study (reasons included illness and participants did not show up) and did not answer the questionnaires. Overall, 4% (3 of 80) of participants at T1, 6% (5 of 80) of partici‐ pants at T2, and 5% (4 of 80) of participants at T3 did not provide all follow‐up data for the outcomes. Participants who did not provide all follow‐up data did not differ in a meaningful way from those who provided data, neither on the primary outcome or any other baseline outcomes (P > .05)"
Selective reporting (reporting bias)	Unclear risk	No trial registration, nor did we find one online.
Other bias	Low risk	No indication of other bias.

**Table:** Mache 2018

***Study characteristics***		
Methods	**Study design:** randomised controlled trial **Study grouping:** parallel group	
Participants	**Baseline characteristics** Mental health promotion program *Age in years (mean ± SD)*: 27.3 ± 2.5 *Sex (N (% female))*: 22 (62%) *Sample size*: 35 *Years of experience (mean ± SD)*: 1.4 ± 1.1 Control (wait list) *Age in years (mean ± SD)*: 27.1 ± 2.1 *Sex (N (% female))*: 24 (68%) *Sample size*: 35 *Years of experience (mean ± SD)*: 1.1 ± 1.2 Overall *Age in years (mean ± SD)*: 27 ± 2.4 *Sex (N (% female))*: 46 (66%) *Sample size*: 70 *Years of experience (mean ± SD)*: 1 ± 1.3 **Included criteria:** inclusion criteria were as follows: (a) employment in emergency medicine, (b) working full‐time in the hospital, (c) working experience of less than 3 years, (d) being able and willing to participate, (e) agreement to complete the questionnaires, and (f) e‐mail access, availability of a computer, tablet, or a smartphone, and access to the Internet. **Excluded criteria:** exclusion criteria were as follows: (a) having any psychiatric illness, (b) taking any psychiatric drugs, (c) engaging any counselling service, and (d) parallel use of psychosocial counselling. **Pretreatment:** baseline data on socio‐demographic differences indicated only small, insignificant differences between the intervention and the comparison group. **Type of healthcare worker:** exclusively junior physicians **Response rate:** NR **Compliance rate:** 100%	
Interventions	**Intervention characteristics** Mental health promotion program *Type of the intervention*: Intervention type 1 ‐ to focus one’s attention on the experience of stress *Description of the intervention*: The main focus was on actual working situations and problems, coping strategies, and support between colleagues and goals for the future. The training sessions included psycho‐education (theoretical input, watching videos, oral group discussions, experiential exercises, and home assignments). *The number of sessions*: 12 *Duration of each session on average*: 1.5 hours *Duration of the entire intervention*: 12 weeks *Duration of the entire intervention short vs long*: long *Intervention deliverer*: Two qualified psychologists, both trained in cognitive behavioural and solution‐focused work, performed the training. *Intervention form*: Group and individual, face‐to‐face and at home Control (wait list) *Type of the intervention*: NA *Description of the intervention*: NA *The number of sessions*: NA *Duration of each session on average* NA *Duration of the entire intervention*: NA *Duration of the entire intervention short vs long*: NA *Intervention deliverer*: NA *Intervention form*: NA	
Outcomes	*Perceived Stress Questionnaire (PSQ)* **Outcome type**: ContinuousOutcome *Maslach Burnout Inventory ‐ Emotional Exhaustion* **Outcome type**: ContinuousOutcome	
Identification	**Sponsorship source:** NR **Country:** Germany **Setting:** Six hospitals: clinic departments of emergency medicine **Comments:** NR **Authors name:** Stefanie Mache **Institution:** Institute for Occupational and Maritime Medicine (ZfAM), University Medical Center Hamburg‐Eppendorf **Email:** s.mache@uke.de **Address:** Seewartenstrasse 10, 20459 Hamburg, Germany **Time period:** NR	
Notes	MBi‐EE included in analysis 1.1, 1.2	
***Risk of bias***		
**Bias**	**Authors' judgement**	**Support for judgement**
Random sequence generation (selection bias)	Low risk	Quote: "participants were randomized at a ratio of 1: 1 to the two study arms (intervention or control group). The randomization was performed with a computer‐generated list of numbers."
Allocation concealment (selection bias)	Low risk	Quote: "This list was generated by an independent research assistant; the other researcher was blinded to the list, ensuring concealed allocation to research conditions."
Blinding of participants and personnel (performance bias) All outcomes	High risk	Participants were not blinded.
Blinding of outcome assessment (detection bias) All outcomes	High risk	Participants were not blinded whereas outcomes are self‐reported.
Incomplete outcome data (attrition bias) All outcomes	Low risk	63 of the 70 randomised participants included in the analysis.
Selective reporting (reporting bias)	Unclear risk	No trial registration, nor did we find on online
Other bias	Unclear risk	Response rate not reported.

**Table:** Mackenzie 2006

***Study characteristics***		
Methods	Randomised controlled trial, Canada	
Participants	30 nurses and nurse aides working in a large urban geriatric teaching hospital	
Interventions	1) Experimental: mindfulness‐based stress reduction programme: four 30‐minute group sessions including didactic section and experiential exercises. Participants also received a CD or audiocassette of guided exercises and a manual with the help of which they were instructed to practise for at least 10 minutes per day five days per week. 2) Control: no intervention	
Outcomes	MBI, Smith Relaxation Dispositions Inventory	
Identification		
Notes	MBI‐EE included in analysis 1.1	
***Risk of bias***		
**Bias**	**Authors' judgement**	**Support for judgement**
Random sequence generation (selection bias)	High risk	"Nurses and nurse aides were recruited from long‐term and complex continuing care units in a large urban geriatric teaching hospital and randomly assigned to intervention or wait‐list control groups. Because the study was conducted during the summer, however, several exceptions were made to accommodate participants' vacation schedules and additional control participants were recruited." (p. 106)
Allocation concealment (selection bias)	Unclear risk	Not reported.
Blinding of participants and personnel (performance bias) All outcomes	High risk	Participants not blinded.
Blinding of outcome assessment (detection bias) All outcomes	High risk	Participants were not blinded whereas outcomes are self‐reported.
Incomplete outcome data (attrition bias) All outcomes	Unclear risk	It is unclear if any participants dropped out.
Selective reporting (reporting bias)	Low risk	All outcomes reported
Other bias	Low risk	We did not find any indications of other sources of bias.

**Table:** Mandal 2021

***Study characteristics***		
Methods	**Study design:** randomised controlled trial **Study grouping:** parallel group	
Participants	**Baseline characteristics** Structured Yoga Program *Age (mean ± SD)*: 35 ± 7.9 *Sex (N (% female))*: 50 (86%) *Sample size*: 58 *Years of experience (mean ± SD)*: 12.1 + 7.7 Control (wait list) *Age (mean ± SD)*: 32.5 ± 6.8 *Sex (N (% female))*: 30 (58%) *Sample size*: 52 *Years of experience (mean ± SD)*: 10.3 + 7.7 Overall *Age (mean ± SD)*: NR *Sex (N (% female))*: 80 (73%) *Sample size*: 110 *Years of experience (mean ± SD)*: NR **Included criteria:** Working at the hospital for at least 1 year. **Excluded criteria:** We excluded those who were already under pharmacological treatment for any psychiatric disorder at the time of enrolment; having service left for less than a year (from the date of enrolment); any clinical condition that would affect the ability to practice yoga. **Pretreatment:** the baseline characteristics were comparable in both the groups except sex, where a higher proportion of males were present in the wait‐list group. The main outcome parameters were similar in both groups. As per the per‐protocol analysis the baseline parameters also showed the similar findings. **Type of healthcare worker:** exclusively in‐service nursing staff working at the hospital **Response rate:** 97% **Compliance rate:** 42%	
Interventions	**Intervention characteristics** Structured Yoga Program *Type of the intervention*: Intervention type 2 ‐ to focus one’s attention away from the experience of stress *Description of the intervention*: The yoga module which consisted of asana, pranayama, and deep relaxation technique was developed by a committee of yoga physicians and yoga therapists at the institutional yoga facility (Figure 1). We adopted the 5 minutes deep relaxation technique practised in supine position, Shavasana (Corpse Pose) which is an evidence based scientific way to relax the whole body completely within a short amount of time. It is usually, which literally translates to dead body posture. *The number of sessions*: 24 *Duration of each session on average*: 50 minutes *Duration of the entire intervention*: 12 weeks *Duration of the entire intervention short vs long*: long *Intervention deliverer*: The yoga therapists were trained professionals who completed their post‐graduation in the subject from reputed yoga institute *Intervention form*: All the yoga sessions were provided before or after their duty hours. Control (wait list) *Type of the intervention*: NA *Description of the intervention*: NA *The number of sessions*: NA *Duration of each session on average*: NA *Duration of the entire intervention*: NA *Duration of the entire intervention short vs long*: NA *Intervention deliverer*: NA *Intervention form*: NA	
Outcomes	*Perceived Stress Scale (PSS)* **Outcome type**: ContinuousOutcome	
Identification	**Sponsorship source:** The author(s) disclosed receipt of the following financial support for the research, authorship, and/or publication of this article: All the lab investigations were done free of cost for the participants. The necessary requirements were provided from the institution where I work. **Country:** India **Setting:** A tertiary care hospital **Comments:** NR **Authors name:** Puneet Misra **Institution:** Centre for Community Medicine, Room No. 30, Centre for Community Medicine, Old OT Block, All India Institute of Medical Sciences **Email:** doctormisra@gmail.com **Address:** Ansarinagar East, 110029, Delhi, India **Time period:** 2018	
Notes	Included in analysis 2.1	
***Risk of bias***		
**Bias**	**Authors' judgement**	**Support for judgement**
Random sequence generation (selection bias)	Low risk	Quote: "The investigation team collected unpredictable allocation sequence [using computer software and permuted block randomization] generated by a third party not involved in the study. The block size was multiple of 2 and variable in size."
Allocation concealment (selection bias)	Unclear risk	Quote: "After the baseline assessment was over, the sealed opaque envelop at her/his respective enrolment number was opened in front of the participants to maintain the allocation concealment. The participants were allocated in either of the 2 groups; intervention i.e. yoga group or the wait‐listed group." Difficult to judge whether participants and/or investigators could possibly foresee assignment.
Blinding of participants and personnel (performance bias) All outcomes	High risk	Quote: "Masking of the allocated group was not feasible in the study."
Blinding of outcome assessment (detection bias) All outcomes	High risk	Participants were not blinded whereas outcomes are self‐reported.
Incomplete outcome data (attrition bias) All outcomes	High risk	Quote: "Of the 52 participants joining the class, 25 participants discontinued in the first month, 5 participants in the second month and 3 participants in the third month. Therefore, the remaining 19 participants completed the minimum required 20 yoga sessions. At the end of the 12 weeks of follow up, 19 participants of intervention group and 32 participants of wait‐list control group was included in the analysis." Judgement Comment: > 20% loss to follow‐up
Selective reporting (reporting bias)	Unclear risk	No trial registration, nor did we find one online.
Other bias	Low risk	No indication of other sources of bias.

**Table:** Mao 2021

***Study characteristics***		
Methods	**Study design:** randomised controlled trial **Study grouping:** parallel group	
Participants	**Baseline characteristics** Emotional intelligence training *Age in years (mean ± SD)*: 30.6 ± 5.0 *Sex (N (% female))*: NR *Sample size*: 53 *Years of experience (mean ± SD)*: 9.3 ± 6.0 Control (daily training) *Age in years (mean ± SD)*: 31.3 ± 6.6 *Sex (N (% female))*: NR *Sample size*: 50 *Years of experience (mean ± SD)*: 9.7 ± 7.2 Overall *Age in years (mean ± SD)*: 30.9 ± 5.8 *Sex (N (% female))*: NR *Sample size*: 103 *Years of experience (mean ± SD)*: 9.5 ± 6.6 **Included criteria:** the inclusion criterion was the possession of Chinese nurses' practice qualification certificates. **Excluded criteria:** the exclusion criteria were as follows: (a) suffering from serious physical or mental illness; (b) taking psychotropic drugs; (c) nursing staff who took maternity leave, sick leave, retirement, or further study in the study period; (d) had participated in systematic EI training before; and (e) had worked in a psychiatric ward. If participants in the intervention group were absent twice, they were excluded from the study. **Pretreatment:** there were no statistically significant differences in demographic characteristics between the two groups at baseline. **Type of healthcare worker:** exclusively nurses **Response rate:** 76% **Compliance rate:** 81%	
Interventions	**Intervention characteristics** Emotional intelligence training *Type of the intervention*: Intervention type 1 ‐ to focus one’s attention on the experience of stress *Description of the intervention*: In the system training phase, educators explained emotional intelligence to the intervention group through class lectures. Lectures covered themes such as perception of emotions, awareness of emotions, regulation of emotions and practice. // Phase II was the consolidated learning phase. The process of each case discussion was as follows: (a) Preparation, (b) Case description, (c) Case confirmation, (d) Case discussion, (e) Case summary *The number of sessions*: 48 *Duration of each session on average*: 60 to 90 minutes *Duration of the entire intervention*: 48 weeks *Duration of the entire intervention short vs long*: long *Intervention deliverer*: four educators with a National Counsellor Level 2 or higher certification, a master's degree, and 10 years of clinical psychology teaching experience to deliver the EI intervention to the nurses *Intervention form*: Class lectures, case discussions Control (received daily briefings in meetings between head nurses, which were held regularly to discuss specific problems. There was no emotional intelligence training conducted with the control group.) *Type of the intervention*: NA *Description of the intervention*: NA *The number of sessions*: NA *Duration of each session on average*: NA *Duration of the entire intervention*: NA *Duration of the entire intervention short vs long*: NA *Intervention deliverer*: NA *Intervention form*: NA	
Outcomes	*Perceived Stress Scale (PSS)* **Outcome type**: ContinuousOutcome	
Identification	**Sponsorship source:** This study was supported by the Nursing Research Project of The Second Xiangya Hospital, Central South University (2017‐YHL‐15). **Country:** China **Setting:** NR **Comments:** NR **Authors name:** LingZhi Huang, QiongNi Chen **Institution:** The Second Xiangya Hospital, Central South University **Email:** huanglingzhi@csu.edu.cn, chenqiongni@csu.edu.cn **Address:** 139 Renmin Middle Road, The Second Xiangya Hospital, Central South University, Changsha, Hunan 410011, China. **Time period:** January 2019 ‐ January 2020	
Notes	PSS included in analysis 1.1	
***Risk of bias***		
**Bias**	**Authors' judgement**	**Support for judgement**
Random sequence generation (selection bias)	Unclear risk	Quote: "Ten wards were randomly assigned to the intervention group and ten to the control group."
Allocation concealment (selection bias)	Unclear risk	Difficult to judge whether participants and/or investigators could possibly foresee assignment.
Blinding of participants and personnel (performance bias) All outcomes	High risk	Participants were not blinded.
Blinding of outcome assessment (detection bias) All outcomes	High risk	Participants were not blinded whereas outcomes are self‐reported.
Incomplete outcome data (attrition bias) All outcomes	Low risk	Quote: "Overall, the intervention and control groups lost 10 and 5 participants, respectively." 15 of the 103 randomised participants lost to follow‐up, which is below our pre‐defined cut‐off value.
Selective reporting (reporting bias)	Unclear risk	No trial registration, nor did we find one online.
Other bias	Low risk	No indication of other bias.

**Table:** Martins 2011

***Study characteristics***		
Methods	Randomised controlled trial, Argentina	
Participants	74 hospital paediatric resident physicians. " A total of 81% were female; the mean age was 27.3 ± 1.4 years; 57% were working in inpatient areas, 35% in the outpatient clinic, and 8% in the intensive care unit." " A comparison of the characteristics of both groups (experimental and control) revealed no significant differences." (p. 494)	
Interventions	1) Experimental: self‐care workshop intervention (n = 37). quote: "The experimental group received a brief intervention consisting of two 2.5‐hour workshops directed by mental health professionals, which covered repercussions of burnout syndrome on professional activity, recognition of risk indicators for burnout syndrome, and tools to cope (identification of strengths, coping behaviors, preventive and self‐care behaviors)." (p. 494) 2) Control: (n = 37) No intervention	
Outcomes	MBI	
Identification		
Notes	It is unclear why 43 (37%) out of the available 117 resident physicians did not participate in the study. Not able to include in analysis due to missing data.	
***Risk of bias***		
**Bias**	**Authors' judgement**	**Support for judgement**
Random sequence generation (selection bias)	Unclear risk	"After administration of the questionnaire, subjects were randomly assigned to one of the two study groups." (p. 494)
Allocation concealment (selection bias)	Unclear risk	Not reported.
Blinding of participants and personnel (performance bias) All outcomes	High risk	Participants not blinded.
Blinding of outcome assessment (detection bias) All outcomes	High risk	Participants were not blinded whereas outcomes are self‐reported.
Incomplete outcome data (attrition bias) All outcomes	Unclear risk	Apparently no participants were lost to follow‐up
Selective reporting (reporting bias)	Unclear risk	The authors do not report standard deviations with the mean MBI subscale scores.
Other bias	Unclear risk	It is unclear if these 74 were all the participants or only those that could be followed up.

**Table:** McConachie 2014

***Study characteristics***		
Methods	**Study design:** randomised controlled trial **Study grouping:** parallel group	
Participants	**Baseline characteristics** Acceptance and mindfulness workshop *Age in years (median / range)*: 43 / 19‐69 *Sex (N (% female))*: 47 (71%) *Sample size*: 66 *Years of experience (median / range)*: 6.5 / 0.5‐25 Control (wait list) *Age in years (median / range)*: 44 / 22‐64 *Sex (N (% female))*: 42 (78%) *Sample size*: 54 *Years of experience (median / range)*: 6.4 / 0.9‐30 Overall *Age in years (median / range)*: 43 / 19‐69 *Sex (N (% female))*: 89 (74%) *Sample size*: 120 *Years of experience (median / range)*: 6.4 / 0.5‐30 **Included criteria:** inclusion criteria were that participants were over 18 years, able to provide informed consent, and had at least six months experience of working within ID services. **Excluded criteria:** NR **Pretreatment:** no significant differences were found between the intervention and control groups in relation to age, experience of working in ID services, hours worked per week, gender, professional qualifications or education. **Type of healthcare worker:** Support staff involved in the direct care of individuals with ID. **Response rate:** 100% **Compliance rate:** 85%	
Interventions	**Intervention characteristics** Acceptance and mindfulness workshop *Type of the intervention*: Intervention type 1 ‐ to focus one’s attention on the experience of stress *Description of the intervention*: The overall aim of the workshop was to change the way support staff reacted to stressful situations, such as supporting a client with ID and who displayed behaviour that challenges. The workshop involved the use of didactic teaching, group discussions, written exercises, the use of metaphors, short video presentations and practical and interactive exercises– all of which aimed to illustrate the key components of the intervention. Mindfulness exercises were practised during sessions, and given as homework assignments to be completed between sessions. *The number of sessions*: 2 *Duration of each session on average*: full day and half day *Duration of the entire intervention*: 6 weeks *Duration of the entire intervention short vs long* : short *Intervention deliverer*: NR *Intervention form*: In‐person group sessions and homework assignments Control (wait list) *Type of the intervention*: NA *Description of the intervention*: NA *The number of sessions*: NA *Duration of each session on average*: NA *Duration of the entire intervention*: NA *Duration of the entire intervention short vs long*: NA *Intervention deliverer*: NA *Intervention form*: NA	
Outcomes	*Staff Stressor Questionnaire (SSQ)* **Outcome type**: ContinuousOutcome *General Health Questionnaire (GHQ‐12)* **Outcome type**: ContinuousOutcome	
Identification	**Sponsorship source:** NR **Country:** NR **Setting:** Independent care organisations working with individuals with ID **Comments:** NR **Authors name:** Douglas AlexanderJames McConachie **Institution:** University of Edinburgh, Department of Clinical Psychology, School of Health in Social Science **Email:** Douglas.mcconachie@nhslothian.scot.nhs.uk **Address:** University of Edinburgh, Teviot Place, Edinburgh, Scotland EH8 9AG, UK **Time period:** NR	
Notes	GHQ included in analysis 1.1	
***Risk of bias***		
**Bias**	**Authors' judgement**	**Support for judgement**
Random sequence generation (selection bias)	Low risk	Quote: "Permuted block randomisation was used to generate quasi‐random numbers (www.jerrydallal.com/random/ random_block_size.htm) to allocate the 120 participants to the intervention or control conditions (see Fig. 1)."
Allocation concealment (selection bias)	High risk	Quote: "there was no allocation concealment, and the allocation of staff to the two conditions was not fully adhered to by line managers."
Blinding of participants and personnel (performance bias) All outcomes	High risk	Quote: "The latter factor is a particular source of potential bias, as the reason the participants changed conditions is unknown. They may have either been particularly motivated to attend the workshop, or the line manager may have been keen for them to attend or not attend."
Blinding of outcome assessment (detection bias) All outcomes	High risk	Participants not blinded whereas outcomes are self‐reported
Incomplete outcome data (attrition bias) All outcomes	Low risk	Quote: "There were similar levels of attrition from both the intervention and control group (see Fig. 1). The data was found to be missing completely at random (MCAR) (Schlomer, Bauman, & Card, 2010) considering all cases and outcome measures MCAR (P > 0.05) (X 2 = 30.686, df = 27, p = .284). The" 27.5 % lost to follow‐up ‐> MCAR
Selective reporting (reporting bias)	Low risk	No trial registration, no indication of selective reporting.
Other bias	Low risk	No indication of other bias.

**Table:** McGonagle 2020

***Study characteristics***		
Methods	**Study design:** randomised controlled trial **Study grouping:** parallel group	
Participants	**Baseline characteristics** Positive psychology‐based coaching intervention *Age in years (mean ± SD)*: 43.41 (8.76) *Sex (N (% female))*: 21 (72.41%) *Sample size*: 29 *Years of experience (mean ± SD)*: 12.12 (7.40) Control (wait list) *Age in years (mean ± SD)*: 41.83 (7.42) *Sex (N (% female))*: 25 (86.21%) *Sample size*: 29 *Years of experience (mean ± SD)*: 10.05 (7.47) Overall *Age in years (mean ± SD)*: NR *Sex (N (% female))*: 46 (79.31%) *Sample size* : 58 *Years of experience (mean ± SD)*: NR **Included criteria:** inclusion criteria were currently working at least part‐time as a PCP (0.5 FTE clinical practice), having 25 years or less of experience as a PCP, and not planning to retire within two years. **Excluded criteria:** potential participants were screened for psychological distress using the SCL‐10 (Nguyen et al.,1983). We used the cut‐off score determined by Müller et al. (2010) of 4.0 to indicate those with high levels of psychological distress and a licenced mental health professional was retained to speak with those who reported a level of distress ≥ 4.0. All participants attained scores < 4.0 **Pretreatment:** no demographic variables were significantly different between the primary and wait‐listed groups. **Type of healthcare worker:** exclusively Primary Care Physician **Response rate:** 100% **Compliance rate:** 97%	
Interventions	**Intervention characteristics** Positive psychology‐based coaching intervention *Type of the intervention*: Intervention type 1 ‐ to focus one’s attention on the experience of stress *Description of the intervention*: Prior to the first coaching session, participants completed the Workplace PERMA Profiler (Butler & Kern, 2016) which measures the five pillars of PERMA and workplace well‐being. The individual’s PERMA results were shared by the coach at the first session as a standardised focus for that first conversation. The last coaching session focused on assessing progress, defining ways to sustain success, and conducting a gratitude reflection. The second of the fifth sessions utilised participant‐chosen topics and a toolbox of evidence‐based positive psychology coaching exercises, designed to be used flexibly based on client goals and learning preferences. *The number of sessions*: 6 *Duration of each session on average*: 30 minutes *Duration of the entire intervention*: 12 weeks *Duration of the entire intervention short vs long*: long *Intervention deliverer*: While the five study coaches differed in terms of specific degrees and certifications attained, all agreed on the coaching format, philosophy, and tools used in this study. Coaches had between 11 and 25 years of professional coaching experience, and all previously coached healthcare personnel. All held post‐graduate degrees, including a doctorate in organisational behaviour and master’s degrees in health psychology, human resource management, mental health counselling, anthropology, adult and organisational learning, and health education. *Intervention form*: The first session was conducted face to face and the remainder were conducted by phone Control (wait list) *Type of the intervention*: NA *Description of the intervention*: NA *The number of sessions*: NA *Duration of each session on average*: NA *Duration of the entire intervention*: NA *Duration of the entire intervention short vs long*: NA *Intervention deliverer*: NA *Intervention form*: NA	
Outcomes	*Stress in General Scale* **Outcome type**: ContinuousOutcome **Notes**: (Stanton et al., 2001) *Maslach Burnout Index* **Outcome type**: ContinuousOutcome **Notes**: (Maslach et al.,1996)	
Identification	**Sponsorship source:** This project was supported by the Institute of Coaching at McLean Hospital, Harvard Medical School affiliate. **Country:** United States **Setting:** Four medical practices in a large city (both community and hospital‐based settings). **Comments:** NR **Authors name:** Alyssa McGonagle **Institution:** University of North Carolina at Charlotte **Email:** amcgonag@uncc.edu **Address:** 9201 University City Boulevard, Charlotte, NC 28223‐0001 **Time period:** NR	
Notes	MBI ‐ one combined scale included in analysis 1.1	
***Risk of bias***		
**Bias**	**Authors' judgement**	**Support for judgement**
Random sequence generation (selection bias)	Low risk	Quote: "and received a participant code. Eligible participants then completed an initial survey assessing all outcome measures, and were randomized using a coin flip into either an immediate start coaching group (primary) or wait‐listed control group with a six‐month delay.
Allocation concealment (selection bias)	Unclear risk	Not mentioned.
Blinding of participants and personnel (performance bias) All outcomes	High risk	Participants were not blinded.
Blinding of outcome assessment (detection bias) All outcomes	High risk	Participants were not blinded whereas outcomes are self‐reported.
Incomplete outcome data (attrition bias) All outcomes	Low risk	No loss to follow‐up.
Selective reporting (reporting bias)	Low risk	No trial registration, no indication of selective reporting.
Other bias	Unclear risk	Judgement Comment: The authors combined the MBI into one scale, which is not according to the MBI handbook.

**Table:** Mealer 2014

***Study characteristics***		
Methods	**Study design:** randomised controlled trial **Study grouping:** parallel group	
Participants	**Baseline characteristics** Multimodal resilience training program *Age in years (mean ± SD)*: NR *Sex (N (% female))*: 12 (92%) *Sample size*: 13 *Years of experience (mean ± SD)*: 4.9 ± 4.2 Control (no intervention) *Age in years (mean ± SD)*: NR *Sex (N (% female))*: 12 (86%) *Sample size*: 14 *Years of experience (mean ± SD)*: 5.8 ± 7.4 Overall *Age in years (mean ± SD)*: NR *Sex (N (% female))*: 24 (89%) *Sample size*: 27 *Years of experience (mean ± SD)*: NR **Included criteria:** nurses were eligible to participate if they (1) were currently working 20 hours per week at the ICU bedside, (2) had no underlying medical condition that would be a contraindication to exercise, and (3) scored 82 or less on the Connor‐Davidson Resilience Scale (CD‐RISC). **Excluded criteria:** nurses were excluded from participating if they (1) were unable to participate in a two‐day educational workshop or (2) had a medical condition that would limit exercise. **Pretreatment:** measures of PTSD, burnout syndrome, resiliency, and symptoms of anxiety or depression did not differ significantly between the 2 groups. **Type of healthcare worker:** exclusively ICU nurses **Response rate:** NR **Compliance rate:** 93%	
Interventions	**Intervention characteristics** Multimodal resilience training program *Type of the intervention*: Intervention type 4 ‐ Combination of two or more of the above *Description of the intervention*: The intervention included a 2‐day educational workshop, written exposure sessions, event‐triggered counselling sessions, mindfulness‐based stress reduction exercises, and a protocolised aerobic exercise regimen. *The number of sessions*: 84 *Duration of each session on average*: 30 *Duration of the entire intervention*: 12 weeks *Duration of the entire intervention short vs long*: long *Intervention deliverer*: A MBSR expert, expressive writing expert and an experienced licenced clinical social worker trained in traumatic stress. *Intervention form*: Combination Control (no intervention) *Type of the intervention*: NA *Description of the intervention*: NA *The number of sessions*: NA *Duration of each session on average*: NA *Duration of the entire intervention*: NA *Duration of the entire intervention short vs long*: NA *Intervention deliverer*: NA *Intervention form*: NA	
Outcomes	*Maslach Burnout Inventory ‐ Emotional exhaustion* **Outcome type**: ContinuousOutcome *Maslach Burnout Inventory ‐ Depersonalization* **Outcome type**: ContinuousOutcome *Maslach Burnout Inventory ‐ Personal accomplishment (lack of)* **Outcome type**: ContinuousOutcome *The Hospital Anxiety and Depression Scale (HADS)* **Outcome type**: ContinuousOutcome	
Identification	**Sponsorship source:** This study was funded by a grant from the National Institutes of Health (grant number K24 HL‐089223‐07). **Country:** United States **Setting:** An academic institution **Comments:** NR **Authors name:** Meredith Mealer **Institution:** Pulmonary Sciences and Critical Care Medicine, University of Colorado **Email:** Meredith.Mealer@ucdenver.edu **Address:** Anschutz Medical Center, 12700 E 19th Ave, C‐272, Aurora, CO 80045 **Time period:** 2012‐2013	
Notes	MBI‐EE included in analysis 4.1 HADS included in analysis 4.4	
***Risk of bias***		
**Bias**	**Authors' judgement**	**Support for judgement**
Random sequence generation (selection bias)	Low risk	Quote: "An honest broker was used to ensure that participants’ responses remained anonymous. The honest broker was not part of the study team, assigned unique identification numbers to participants, and then linked individual participants’ information with those identification numbers.
Allocation concealment (selection bias)	Unclear risk	See above Difficult to judge whether participants could possibly foresee assignment.
Blinding of participants and personnel (performance bias) All outcomes	High risk	Quote: "All data were entered into the REDCap data management system 30 by using unique study identfication numbers so that study personnel remained blinded to the identity of the participants. Participants were not blinded.
Blinding of outcome assessment (detection bias) All outcomes	High risk	Participants were not blinded whereas outcomes are self‐reported.
Incomplete outcome data (attrition bias) All outcomes	Low risk	Quote: "randomized to the control arm. Two participants withdrew from the study before the start of the 12‐week training period: 1 from the intervention arm and 1 from the control arm. Therefore, 27 participants participated"
Selective reporting (reporting bias)	Unclear risk	No trial registration, nor did we find one online.
Other bias	Unclear risk	Response rate not reported.

**Table:** Medisauskaite 2019

***Study characteristics***		
Methods	**Study design:** randomised controlled trial **Study grouping:** parallel group	
Participants	**Baseline Characteristics** Interventions about the psychology of burnout, stress, coping with patient death, and managing distress *Age in years (mean ± SD)*: NR *Sex (N (% female))*: 14 (35.9%) *Sample size*: 39 *Years of experience (mean ± SD)*: 23.90 (11.18) Control (no intervention) *Age in years (mean ± SD)*: NR *Sex (N (% female))*: 28 (53.8%) *Sample size*: 52 *Years of experience (mean ± SD)*: 24.54 (11.39) Overall *Age in years (mean ± SD)*: NR *Sex (N (% female))*: 42 (46.2%) *Sample size*: 91 *Years of experience (mean ± SD)*: 24.26 (11.25) **Included criteria:** doctors who currently practice medicine, have regular contact with patients **Excluded criteria:** NR **Pretreatment:** there were no significant differences between the two trial groups at baseline **Type of healthcare worker:** exclusively doctors **Response rate:** 89% **Compliance rate:** 78%	
Interventions	**Intervention characteristics** Interventions about the psychology of burnout, stress, coping with patient death, and managing distress *Type of the intervention*: Intervention type 1 ‐ to focus one’s attention on the experience of stress *Description of the intervention*: We developed and piloted an intervention consisting of four modules. Module 1 taught doctors about stress, Module 2 taught doctors about burnout, Module 3 taught doctors about coping with patient death, and Module 4 taught doctors about methods of managing distress. *The number of sessions*: NR *Duration of each session on average*: NR *Duration of the entire intervention*: 7 days *Duration of the entire intervention short vs long*: short *Intervention deliverer*: NR *Intervention form*: NR Control (no intervention) *Type of the intervention*: NA *Description of the intervention*: NA *The number of sessions*: NA *Duration of each session on average*: NA *Duration of the entire intervention*: NA *Duration of the entire intervention short vs long*: NA *Intervention deliverer*: NA *Intervention form*: NA	
Outcomes	*Maslach Burnout Inventory ‐ Emotional exhaustion* **Outcome type**: ContinuousOutcome *Maslach Burnout Inventory ‐ Depersonalisation* **Outcome type**: ContinuousOutcome *Maslach Burnout Inventory ‐ Personal accomplishment* **Outcome type**: ContinuousOutcome *General Anxiety Disorder* **Outcome type**: ContinuousOutcome **Scale**: The General Anxiety Disorder‐7 (Spitzer et al. 2006)	
Identification	**Sponsorship source:** The RCT was not funded or determined by Focus Games or any organization involved with the app/board game. **Country:** United Kingdom **Setting:** Among nine randomly selected NHS trusts, 9 royal colleges of medicine, and the British Medical Association (BMA) **Comments:** NR **Authors name:** Asta Medisauskaite **Institution:** Research Department of Medical Education **Email:** a.medisauskaite@ucl.ac.uk **Address:** UCL Medical School Room GF/644, Royal Free Hospital, NW3 2PF. **Time period:** From July to November 2016	
Notes	MBI‐EE included in analysis 1.1. GAD included in analysis 1.4.	
***Risk of bias***		
**Bias**	**Authors' judgement**	**Support for judgement**
Random sequence generation (selection bias)	Low risk	Quote: "Qualtrics software randomly assigned doctors to one of 5 trial groups:"
Allocation concealment (selection bias)	Low risk	Quote: "Blindly to the researchers, Qualtrics software randomly assigned doctors to one of 5 trial groups:"
Blinding of participants and personnel (performance bias) All outcomes	Low risk	Quote: "doctors were randomly and blindly assigned to one of 5 trial groups."
Blinding of outcome assessment (detection bias) All outcomes	Low risk	Quote: "The trial group was manipulated between‐subjects such that doctors were randomly and blindly assigned to one of 5 trial groups." Outcomes are self‐reported.
Incomplete outcome data (attrition bias) All outcomes	High risk	Quote: "2.2.4. Sample" Loss to follow‐up: 25% intervention; 27% control
Selective reporting (reporting bias)	Unclear risk	No trial registration, nor did we find one online.
Other bias	Low risk	No indication of other bias.

**Table:** Melchior 1996

***Study characteristics***		
Methods	RCT, the Netherlands	
Participants	161 psychiatric nurses in long‐stay settings	
Interventions	1) Experimental: support and advice given by nurse managers or quality care co‐ordinators: Participants were assigned to patients as primary nurses and given advice by nurse managers or quality care co‐ordinators and they followed a training programme about communication skills over a year. 2) Control: no intervention	
Outcomes	MBI	
Identification		
Notes	MBI‐EE included in analysis 3.3.	
***Risk of bias***		
**Bias**	**Authors' judgement**	**Support for judgement**
Random sequence generation (selection bias)	Unclear risk	Quote: "Random sampling was used to select 492 nurses to complete the questionnaires." (p. 696)
Allocation concealment (selection bias)	Unclear risk	Not reported
Blinding of participants and personnel (performance bias) All outcomes	High risk	Participants not blinded.
Blinding of outcome assessment (detection bias) All outcomes	High risk	Participants were not blinded whereas outcomes are self‐reported.
Incomplete outcome data (attrition bias) All outcomes	High risk	Quote: "Two main problems were encountered in this study, namely a high drop‐out rate largely due to job turnover among nurses, and the imitation of the intervention by the control group." (p. 697) A total of 51.6% of the participants dropped out during the study
Selective reporting (reporting bias)	Low risk	All outcomes reported.
Other bias	Low risk	We did not find any indications of other sources of bias.

**Table:** Moench 2021

***Study characteristics***		
Methods	**Study design:** randomised controlled trial **Study grouping:** parallel group	
Participants	**Baseline characteristics** Self‐Care Traumatic Episode Protocol *Age in years (mean ± SD)*: NR *Sex (N (% female))*: NR *Sample size*: 16 *Years of experience (mean ± SD)*: NR Control (wait list) *Age in years (mean ± SD)*: NR *Sex (N (% female))*: NR *Sample size*: 17 *Years of experience (mean ± SD)*: NR Overall *Age in years (mean ± SD)*: NR *Sex (N (% female))*: NR *Sample size*: 33 *Years of experience (mean ± SD)*: NR **Included criteria:** participants were considered suitable for study inclusion if they met the following criteria: they were willing to participate voluntarily in treatment; they provided written consent; and were licenced mental health clinicians who had taken basic EMDR training. **Excluded criteria:** participants were excluded if they disclosed severe levels of clinical distress, if they were concurrently receiving psychological treatment during the study period, or if they endorsed suicidal intent. **Pretreatment:** NR **Compliance rate:** 94% **Response rate:** NR **Type of healthcare worker:** 34 participants included master’s level clinical social workers (n = 8), Canadian Certified Counsellors (n = 4), master’s or PhD‐level registered psychologists (n = 21), and psychiatrists (n = 1).	
Interventions	**Intervention characteristics** Self‐Care Traumatic Episode Protocol *Type of the intervention*: Intervention type 4 ‐ Combination of two or more of the above *Description of the intervention*: The intervention required participating in a video‐ and worksheet‐guided STEP intervention protocol. Within the intervention, participants watched a series of videos (e.g. on self‐care, relaxation), using the protocol to assist in processing any points of disturbance that would come up in relation to the current situation that they have found distressing or what may happen in the future in relation to the COVID‐19 pandemic. Participants were asked to target the COVID‐19 episode from the onset of COVID‐19 until now or even into the future. *The number of sessions*: 1 *Duration of each session on average*: 1.5 hours *Duration of the entire intervention*: 0 weeks *Duration of the entire intervention short vs long*: short *Intervention deliverer*: Therapists were the first author and a Master of Counselling graduate student in her last year of the program. *Intervention form*: individual, distance Control (wait list) *Type of the intervention*: NA *Description of the intervention*: NA *The number of sessions*: NA *Duration of each session on average*: NA *Duration of the entire intervention*: NA *Duration of the entire intervention short vs long*: NA *Intervention deliverer*: NA *Intervention form*: NA	
Outcomes	*Depression Anxiety Stress Scale (DASS‐21)* **Outcome type**: ContinuousOutcome **Scale**: Lovibond & Lovibond, 1995	
Identification	**Sponsorship source:** the authors received no specific grant or financial support for the research, authorship, and/or publication of this article. **Country:** Canada **Setting:** NR **Comments:** NR **Authors name:** Judy Moench **Institution:** Judy Moench Psychological Services Ltd. **Email:** jmoench@telusplanet.net **Address:** #260, 10230 142 Street, NW Edmonton, AB, T5N 3Y6, Canada. **Time period:** June 2020	
Notes	Included in analysis 4.1	
***Risk of bias***		
**Bias**	**Authors' judgement**	**Support for judgement**
Random sequence generation (selection bias)	Low risk	Using a randomisation sequence based on a random number table.
Allocation concealment (selection bias)	Unclear risk	Participants were randomized by a research assistant. Insufficient information to understand whether intervention allocations could have been foreseen by participants in advance of, during, enrolment.
Blinding of participants and personnel (performance bias) All outcomes	High risk	Participants were not blinded.
Blinding of outcome assessment (detection bias) All outcomes	High risk	Participants were not blinded whereas outcomes are self‐reported.
Incomplete outcome data (attrition bias) All outcomes	Low risk	5% lost to follow‐up.
Selective reporting (reporting bias)	Unclear risk	No trial registration, nor did we find one online.
Other bias	Unclear risk	Response rate was not reported.

**Table:** Montaner 2021

***Study characteristics***		
Methods	**Study design: randomised** controlled trial **Study grouping:** parallel group	
Participants	**Baseline characteristics** Acceptance and Commitment Therapy (ACT) *Age (mean ± SD)*: 40.5 *±* 12.8 *Sex (N (% female))*: 49 (96%) *Sample size*: 51 *Years of experience (mean ± SD)*: NR Control (wait list) *Age (mean ± SD)*: 41.8 *±* 12.3 *Sex (N (% female))*: 49 (91%) *Sample size*: 54 *Years of experience (mean ± SD)*: NR Overall *Age (mean ± SD)*: NR *Sex (N (% female))*: 98 (93%) *Sample size*: 105 *Years of experience (mean ± SD)*: NR **Included criteria:** the inclusion criteria were to be over 18 years old and have at least 6 months of experience in the ce **Excluded criteria:** NR **Pretreatment:** no significant group differences were found at baseline for demographics, neither for outcome measures. **Compliance rate:** 86% **Response rate:** 43% **Type of healthcare worker:** various	
Interventions	**Intervention characteristics** Acceptance and Commitment Therapy (ACT) *Type of the intervention*: Intervention type 4 ‐ Combination of two or more of the above *Description of the intervention*: The intervention was specifically designed for workers providing services in the dementia context. Implement each of the hexaflex components of ACT (Contact with the Present Moment, Acceptance, Self as Context, Cognitive Defusion, Values and Committed Action) in different sessions (including meditation exercise). *The number of sessions*: 6 sessions *Duration of each session on average*: 90 minutes *Duration of the entire intervention*: NR *Duration of the entire intervention short vs long*: Short *Intervention deliverer* :neuropsychologist of the Social and Health Centre widely trained and experienced in dementia care and ACT *Intervention form*: conducted in the work setting Control (wait list) *Type of the intervention*: NA *Description of the intervention*: NA *The number of sessions*: NA *Duration of each session on average*: NA *Duration of the entire intervention*: NA *Duration of the entire intervention short vs long*: NA *Intervention deliverer*: NA *Intervention form*: NA	
Outcomes	*Maslach Burnout Inventory ‐ Emotional Exhaustion* **Outcome type**: ContinuousOutcome *Maslach Burnout Inventory ‐ Depersonalisation* **Outcome type**: ContinuousOutcome *Maslach Burnout Inventory ‐ Personal accomplishment* **Outcome type**: ContinuousOutcome *State‐Trait Anxiety Inventory (STAI) ‐ Anxiety‐Trait* **Outcome type**: ContinuousOutcome	
Identification	**Sponsorship source:** **Country:** NR **Setting:** CSSV Ricard Fortuny Hospital: a center made up of 6 long‐term hospitalization units, two nursing home units, a day center and a palliative unit. **Comments:** NR **Authors name:** Xavier Montaner **Institution:** Consorci Sociosanitari Ricard Fortuny **Email:** xavier.casino@gmail.com **Address:** Avinguda Garraf 3, 08720, Vilafranca del Penedés (Barcelona), Spain **Time period:** The study was carried out between May 2017 and September 2018	
Notes	MBI‐EE included in analysis 4.1 and 4.2 STAI included in analysis 4.4 and 4.5	
***Risk of bias***		
**Bias**	**Authors' judgement**	**Support for judgement**
Random sequence generation (selection bias)	Unclear risk	Quote: "randomly assigned"
Allocation concealment (selection bias)	Unclear risk	Not described
Blinding of participants and personnel (performance bias) All outcomes	High risk	Participants were not blinded.
Blinding of outcome assessment (detection bias) All outcomes	High risk	Participants were not blinded whereas outcomes are self‐reported.
Incomplete outcome data (attrition bias) All outcomes	Low risk	Quote: "The drop‐out rate at the end of the intervention in the sample as a whole was 15.5%, 17.3% at 3 months of follow‐up, and 20% at 12 months of follow‐up. Although the attrition effect was higher in the IG, there were no statistically significant differences between groups drop‐out rates (Table 2)."
Selective reporting (reporting bias)	Unclear risk	No trial registration, nor did we find one online.
Other bias	Low risk	No indication of other bias.

**Table:** Montibeler 2018

***Study characteristics***		
Methods	**Study design:** randomised controlled trial **Study grouping:** parallel group	
Participants	**Baseline characteristics** Massage with aromatherapy *Age in years (range(N (%)))*: 25‐39 (12 (63%)) *Sex (N (% female))*: 17 (89%) *Sample size*: 19 *Years of experience (range(N (%)))*: > 5 years (11 (58%)) Control (no intervention) *Age in years (range (N (%)))*: 25‐39 (9 (47%)) *Sex (N (% female))*: 16 (84%) *Sample size*: 19 *Years of experience (range(N (%)))*: > 5 years (14 (74%)) Overall *Age in years (range(N (%)))*: 25‐39 (21 (55%)) *Sex (N (% female))*: 33 (87%) *Sample size*: 38 *Years of experience (range(N (%)))*: > 5 years (25 (66%)) **Included criteria:** under employment bond and working in the surgical centre for at least one year; acceptance to participate in the study, including the stages of the study protocol; score of at least 12 points on the List of Stress Symptoms (LSS); and olfactory acceptance of the Lavandula angustifolia and Pelargonium graveolens aromas. **Excluded criteria:** all workers on vacation or on leave during the data collection period, as well as pregnant women. **Pretreatment:** except for the variable of time in the institution, there were no differences with statistical significance, confirming homogeneity among the study groups. **Type of healthcare worker:** nursing staff workers (nurses and nursing technicians) **Response rate:** 81% **Compliance rate:** 100%	
Interventions	**Intervention characteristics** Massage with aromatherapy *Type of the intervention*: Intervention type 2 ‐ to focus one’s attention away from the experience of stress *Description of the intervention*: The massage technique applied was effleurage (or smoothing), in the posterior thoracic and cervical region. The aromatherapeutic formula applied to the massage was a neutral cream containing essential oils of Lavandula angustifolia and Pelargonium graveolens in the concentration of 1% each, and totalling 2% of essential oil in the formulation prepared by a professional pharmacist. *The number of sessions*: 6 *Duration of each session on average*: 10 to 5 mins *Duration of the entire intervention*: 1 to 2 weeks *Duration of the entire intervention short vs long*: short *Intervention deliverer*: Massages were performed by the first author, who was previously trained, and by an aromatherapy specialist nurse. *Intervention form*: During morning and afternoon sessions in the rest area of the sector with participants sitting in an armchair and ensuring their privacy Control (no intervention) *Type of the intervention*: NA *Description of the intervention*: NA *The number of sessions*: NA *Duration of each session on average*: NA *Duration of the entire intervention*: NA *Duration of the entire intervention short vs long*: NA *Intervention deliverer*: NA *Intervention form*: NA	
Outcomes	*The List of Stress Symptoms (LSS)* **Outcome type**: ContinuousOutcome *Work stress scale (WSS)* **Outcome type**: ContinuousOutcome	
Identification	**Sponsorship source:** NR **Country:** Brazil **Setting:** A surgical center of a teaching hospital **Comments:** NR **Authors name:** Juliana Montibeler **Institution:** Universidade Estadual Paulista, Faculdade de Medicina de Botucatu, Departamento de Enfermagem **Email:** ju.montibeller.jm@gmail.com **Address:** Rua Dr. José Barbosa de Barros, 1540 – Bloco 2 – Apto 106CEP 18610‐307 – Botucatu, SP, Brazil **Time period:** 2016	
Notes	LSS included in analysis 2.1	
***Risk of bias***		
**Bias**	**Authors' judgement**	**Support for judgement**
Random sequence generation (selection bias)	High risk	Quote: "A draw was made to randomize participants into two groups:"
Allocation concealment (selection bias)	High risk	Quote: "Esc Enferm USP · 2018;52:03348 A draw was made to randomize participants into two groups.
Blinding of participants and personnel (performance bias) All outcomes	High risk	Participants were not blinded.
Blinding of outcome assessment (detection bias) All outcomes	High risk	Participants were not blinded whereas outcomes are self‐reported.
Incomplete outcome data (attrition bias) All outcomes	Low risk	No loss to follow‐up.
Selective reporting (reporting bias)	Unclear risk	No trial registration, nor did we find on online.
Other bias	Low risk	No indication of other bias.

**Table:** Moody 2013a

***Study characteristics***		
Methods	RCT with individual participants, USA	
Participants	Paediatric oncology staff (50% nurses, 20% physicians); Experimental 23 Control 24; Men: Experimental 30% Control 8%; Age: not reported; stated to be equal; Experience > 10 years: Experimental 48% Control 46%	
Interventions	1) Experimental: 8 weeks of didactic and experiential mindfulness education via a structured, skills‐training course delivered in a group setting at their hospital. The course included 1 initial 6‐hour session; 6 weekly 1‐hour follow‐up sessions; and a final 3‐hour wrap‐up session (15 hours total class time) 2) Control: No intervention	
Outcomes	MBI; Perceived Stress Scale	
Identification		
Notes	Authors provided additional data: SDs of MBI at follow‐up: Experimental: EE: 7.67; DP: 3.54; PA 3.69 Control: EE: 6.39; DP: 4.59; PA: 5.27 MBI‐EE included in analysis 1.1	
***Risk of bias***		
**Bias**	**Authors' judgement**	**Support for judgement**
Random sequence generation (selection bias)	Low risk	Using a computer‐generated random numbers schema in blocks of ten
Allocation concealment (selection bias)	Unclear risk	Participants were stratified according to their respective professions and randomised to a mindfulness‐based course (intervention) or no intervention (control)
Blinding of participants and personnel (performance bias) All outcomes	High risk	Participants not blinded.
Blinding of outcome assessment (detection bias) All outcomes	High risk	Participants were not blinded whereas outcomes are self‐reported.
Incomplete outcome data (attrition bias) All outcomes	Low risk	All participants reported outcomes
Selective reporting (reporting bias)	Low risk	All outcomes stated in the 'Methods' section reported.
Other bias	Low risk	We did not find any indications of other sources of bias.

**Table:** Norvell 1987

***Study characteristics***		
Methods	RCT, USA	
Participants	12 respiratory therapists	
Interventions	1) Experimental: stress management programme: 8 weekly group sessions on average 60 minutes and a manual containing homework assignments to be completed between sessions. Topics covered were deep muscle relaxation, cognitive‐behavioural exercises to identify and examine stressful situations, replacing negative thoughts and emotions with adaptive rational cognitions, effective communication skills, social support networks and problem‐solving skills, physical fitness, nutrition and weight management and maintenance of behaviour change. 2) Control: no intervention	
Outcomes	MBI, C‐H Inventory of Phys Symptoms, The Hassles Scale, The Uplifts Scale	
Identification		
Notes	MBI included in analysis 4.1	
***Risk of bias***		
**Bias**	**Authors' judgement**	**Support for judgement**
Random sequence generation (selection bias)	Unclear risk	"The 12 participating employees were randomly assigned to one of two conditions: 6 to an 8‐week stress management programme and 6 to a wait‐list control group." (p. 120)
Allocation concealment (selection bias)	Unclear risk	Not reported
Blinding of participants and personnel (performance bias) All outcomes	High risk	Participants not blinded.
Blinding of outcome assessment (detection bias) All outcomes	High risk	Participants were not blinded whereas outcomes are self‐reported.
Incomplete outcome data (attrition bias) All outcomes	Low risk	There were no dropouts because of small sample size.
Selective reporting (reporting bias)	Low risk	All outcomes reported.
Other bias	Low risk	We did not find any indications of other sources of bias.

**Table:** Novoa 2014

***Study characteristics***		
Methods	**Study design:** randomised controlled trial **Study grouping:** parallel group	
Participants	**Baseline characteristics** Reiki treatment *Age in years (N(%))*: NR *Sex (N (% female))*: NR *Sample size*: 22 *Years of experience (mean ± SD)*: NR Placebo condition *Age in years (N(%))*: NR *Sex (N (% female))*: NR *Sample size*: 21 *Years of experience (mean ± SD)*: NR Control (no intervention) *Age in years (N(%))*: NR *Sex (N (% female))*: NR *Sample size*: 24 *Years of experience (mean ± SD)*: NR Overall *Age in years (N(%))*: 20–30 (35 (53.0%)) / 31–40 (14 (21.2%)) / 41–50 (8 (12.2%)) / > 51 (9 (13.6%)) *Sex (N (% female))*: 62 (93%) *Sample size*: 67 *Years of experience (mean ± SD)*: NR **Included criteria:** inclusion criteria included identification of a moderate to high risk of STS as determined by the Professional Quality of Life scale: Compassion Satisfaction, Burnout, and Compassion Fatigue/Secondary Trauma subscales (ProQOL R‐V; Stamm, 2009) **Excluded criteria:** respondents who were not at moderate to high risk for STS were not included in the study sample. Additional exclusion criteria included having received a Reiki treatment or other energy modality in the past month and pregnancy. **Pretreatment:** the dependent variables did not differ at baseline among the three groups (Reiki, placebo, and control). **Compliance rate:** NR **Response rate:** 85% **Type of healthcare worker:** 51% social work professionals,42% social work student interns, 5% licenced professional counsellors (LPCs).	
Interventions	**Intervention characteristics** Reiki treatment *Type of the intervention*: Intervention type 2 ‐ to focus one’s attention away from the experience of stress *Description of the intervention*: For the treatment group, sessions started with the participant lying on his or her back, fully clothed, with a cloth over the eyes. The practitioner started at the head and worked towards the feet, keeping hands approximately 1.5 to 2.0 inches away from the body. The participant then turned over to the stomach and the practitioner again worked from the head to the feet. *The number of sessions*: four *Duration of each session on average*: 50 minutes *Duration of the entire intervention*: four weeks *Duration of the entire intervention short vs long*: short *Intervention deliverer*: One of the researchers who had twelve years of experience as a Reiki master practitioner. *Intervention form*: The room was a quiet space with no visual or auditory distraction. Placebo condition *Type of the intervention*: NA *Description of the intervention*: After covering the participant’s eyes with a piece of cloth, the practitioner stood next to the table and moved every 2.5 minutes, following the treatment protocol but without the placement of hands. *The number of sessions*: four *Duration of each session on average*: 50 minutes *Duration of the entire intervention*: four weeks *Duration of the entire intervention short vs long*: short *Intervention deliverer*: One of the researchers who had twelve years of experience as a Reiki master practitioner. *Intervention form*: The room was a quiet space with no visual or auditory distractions. Control (no intervention) *Type of the intervention*: NA *Description of the intervention*: NA *The number of sessions*: NA *Duration of each session on average*: NA *Duration of the entire intervention*: NA *Duration of the entire intervention short vs long*: NA *Intervention deliverer*: NA *Intervention form*: NA	
Outcomes	*Professional Quality of Life ‐ Compassion Fatigue/Secondary Trauma* **Outcome type**: ContinuousOutcome **Scale**: ProQOL R‐V; Stamm, 2009 *Symptom Questionnaire (SQ)* **Outcome type**: ContinuousOutcome	
Identification	**Sponsorship source:** NR **Country:** United States **Setting:** One state in the Deep South **Comments:** NR **Authors name:** Martha P. Novoa **Institution:** energy practitioner at the White Horse **Email:** NR **Address:** Baton Rouge, LA **Time period:** Recruitment for the study started in January 2010 and ended in May 2011.	
Notes	Not able to include in analysis due to missing data.	
***Risk of bias***		
**Bias**	**Authors' judgement**	**Support for judgement**
Random sequence generation (selection bias)	Low risk	Quote: "The researchers randomly assigned treatment conditions using three differ‐ently colored pebbles: orange corresponded to Reiki treatment, white corre‐sponded to the placebo condition, and blue corresponded to the control group. Thirty‐three pebbles of each color were put in a paper bag and mixed."
Allocation concealment (selection bias)	High risk	Quote: "The researcher pulled a random pebble from the bag and the participant was assigned to treatment according to the colour of the pebble. Once the pebble had been selected it was discarded." At the end of the randomisation the researcher could possibly foresee assignment as not many pebbles were left.
Blinding of participants and personnel (performance bias) All outcomes	Low risk	Participants were blinded to treatment condition.
Blinding of outcome assessment (detection bias) All outcomes	Low risk	Participants were blinded to treatment condition and outcomes are self‐reported.
Incomplete outcome data (attrition bias) All outcomes	Unclear risk	Not recorded.
Selective reporting (reporting bias)	Unclear risk	No trial registration, no indication of selective reporting.
Other bias	Unclear risk	Compliance rate was not reported.

**Table:** OBrien 2019

***Study characteristics***		
Methods	**Study design:** randomised controlled trial **Study grouping:** parallel group	
Participants	**Baseline characteristics** Acceptance and Commitment Therapy *Age in years (mean ± SD)*: NR *Sex (N (% female))*: NR *Sample size*: 37 *Years of experience (mean ± SD)*: NR Control (wait list) *Age in years (mean ± SD)*: NR *Sex (N (% female))*: NR *Sample size*: 34 *Years of experience (mean ± SD)*: NR Overall *Age in years (mean ± SD)*: 37.91 ± 13.24 *Sex (N (% female))*: 61 (86%) *Sample size*: 71 *Years of experience (mean ± SD)*: 11.97 ± 9.86 **Included criteria:** NR **Excluded criteria:** NR **Pretreatment:** there were also no significant between‐group differences on any demographic measure at baseline or follow‐up (all P‐values > 0.20) **Compliance rate:** 87% **Response rate:** NR **Type of healthcare worker:** Nurses and nurse aides	
Interventions	**Intervention characteristics** Acceptance and Commitment Therapy *Type of the intervention*: Intervention type 4 ‐ Combination of two or more of the above *Description of the intervention*: The intervention topics included acceptance, mindfulness, psychological flexibility, willingness to experience discomfort, present‐moment focus, self‐as‐context, values identification, and values‐congruent committed action. *The number of sessions*: 2 *Duration of each session on average*: 2.5 h *Duration of the entire intervention*: 1 week *Duration of the entire intervention short vs long*: short *Intervention deliverer*: Faculty and trained graduate students pro‐vided the ACT intervention. All graduate student therapists completed a semester‐long seminar in the research, theory, and application of mindfulness and ACT therapies. Experiential training was also provided to the therapists in the Bowling Green State University psychology clinic. *Intervention form*: at participant work sites, the average group size per session was three with a maximum of seven per group. Control (wait list) *Type of the intervention*: NA *Description of the intervention*: NA *The number of sessions*: NA *Duration of each session on average*: NA *Duration of the entire intervention*: NA *Duration of the entire intervention short vs long*: NA *Intervention deliverer*: NA *Intervention form*: NA	
Outcomes	*General Health Questionnaire (GHQ‐12)* **Outcome type**: ContinuousOutcome	
Identification	**Sponsorship source:** This project was supported by a research grant provided by the Ohio Bureau Workers Compensation Ohio Occupational Safety and Health Research Program. **Country:** United States **Setting:** Nursing homes and assisted living facilities **Comments:** NR **Authors name:** William H. O’Brien **Institution:** Department of Psychology, Bowling Green State University **Email:** wobrien@bgsu.edu **Address:** Bowling Green, OH 43403 **Time period:** NR	
Notes	GHQ included in analysis 4.1	
***Risk of bias***		
**Bias**	**Authors' judgement**	**Support for judgement**
Random sequence generation (selection bias)	Unclear risk	Interested participants were randomly assigned to the treatment or control group and then contacted by the project coordinators. An assessment session was then scheduled. Randomisation sequence generation process not described
Allocation concealment (selection bias)	Unclear risk	Insufficient information to understand whether intervention allocations could have been foreseen in advance of, during, enrolment.
Blinding of participants and personnel (performance bias) All outcomes	High risk	Quote: "participants were informed that they were either in the treatment group or wait‐list control group."
Blinding of outcome assessment (detection bias) All outcomes	High risk	Quote: "This was described to them as the immediate treatment group or the delayed treatment group." Participants were not blinded whereas outcomes are self‐reported.
Incomplete outcome data (attrition bias) All outcomes	Low risk	Quote: "There were no signiﬁcant differences in dropout rates between the ACT and control groups (Fisher’s exact test p = 0.36)."
Selective reporting (reporting bias)	Unclear risk	No trial registration, nor did we find one online.
Other bias	Unclear risk	Response rate was not reported.

**Table:** Oman 2006

***Study characteristics***		
Methods	RCT, USA	
Participants	58 staff members (64% nurses, 12% physicians and 24% other) of a large hospital	
Interventions	1) Experimental: Eight‐Point Program Spiritual Skills Training: 8 weekly 2‐hour training sessions about meditation skills (passage meditation, mantram repetition, slowing down, focused attention, training the senses, putting others first, spiritual association and inspirational reading). 2) Control: no intervention	
Outcomes	Perceived Stress Scale, MBI, Medical Outcomes Study	
Identification		
Notes	Included in analysis 2.2	
***Risk of bias***		
**Bias**	**Authors' judgement**	**Support for judgement**
Random sequence generation (selection bias)	Low risk	Details of randomisation were provided in a separate supplement to the article: "While participants were completing pretests, individuals were randomly assigned to treatment (n = 30) or wait‐list control (n = 31). Computer pre‐generated 1:1 random assignment tables had been prepared by the lead investigator for each potential number of registering participants, up to 60, for each session (precise number of registrants could not be anticipated). At each session, as participants completed pretests, their consent forms were rapidly assembled in an arbitrary order and given sequential numbers by the lead investigator or the main instructor. The total number of received consent forms dictated the appropriate random assignment table, which dictated how to separate the numbered consent forms into two groups." (p. S4)
Allocation concealment (selection bias)	Unclear risk	"Immediately following pretest, participants were informed of their group assignment. One or two weeks later, those in the treatment condition began the eight‐week training, meeting together in one large group." (p. S4)
Blinding of participants and personnel (performance bias) All outcomes	High risk	Participants not blinded.
Blinding of outcome assessment (detection bias) All outcomes	High risk	Participants were not blinded whereas outcomes are self‐reported.
Incomplete outcome data (attrition bias) All outcomes	Low risk	"Selected characteristics of the 58 final participants included in the intention‐to‐treat analysis are displayed in Table 1" (p. 715)
Selective reporting (reporting bias)	Low risk	All outcomes reported.
Other bias	Unclear risk	We did not find any indications of other sources of bias.

**Table:** Ozbas 2016

***Study characteristics***		
Methods	**Study design:** randomised controlled trial **Study grouping:** parallel group	
Participants	**Baseline characteristics** Psychological empowerment program *Age in years (category (N (%)))*: 18‐27 (11 (29%)) ‐ 28‐37 (22 (58%)) ‐ 38+ (5 (13%)) *Sex (N (% female))*: NR *Sample size*: 38 *Years of experience (category (N (%)))*: 0‐5n(12n(32%)) ‐ 6‐10 (14 (37%)) ‐ 11+(12 (32%)) Control (no intervention) *Age in years (category (N (%)))*: 18‐27 (21 (48%)) ‐ 28‐37 (23 (52%)) ‐ 38+ (0 (0%)) *Sex (N (% female))*: NR *Sample size*: 44 *Years of experience (category (N (%)))*: 0‐5 (28 (64%)) ‐ 6‐10 (11 (25%)) ‐ 11+ (5 (11%)) Overall *Age in years (category (N (%)))*: 18‐27 (32 (40%)) ‐ 28‐37 (45 (55%)) ‐ 38+ (5 (6%)) *Sex (N (% female))*: NR *Sample size*: 82 *Years of experience (category (N (%)))*: 0‐5 (40 (49%)) ‐ 6‐10 (25 (31%)) ‐ 11+ (17 (21%)) **Included criteria:** scored less than 17 on the Beck Depression Inventory and did not have any diagnosed physical or mental disease. **Excluded criteria:** NR **Pretreatment:** NR **Type of healthcare worker:** exclusively nurses **Response rate:** 86% **Compliance rate:** 83%	
Interventions	**Intervention characteristics** Psychological empowerment program *Type of the intervention*: Intervention type 4 ‐ Combination of two or more of the above *Description of the intervention*: In every session, participants were given a module related to a particular scenario and asked to act it out extemporaneously. After every session, participant feedback was offered, and their awareness about the style of discussing the theme by themselves and with other group members, communication patterns and emotions, ideas and behaviours were registered. Program sessions included a time for getting acquainted and for an introduction to the program. Discussions about the group contract, coping with stress, and cognitive distortion were held. Relaxation techniques were also taught, along with problem‐solving, self‐recognition, empathy, and dispute resolution *The number of sessions*: 10 *Duration of each session on average*: 2 hours *Duration of the entire intervention*: 10 weeks *Duration of the entire intervention short vs long*: short *Intervention deliverer*: The first author, who conducted all the psychodrama intervention sessions and is a certified psychodramatist *Intervention form*: Group Control (no intervention) *Type of the intervention*: NA *Description of the intervention*: NA *The number of sessions*: NA *Duration of each session on average*: NA *Duration of the entire intervention*: NA *Duration of the entire intervention short vs long*: NA *Intervention deliverer*: NA *Intervention form*: NA	
Outcomes	*Maslach Burnout Inventory ‐ Emotional exhaustion* **Outcome type**: ContinuousOutcome *Maslach Burnout Inventory ‐ Desensitization* **Outcome type**: ContinuousOutcome *Maslach Burnout Inventory ‐ Personal achievement* **Outcome type**: ContinuousOutcome	
Identification	**Sponsorship source:** NR **Country:** Turkey **Setting:** Adult inpatient oncology clinics **Comments:** NR **Authors name:** Azize AtliO ¨zbas **Institution:** Hacettepe University Nursing Faculty, Psychiatric Nursing Department, Hacettepe Universitesi Hems ¸irelik Faku ¨ ltesi **Email:** azeozbas@gmail.com **Address:** 06100 Sıhhiye, Ankara, Turkey. **Time period:** NR	
Notes	MBI‐EE included in analysis 4.1	
***Risk of bias***		
**Bias**	**Authors' judgement**	**Support for judgement**
Random sequence generation (selection bias)	Low risk	Quote: "Using a computerized black‐box randomization assignment program,"
Allocation concealment (selection bias)	Unclear risk	Insufficient information to understand whether intervention allocations could have been foreseen in advance of, during, enrolment.
Blinding of participants and personnel (performance bias) All outcomes	High risk	Participants were not blinded.
Blinding of outcome assessment (detection bias) All outcomes	High risk	Participants were not blinded whereas outcomes are self‐reported.
Incomplete outcome data (attrition bias) All outcomes	Low risk	Quote: "Some 8 nurses dropped out of the intervention group and 1 dropped of the control group," Nine of 82 randomised (11%) nurses were lost to follow‐up. This is below our pre‐defined cut‐off value.
Selective reporting (reporting bias)	Unclear risk	No trial registration, nor did we find one online.
Other bias	Low risk	No indication of other bias.

**Table:** Ozgundondu 2019

***Study characteristics***		
Methods	**Study design:** randomised controlled trial **Study grouping:** parallel group	
Participants	**Baseline characteristics** Progressive muscle relaxation combined with music *Age in years (mean ± SD)*: 24.61 ± 2.61 *Sex (N (% female))*: 28 (100%) *Sample size*: 28 *Years of experience (category (n (%)))*: 6 to 23 months (16 (57.1%)) ‐ 24 months+ (12 (42.9 %)) Control (no intervention) *Age in years (mean ± SD)*: 27.75 ± 4.75 *Sex (N (% female))*: 28 (100%) *Sample size*: 28 *Years of experience (category (n (%)))*: 6 to 23 months (10 (35.7%)) ‐ 24 months+ (18 (64.3 %)) Overall *Age in years (mean ± SD)*: NR *Sex (N (% female))*: 56 (100%) *Sample size*: 56 *Years of experience (category (n (%)))*: NR **Included criteria:** the eligible nurses included those who (a) were 18 years old, (b) had an experience of at least three months in ICUs, and (c) had no documented history of chronic obstructive pulmonary disease, heart failure and asthma. **Excluded criteria:** the exclusion criteria were as follows: (a) history of severe psychiatric disorder, (b) ICU experience of less than three months, and (c) not currently using any complementary therapy modalities such as acupuncture, massage therapy, relaxation techniques, and yoga that can be influential on perceived stress and fatigue or coping styles. **Pretreatment:** the study groups were homogeneous in terms of age, gender, marital status, educational level, income status, living with either their family or friends, comorbid conditions, experience in ICU, working hours per week and satisfaction levels with ICUs (P > 0.05). **Compliance rate:** 90% **Response rate:** 89% **Type of healthcare worker:** exclusively nurses	
Interventions	**Intervention characteristics** Progressive muscle relaxation combined with music *Type of the intervention*: Intervention type 2 ‐ to focus one’s attention away from the experience of stress *Description of the intervention*: The nurses who were grouped in the intervention group were provided with a booklet containing the definition, purpose, benefits, and application techniques of PMR and music therapy. The participants were first taught the description, using fields and effects on the body of the PMR plus music therapy. Subsequently, step‐by‐step instructions on PMR were provided by the PI. After the demonstration by PI, all the PMR steps were practised by the participants under the supervision of PI within the duration of 20 minutes, following which, PMR booklets prepared by the researchers were delivered to the nurses in the training session itself. *The number of sessions*: 8 *Duration of each session on average*: 20 minutes *Duration of the entire intervention*: 8 weeks *Duration of the entire intervention short vs long*: short *Intervention deliverer*: The principal investigator (PI), who had work experience in relaxation and music therapy *Intervention form*: group sessions, which composed of 10–15 participants Control (no intervention) *Type of the intervention*: NA *Description of the intervention*: a single‐time face‐to‐face attention‐matched educational session, on the first day of the study and no additional intervention was conducted for this group during the whole study period. Was performed with a booklet containing the causes, negative effects of stress and fatigue on the body, and techniques for coping with stress *The number of sessions*: 1 *Duration of each session on average*: 20 minutes *Duration of the entire intervention*: NA *Duration of the entire intervention short vs long*: NA *Intervention deliverer*: NA *Intervention form*: face‐to‐face; 10–15 participants; a silent room located in the hospital.	
Outcomes	*Perceived Stress Scale (PSS)* **Outcome type**: ContinuousOutcome	
Identification	**Sponsorship source:** The author(s) received no financial support for the research, authorship, and/or publication of this article **Country:** Turkey **Setting:** University of Health Sciences, Ankara Gulhane Training and Research Hospital **Comments:** NR **Authors name:** Zehra Gok Metin **Institution:** Hacettepe University **Email:** zehragok85@hotmail.com **Address:** Hacettepe University, Faculty of Nursing, 06000 Sihhiye, Ankara, Turkey. **Time period:** were recruited from 1 July 2018 to 15 January 2019.	
Notes	We kindly received the mean and SD from the author. PSS included in analysis 2.1	
***Risk of bias***		
**Bias**	**Authors' judgement**	**Support for judgement**
Random sequence generation (selection bias)	Low risk	Quote: "were assigned randomly into two groups (Group A: 31; Group B: 32) through lottery method (A: Control; B: Intervention)"
Allocation concealment (selection bias)	Low risk	Quote: "by the second author of the present report who was not involved in the intervention procedures, and the PI informed the nurses about the randomisation results."
Blinding of participants and personnel (performance bias) All outcomes	High risk	Quote: "All the sessions comprising PMR combined with music were also conducted by the PI who was not blinded to the study groups due to the nature of PMR. ,"
Blinding of outcome assessment (detection bias) All outcomes	High risk	Participants were not blinded whereas outcomes are self‐reported.
Incomplete outcome data (attrition bias) All outcomes	Low risk	No loss to follow‐up
Selective reporting (reporting bias)	Unclear risk	No trial registration, nor did we find one online.
Other bias	Low risk	No indication of other bias.

**Table:** Palumbo 2012

***Study characteristics***		
Methods	RCT, USA	
Participants	14 registered or licenced practical female nurses aged 49 years and older who were currently employed at an academic medical centre full‐time or part‐time in a staff nurse position that involved lifting patients.	
Interventions	1) Experimental: Tai Chi: onsite Tai Chi classes once a week and to practise on their own for 10 minutes each day at least four days per week for 15 weeks. Each Tai Chi class lasted 45 minutes, with 10 minutes of breathing exercises, followed by 30 minutes of Tai Chi practice, and ended with 5 minutes of visualisation and cool‐down exercises. 2) Control: o intervention	
Outcomes	Nursing Stress Scale, Perceived Stress Scale	
Identification	Not able to include in analysis due to missing data.	
Notes		
***Risk of bias***		
**Bias**	**Authors' judgement**	**Support for judgement**
Random sequence generation (selection bias)	Unclear risk	Not reported.
Allocation concealment (selection bias)	Unclear risk	Not reported.
Blinding of participants and personnel (performance bias) All outcomes	High risk	Participants not blinded.
Blinding of outcome assessment (detection bias) All outcomes	High risk	Participants were not blinded whereas outcomes are self‐reported.
Incomplete outcome data (attrition bias) All outcomes	High risk	3/14 participants dropped out. No imputation of data.
Selective reporting (reporting bias)	Unclear risk	Only change values were reported.
Other bias	Low risk	We did not find any indications of other sources of bias.

**Table:** Pehlivan 2020

***Study characteristics***		
Methods	**Study design:** randomised controlled trial **Study grouping:** parallel group	
Participants	**Baseline characteristics** Compassion fatigue resiliency I *Age in years (mean ± SD)*: 25.0 ± 5.1 *Sex (N (% female))*: 44 (90%) *Sample size*: 34 *Years of experience (mean ± SD)*: 3.0 ± 3.7 Compassion fatigue resiliency II *Age in years (mean ± SD)*: 27.8 ± 5.3 *Sex (N (% female))*: 32 (94%) *Sample size*: 49 *Years of experience (mean ± SD)*: 5.6 ± 5.7 Control (no intervention) *Age in years (mean ± SD)*: 27.2 ± 5.3 *Sex (N (% female))*: 36 (86%) *Sample size*: 42 *Years of experience (mean ± SD)*: 4.9 ± 4.7 Overall *Age in years (mean ± SD)*: NR *Sex (N (% female))*: 112 (90%) *Sample size*: 125 *Years of experience (mean ± SD)*: NR **Included criteria:** nurses working in inpatient oncology–haematology, outpatient chemotherapy, or BMT unit **Excluded criteria:** providing care for paediatric oncology patients, being a nurse manager, not providing direct patient care **Pretreatment:** there was a statistically significant difference between the groups regarding age, total work hours per week, total years of professional experience, total years of oncology experience, level of received social support, and marital status (P < 0.05). The comparison of the mean scores for nurses’ compassion satisfaction P = 0.036 **Compliance rate:** 100% **Response rate:** NR **Type of healthcare worker:** nurses	
Interventions	**Intervention characteristics** Compassion fatigue resiliency I *Type of the intervention*: Intervention type 1 ‐ to focus one’s attention on the experience of stress *Description of the intervention*: Explain the historical development of compassion fatigue among caregivers, Define the developmental process of compassion fatigue, specify the risk factors for compassion fatigue, Explain the symptoms of compassion fatigue, Raise awareness about their personal history, Explain the concept of stress and its impact on the body, Apply compassion fatigue resilience skills acquired in the program, Professionally create a self‐directed resilience plan. *The number of sessions*: 2 *Duration of each session on average*: 5 hours *Duration of the entire intervention*: 2 days *Duration of the entire intervention short vs long*: short *Intervention deliverer*: study's principal investigator had participated online in a CFRP, developed by Eric Gentry (Licensed Mental Health Counsellor) (2002), and received the certificate, and then conducted the pro‐gram with the nurses *Intervention form*: Meetings were held with each institution's directorate of nursing services to determine the training schedules and content. The schedules were planned in accordance with the hospital administration's preferences and by taking nurses’ busy schedules into consideration Compassion fatigue resiliency II *Type of the intervention*: Intervention type 1 ‐ to focus one’s attention on the experience of stress *Description of the intervention*: Explain the historical development of compassion fatigue among caregivers, Define the developmental process of compassion fatigue, Specify the risk factors for compassion fatigue, Explain the symptoms of compassion fatigue, Raise awareness about their personal history, Explain the concept of stress and its impact on the body, Apply compassion fatigue resilience skills acquired in the program, Professionally create a self‐directed resilience plan. *The number of sessions*: 5 *Duration of each session on average*: 2 hours *Duration of the entire intervention*: 5 weeks *Duration of the entire intervention short vs long*: short *Intervention deliverer* : study's principal investigator had participated online in a CFRP, developed by Eric Gentry (Licensed Mental Health Counsellor) (2002), and received the certificate, and then conducted the pro‐gram with the nurses *Intervention form*: Meetings were held with each institution's directorate of nursing services to determine the training schedules and content. The schedules were planned in accordance with the hospital administration's preferences and by taking nurses’ busy schedules into consideration Control (no intervention) *Type of the intervention*: NA *Description of the intervention*: NA *The number of sessions*: NA *Duration of each session on average*: NA *Duration of the entire intervention*: NA *Duration of the entire intervention short vs long*: NA *Intervention deliverer*: NA *Intervention form*: NA	
Outcomes	*Professional quality of life scale‐IV ‐ Compassion fatigue* **Outcome type**: ContinuousOutcome *Professional quality of life scale‐IV ‐ Burnout* **Outcome type**: ContinuousOutcome *Professional quality of life scale‐IV ‐ Compassion satisfaction* **Outcome type**: ContinuousOutcome *Perceived Stress Scale (PSS)* **Outcome type**: ContinuousOutcome	
Identification	**Sponsorship source:** This research received no specific grant from any funding agency in the public, commercial, or not‐for‐profit sectors **Country:** Turkey **Setting:** Oncology–haematology inpatient services, outpatient chemotherapy units, and bone marrow transplant (BMT) units of three private hospitals in Istanbul **Comments:** NR **Authors name:** Tuğba Pehlivan **Institution:** Koç University Hospital **Email:** tpehlivan14@ku.edu.tr **Address:** Istanbul, Turkey. **Time period:** January 2017 January 2019	
Notes	We kindly got the mean and SD for the primary outcome from author T. Pehlivan. PSS included in analysis 1.1, 1.2 and 1.3. Intervention groups combined to create a single pair‐wise comparison	
***Risk of bias***		
**Bias**	**Authors' judgement**	**Support for judgement**
Random sequence generation (selection bias)	Unclear risk	Quote: "Nurses were randomly assigned to the Experimental I, Experimental II, or control group" Sequence generation process insufficiently described.
Allocation concealment (selection bias)	Unclear risk	Difficult to judge whether participants and/or investigators could possible foresee assignment.
Blinding of participants and personnel (performance bias) All outcomes	High risk	Participants were not blinded.
Blinding of outcome assessment (detection bias) All outcomes	High risk	Participants were not blinded whereas outcomes are self‐reported.
Incomplete outcome data (attrition bias) All outcomes	High risk	Low number of participants that filled in the one‐year follow‐up questionnaire. Lost to follow‐up 57/125 (45%). Reasons provided. Not sure whether loss to follow‐up was at random.
Selective reporting (reporting bias)	Low risk	Outcomes reported according to trial registration.
Other bias	Unclear risk	Not able to assess response rate.

**Table:** PelitAksu 2020

***Study characteristics***		
Methods	**Study design:** randomised controlled trial **Study grouping:** parallel group	
Participants	**Baseline characteristics** Progressive muscle relaxation exercise (PMRE) *Age (mean ± SD)*: 21.9 ± 0.84 *Sex (N (% female))*: NR (87%) *Sample size*: 100 *Years of experience (mean ± SD)*: NA Control (no intervention) *Age (mean ± SD)*: 22.07 ± 0.86 *Sex (N (% female))*: NR (83%) *Sample size*: 100 *Years of experience (mean ± SD)*: NA Overall *Age (mean ± SD)*: NR *Sex (N (% female))*: NR *Sample size*: 200 *Years of experience (mean ± SD)*: NA **Included criteria:** sample inclusion criteria were as follows: Not having a disease that prevents students from doing PMR, such as metabolic diseases, cancer, heart disease, and diabetes, and not receiving antidepressant treatment, not doing any other mind–body exercises and using a smartphone **Excluded criteria:** NR **Pretreatment:** there were no statistically significant differences between the groups’ age and gender (P > 0.05) **Compliance rate:** 16 of the 100 less than 4 days (16%) **Response rate:** 100% **Type of healthcare worker:** exclusively student nurse interns	
Interventions	**Intervention characteristics** Progressive muscle relaxation exercise (PMRE) *Type of the intervention*: Intervention type 2 ‐ to focus one’s attention away from the experience of stress *Description of the intervention*: Guided by verbal directions (The muscle relaxation CDproduced by the Turkish Psychologists Association), the students tensed and relaxed the muscle groups in hands, arms, neck, shoulder, chest, stomach, hips, foot and fingers, and all muscles in the face and body, in sequence. During the PMRE, the students listened to the audio instructions and applied the exercises exactly according to this audio instructions. In the meantime, the researcher observed the students and gave feedback by making the necessary corrections at the end of the exercise. The researcher did not interfere with the original version of the exercise. After the first session, the students were asked to perform the PMRE before sleeping, at least 4 days each week, every day if possible, for 3 weeks. At the end of the first session, a Whatsapp® group was created, and to follow‐up and motivate the students, they were asked to send a message to the group after each PMRE practice. The verbal direction was installed on the students’ smartphones, and the first meeting was concluded. *The number of sessions*: At least 4 days each week *Duration of each session on average*: 30 min *Duration of the entire intervention*: 3 weeks *Duration of the entire intervention short vs long*: Short *Intervention deliverer*: Experienced nurse with her PhD on progressive muscle relaxation technique *Intervention form*: Group Control (no intervention) *Type of the intervention*: NA *Description of the intervention*: After filling out the instruments during the first meeting in the classroom, the CG received no intervention. *The number of sessions*: NA *Duration of each session on average*: NA *Duration of the entire intervention*: NA *Duration of the entire intervention short vs long*: NA *Intervention deliverer*: NA *Intervention form*: NA	
Outcomes	*Burnout Measure Short Version* **Outcome type**: ContinuousOutcome	
Identification	**Sponsorship source:** This study did not receive any specific grant from funding agencies in the public, commercial, or not‐for‐profit sectors **Country:** Turkey **Setting:** Gynecology and obstetrics clinics **Comments:** NR **Authors name:** Sıdıka Pelit‐Aksu **Institution:** Department of Nursing, Faculty of HealthSciences, Gazi University, Ankara, Turkey **Email:** sidikapelit@hotmail.com **Address:** Sıdıka Pelit‐Aksu, MSN, RN, Department ofNursing, Faculty of Health Sciences, GaziUniversity, Emek mah. Bişkek Cad. 6. Cad.(eski 81. sokak) No:2 06490 Çankaya, Ankara, Turkey **Time period:** 2018–2019	
Notes	Burnout Measure Short Version included in analysis 2.1.	
***Risk of bias***		
**Bias**	**Authors' judgement**	**Support for judgement**
Random sequence generation (selection bias)	Unclear risk	Quote: "All of the students who met the inclusion criterias were grouped randomly using simple randomization method. Students names were selected from course attendance sheet of the internship and they were assigned simultaneously and sequentially to experimental group (EG = 100) and control group (CG = 100)." Sequence generation process insufficiently described
Allocation concealment (selection bias)	Low risk	Quote: "Students names were selected from course attendance sheet of the internship and they were assigned simultaneously and sequentially to experimental group (EG = 100) and control group (CG = 100)."
Blinding of participants and personnel (performance bias) All outcomes	High risk	Participants were not blinded.
Blinding of outcome assessment (detection bias) All outcomes	High risk	Participants were not blinded whereas outcomes are self‐reported.
Incomplete outcome data (attrition bias) All outcomes	High risk	55 of the 200 randomised participants were lost to follow‐up (28%). Not mentioned whether loss to follow‐up was at random.
Selective reporting (reporting bias)	Unclear risk	No trial registration or no study protocol reported, nor did we find one online.
Other bias	Unclear risk	Quote: "and the information leakage from experimental groups to the control groups may have affected the results of the study."

**Table:** Peterson 2008

***Study characteristics***		
Methods	RCT, Sweden	
Participants	131 healthcare workers who scored above the 75th percentile on the exhaustion dimension of the Oldenburg Burnout Inventory	
Interventions	1) Experimental: reflecting peer‐support group: Ten 2‐hour weekly sessions where participants discussed and reflected with colleagues about work‐related stress and burnout, provided mutual support for each other, compared experiences and set individual goals to find out alternative ways to handle perceived stressful situations. The sessions started with a short 10‐minute guided relaxation. 2) Control: no intervention	
Outcomes	The General Nordic Questionnaire for Psychological and Social Factors at Work (QPS Nordic), Oldenburg Burnout Inventory, The Hospital Anxiety and Depression Scale, The Short Form Health Survey (SF‐36)	
Identification		
Notes	OBI included in analysis 3.2 and the HADS in analysis 3.4	
***Risk of bias***		
**Bias**	**Authors' judgement**	**Support for judgement**
Random sequence generation (selection bias)	Low risk	Quote: "The randomization procedure was performed by a statistician using the Statistical Analysis Software, version 8.2." (p. 508)
Allocation concealment (selection bias)	Unclear risk	Not reported
Blinding of participants and personnel (performance bias) All outcomes	High risk	Participants not blinded.
Blinding of outcome assessment (detection bias) All outcomes	High risk	Participants were not blinded whereas outcomes are self‐reported.
Incomplete outcome data (attrition bias) All outcomes	High risk	Altogether 27% of participants dropped out during follow‐up and the reasons for the control group participants leaving were not known
Selective reporting (reporting bias)	Low risk	All outcomes reported.
Other bias	Low risk	We did not find any indications of other sources of bias.

**Table:** Prado 2018

***Study characteristics***		
Methods	**Study design:** randomised controlled trial **Study grouping:** parallel group	
Participants	**Baseline characteristics** Auriculotherapy *Age (mean ± SD)*: NR *Sex (N (% female))*: 40 (93%) *Sample size*: 43 *Years of experience (mean ± SD)*: NR Placebo *Age (mean ± SD)*: NR *Sex (N (% female))*: 44 (94%) *Sample size*: 47 *Years of experience (mean ± SD)*: NR Control (wait list) *Age (mean ± SD)*: NR *Sex (N (% female))*: 42 (98%) *Sample size*: 43 *Years of experience (mean ± SD)*: NR Overall *Age (mean ± SD)*: 35 ± 8.4 *Sex (N (% female))*: 126 (95%) *Sample size*: 133 *Years of experience (mean ± SD)*: NR **Included criteria:** The sample consisted of 168 nurses who presented a stress score between 40 and 110 points on the List of Stress Symptoms (LSS). Since few people presented very high stress level, the level was limited to medium and high in order to obtain homogeneous samples. Other inclusion criteria were voluntary participation and availability for the auriculotherapy sessions **Excluded criteria:** NR **Pretreatment:** No socio‐demographic NR for primary outcomes. **Compliance rate:** NR **Response rate:** NR appears to be 100% **Type of healthcare worker:** exclusively nurses	
Interventions	**Intervention characteristics** Auriculotherapy *Type of the intervention*: Intervention type 2 ‐ to focus one’s attention away from the experience of stress *Description of the intervention*: Two points with calming properties were used, the Shenmen point and the Brainstem *The number of sessions*: 12 sessions *Duration of each session on average*: NR *Duration of the entire intervention*: 6 weeks *Duration of the entire intervention short vs long*: short *Intervention deliverer*: NR *Intervention form*: NR Placebo *Type of the intervention*: Intervention type 2 ‐ to focus one’s attention away from the experience of stress *Description of the intervention*: The sham points chosen were External Ear and Face Area *The number of sessions*: 12 sessions *Duration of each session on average*: NR *Duration of the entire intervention*: 6 weeks *Duration of the entire intervention short vs long*: short *Intervention deliverer*: NR *Intervention form*: NR Control (wait list) *Type of the intervention*: NA *Description of the intervention*: NA *The number of sessions*: NA *Duration of each session on average*: NA *Duration of the entire intervention*: NA *Duration of the entire intervention short vs long*: NA *Intervention deliverer*: NA *Intervention form*: NA	
Outcomes	*The List of Stress Symptoms (LSS)* **Outcome type**: ContinuousOutcome	
Identification	**Sponsorship source:** NR **Country:** Brazil **Setting:** Hospital **Comments:** NR **Authors name:** Juliana Miyuki do Prado **Institution:** Universidade de São Paulo, Escola de Enfermagem, São Paulo, SP, Brazil **Email:** fumieibez@gmail.com **Address:** Leonice Fumiko Sato Kurebayashi Rua Vieira Fazenda, 80, Vila MarianaCEP 04117‐030 – São Paulo, SP, Brazil **Time period:** 2014	
Notes	LSS included in analysis 2.1	
***Risk of bias***		
**Bias**	**Authors' judgement**	**Support for judgement**
Random sequence generation (selection bias)	Low risk	Quote: "From the inclusion criteria, the participants were randomized to the three groups by the Random Allocation Software, each group receiving 56 participants. Thirty‐five people left the study. "
Allocation concealment (selection bias)	Unclear risk	Quote: "From the inclusion criteria, the participants were randomized to the three groups by the Random Allocation Software, each group receiving 56 participants. Thirty‐five people left the study. " Difficult to judge whether participants and/or investigators could possibly foresee assignment
Blinding of participants and personnel (performance bias) All outcomes	Low risk	Quote: "This is a randomized, single‐blind, controlled trial with three groups: experimental auriculotherapy (with points indicated for stress), sham auriculotherapy (with sham points), and control group (without any treatment). The study was conducted with nurses of the Hospital Beneficência Portuguesa in São Paulo, São Joaquim Unit, in 2014. The control group without intervention was a waiting list, and the participants of this group later received the auriculotherapy intervention for the same time and number of sessions." Participants were blinded.
Blinding of outcome assessment (detection bias) All outcomes	Low risk	Participants were blinded and outcomes are self‐reported.
Incomplete outcome data (attrition bias) All outcomes	High risk	Judgement Comment: 35 of the 168 randomised participants were lost to follow‐up (21%). Not reported whether lost to follow‐up was at random. Reasons for lost to follow‐up provided.
Selective reporting (reporting bias)	Unclear risk	No trial registration or study protocol reported, nor did we find one online.
Other bias	Unclear risk	Compliance rate and response rate difficult to assess.

**Table:** Redhead 2011

***Study characteristics***		
Methods	RCT, UK	
Participants	42 nurses working in a low‐secure mental health unit (LSU). Inclusion criteria: working on the LSU for a minimum of 35 hours and having direct contact with service users. Exclusion criteria: having been previously trained in Psychosocial Intervention. Quote: "A total of 79 nursing staff worked on the LSU. Forty‐two (58%) volunteered to participate in the study and provided informed consent. Of the remaining 37 staff, none actively refused, but eight were on sick leave, 23 were unable to be released by their managers to attend the training and six were excluded as they had previously been trained in PSI." (p. 61) Quote"There were no significant differences at baseline between the experimental and control groups in terms of age, gender, clinical area or qualification." (p. 62)	
Interventions	1) Experimental: psychosocial intervention training (12 qualified and 10 unqualified nurses). Quote: "Nurses allocated to the experimental group attended a PSI [Psychosocial Intervention] training programme which was delivered in a meeting room within the LSU. As the learning outcomes for qualified and unqualified staff were different, they were trained on separate courses. The training programme for qualified staff consisted of 16 half‐day sessions delivered over 8 months. The content covered a broad range of PSI including cognitive behavioural approaches for managing symptoms..." " The training for unqualified staff was delivered in 8 half‐day sessions and focused on understanding symptom related behaviours, relationship formation and helping services users to cope with symptoms..." "Teaching sessions were supplemented by small group supervision..." (p. 61) 2) Control: no intervention control (9 qualified and 11 unqualified nurses)	
Outcomes	The MBI	
Identification		
Notes	From reference list: Doyle 2007 ‐ check for inclusion MBI‐EE included in analysis 3.2	
***Risk of bias***		
**Bias**	**Authors' judgement**	**Support for judgement**
Random sequence generation (selection bias)	Unclear risk	Quote: "A randomized controlled design was adopted with nurses who volunteered to participate being allocated to either the experimental PSI training group or a waiting list control group." (p. 60) Random sequence generation process insufficiently described.
Allocation concealment (selection bias)	Unclear risk	Not reported if group allocation was concealed
Blinding of participants and personnel (performance bias) All outcomes	High risk	Participants not blinded.
Blinding of outcome assessment (detection bias) All outcomes	High risk	Participants were not blinded whereas outcomes are self‐reported.
Incomplete outcome data (attrition bias) All outcomes	Unclear risk	Apparently no participants were lost to follow‐up
Selective reporting (reporting bias)	Low risk	The authors report all results for outcome measures listed in the Methods section
Other bias	Unclear risk	We did not find any indications of other sources of bias

**Table:** Reynolds 1993

***Study characteristics***		
Methods	RCT, UK	
Participants	62 health service workers	
Interventions	1) Experimental: Stress Management Training (SMT): 6 weekly 2‐hour sessions of didactic learning, practice of techniques, group exercises and discussion. Topics covered were: nature, signs, causes and symptoms of stress, progressive muscular relaxation, relationship difficulties at home and work, assertiveness techniques, cognitive appraisal and reappraisal, time‐management and goal‐setting skills and emotions and seeking social support. 2) Control: no intervention	
Outcomes	General Health Questionnaire (GHQ‐12) used to obtain a score labelled psychological distress	
Identification		
Notes	GHQ included in analysis 4.1	
***Risk of bias***		
**Bias**	**Authors' judgement**	**Support for judgement**
Random sequence generation (selection bias)	Unclear risk	Quote: "The main features of the overall study design include random allocation of groups to receive SMT either immediately or after a waiting period." (p. 329)
Allocation concealment (selection bias)	Unclear risk	Not reported
Blinding of participants and personnel (performance bias) All outcomes	High risk	Participants not blinded.
Blinding of outcome assessment (detection bias) All outcomes	High risk	Participants were not blinded whereas outcomes are self‐reported.
Incomplete outcome data (attrition bias) All outcomes	High risk	Only the data for those participants (62/92) who had completed the full set of assessment questionnaires were used in the analyses
Selective reporting (reporting bias)	Low risk	All outcomes reported
Other bias	Low risk	We did not find any indications of other sources of bias

**Table:** Riley 2017

***Study characteristics***		
Methods	**Study design:** randomised controlled trial **Study grouping:** parallel group	
Participants	**Baseline Characteristics** Cognitive Behavioral Stress Management (CBSM) *Age (mean ± SD)*: NR *Sex (N (% female))*: NR *Sample size*: 19 *Years of experience (mean ± SD)*: NR Yoga‐Based Stress Management (YBSM) *Age (mean ± SD)*: NR *Sex (N (% female))*: NR *Sample size*: 19 *Years of experience (mean ± SD)*: NR Overall *Age (mean ± SD)*: 44.6 ± 6.2 *Sex (N (% female))*: 32 (84%) *Sample size*: 38 *Years of experience (mean ± SD)*: NR **Included criteria:** to determine eligibility, potential participants completed the Physical Activity Readiness Questionnaire (PAR‐Q; Canadian Society for Exercise Physiology, 2002), a 7‐item questionnaire assessing individuals’ ability to engage in exercise without risk. Only potential participants who answered No to all of the PAR‐Q items were eligible to participate in this study. Employees who attested to meeting all eligibility requirements underwent an informed consent process. **Excluded criteria:** NR **Pretreatment:** The two intervention groups were statistically similar to each other on most demographic baseline variables. However, the sample of mental health care providers was, on average, significantly less stressed than national norms (Table 2; Crawford & Henry, 2003). **Compliance rate:** YBSM participants attended an average of 6.43 sessions (SD = 1.78), and CBSM participants attended an average of 6.13 sessions (SD = 1.86), a difference that was not statistically significantly different. **Response rate:** not able to assess **Type of healthcare worker:** exclusively frontline mental health care providers.	
Interventions	**Intervention characteristics** Cognitive Behavioural Stress Management (CBSM) *Type of the intervention*: Intervention type 1 ‐ to focus one’s attention on the experience of stress *Description of the intervention*: Week 1 Introduction to program, stress awareness/ physical response, 8 muscle group progressive muscle relaxation (PMR). Week 2 Stress awareness and appraisal processes, diaphragmatic breathing. Week 3 Automatic thoughts, deep breathing and counting, passive PMR, special place imagery. Week 4 Rational thought replacement, autogenic training, coping part 1. Week 5 Coping part 2, patterns of coping, light meditation. Week 6 Social support, anger management, Colour meditation. Week 7 Assertiveness training, mantra meditation. Week 8 Review of course, monitor your thinking, imagery exercise *The number of sessions*: 8 *Duration of each session on average*: 1 hour *Duration of the entire intervention*: 8 weeks *Duration of the entire intervention short vs long*: Short *Intervention deliverer*: the two CBSM leaders were licenced clinical psychologists with training in cognitive behavioural therapy. *Intervention form*: Group Yoga‐Based Stress Management (YBSM) *Type of the intervention*: Intervention type 2 ‐ to focus one’s attention away from the experience of stress *Description of the intervention*: Week 1. Introduction to program, Introduction to Complete breath, 40 minutes of yoga flow. Week 2. Self‐observation without judgement, body scan, 45 minutes of yoga. Week 3. Depletion and renewal discussion, self‐reflective journaling exercise, 45 minutes of yoga practice. Week 4. Riding the wave stress management discussion and practice, 45 minutes of yoga: breathe, relax, feel, watch, and allow. Week 5. Tools for renewal discussion, 45 minutes of yoga, alternate nostril breathing, seated meditation, one‐word integration Week 6. Working with resistance discussion (awareness, acceptance, and adjustment of goals), 45 minutes of yoga: Exploring the core. Week 7. Loving kindness meditation, 45 minutes of yoga: heart opening yoga poses, guided visualization, and discussion of social connection. Week 8. Review of course, 45‐minute yoga class, reflective journaling, creating realistic intentions *The number of sessions*: 8 *Duration of each session on average*: 1 hour *Duration of the entire intervention*: 8 weeks *Duration of the entire intervention short vs long*: Short *Intervention deliverer*: The two YBSM teachers were certified Kripalu yoga instructors *Intervention form*: Group	
Outcomes	*DASS‐stress* **Outcome type**: ContinuousOutcome *DASS anxiety* **Outcome type**: ContinuousOutcome *DASS depression* **Outcome type**: ContinuousOutcome *ProQOL ‐ Compassion satisfaction* **Outcome type**: ContinuousOutcome *ProQOL ‐ Burnout* **Outcome type**: ContinuousOutcome *ProQOL ‐ Secondary Trauma* **Outcome type**: ContinuousOutcome *PHQ‐9‐ Patient Health Questionnaire.* **Outcome type**: ContinuousOutcome	
Identification	**Sponsorship source:** NR **Country:** USA **Setting:** Department of Psychiatry at a large regional hospital in New England. **Comments:** NR **Authors name:** Kristen E. Riley **Institution:** Department of Psychology, University of Connecticut **Email:** kristen.e@gmail.com **Address:** 486 Babbidge Rd., Unit 1020, Storrs, CT 06269, USA **Time period:** NR	
Notes	DASS‐stress included in analysis 5.1 and 5.2. PHQ included in analysis 5.4 and 5.5.	
***Risk of bias***		
**Bias**	**Authors' judgement**	**Support for judgement**
Random sequence generation (selection bias)	Unclear risk	Quote: "Participants were directed to an online, secure website (Qualtrics) to complete the online survey assessments at each of the four study time points. Baseline survey assessments were completed within 3 days after completing informed consent. After completing baseline measures, participants were e‐mailed their randomized group assignment (CBSM or YBSM) and information on the meeting time and place of the assigned group." Random sequence generation process insufficiently described.
Allocation concealment (selection bias)	Unclear risk	Difficult to judge whether participants and/or investigators could possibly foresee assignment, however it is assumed that it affects outcomes is small.
Blinding of participants and personnel (performance bias) All outcomes	High risk	Quote: "After completing baseline measures, participants were e‐mailed their randomized group assignment (CBSM or YBSM) and information on the meeting time and place of the assigned group. Two 8‐week" Participants were not blinded.
Blinding of outcome assessment (detection bias) All outcomes	High risk	Participants were not blinded whereas outcomes are self‐reported.
Incomplete outcome data (attrition bias) All outcomes	Low risk	Quote: "Thirty‐eight participants completed baseline questionnaires (YBSM = 19, CBSM = 19), 28 participants completed postintervention Time 2 questionnaires, 25 participants completed Time 3 2‐month follow‐up questionnaires, and 19 completed 6‐month post‐follow‐up questionnaires. Based on t tests and chi‐squared tests, drop‐out participants did not differ significantly on any baseline variable from those who remained in the study."
Selective reporting (reporting bias)	Unclear risk	No trial registration or study protocol reported, nor did we find one online
Other bias	Unclear risk	Response rate not reported.

**Table:** Saganha 2012

***Study characteristics***		
Methods	RCT with individual participants, Portugal	
Participants	Physiotherapists scoring more than 26 on Emotional Exhaustion subscale of MBI out of 106 screened with MBI, and not familiar with Qigong; Experimental n = 8 Control n = 8	
Interventions	1) Experimental: Qigong exercise: posture, breathing and mind focus; classes 20 min/day 1 week; self‐treatment 2X / day 2 weeks, total 3 weeks 2) Control: waiting list for 3 weeks; after that they got the treatment as well	
Outcomes	MBI	
Identification		
Notes	MBI‐EE included in analysis 2.1	
***Risk of bias***		
**Bias**	**Authors' judgement**	**Support for judgement**
Random sequence generation (selection bias)	Unclear risk	Quote: "the design was a prospective randomized controlled study"
Allocation concealment (selection bias)	Unclear risk	Quote: "the design was a prospective randomized controlled study"
Blinding of participants and personnel (performance bias) All outcomes	High risk	Participants not blinded.
Blinding of outcome assessment (detection bias) All outcomes	High risk	Participants were not blinded whereas outcomes are self‐reported.
Incomplete outcome data (attrition bias) All outcomes	Low risk	Outcomes for all participants reported
Selective reporting (reporting bias)	Unclear risk	Baseline data not reported for the RCT group only
Other bias	Low risk	We did not find any indications of other sources of bias

**Table:** Sampson 2019

***Study characteristics***		
Methods	**Study design:‐**cluster randomised controlled trial **Study grouping:** parallel group	
Participants	**Baseline characteristics** MINDBODYSTRONG *Age (mean ± SD)*: NR *Sex (N (% female))*: 41 (87%) *Sample size*: 49 *Years of experience (mean ± SD)*: < 1 Control (no intervention) *Age (mean ± SD)*: NR *Sex (N (% female))*: 35 (83%) *Sample size*: 44 *Years of experience (mean ± SD)*: < 1 Overall *Age (mean ± SD)*: 24.5 ± NR *Sex (N (% female))*: 76 (84%) *Sample size*: 90 *Years of experience (mean ± SD)*: < 1 **Included criteria:** inclusion criteria included all NLRNs hired during the study period who signed consent for the study. **Excluded criteria:** exclusion criteria included any NLRN who did not consent to participate in the study. **Pretreatment:** A MANOVA was used to determine group differences at baseline. No significant differences were noted in mean variable scores between the control group and the intervention group for any of the study variables **Compliance rate:** 96% **Response rate:** 85% (89 out of 105) **Type of healthcare worker:** exclusively newly licenced nurses	
Interventions	**Intervention characteristics** MINDBODYSTRONG *Type of the intervention*: Intervention type 1 ‐ to focus one’s attention on the experience of stress *Description of the intervention*: The MINDBODYSTRONG Program is a novel adaptation of an evidence‐based cognitive behavioural skills–building intervention that provides a theory‐based approach to improve the mental health, healthy lifestyle beliefs and behaviours, and job satisfaction ofNLRNs. The curriculum is manualized into a workbook to ensure consistency of information, allow participants to interact during all skills building/practice activities, and provide a guide for accomplishing goals. The MINDBODYSTRONG Program was adapted from the evidence‐based Creating Opportunities for Personal Empowerment Program by Melnyk, which has been shown to decrease depressive, anxiety, and stress symptoms and increase healthy lifestyle behaviours in children, adolescents, and college‐age youth in numerous studies. 23‐27 Each of the 8 MINDBODYSTRONG sessions (Table 1) focus' on three areas: caring for the mind, caring for the body, and skills building. Participants learn CBT concepts, establish weekly goals, and complete skills‐building activities weekly. Each session begins with a review of previously learnt concepts and a review of skills‐building activities. *The number of sessions*: 8 *Duration of each session on average*: 45‐minute *Duration of the entire intervention*: 8 weeks *Duration of the entire intervention short vs long*: short *Intervention deliverer*: Author *Intervention form*: Group Control (no intervention) *Type of the intervention*: Usual care *Description of the intervention*: The attention control group received the usual nurse residency curriculum that included a 30‐ to 45‐minute debriefing session each week where they discussed successes and challenges experienced the prior week while also receiving peer support from other cohort members. Members of the control group were given information on the Employee Assistance Program and other resources available to employees of the organization. *The number of sessions*: 8 *Duration of each session on average*: 30‐ to 45‐minute *Duration of the entire intervention*: 8 weeks *Duration of the entire intervention short vs long*: short *Intervention deliverer*: Author *Intervention form*: Group	
Outcomes	*Perceived Stress Scale (PSS)* **Outcome type**: ContinuousOutcome *Generalized Anxiety Disorder Scale (GAD‐7)* **Outcome type**: ContinuousOutcome *Personal Health Questionnaire* **Outcome type**: ContinuousOutcome	
Identification	**Sponsorship source:** NR **Country:** USA **Setting:** a large, Midwestern academic medical center with an accredited nurse residency program. **Comments:** NR **Authors name:** Dr Sampson **Institution:** The Ohio State University Wexner Medical Center **Email:** marlene.sampson@osumc.edu **Address:** 600 Ackerman Rd, Suite 2017E, Columbus, OH 43202 **Time period:** 2018‐2019	
Notes	We kindly received the mean and SD of the PSS for both groups from author M. Sampson PSS included in analysis 1.1 and 1.2.	
***Risk of bias***		
**Bias**	**Authors' judgement**	**Support for judgement**
Random sequence generation (selection bias)	Unclear risk	Quote: "Participants were NLRNs hired between July 1, 2018, and September 30, 2018, and placed in 1 of 4 cohorts based on hire date. Two cohorts were randomly assigned to the control group, and 2 cohorts were randomly assigned to the MINDBODYSTRONG intervention group." Sequence generation process insufficiently described
Allocation concealment (selection bias)	Unclear risk	Difficult to judge whether participants and/or investigators could possibly foresee assignment. However, it is assumed that randomization was performed in one go and that participants and/or investigators could not foresee assignment.
Blinding of participants and personnel (performance bias) All outcomes	High risk	Participants were not blinded.
Blinding of outcome assessment (detection bias) All outcomes	High risk	Participants were not blinded whereas outcomes are self‐reported.
Incomplete outcome data (attrition bias) All outcomes	Low risk	89 of the 93 (96%) randomised participants included in the analyses. Not reported whether loss to follow‐up was at random.
Selective reporting (reporting bias)	Unclear risk	No trial registration or no study protocol reported, nor did we find one online
Other bias	Low risk	No indication of other sources of bias

**Table:** Sawyer 2021

***Study characteristics***		
Methods	**Study design:** randomised controlled trial **Study grouping:** parallel group	
Participants	**Baseline characteristics** Resilience, insight, self‐compassion, and empowerment (RISE) *Age in years (20‐29; 30‐39; 30‐39; 50‐59; 60‐69)*: 10 (30%); 8 (24%); 4 (12%); 10 (30%); 1 (3%) *Sex (N (% female))*: 29 (88%) *Sample size*: 33 *Years of experience (mean ± SD)*: NR Control (no intervention) *Age in years (20‐29; 30‐39; 30‐39; 50‐59; 60‐69)*: 18 (43%); 14 (33%); 3 (7%); 6 (14%); 1 (2%) *Sex (N (% female))*: 37 (88%) *Sample size*: 42 *Years of experience (mean ± SD)*: NR Overall *Age in years (20‐29; 30‐39; 30‐39; 50‐59; 60‐69)*: NR *Sex (N (% female))*: 66 (88%) *Sample size*: 75 *Years of experience (mean ± SD)*: NR **Included criteria:** inclusion criteria for participants were age 18 years or older; licenced as a registered nurse; employed by the health care system in a hospital‐based setting; able to speak, read, and understand English fluently; able to provide informed consent; and willing and able to comply with all study procedures and requirements for the duration of the study. **Excluded criteria:** exclusion criteria were employed by the health care system as an advanced registered nurse practitioner, employed in a role that completes another registered nurse’s annual evaluations, or at imminent risk of harm to themselves or others. **Pretreatment:** NR **Compliance rate:** many nurses in both the intervention group and the control group withdrew from the study before starting the intervention because they had scheduling conflicts, employment changes, or personal obligations that affected their ability to attend. **Response rate:** NR **Type of healthcare worker:** exclusively nurses	
Interventions	**Intervention characteristics** Resilience, insight, self‐compassion, and empowerment (RISE) *Type of the intervention*: Intervention type 1 ‐ to focus one’s attention on the experience of stress *Description of the intervention*: 1 Introduction. Group process and guidelines RISE framework Drivers and symptoms of burnout Mindfulness. 2 Resilience. Definition of resilience Stress recovery and oscillation Coping skills Connection to joy and purpose. 3 Insight. Definition of insight Cognitive awareness Emotional literacy. 4 Self‐compassion. Compassion in nursing Compassion fatigue and secondary trauma Definition of self‐compassion Introduction to self‐compassion skills. 5 Self‐compassion. Self‐validation Combatting negative self‐talk and self‐criticism. 6 Empowerment. Definition of personal empowerment Environmental impact on empowerment Learned helplessness Values‐behaviour alignment. 7 Empowerment. Boundaries Assertive communication Self‐advocacy. 8 Closing. Closing and goodbye Synthesis of learning Call to action self‐care guide. *The number of sessions*: 8 *Duration of each session on average*: 90‐minutes *Duration of the entire intervention*: 7 months *Duration of the entire intervention short vs long*: Long *Intervention deliverer*: by a licensed mental health Counsellor, educator, and full‐time employee of the organisation's' research institute *Intervention form*: Group Control (no intervention) *Type of the intervention*: NA *Description of the intervention*: NA *The number of sessions*: NA *Duration of each session on average*: NA *Duration of the entire intervention*: NA *Duration of the entire intervention short vs long*: NA *Intervention deliverer*: NA *Intervention form*: NA	
Outcomes	*Perceived Stress Scale (PSS)* **Outcome type**: ContinuousOutcome *Maslach Burnout Inventory ‐ Emotional Exhaustion* **Outcome type**: ContinuousOutcome *Maslach Burnout Inventory ‐ Depersonalisation* **Outcome type**: ContinuousOutcome *Maslach Burnout Inventory ‐ Personal accomplishment (lack of)* **Outcome type**: ContinuousOutcome	
Identification	**Sponsorship source:** The authors received no financial support for the research, authorship, and/or publication of this article. **Country:** USA **Setting:** Hospital‐based setting **Comments:** NR **Authors name:** Amanda T. Sawyer, **Institution:** Research Institute, Advent Health **Email:** amanda.sawyer@adventhealth.com **Address:** Orlando, 301 East Princeton Street, Orlando, FL 32804, USA. **Time period:** NR	
Notes	We kindly received the mean and SD for the primary outcome from author A. Swayer PSS included in analysis 1.2	
***Risk of bias***		
**Bias**	**Authors' judgement**	**Support for judgement**
Random sequence generation (selection bias)	Low risk	Quote: "This study was a parallel randomized controlled trial with an allocation ratio of 1:1. Participants were randomized into the intervention or wait‐list control group by a computer‐generated randomized number concealed from the recruitment team member in an opaque envelope until it was time to assign a participant to a group."
Allocation concealment (selection bias)	Low risk	Quote: "This study was a parallel randomized controlled trial with an allocation ratio of 1:1. Participants were randomized into the intervention or wait‐list control group by a computer‐ generated randomized number concealed from the recruitment team member in an opaque envelope until it was time to assign a participant to a group. "
Blinding of participants and personnel (performance bias) All outcomes	High risk	Participants were not blinded.
Blinding of outcome assessment (detection bias) All outcomes	High risk	Participants were not blinded whereas outcomes are self‐reported.
Incomplete outcome data (attrition bias) All outcomes	High risk	Quote: "Many nurses in both the intervention group and the control group withdrew from the study before starting the intervention because they had scheduling conflicts, employment changes, or personal obligations that affected their ability to attend. At the 1‐month, 3‐month, and 6‐month follow‐up timepoints, there were 75, 63, and 49 participants in the study, respectively." 26 of the 75 randomised participants were lost to follow‐up (35%). Not reported whether loss to follow‐up was at random.
Selective reporting (reporting bias)	Low risk	Quote: "The study was approved by the institutional review board and registered on ClinicalTrials.gov." The authors did not provide a registration number. Using Google we found registration information for a pilot study ‐> https://clinicaltrials.gov/ct2/show/NCT03645707. No indication of selective reporting
Other bias	Unclear risk	Quote: "Many nurses in both the intervention group and the control group withdrew from the study before starting the intervention because they had scheduling conflicts, employment changes, or personal obligations that affected their ability to attend. At the 1‐month, 3‐month, and 6‐month follow‐up timepoints, there were 75, 63, and 49 participants in the study, respectively." Compliance rate could not be assessed. Baseline differences not reported.

**Table:** Schrijnemaekers 2003

***Study characteristics***		
Methods	Cluster‐randomised trial, the Netherlands	
Participants	300 professional caregivers in homes for elderly persons	
Interventions	1) Experimental: emotion‐oriented care training, clinical lessons and supervision meetings: 1‐hour clinical lesson, 6‐day training programme with 4 days at a 2‐week interval and last 2 days at a 4‐week interval. The participants were taught about the dementia syndrome and various care models for communicating with elderly people with dementia (e.g. reality orientation, validation and reminiscence), inequality of the resident‐caregiver relation, understanding the residents' perception of the environment and the attitude and (non‐)verbal communication of staff towards the resident. Intervention homes also received 3 half‐day supervision meetings to support the implementation of emotion‐oriented care. 2) Control: no intervention	
Outcomes	MBI, Job satisfaction	
Identification	Not able to include in analysis due to missing data.	
Notes		
***Risk of bias***		
**Bias**	**Authors' judgement**	**Support for judgement**
Random sequence generation (selection bias)	Unclear risk	Quote: "Randomization was performed on the level of homes. ...within each pair, one home was randomly assigned to the intervention or control group, and the home was assigned to the alternate state." (p. S51)
Allocation concealment (selection bias)	Unclear risk	Not reported
Blinding of participants and personnel (performance bias) All outcomes	High risk	Participants not blinded.
Blinding of outcome assessment (detection bias) All outcomes	High risk	Participants were not blinded whereas outcomes are self‐reported.
Incomplete outcome data (attrition bias) All outcomes	Low risk	Quote: "Missing values on items that were part of a scale or subscale were replaced according to the "mean value of valid subtests" principle (i.e. replacement by the mean value calculated from the valid item scores of the [sub‐] scale obtained for the same subject at the same time point). This replacement strategy was only used if less than 25% of the items of a scale or subscale had missing values." (p. S52)
Selective reporting (reporting bias)	Low risk	All outcomes reported
Other bias	Low risk	We did not find any indications of other sources of bias

**Table:** Schroeder 2018

***Study characteristics***		
Methods	**Study design:** randomised controlled trial **Study grouping:** parallel group	
Participants	**Baseline characteristics** Mindful Medicine Curriculum (MMC) *Age (mean ± SD)*: NR *Sex (N (% female))*: NR *Sample size*: 17 *Years of experience (mean ± SD)*: NR Control (waitlist) *Age (mean ± SD)*: NR *Sex (N (% female))*: NR *Sample size*: 16 *Years of experience (mean ± SD)*: NR Overall *Age (mean ± SD)*: 42.8 ± 8.4 *Sex (N (% female))*: 24 (73%) *Sample size*: 33 *Years of experience (mean ± SD)*: 13.3 ± 8.1 **Included criteria:** inclusion criteria were (a) employed as a primary care physician by Providence Medical Group (PMG), (b) working at least 30% time in direct patient care, (c) aged between 25 and 75 years, (d) willing to be randomised to the intervention or wait list control group, and (e) no prior participation in the same MBI offered at PMG. **Excluded criteria:** NR **Pretreatment:** there were no significant differences between the intervention (n = 17) and wait list control (n = 16) group on any demographic variables (all P > 0.05). **Compliance rate:** 1/15 (6.7%) did not receive the intervention **Response rate:** NR **Type of healthcare worker:** exclusively primary care physicians	
Interventions	**Intervention characteristics** Mindful Medicine Curriculum (MMC) *Type of the intervention*: Intervention type 1 ‐ to focus one’s attention on the experience of stress *Description of the intervention*: Key to the MMC is an introduction to mindfulness that is relevant to the professional contexts in which physicians work, hence emphasising the physicians’ ability to incorporate mindfulness and compassion into interpersonal relationships. Instructors present the MMC using secular, accessible language, and they have extensive experience in secular MBIs and familiarity with the culture of physicians. *The number of sessions*: 4 *Duration of each session on average*: MMC is a 13‐hourweekend training program plus 2‐hour follow‐up sessions scheduled at 2 and 4 weeks after the weekend. *Duration of the entire intervention*: 4 weeks *Duration of the entire intervention short vs long*: Short *Intervention deliverer*: NR *Intervention form*: Group Control (waitlist) *Type of the intervention*: NA *Description of the intervention*: NA *The number of sessions*: NA *Duration of each session on average*: NA *Duration of the entire intervention*: NA *Duration of the entire intervention short vs long*: NA *Intervention deliverer*: NA *Intervention form*: NA	
Outcomes	*Perceived Stress Scale* **Outcome type**: ContinuousOutcome *Maslach Burnout Inventory ‐ Emotional Exhaustion* **Outcome type**: ContinuousOutcome *Maslach Burnout Inventory ‐ Depersonalisation* **Outcome type**: ContinuousOutcome *Maslach Burnout Inventory ‐ Personal accomplishment (lack of)* **Outcome type**: ContinuousOutcome	
Identification	**Sponsorship source:** The author(s) received no financial support for the research, authorship, and/or publication of this article. **Country:** USA **Setting:** family medicine and internal medicine departments **Comments:** NA **Authors name:** David A. Schroeder **Institution:** Providence Heart Clinic, Portland, OR (DAS); Endocrinology–Medical Education, Providence Medical Center, Portland, OR (ES); Department of Internal Medicine, Oregon Health and Sciences University, **Email:** mchristopher@pacificu.edu. **Address:** Michael S. Christopher, PhD, School of Professional Psychology, Pacific University, 190 SE 8th Avenue, Suite 260, Hillsboro, OR 97123; e‐mail: mchristopher@pacificu.edu **Time period:** December 2014 and May 2015	
Notes	PSS included in analysis 1.1.	
***Risk of bias***		
**Bias**	**Authors' judgement**	**Support for judgement**
Random sequence generation (selection bias)	Unclear risk	Quote: "After completing the baseline measures, participants were randomized 1:1 into the intervention or a waitlist control." Sequence generation process insufficiently described.
Allocation concealment (selection bias)	Unclear risk	Quote: "After completing the baseline measures, participants were randomized 1:1 into the intervention or a waitlist control." Difficult to judge whether participants and/or investigators could possibly foresee assignment
Blinding of participants and personnel (performance bias) All outcomes	High risk	Participants were not blinded.
Blinding of outcome assessment (detection bias) All outcomes	High risk	Participants were not blinded whereas outcomes are self‐reported.
Incomplete outcome data (attrition bias) All outcomes	Low risk	4 of the 33 (12%) randomised participants included in the analyses. Not reported whether loss to follow‐up was at random. However loss to follow‐up is below our pre‐defined cut‐off point.
Selective reporting (reporting bias)	Low risk	Quote: "Trial Registration Not applicable, because this article does not contain any clinical trials." No trial registration or no study protocol reported. No indication of selective reporting.
Other bias	Unclear risk	Quote: "Potential participants were recruited via email for 3 weeks prior to the first MBI group (January 2015). Participants responded to the recruitment email by directing their browser to the study website, which was housed on Qualtrics, a secure web‐based survey system." Not able to assess response rate.

**Table:** Seidel 2021

***Study characteristics***		
Methods	**Study design:** randomised controlled trial **Study grouping:** parallel group	
Participants	**Baseline characteristics** Mindfulness Curriculum *Age (mean ± SD)*: NR *Sex (N (% female))*: NR *Sample size*: NR *Years of experience (mean ± SD)*: NR Control (wait list) *Age (mean ± SD)*: NR *Sex (N (% female))*: NR *Sample size*: NR *Years of experience (mean ± SD)*: NR Overall *Age (mean ± SD)*: NR *Sex (N (% female))*: 71 (86%) *Sample size*: 83 *Years of experience (mean ± SD)*: NR **Included criteria:** NR **Excluded criteria:** NR **Pretreatment:** NR **Compliance rate:** NR **Response rate:** NR **Type of healthcare worker:** quote: "Physicians, nurses, physician assistants, nurse practitioners, clinical nurse specialists, social workers, physical therapists, occupational therapists, pharmacists, psychologists, and chaplains"	
Interventions	**Intervention characteristics** Mindfulness Curriculum *Type of the intervention*: Intervention type 2 ‐ to focus one’s attention away from the experience of stress *Description of the intervention*: "A series of 4 consecutive weekly 1‐hour classes on mindfulness (Building a Mindful CommUnity) and 15 minutes of daily practice." *The number of sessions*: 4 *Duration of each session on average*: 1 hour *Duration of the entire intervention*: 4 weeks *Duration of the entire intervention short vs long*: Short *Intervention deliverer*: NR *Intervention form*: Face‐to‐face classes AND at home practice by individual Control (wait list) *Type of the intervention*: NA *Description of the intervention*: NA *The number of sessions*: NA *Duration of each session on average*: NA *Duration of the entire intervention*: NA *Duration of the entire intervention short vs long*: NA *Intervention deliverer*: NA *Intervention form*: NA	
Outcomes	*Maslach Burnout Inventory ‐ Emotional Exhaustion* **Outcome type**: ContinuousOutcome *Maslach Burnout Inventory ‐ Depersonalisation* **Outcome type**: ContinuousOutcome *Maslach Burnout Inventory ‐ Personal accomplishment (lack of)* **Outcome type**: ContinuousOutcome	
Identification	**Sponsorship source:** "Portions of the project were funded by a Research AccelerationProgram grant from Carilion Clinic." **Country:** USA **Setting:** Academic medical centre **Comments:** The publication discusses two studies: 1 a quasi‐experimental study and another an RCT. The RCT details are abstracted here. **Authors name:** Laurie Walker Seidel **Institution:** Virginia Tech **Email:** wseidel@vt.edu **Address:** Roanoke City Public Schools Central Office, 40 Douglass Ave, Roanoke, VA 24012, USA **Time period:** 2016‐2017	
Notes	MBI‐EE included in analysis 2.1 and 2.2	
***Risk of bias***		
**Bias**	**Authors' judgement**	**Support for judgement**
Random sequence generation (selection bias)	Unclear risk	Participants were randomly assigned to either the intervention group or a waitlist control group. Sequence generation process insufficiently described.
Allocation concealment (selection bias)	Unclear risk	Unable to judge whether participants and/or investigators could possible foresee assignment
Blinding of participants and personnel (performance bias) All outcomes	High risk	Blinding not possible
Blinding of outcome assessment (detection bias) All outcomes	High risk	Participants were not blinded whereas outcomes are self‐reported.
Incomplete outcome data (attrition bias) All outcomes	Low risk	Of the 103 healthcare professionals who enroled, 83 completed the study (81%). Reasons not provide, nor whether missing was at random. Lost to follow‐up just below our pre‐defined cut‐off value.
Selective reporting (reporting bias)	Low risk	No trial registration or no study protocol reported. No indication of selective reporting.
Other bias	Unclear risk	No information on baseline differences. Contamination between groups ‐> There was a low attrition rate, supporting the effectiveness of offering each class multiple times within the week to expand choice and flexibility with the typical scheduling of a healthcare environment. The pattern of results may indicate that participants' knowing they were eventually to receive mindfulness training motivated members of the control group to improve their outlook. Although one cannot be certain about any post hoc explanation, it seems more likely that intervention participants shared what they learnt with those in the control group during the training period. It was observed during study 2 enrolment, for example, that several groupings of friends were assigned randomly to different conditions. Sharing information may explain why control participants increased mindful awareness during the intervention but did not continue that increase to the 6‐month posttest

**Table:** Shapiro 2005

***Study characteristics***		
Methods	RCT, USA	
Participants	38 healthcare professionals including physicians, nurses, social workers, physical therapists and psychologists	
Interventions	1) Experimental: Mindfulness‐Based Stress Reduction programme: 8 weekly 2‐hour training sessions about employing the techniques involved in sitting meditation, body scan, Hatha yoga, 3‐minute breathing space (a "minimeditation") and a "loving kindness" meditation. 2) Control: no intervention	
Outcomes	MBI, Perceived Stress Scale, Brief Symptom Inventory	
Identification		
Notes	PSS included in analysis 2.1	
***Risk of bias***		
**Bias**	**Authors' judgement**	**Support for judgement**
Random sequence generation (selection bias)	Unclear risk	Quote: "Participants were randomly assigned to an 8‐week MBSR group or a wait‐list control group." (p. 167)
Allocation concealment (selection bias)	Unclear risk	Not reported
Blinding of participants and personnel (performance bias) All outcomes	High risk	Participants not blinded.
Blinding of outcome assessment (detection bias) All outcomes	High risk	Participants were not blinded whereas outcomes are self‐reported.
Incomplete outcome data (attrition bias) All outcomes	High risk	Quote: "Owing to the pilot nature of this study and the small sample size, we did not perform intention‐to‐treat analyses but compared only those participants who did not drop out" (p. 169)
Selective reporting (reporting bias)	Low risk	All outcomes reported
Other bias	Low risk	We did not find any indications of other sources of bias

**Table:** Sharif 2013

***Study characteristics***		
Methods	**Study design:** cluster‐randomised controlled trial **Study grouping:** parallel group	
Participants	**Baseline Characteristics** Emotional intelligence training *Age in years (mean ± SD)*: 36.3 ± 6.7 *Sex (N (% female))*: NR *Sample size*: 28 *Years of experience (mean ± SD)*: 11.6 ± 6.03 Control (no intervention) *Age in years (mean ± SD)*: 33.0 ± 6.3 *Sex (N (% female))*: NR *Sample size*: 28 *Years of experience (mean ± SD)*: 9.3 ± 6.2 Overall *Age in years (mean ± SD)*: NR *Sex (N (% female))*: NR *Sample size*: 56 *Years of experience (mean ± SD)*: NR **Included criteria:** NR **Excluded criteria:** NR **Pretreatment:** the two study arms were not significantly different in terms of age, working experience, and working hours (Table 1); the distribution of employment status, shift working, and number of night shifts per week, were also not different between the two groups (Table 2). No significant difference was observed between the two groups in terms of emotional intelligence and mental health mean scores before the intervention (Tables 3 and 4). **Compliance rate:** 25 of the 28 randomised participants did not receive the intervention for being busy. **Response rate:** 84 of the 140 eligible participants did not participate (60%) **Type of healthcare worker:** various healthcare professionals including nurses 44 (75%), head nurses 10 (17%) and supervisors 5 (9%)	
Interventions	**Intervention characteristics** Cognitive‐behavioral therapy + relaxation *Type of the intervention*: Intervention type 1 ‐ to focus one’s attention on the experience of stress *Description of the intervention*: The nurses of the intervention group were invited to take part in a training courses held in the College of Nursing and Midwifery, Shiraz University of Medical Sciences, Shiraz, Iran. During this two day workshop, components of emotional intelligence were taught. Of the intervention group nurses, 25 completed the training course. The course included the concept of health, self‐awareness skills, stress and its symptoms, stress management, the relationship between thoughts and emotions, emotional intelligence, management of emotions, relationship management, and self‐management. *The number of sessions*: 2 *Duration of each session on average*: NR *Duration of the entire intervention*: 2 days *Duration of the entire intervention short vs long*: Short *Intervention deliverer*: NR *Intervention form*: Group, face‐to‐face Control (no intervention) *Type of the intervention*: NA *Description of the intervention*: To consider ethical issues, the nurses in the control group were provided with educational materials after completion of the study *The number of sessions*: NA *Duration of each session on average*: NA *Duration of the entire intervention*: NA *Duration of the entire intervention short vs long*: NA *Intervention deliverer*: NA *Intervention form*: NA	
Outcomes	*28‐item Goldberg's general health questionnaires (GHQ‐20)* **Outcome type**: ContinuousOutcome	
Identification	**Sponsorship source:** The authors would like to thank the Vice‐Chancellor for Research, Shiraz University of Medical Sciences, for financially supporting this study. **Country:** Iran **Setting:** Hospital **Comments:** NR **Authors name:** Farkhondeh Sharif, PhD **Institution:** Faculty of Nursing, Department of Mental Health Nursing, Shiraz University of Medical Science. **Email:** fsharif@sums.ac.ir **Address:** NR **Time period:** NR	
Notes	GHQ included in analysis 1.1	
***Risk of bias***		
**Bias**	**Authors' judgement**	**Support for judgement**
Random sequence generation (selection bias)	Unclear risk	Of 200 ICU nurses, 28 were working in Shahid Faghihi hospital and selected as the control group; 28 nurses who were working in Namazi hospital were allocated to the intervention group. Selection of the hospitals for the control and the intervention group was random.
Allocation concealment (selection bias)	Unclear risk	Not reported.
Blinding of participants and personnel (performance bias) All outcomes	High risk	Participants not blinded.
Blinding of outcome assessment (detection bias) All outcomes	High risk	Participants not blinded whereas outcomes are self‐reported.
Incomplete outcome data (attrition bias) All outcomes	Low risk	52 of the 56 randomised participants included in the analysis. Not reported whether missing was at random.
Selective reporting (reporting bias)	Unclear risk	Inaccessible trial registration. No indication of selective reporting.
Other bias	Unclear risk	Low participation rate (40%). Did not incorporate unit of analysis error.

**Table:** Shin 2020

***Study characteristics***		
Methods	**Study design:** randomised controlled trial **Study grouping:** parallel group	
Participants	**Baseline characteristics** Patchouli *Age (mean ± SD)*: 26.4 ± 3.1 *Sex (N (% female))*: 25 (100%) *Sample size*: 25 *Years of experience (mean ± SD)*: 3.7 ± 2.2 Control (placebo) *Age (mean ± SD)*: 26.6 ± 3.4 *Sex (N (% female))*: 25 (100%) *Sample size*: 25 *Years of experience (mean ± SD)*: 3.6 ± 2.4 Overall *Age (mean ± SD)*: 26.5 ± 3.2 *Sex (N (% female))*: 25 (100%) *Sample size*: 50 *Years of experience (mean ± SD)*: 3.7 ± 2.3 **Included criteria:** participants were included if they (1) understood the purpose of this study and agreed to participate voluntarily, (2) had been a nurse in the emergency room for at least 6 months, (3) did not have any disease and were not being treated for illness, (4) were not pregnant, (5) did not have an abnormality in olfactory function, (6) did not have asthma or an allergic reaction to patchouli oil, (7) were not receiving stress management such as an exercise or massage program, (8) had never received aromatherapy, and (9) were not regularly taking any medication that was likely to affect mental health status. **Excluded criteria:** NR **Pretreatment:** There were no significant differences between the groups in age, gender, body mass index, marital status, education, religion, duration of nursing career, duration of serving as emergency nurses, and experience of traumatic events. **Compliance rate:** 83% **Response rate:** 100% **Type of healthcare worker:** exclusively emergency nurses	
Interventions	**Intervention characteristics** Patchouli *Type of the intervention*: Intervention type 2 ‐ to focus one’s attention away from the experience of stress *Description of the intervention* : Patchouli oil was purchased from Aromarant Co. (Rottingen, Germany), and its composition was analyzed by gaschromatography/mass spectrometry and capillary column(HP‐INNOWAX; Agilent Technologies, USA) before administration. The carrier gas was helium, maintained at a rate of 1.0 mL/min. The initial column temperature was 40C,increasing 3C/min to a maximum of 230C. Phytochemical compounds were identified by their relative retention time, and their identities were confirmed by comparison with reference data. The three most abundant compounds were patchouli alcohol, d‐guaiene, and a‐guaiene (Table 1). In the patchouli group, a 0.5 mL aliquot of 5% patchouli oil dissolved in sweet almond oil (used as solvent, control group) was dropped onto a piece of gauze, measuring 5 mm· 10 mm. The gauze was positioned in the philtrum. Each participant took three deep breaths and was subsequently allowed to inhale the essential oil for 20 min through natural breathing. The length of the study intervention was established based on the results of a previous study. *The number of sessions*: 2 *Duration of each session on average*: NR *Duration of the entire intervention*: 2 days *Duration of the entire intervention short vs long*: Short *Intervention deliverer*: investigator *Intervention form*: Group Control (placebo) *Type of the intervention*: Placebo *Description of the intervention* : In the control group, a 0.5 mL aliquot of pure sweet almond oil was dropped onto a piece of gauze, and subjects inhaled this oil in the same manner. *The number of sessions*: 2 *Duration of each session on average*: NR *Duration of the entire intervention*: 2 days *Duration of the entire intervention short vs long*: Short *Intervention deliverer*: investigator *Intervention form*: Group	
Outcomes	*ProQOL ‐ Compassion satisfaction* **Outcome type**: ContinuousOutcome *Professional quality of life scale‐IV ‐ Compassion fatigue* **Outcome type**: ContinuousOutcome *ProQOL ‐ Burnout* **Outcome type**: ContinuousOutcome *Stress (VAS)* **Outcome type**: ContinuousOutcome	
Identification	**Sponsorship source:** This work was supported by a grant from the Basic Science Research Program through the National ResearchFoundation of Korea (NRF‐2018R1D1A1B07050048) and the Institute of Nursing Research, Korea University Grant. **Country:** Korea **Setting:** University hospital **Comments:** NR **Authors name:** You Kyoung Shin **Institution:** Department of Basic Nursing Science, College of Nursing, Korea University, Seoul, Republic of Korea **Email:** NR **Address:** NR **Time period:** May to August 2018	
Notes	VAS Stress Included in analysis 2.1	
***Risk of bias***		
**Bias**	**Authors' judgement**	**Support for judgement**
Random sequence generation (selection bias)	Low risk	Quote: "Therefore, 60 subjects were recruited and randomly assigned to the two groups by an investigator using a random number table."
Allocation concealment (selection bias)	Low risk	Quote: "To conceal the allocation sequence, generation of the random allocation sequence and recruitment of participants were conducted by independent investigators."
Blinding of participants and personnel (performance bias) All outcomes	Low risk	Quote: "Participants were blinded to the type of essential oil they inhaled to avoid any placebo effect and were not informed of the study group to which they were allocated."
Blinding of outcome assessment (detection bias) All outcomes	Low risk	Participants were blinded and outcomes are self‐reported.
Incomplete outcome data (attrition bias) All outcomes	High risk	Quote: "Of the 60 subjects recruited to this study, 10 discontinued interventions and were therefore excluded. Thus, 50 subjects were included, 25 in 5% patchouli oil (patchouli) group and 25 in sweet almond oil (control) group (Fig. 1)." No intention‐to‐treat analysis.
Selective reporting (reporting bias)	Low risk	Trial registration KCT0004615. No indication of selective reporting
Other bias	Low risk	No indication of other source of bias

**Table:** Sood 2011

***Study characteristics***		
Methods	RCT, USA	
Participants	40 physicians working at Mayo Clinic Rochester Quote: "Inclusion criteria were: (1) being a faculty member of the DOM and (2) being able and willing to participate. Exclusion criteria were: (1) recent (within the past 6 months) psychotic episode or (2) clinically significant acute unstable neurological, psychiatric, hepatic, renal, cardiovascular, or respiratory disease that prevented participation in the study." (p. 859) Quote: "Mean age of the participants in the active arm (46.8 ± 8.3 years) was comparable to the control arm (50.2 ± 5.7 years). Gender distribution was comparable across the two arms (55% vs 50% males in the active and control arm, respectively)." (p. 859)	
Interventions	1) Experimental: Stress Management and Resiliency Training (SMART) programme (n = 20). Quote: "The study intervention was a single 90‐min session training in the SMART program. The SMART program has been adapted from Attention and Interpretation Therapy (AIT). AIT is a structured therapy developed at the Mayo Clinic to decrease stress and enhance resilience. AIT addresses two aspects of human experience, attention and interpretation." "AIT guides learners to delay judgement and pay greater attention to the novelty of the world. Complementing attention training is instruction to help participants direct their interpretations away from fixed prejudices towards a more flexible disposition while cultivating skills such as gratitude, compassion, acceptance, forgiveness, and higher meaning." " Participants were also offered an optional 30–60‐min follow‐up session depending on individual needs. (p. 859) 2) Control: No intervention control (n = 20)	
Outcomes	Perceived Stress Scale, Smith Anxiety Scale	
Identification	PSS included in analysis 4.1 and the Smith Anxiety Scale in 4.4	
Notes		
***Risk of bias***		
**Bias**	**Authors' judgement**	**Support for judgement**
Random sequence generation (selection bias)	Unclear risk	Quote: "After obtaining the informed consent, physicians were randomly assigned to one of two groups ‐ an active arm or a wait‐list control arm." (p. 859)
Allocation concealment (selection bias)	Unclear risk	The authors do not report if they concealed allocation
Blinding of participants and personnel (performance bias) All outcomes	High risk	Participants not blinded.
Blinding of outcome assessment (detection bias) All outcomes	High risk	Participants were not blinded whereas outcomes are self‐reported.
Incomplete outcome data (attrition bias) All outcomes	Unclear risk	Apparently there were no dropouts
Selective reporting (reporting bias)	Unclear risk	The authors report all results for outcome measures listed in the 'Methods' section.
Other bias	Unclear risk	Quote:"Eight participants (all in the control arm) declined to participate after randomization and prior to filling out any assessments because of scheduling issues" (p. 860)

**Table:** Stanton 1988

***Study characteristics***		
Methods	RCT, Australia	
Participants	40 trained hospital nurses who complained being overstressed	
Interventions	1) Experimental: Ego‐enhancement training: One 50‐minute session and three 20‐minute sessions 1 week as part of training in the techniques of: physical relaxation, mental calmness, disposing of "rubbish", removal of a barrier and enjoyment of a special place. 2) Control: no intervention	
Outcomes	Stress Profile	
Identification		
Notes	Stress profile included in analysis 2.2.	
***Risk of bias***		
**Bias**	**Authors' judgement**	**Support for judgement**
Random sequence generation (selection bias)	Unclear risk	Quote:"In the first stage of the experiment the nurses were matched on their Profile scores, one member of each pair being allocated at random to either a non‐treatment control group or an experimental group experiencing four treatment sessions" (p. 318)
Allocation concealment (selection bias)	Unclear risk	Not reported
Blinding of participants and personnel (performance bias) All outcomes	High risk	Participants not blinded.
Blinding of outcome assessment (detection bias) All outcomes	High risk	Participants were not blinded whereas outcomes are self‐reported.
Incomplete outcome data (attrition bias) All outcomes	Unclear risk	It is unclear if any participants dropped out
Selective reporting (reporting bias)	Low risk	Only 1 outcome measure used and reported
Other bias	Low risk	We did not find any indications of other sources of bias

**Table:** Tonarelli 2018

***Study characteristics***		
Methods	**Study design:** randomised controlled trial **Study grouping:** parallel group	
Participants	**Baseline characteristics** Experimental group: expressive writing *Age (mean ± SD)*: NR *Sex (N (% female))*: NR *Sample size*: 15 *Years of experience (mean ± SD)*: NR Control group: neutral writing instruction *Age (mean ± SD)*: NR *Sex (N (% female))*: NR *Sample size*: 11 *Years of experience (mean ± SD)*: NR Overall *Age (mean ± SD)*: 46 (nr) *Sex (N (% female))*: NR *Sample size*: 26 *Years of experience (mean ± SD)*: NR **Included criteria:** professionals that work in the Palliative Care field, speak and write Italian **Excluded criteria:** NR **Pretreatment:** NR **Compliance rate:** NR **Response rate:** NR **Type of healthcare worker:** palliative healthcare workers	
Interventions	**Intervention characteristics** Experimental group: expressive writing *Type of the intervention*: Intervention type 1 ‐ to focus one’s attention on the experience of stress *Description of the intervention*: Expressive writing protocol. Expressive writing is a tool through which the person describes his/her most profound thoughts and feelings about emotional events *The number of sessions*: Expressive writing is a tool through which the person describes his/her most profound thoughts and feelings about emotional events *Duration of each session on average*: 20 minutes *Duration of the entire intervention*: 3 days *Duration of the entire intervention short vs long*: short *Intervention deliverer*: NR *Intervention form*: individual Control group: neutral writing instruction *Type of the intervention*: Active control *Description of the intervention*: NA *The number of sessions*: NA *Duration of each session on average*: NA *Duration of the entire intervention*: NA *Duration of the entire intervention short vs long*: NA *Intervention deliverer*: NA *Intervention form*: NA	
Outcomes	*Maslach Burnout Inventory ‐ Emotional Exhaustion* **Outcome type**: ContinuousOutcome *Maslach Burnout Inventory ‐ Depersonalisation* **Outcome type**: ContinuousOutcome *Maslach Burnout Inventory ‐ Personal accomplishment (lack of)* **Outcome type**: ContinuousOutcome	
Identification	**Sponsorship source:** NR **Country:** Italy **Setting:** Palliative care and hospice **Comments:** NR **Authors name:** Annalisa Tonarelli **Institution:** Department of Medicine and Surgery, University of Parma, Italy; **Email:** annalisa.tonarelli@unipr.it **Address:** Annalisa TonarelliDepartment of Medicine and Surgery, University of Parma, ItalyVia Gramsci 14 ‐ 43126, Parma, Italy **Time period:** NR	
Notes	Not able to include in analysis due to missing data.	
***Risk of bias***		
**Bias**	**Authors' judgement**	**Support for judgement**
Random sequence generation (selection bias)	Unclear risk	Quote: "Participants who expressed the desire to partici‐ pate in the study, after signing the informed consent, were assigned by randomization" No details on how randomisation sequence was generated.
Allocation concealment (selection bias)	Unclear risk	Not described well enough
Blinding of participants and personnel (performance bias) All outcomes	High risk	Participants not blinded.
Blinding of outcome assessment (detection bias) All outcomes	High risk	Participants were not blinded whereas outcomes are self‐reported.
Incomplete outcome data (attrition bias) All outcomes	Unclear risk	Not described
Selective reporting (reporting bias)	Unclear risk	No registration protocol nor did we find one online
Other bias	Unclear risk	Did not report reponse rate, compliance rate, differences between the groups at baseline

**Table:** Tsai 1993

***Study characteristics***		
Methods	RCT, Taiwan	
Participants	137 nurses	
Interventions	1) Experimental: Training about stress at work, relaxation, breathing, imagery and meditation: One 90‐minute session in each of 2 weeks and 1 follow‐up session in the 5th week. Training covered: sources of stress at work, relaxation as a coping method and meditation including breathing exercise and imagery that emphasised the underlying cognitive process of meditation. 2) Control: traditional in‐service education about theory analysis	
Outcomes	Nurse Stress Checklist, Chinese General Health Questionnaire	
Identification		
Notes	GHQ included in analysis 2.1.	
***Risk of bias***		
**Bias**	**Authors' judgement**	**Support for judgement**
Random sequence generation (selection bias)	Low risk	Quote: "For each unit, a coin was thrown to select which nurse from this unit would be assigned to either the experimental or control group." (p. 56)
Allocation concealment (selection bias)	Unclear risk	Not reported
Blinding of participants and personnel (performance bias) All outcomes	High risk	Participants not blinded.
Blinding of outcome assessment (detection bias) All outcomes	High risk	Participants were not blinded whereas outcomes are self‐reported.
Incomplete outcome data (attrition bias) All outcomes	Unclear risk	It is unclear if any participants dropped out
Selective reporting (reporting bias)	Low risk	All outcomes reported
Other bias	Low risk	We did not find any indications of other sources of bias

**Table:** Verdes Montenegro Atalaya 2021

***Study characteristics***		
Methods	**Study design:** cluster‐randomised controlled trial **Study grouping:** parallel group	
Participants	**Baseline characteristics** Experimental group 1: MRBS 4 weeks *Age (mean ± SD)*: 47.7 ± 13.7 *Sex (N (% female))*: 18 (75%) *Sample size*: 24 *Years of experience (mean ± SD)*: 19.5 ± 13.9 Experimental group 1: MRBS 8 weeks *Age (mean ± SD)*: 35.7 ± 12 *Sex (N (% female))*: 28 (76%) *Sample size*: 37 *Years of experience (mean ± SD)*: 8.9 ± 11 Control: no intervention *Age (mean ± SD)*: 40.3 ± 13 *Sex (N (% female))*: 40 (78%) *Sample size*: 51 *Years of experience (mean ± SD)*: 13.1 ± 13 Overall *Age (mean ± SD)*: 41.6 ± 12 *Sex (N (% female))*: 86 (76.8) *Sample size*: 112 *Years of experience (mean ± SD)*: 12.9 ± 13.2 **Included criteria:** NR **Excluded criteria:** NR **Pretreatment:** At baseline, statistically significant differences were found between the three groups in age, professional type, and work experience. **Compliance rate:** NR **Response rate:** NR **Type of healthcare worker:** various types of healthcare workers	
Interventions	**Intervention characteristics** Experimental group 1: MRBS 4 weeks *Type of the intervention*: Intervention type 1 ‐ to focus one’s attention on the experience of stress *Description of the intervention*: MBSR training program complemented with practices of the Mindful Self‐Compassion (MSC) program *The number of sessions*: 4 *Duration of each session on average*: 2.5 hours *Duration of the entire intervention*: 4 weeks *Duration of the entire intervention short vs long*: short *Intervention deliverer*: NR *Intervention form*: group Experimental group 1: MRBS 8 weeks *Type of the intervention*: Intervention type 1 ‐ to focus one’s attention on the experience of stress *Description of the intervention*: MBSR training program complemented with practices of the Mindful Self‐Compassion (MSC) program *The number of sessions*: 8 *Duration of each session on average*: 2.5 hours *Duration of the entire intervention*: 8 weeks *Duration of the entire intervention short vs long*: short *Intervention deliverer*: NR *Intervention form*: group Control: no intervention *Type of the intervention*: NA *Description of the intervention*: NA *The number of sessions*: NA *Duration of each session on average*: NA *Duration of the entire intervention*: NA *Duration of the entire intervention short vs long*: NA *Intervention deliverer*: NA *Intervention form*: NA	
Outcomes	PSQ **Outcome type**: ContinuousOutcome	
Identification	**Sponsorship source:** NR **Country:** Spain **Setting:** Spanish National Health System teaching units **Comments:** NR **Authors name:** Juan Carlos Verdes‐Montenegro‐Atalaya **Institution:** Family and Community Medicine Teaching Department of Burgos, 0 **Email:** juancarlosverdesm@yahoo.es **Address:** Family and Community Medicine Teaching Department of Burgos, 09006 Burgos, Spain **Time period:** NR	
Notes	PHQ included in analysis 1.1. Intervention groups combined to create a single pair‐wise comparison	
***Risk of bias***		
**Bias**	**Authors' judgement**	**Support for judgement**
Random sequence generation (selection bias)	Unclear risk	Quote: "Each TU was considered as a different and independent cluster, randomly assigned to the CG (2 TUs) or one of the two EGs (4 TUs)." Insufficiently described.
Allocation concealment (selection bias)	Unclear risk	Quote: "EG1 participants were included in a standard training program of mindfulness and self‐compassion; while EG2, in an abbreviated one. Furthermore, the participants from each TU were stratiﬁed according to the type of professional (66 tutors versus 66 resident intern specialists)." Insufficient information to understand whether intervention allocations could have been foreseen in advance of, during, enrolment.
Blinding of participants and personnel (performance bias) All outcomes	High risk	Participants not blinded.
Blinding of outcome assessment (detection bias) All outcomes	High risk	Participants were not blinded whereas outcomes are self‐reported.
Incomplete outcome data (attrition bias) All outcomes	Low risk	53 of the 165 (32%) randomised participants lost to follow‐up. Reasons provided. The baseline characteristics of participants who dropped out of the study were similar to those who completed it, so systematic selectionbias is unlikely.
Selective reporting (reporting bias)	Unclear risk	No trial registration or no study protocol reported nor did we find one online
Other bias	Unclear risk	Statistically significant differences were observed between the three groups in age, type of professional, and time working in the Spanish National Health System

**Table:** Wei 2017

***Study characteristics***		
Methods	**Study design:** randomised controlled trial **Study grouping:** parallel group	
Participants	**Baseline characteristics** Experimental group: active intervention and regular management. *Age (mean ± SD)*: NR *Sex (N (% female))*: NR *Sample size*: 51 *Years of experience (mean ± SD)*: NR Control (no intervention) *Age (mean ± SD)*: NR *Sex (N (% female))*: NR *Sample size*: 51 *Years of experience (mean ± SD)*: NR Overall *Age (mean ± SD)*: NR *Sex (N (% female))*: NR *Sample size*: 102 *Years of experience (mean ± SD)*: NR **Included criteria:** NR **Excluded criteria:** NR **Pretreatment:** there were no significant differences between the control group and intervention group nurses in the job burnout scales before the intervention **Compliance rate:** NR **Response rate:** NR **Type of healthcare worker:** exclusively nurses	
Interventions	**Intervention characteristics** Experimental group: active intervention and regular management. *Type of the intervention*: Intervention type 1 ‐ to focus one’s attention on the experience of stress *Description of the intervention* : Classes pertaining to communication skills, approaches to conflict, efficacy elevation, and emotion control, as well as working skills *The number of sessions*: NR *Duration of each session on average*: 30 minutes *Duration of the entire intervention*: 6 months *Duration of the entire intervention short vs long*: long *Intervention deliverer*: Nurse managers *Intervention form*: group Control (no intervention) *Type of the intervention*: NA *Description of the intervention*: NA *The number of sessions*: NA *Duration of each session on average*: NA *Duration of the entire intervention*: NA *Duration of the entire intervention short vs long*: NA *Intervention deliverer*: NA *Intervention form*: NA	
Outcomes	*Maslach Burnout Inventory ‐ Emotional Exhaustion* **Outcome type**: ContinuousOutcome *Maslach Burnout Inventory ‐ Depersonalisation* **Outcome type**: ContinuousOutcome *Maslach Burnout Inventory ‐ Personal accomplishment (lack of)* **Outcome type**: ContinuousOutcome	
Identification	**Sponsorship source:** NR **Country:** China **Setting:** Hospital **Comments:** NA **Authors name:** Rong Wei **Institution:** Department of Clinical Nursing, Qianfoshan Hospital, Jinan, Shandong, China. **Email:** lijianxin0531@126.com **Address:** Jianxin Li, Department of Clinical Nursing, Qianfoshan Hospital, NO. 16766, Jingshi Road, Jinan, Shandong, China; **Time period:** NR	
Notes	MBI‐EE included in analysis 1.1.	
***Risk of bias***		
**Bias**	**Authors' judgement**	**Support for judgement**
Random sequence generation (selection bias)	Unclear risk	Quote: "total of 112 registered nurses were randomly selected from 3 of 8 comprehensive high‐level hospitals in Jinan, China." Only mentioned that nurses where randomly selected.
Allocation concealment (selection bias)	Unclear risk	Unable to judge whether participants and/or investigators could possible foresee assignment
Blinding of participants and personnel (performance bias) All outcomes	High risk	Participants not blinded.
Blinding of outcome assessment (detection bias) All outcomes	High risk	Participants not blinded whereas outcomes are self‐reported.
Incomplete outcome data (attrition bias) All outcomes	Low risk	No loss to follow‐up.
Selective reporting (reporting bias)	Unclear risk	No trial registration or no study protocol reported, nor did we find one online
Other bias	Unclear risk	Compliance rate and response rate not reported.

**Table:** West 1984

***Study characteristics***		
Methods	RCT, USA	
Participants	60 acute care hospital nurses	
Interventions	1) Experimental: four weeks of Stress Inoculation (SI) training divided as: i) Education‐only group (Ed) weekly 30‐minute sessions of information about anxiety, stress and coping skills and practice of self‐monitoring of stress producing events (n = 12) ii) Education + coping skills group (CS), 4 weekly 60‐minute sessions including education plus CS. CS: relaxation training, assertive skill training, cognitive restructuring and time‐management instruction (n = 12) iii) Education + exposure group (Ex) 4 weekly 60‐minute including education plus simulated stress‐producing situations via role play (n = 12) iv) Education + coping skills + exposure group (SI) 60‐minute sessions twice a week during 4 weeks including all the above (n = 12) 2) Control: No intervention (n = 12)	
Outcomes	MBI (used frequency and intensity separately for each subscale); we used Emotional Exhaustion intensity scores. Job‐Related Tension Index, Life Satisfaction Index, STAI, Rathus Assertiveness Schedule, systolic and diastolic blood pressure	
Identification		
Notes	Results are only presented for the group including CS (n = 24) versus education plus no‐intervention (n = 24). MBI‐EE included in analysis 1.1 and STAI included in analysis 1.4.	
***Risk of bias***		
**Bias**	**Authors' judgement**	**Support for judgement**
Random sequence generation (selection bias)	Unclear risk	Quote: "...60 registered nurses... were stratified on the basis of work shift and randomly assigned to 1 of 6 counselors and one of five treatment conditions." (p. 212)
Allocation concealment (selection bias)	Unclear risk	Not reported
Blinding of participants and personnel (performance bias) All outcomes	High risk	Participants not blinded.
Blinding of outcome assessment (detection bias) All outcomes	High risk	Participants were not blinded whereas outcomes are self‐reported.
Incomplete outcome data (attrition bias) All outcomes	Low risk	Quote: "No subject attrition occurred at posttesting." (p. 213)
Selective reporting (reporting bias)	Low risk	All outcomes reported
Other bias	Low risk	We did not find any indications of other sources of bias

**Table:** West 2014

***Study characteristics***		
Methods	**Study design:** randomised controlled trial **Study grouping:** parallel group	
Participants	**Baseline characteristics** Protected time with facilitated small group curriculum *Age (mean ± SD)*: NR *Sex (N (% female))*: NR *Sample size*: NR *Years of experience (mean ± SD)*: NR Control (protected time unstructured) *Age (mean ± SD)*: NR *Sex (N (% female))*: NR *Sample size*: NR *Years of experience (mean ± SD)*: NR Overall *Age (mean ± SD)*: NR *Sex (N (% female))*: 25 (34%) *Sample size*: 74 *Years of experience (mean ± SD)*: NR **Included criteria:** practising physicians in the Mayo Clinic Department of Medicine **Excluded criteria:** not specified **Pretreatment:** baseline characteristics were similar for both groups with no statistically significant differences observed. However, the intervention group had "slightly higher rates of high emotional exhaustion and overall burnout." P values for statistical significance are not provided in the paper. **Compliance rate:** NR **Response rate:** 13% **Type of healthcare worker:** physicians	
Interventions	**Intervention characteristics** Protected time with facilitated small group curriculum *Type of the intervention*: Intervention type 4 ‐ Combination of interventions *Description of the intervention*: Quote: "Participants randomized to the intervention arm engaged in a facilitated small‐group curriculum administered at 1‐hour meetings occurring once every 2 weeks for 9 months, for a total of 19 sessions. The 37 intervention arm participants were divided into 4 small groups (8‐10 physicians each) with similar compositions by sex and specialty. Topics addressed during these sessions were organised into modules entitled“self,” “patient,” and “balance” and included meaning in work, personal and professional balance, medical mistakes, community, caring for patients, and other topics relevant to the work experiences of practising physicians. Each session followed the same general structure: (1) check‐in and welcome, (2) preparing the environment (eg, journaling and reflective exercise), (3) facilitated group discussion, (4) learned skills and solutions, and (5) check‐out and summary *The number of sessions*: 19 *Duration of each session on average*: 1 hour *Duration of the entire intervention*: 36 weeks *Duration of the entire intervention short vs long*: Long *Intervention deliverer*: "Practising internal medicine physicians with specific expertise in communication and teaching courses involving small‐group facilitation." *Intervention form*: Group Control (protected time unstructured) *Type of the intervention*: NA *Description of the intervention*: "Those in the control arm could schedule and use this hour of protected time in any manner they believed was most useful but did not participate in the formal curriculum." *The number of sessions*: NA *Duration of each session on average*: NA *Duration of the entire intervention*: NA *Duration of the entire intervention short vs long*: NA *Intervention deliverer*: NA *Intervention form*: NA	
Outcomes	*Maslach Burnout Inventory ‐ Emotional Exhaustion* **Outcome type**: ContinuousOutcome *Maslach Burnout Inventory ‐ Depersonalisation* **Outcome type**: ContinuousOutcome *Perceived Stress Scale (PSS)* **Outcome type**: ContinuousOutcome	
Identification	**Sponsorship source:** Mayo Clinic Program on Professionalism and Ethics and the Department of Medicine at Mayo Clinic, Rochester, USA. **Country:** USA **Setting:** One medical centre **Comments:** NR **Authors name:** Colin P. West **Institution:** Division of General Internal Medicine, Department of Medicine, Mayo Clinic **Email:** west.colin@mayo.edu **Address:** Division of General Internal Medicine, Department of Medicine, Mayo Clinic, 200 First St, Rochester, MN 55905, USA. **Time period:** 2010‐2012	
Notes	We kindly receivede the mean and SD for the primary outcome from author C. West. PSS included in analysis 4.1 and 4.2.	
***Risk of bias***		
**Bias**	**Authors' judgement**	**Support for judgement**
Random sequence generation (selection bias)	Low risk	Quote: "Participants were randomized in a concealed fashion into 2 groups via a computer‐generated algorithm."
Allocation concealment (selection bias)	Low risk	Participants were randomized in a concealed fashion into 2 groups via a computer generated algorithm
Blinding of participants and personnel (performance bias) All outcomes	High risk	Participants not blinded.
Blinding of outcome assessment (detection bias) All outcomes	High risk	Participants not blinded whereas outcomes are self‐reported.
Incomplete outcome data (attrition bias) All outcomes	Low risk	Low response rate. 74 of the 565 eligible physicians participated (13%)
Selective reporting (reporting bias)	Low risk	Data we kindly received match trial registration: NCT01159977
Other bias	Low risk	No indication of other source of bias.

**Table:** West 2021

***Study characteristics***		
Methods	**Study design:** randomised controlled trial **Study grouping:** parallel group	
Participants	**Baseline Characteristics** Experimental group: topic focused discussion *Age (< 30, 31‐40, 41‐50, 51‐60, > 60)(%))*: 1 (12), 24 (39), 17(27%), 15,(24%) 5 (8%) *Sex (N (% female))*: 27 (43%) *Sample size* : 62 *Years of experience (mean ± SD)*: NR Control (no intervention) *Age (< 30, 31‐40, 41‐50, 51‐60, > 60)(%))*: 0(0%), 16(26%), 20(33%), 18(30%), 7(12%) *Sex (N (% female))*: 26 (43%) *Sample size*: 61 *Years of experience (mean ± SD)*: NR Overall *Age (< 30, 31‐40, 41‐50, 51‐60, > 60)(%))*: NR *Sex (N (% female))*: 54 (43%) *Sample size*: 123 *Years of experience (mean ± SD)*: NR **Included criteria:** NR **Excluded criteria:** NR **Pretreatment:** NR **Compliance rate:** NR **Response rate:** 21% **Type of healthcare worker:** physicians	
Interventions	**Intervention characteristics** Experimental group: topic focused discussion *Type of the intervention*: 1. Intervention type 1 ‐ to focus one’s attention on the experience of stress *Description of the intervention*: Group discussions about several topics: organised into categories entitled “meaning in work/job satisfaction,” “teamwork/social support/collegiality/relationships/work‐life balance and integration,” and “personal strengths/problem‐solving/coping/resources for thriving and flourishing *The number of sessions*: 12 *Duration of each session on average*: 15 minutes & afterwards 45 minutes *Duration of the entire intervention*: 6 months *Duration of the entire intervention short vs long*: long *Intervention deliverer* : NR *Intervention form*: group Control (no intervention) *Type of the intervention*: No intervention *Description of the intervention*: NA *The number of sessions*: NA *Duration of each session on average*: NA *Duration of the entire intervention*: NA *Duration of the entire intervention short vs long*: NA *Intervention deliverer*: NA *Intervention form*: NA	
Outcomes	*Maslach Burnout Inventory ‐ Emotional Exhaustion* **Outcome type**: ContinuousOutcome *Maslach Burnout Inventory ‐ Depersonalisation* **Outcome type**: ContinuousOutcome *Maslach Burnout Inventory ‐ Personal accomplishment (lack of)* **Outcome type**: ContinuousOutcome	
Identification	**Sponsorship source:** NR **Country:** United States of America **Setting:** Hospital **Comments:** NR **Authors name:** Colin West **Institution:** Department of Quantitative Health Sciences Mayo Clinic, Rochester, MN; and the Division of Hematology, **Email:** west.colin@mayo.edu **Address:** Department of Medicine, Stanford University, Palo Alto, CA **Time period:** 2013‐2014	
Notes	We kindly receivede the mean and SD for the primary outcome from author C. West MBI‐EE included in analysis 1.1 and 1.2.	
***Risk of bias***		
**Bias**	**Authors' judgement**	**Support for judgement**
Random sequence generation (selection bias)	Low risk	Quote: "Participants were randomized in concealed fashion into two arms via a computer‐ generated algorithm."
Allocation concealment (selection bias)	Low risk	Participants were randomized in concealed fashion into two arms via a computer‐ generated algorithm.
Blinding of participants and personnel (performance bias) All outcomes	High risk	Participants not blinded.
Blinding of outcome assessment (detection bias) All outcomes	High risk	Participants not blinded whereas outcomes are self‐reported.
Incomplete outcome data (attrition bias) All outcomes	Low risk	107 of the 123 randomized participants (87%) complete follow‐up data.
Selective reporting (reporting bias)	Low risk	Registation trial checked: NCT04466423. No indication of selective outcome reporting.
Other bias	Unclear risk	Compliance not reported.

**Table:** Xie 2020

***Study characteristics***		
Methods	**Study design:** randomised controlled trial **Study grouping:** parallel group	
Participants	**Baseline characteristics** Educational group *Age (mean ± SD)*: 27.7 ± 7.7 *Sex (N (% female)*: 53 (100%) *Sample size*: 53 *Years of experience (mean ± SD)*: NR Mindfulness‐based intervention *Age (mean ± SD)*: 27.9 ± 4.9 *Sex (N (% female)*: 53 (100%) *Sample size*: 53 *Years of experience (mean ± SD)*: NR Overall *Age (mean ± SD)*: 27.7 ± 7.7 *Sex (N (% female)*: 106 (100%) *Sample size*: 106 *Years of experience (mean ± SD)*: NR **Included criteria:** a) they had obtained professional certification; (b) they were independently responsible for clinical work; (c) they were willing to participate and available to attend sessions; and (d) they were suffering from“moderate or above” occupational burnout **Excluded criteria:** (a) they had been in work for less than one year; (b) they were on vacation or on study leave; (c) they had taken a mindfulness program such as MBSR or MBCT and had been practising mindfulness in the last six months. **Pretreatment:** reported socio demographic baseline characteristics of participants randomised to the intervention group were similar to socio demographic baseline characteristics of participants randomised to the control group **Compliance rate:** EB (46/53 87%). MBIB (45/53 85%) **Response rate:** 63% **Type of healthcare worker:** exclusively nurses	
Interventions	**Intervention characteristics** Educational group *Type of the intervention*: Intervention type 1 ‐ to focus one’s attention on the experience of stress *Description of the intervention* : Educational sessions which entailed training and information on: (a) the nature of occupational burnout; (b) the incidence and adverse effects of occupational burnout among ICU nurses; (c) cognition of emotional exhaustion, depersonalisation, and personal accomplishment; and (d)how to alleviate occupational burnout, including emotional manage‐ment, having friendly engagement with patients, and the value of work *The number of sessions*: 2 *Duration of each session on average*: 1.5 hours *Duration of the entire intervention*: 4 weeks *Duration of the entire intervention short vs long* : short *Intervention deliverer*: ICU head nurse *Intervention form*: Group Mindfulness‐based intervention *Type of the intervention*: Intervention type 2 ‐ to focus one’s attention away from the experience of stress *Description of the intervention*: Mindfulness program, which was based on the theory of mindfulness therapy, including MBSR, MBCT, ACT, and loving‐kindness and compassion meditation *The number of sessions*: 8 *Duration of each session on average*: 2.5 hours *Duration of the entire intervention*: 8 weeks *Duration of the entire intervention short vs long*: short *Intervention deliverer*: Counsellor *Intervention form*: Group	
Outcomes	*Maslach Burnout Inventory ‐ Emotional Exhaustion* **Outcome type**: ContinuousOutcome *Maslach Burnout Inventory ‐ Depersonalisation* **Outcome type**: ContinuousOutcome *Maslach Burnout Inventory ‐ Personal accomplishment (lack of)* **Outcome type**: ContinuousOutcome	
Identification	**Sponsorship source:** NR **Country:** China **Setting:** Hospital **Comments:** NR **Authors name:** Caixia Xie **Institution:** Nursing School, West China School of Medicine/West China Hospital, Sichuan University **Email:** huxiuying@scu.edu.cn xiuying_hu@163.com **Address:** Chengdu 610041, Sichuan, China **Time period:** 2017‐2018	
Notes	MBI‐EE included in analysis 5.1.	
***Risk of bias***		
**Bias**	**Authors' judgement**	**Support for judgement**
Random sequence generation (selection bias)	Unclear risk	Quote: "Participants were assigned to the EB or the MBIB group. This" Sequence generation process insufficiently described.
Allocation concealment (selection bias)	Unclear risk	Quote: "Participants in the EB group were randomly divided into three subgroups, consisting of 18, 17, and 17 individuals, respectively." Insufficient information to understand whether intervention allocations could have been foreseen in advance of, during, enrolment.
Blinding of participants and personnel (performance bias) All outcomes	High risk	Participants not blinded.
Blinding of outcome assessment (detection bias) All outcomes	High risk	Participants not blinded whereas outcomes are self‐reported.
Incomplete outcome data (attrition bias) All outcomes	Low risk	No loss at follow‐up
Selective reporting (reporting bias)	Low risk	Trial registration in Chinese. No indication of selective reporting.
Other bias	Low risk	No indication of other sources of bias

**Table:** Yazdani 2010

***Study characteristics***		
Methods	RCT, Iran	
Participants	76 nursing students Quote: "The study population included all male and female nursing students who were studying in Isfahan Nursing and Midwifery university in 2010‐2011, in the second and third years (third, fourth, fifth and sixth semesters). 72 [sic] students were randomly assigned to two groups using the list of students studying in the second and third year in 2010‐2011 and based on the odd and even numbers." (p. 210) "The groups were heterogeneous in terms of gender..." (p. 210)	
Interventions	1) Experimental: Stress management training (n = 38). Quote: "[First group (n=38) trained stress management training program (8 two hours sessions, twice a week). And second group (n = 38) did not received [sic] training." (p. 210) The stress management program consisted of: information about stress, gradual muscle relaxation and its implementation with mental imagery, consequences and physical symptoms of stress, relaxation and imagery and training and diaphragm breathing practices, linking thoughts and emotions and familiarity with cognitive errors, discussion about relaxation exercises and replacement of logical thoughts and personal stress management program. 2) Control: no intervention (n = 38)	
Outcomes	DASS‐42: Depression, Anxiety and Stress Scale	
Identification		
Notes	DASS‐stress included in analysis 2.1 and DASS‐depression in analysis 2.3.	
***Risk of bias***		
**Bias**	**Authors' judgement**	**Support for judgement**
Random sequence generation (selection bias)	High risk	Quote: "...students were randomly assigned to two groups using the list of students studying in the second and third year in 2010‐2011 and based on the odd and even numbers." (p. 210)
Allocation concealment (selection bias)	Unclear risk	Not reported if group allocation was concealed
Blinding of participants and personnel (performance bias) All outcomes	High risk	Participants not blinded.
Blinding of outcome assessment (detection bias) All outcomes	High risk	Participants were not blinded whereas outcomes are self‐reported.
Incomplete outcome data (attrition bias) All outcomes	Unclear risk	It is difficult to tell if some participants were lost to follow‐up or not as the authors give three separate numbers for the amount of participants. Quote: "This study was a parallel ‐group randomized quasi‐experimental trial...on 68 BSc nursing students.", " 72 students were randomly assigned..." and "Finally seventy‐six subjects elected among them." (p. 210)
Selective reporting (reporting bias)	Low risk	The study had only one outcome and its results are all reported
Other bias	Low risk	We did not find any indications of other sources of bias

**Table:** Yung 2004

***Study characteristics***		
Methods	RCT, China	
Participants	65 nurse managers	
Interventions	1) Experimental 1: cognitive relaxation: participants were asked to imagine the relaxation of different muscle groups. 2) Experimental 2: stretch‐release relaxation: training guided by the model of Stretch Relaxation developed by Carlson and Collins (1990) which focused on the stretching and relaxation of muscle groups. Unlike the popular progressive relaxation exercise which involves the tensing and relaxing of muscle groups, stretch‐release relaxation is less strenuous. Muscle relaxation exercise, based upon the stretching of muscle groups, incorporates the beneficial effects of muscle sensation contrast with accompanied reductions in muscle activity from the stretch procedure resulting in relaxation. 3) Control: No intervention	
Outcomes	C‐STAI, C‐GHQ	
Identification		
Notes	GHQ included in analysis 2.1 and C‐STAI in analysis 2.3. Intervention groups combined to create a single pair‐wise comparison.	
***Risk of bias***		
**Bias**	**Authors' judgement**	**Support for judgement**
Random sequence generation (selection bias)	Unclear risk	Quote: "Of the 65 participants, 35 were randomly assigned to the experimental condition and the remaining 30 were put to the control condition. Subsequently, the 35 subjects assigned to the experimental condition were randomly allocated to the stretch‐release relaxation (n = 17) and cognitive relaxation (n = 18) groups." (p. 256)
Allocation concealment (selection bias)	Unclear risk	Not reported
Blinding of participants and personnel (performance bias) All outcomes	High risk	Participants not blinded.
Blinding of outcome assessment (detection bias) All outcomes	High risk	Participants were not blinded whereas outcomes are self‐reported.
Incomplete outcome data (attrition bias) All outcomes	Unclear risk	Quote: "All participants including the TC [test control] group were assessed again in a follow‐up session after 1 month." (p. 258). Insufficiently recorded to judge attrition bias
Selective reporting (reporting bias)	Low risk	All outcomes reported.
Other bias	Low risk	We did not find any indications of other sources of bias

**Table:** Zarvijani 2021

***Study characteristics***		
Methods	**Study design:** randomised controlled trial **Study grouping:** parallel group	
Participants	**Baseline characteristics** Acceptance and Commitment Therapy (ACT) *Age (mean ± SD)*: NR *Sex (N (% female))*: 19 (54%) *Sample size*: 35 *Years of experience (mean ± SD)*: NR Control (no intervention ‐ routine support) *Age (mean ± SD)*: NR *Sex (N (% female))*: 18 (51%) *Sample size*: 35 *Years of experience (mean ± SD)*: NR Overall *Age (mean ± SD)*: NR *Sex (N (% female))*: 37 (53%) *Sample size*: 70 *Years of experience (mean ± SD)*: NR **Included criteria:** ‐ having a bachelor’s or higher degree in nursing, ‐ at least 2 years of work experience in psychiatric wards, ‐ attending intervention sessions based on ACT for the first time, and ‐ no history of taking psychiatric drugs in the past and present. **Excluded criteria:** ‐ not completing the questionnaires, ‐ absenteeism in more than one intervention session, and ‐ the occurrence of stressful events during the study. **Pretreatment:** statistical difference not reported. Only numbers and proportions were reported which were similar on most variables but no statistical reporting. **Compliance rate:** 94%. two out of 35 participants in the intervention group were excluded since they missed more than one session out of eight, i.e. 33 of the participants attended 6 or more sessions. **Response rate:** NR. 84 psychiatric nurses from all 23 wards in the hospital were randomly selected and screened for eligibility proportional to the required sample size for the study. However, the total number of nurses in the facility is not reported. **Type of healthcare worker:** psychiatric nurses	
Interventions	**Intervention characteristics** Acceptance and Commitment Therapy (ACT) *Type of the intervention*: Intervention type 1 ‐ to focus one’s attention on the experience of stress *Description of the intervention*: ACT‐based training according to Steven Hayes model in eight two‐hour sessions conducted by an ACT therapist *The number of sessions*: 8 *Duration of each session on average*: 2 hours *Duration of the entire intervention*: NR *Duration of the entire intervention short vs long*: NR *Intervention deliverer*: Therapist *Intervention form*: Face‐to‐face Control (no intervention ‐ routine support) *Type of the intervention*: NA *Description of the intervention*: Routine interventions such as stress control, life skills, and anger control workshops, which are normally held by the educational deputy of the centre for the staff. *The number of sessions*: NR *Duration of each session on average*: NR *Duration of the entire intervention*: NR *Duration of the entire intervention short vs long*: NR *Intervention deliverer*: NR *Intervention form*: NR	
Outcomes	*Perceived Stress Scale (PSS)* **Outcome type**: ContinuousOutcome	
Identification	**Sponsorship source:** Islamic Azad University, Tehran. **Country:** Iran **Setting:** One large psychiatric hospital **Comments:** NR **Authors name:** Ladan Fattah Moghaddam **Institution:** Tehran University of Medical Sciences, Islamic Azad University, Tehran, Iran **Email:** lfatah@iautmu.ac.ir **Address:** Department of Psychiatric Nursing, Faculty of Nursing and Midwifery, Tehran University of Medical Sciences, Islamic Azad University, Tehran, Iran **Time period:** 2018	
Notes	PSS included in analysis 1.1.	
***Risk of bias***		
**Bias**	**Authors' judgement**	**Support for judgement**
Random sequence generation (selection bias)	Unclear risk	Quote: "Of the 84 nurses selected, fourteen were excluded due to lack of inclusion criteria, and 70 remaining nurses were each assigned a number and were randomly divided into experimental and control groups, each consisting of 35 participants. (Fig. 1). Sequence generation process insufficiently described.
Allocation concealment (selection bias)	Unclear risk	Allocation concealment not recorded. Difficult to judge whether participants and/or investigators could possibly foresee assignment
Blinding of participants and personnel (performance bias) All outcomes	High risk	Participants were not blinded.
Blinding of outcome assessment (detection bias) All outcomes	High risk	Participants were not blinded whereas outcomes are self‐reported.
Incomplete outcome data (attrition bias) All outcomes	Low risk	Attrition rate was less than 20% and a priori outcomes were reported.
Selective reporting (reporting bias)	Low risk	Trial registration IRCT20180506039557N1. No indication of selective reporting
Other bias	Unclear risk	Response rate could not be calculated since total number of nurses was not provided. Statistical differences if any on baseline parameters not reported.

**ACT:** Acceptance and Commitment Therapy; **BP:** blood pressure; **BSI:** Brief Symptom Inventory; **DCWs:** Direct Care Workers' **ERT:** Emotion regulation training; **ESRT‐1:** Enhanced stress resilience training‐1; **GHQ:** General Health Questionnaire; **HCW:** healthcare worker; **HR:** heart rate; **ICU**: intensive care unit; **MBI:** Maslach Burnout Inventory; **MBSR:** Mindfulness‐based stress reduction; **NLGNs:** newly incensed graduate nurses; **PSQ:** Perceived Stress Questionnaire; **PSS:** Perceived Stress Scale; **SD:** standard deviation; **SMI:** Stress Management Intervention; **TTI:** Transfer Technique Intervention; **PTSD:** Post‐traumatic stress disorder; **STAI:** State‐Trait Anxiety Inventory; **WCG:** waitist control group

### Characteristics of excluded studies [ordered by study ID]

**Table:**

Study	Reason for exclusion
Adair 2020	Wrong population: not only healthcare workers
Akyurek 2020	Wrong comparison (intervention type 2 vs intervention type 2)
Ali 2011	Wrong intervention: organisational intervention
Alkhawaldeh 2020	Wrong outcome (stressor) ‐ trial protocol checked.
Ameli 2020	Wrong population: not only healthcare workers
Anonymous 2021	Not an article, wrong population: consisted of not only healthcare workers (original study considered) .
Aronson 2022	Wrong intervention (organisational intervention).
Barbosa 2016	Wrong population: caregivers of family not officially employed
Barrett 2021	Wrong comparison (intervention type 1 vs intervention type 1)
Beck 2018	Wrong population: students
Behzadi 2021	Wrong outcome (stressor)
Bielderman 2021	Wrong intervention: organisational intervention
Bittman 2003	Wrong study design
Boehm 2017	Thesis (not an article in a peer‐reviewed scientific journal)
BorgesSouza 2019	Not an article
Bourbonnais 2011	Wrong intervention:organisational intervention
Braun 2020	Wrong population: students
Buruck 2016	Wrong study design
Butow 2015	Wrong intervention
Carneiro 2020	Wrong population: not only healthcare workers
Carson 1999	Wrong intervention: organisational intervention
Cascales Perez 2021	Wrong population: not only healthcare workers
Cayir 2021	Wrong intervention: organisational intervention.
Chaabane 2021	Wrong population: also volunteers
Chen 2017	Wrong intervention
Cheung 2020	Wrong comparison (intervention type 2 vs intervention type 2)
Clemow 2018	Wrong population: not only healthcare workers
Coelhoso 2019	Wrong population: not only healthcare workers
Cordoza 2018	Wrong intervention
Cosentino 2021	Wrong study design
Daigle 2018	Wrong outcome assessment: use of unvalidated questionnaire
Davidson 2017	Wrong study design
DeKock 2022	Wrong outcomes. Trial protocol checked.
Delvaux 2004	Wrong outcome (stressor)
Deneckere 2013	Wrong intervention: organisational intervention
Doran 2018	Wrong population: not only healthcare workers
Fadaei 2020	Wrong outcome assessment: use of unvalidated questionnaire
Fang 2015	Wrong outcome (stressor)
Farsi 2021	Wrong outcome (stressor)
Feldman 2017	Wrong study design
Fiore 2021	Wrong comparison (intervention type 2 vs intervention type 2)
Frogeli 2016	Wrong comparison (intervention type 1 vs intervention type 1)
Gaggioli 2014	Wrong population: not only healthcare workers
Gardner 2005	Wrong population: not only healthcare workers
Gartner 2013	Duplicate
Gauthier 2015	Wrong study design
Ghafarzadegan 2014	Wrong intervention
Ghazavi 2010	Use of unvalidated questionnaire.
Grahn 2020	Not an article
Greeson 2015	Wrong study design
Griffith 2008	Wrong population: not only healtcare workers
Grigorescu 2020	Wrong study design
Gross 2018	Not an article
Guerrier 2021	Wrong study design
Gupta 2021	Wrong population: not only healthcare workers
Gutman 2020	Wrong population: students
Hansen 2006	Wrong outcome. Stressor only outcome.
Havermans 2018	Wrong intervention: Organisational intervention
Heaney 1995	Wrong intervention:organisational intervention
HemmatiMaslakpak 2016	Wrong study design
Hill 2016	Wrong study design
Hofer 2018	Wrong population: no healthcare workers
Hu 2015	Wrong study design
Johnson 2015	Wrong population: caregivers with symptoms
Jones 2000a	Wrong population: student nurses
KarbakhshRavari 2020	Wrong outcome (stressor)
Karpaviciute 2016	Wrong study design
Kesselheim 2018	Not an article
Khaghanizadeh 2008	Wrong intervention: organisational intervention
Khalsa 2021	Not an article
Kiley 2018	Wrong population: not only healthcare workers
Kloos 2019	Wrong population: not only healthcare workers
Kon 2019	Not an article
Kubota 2016	Wrong intervention
Kwok 2012	Abstract of a PhD‐thesis (not an article in a peer‐reviewed scientific journal)
Lahn 2015	Abstract of a PhD‐thesis, not an article in a peer‐review scientific journal
Lai 2011	Wrong comparison (intervention type 2 vs intervention type 2)
Lambert 2019	Not an article
Le Blanc 2007	Wrong intervention:organisational intervention
Lebares 2019	Wrong comparison (intervention type 1 vs intervention type 1)
Leiter 2011	Wrong intervention: organisational intervention
Lemaire 2011	wrong comparison (intervention type 1 vs intervention type 1)
Li 2011	Wrong intervention: organisational intervention
Lilly 2019	Wrong population: no healthcare workers
Linzer 2015	Wrong intervention ‐ organisational intervention
Loiselle 2018	Thesis (not an article in a peer‐reviewed scientific journal)
Low 2015	Wrong intervention
Lucas 2012	Wrong intervention: organisational intervention.
Lui 2019	Thesis (not an article in a peer‐reviewed scientific journal)
Luoma 2013	Wrong comparison (intervention type 1 vs intervention type 1)
Lökk 2000	Use of unvalidated questionnaire
Mahdizadeh 2019	Wrong outcome (stressor). Trial registration checked.
Manotas 2013	Thesis (not an article in a peer‐reviewed scientific journal)
McConville 2017	Wrong study design
McElligott 2003	Wrong outcome
Mellis 2019	Not an article
Meng 2018	Wrong population: students
Meyer Lamp 2020	Wrong intervention: organisational intervention
Millspaugh 2021	Wrong study design
Mistretta 2018	Not an article
Miyoshi 2019	Wrong study design
Mohebbi 2019	Wrong outcome (stressor)
Moll 2018	Wrong intervention.
Moody 2013	Duplicate
Moyle 2013	Wrong outcomes
Muller 2016	Wrong outcomes
Navidian 2019	Wrong outcome (stressor)
Nazari 2015	Wrong outcome (stressor)
NeCamp 2020	Wrong outcomes
Niva 2021	Wrong outcomes
Nourian 2021	Wrong outcomes
Ozturk 2021	Wrong population: students
Pehlivan 2019	Not an article
Penprase 2015	Wrong study design
Perula deTorres 2021	Wrong outcomes
Pich 2018	Not an article
Ploukou 2018	Wrong outcomes
Poulsen 2015	Wrong outcomes
Prasad 2018	Not an article
Procaccia 2021	Wrong outcomes
Proctor 1998	Wrong intervention:organisational intervention
Profit 2021	Wrong population: not only healthcare workers
Raglio 2020	Wrong outcome (stressor)
Rajeswari 2019	Wrong intervention
Razavi 1993	Wrong outcome
Riello 2021	Wrong outcome assessment: no use of validated questionnaire
Ripp 2019	Not an article
Rollins 2016	Wrong population: not only healthcare workers
Romig 2012	Wrong intervention: organisational intervention
Rosada 2015	Wrong study design
Rowe 2006	Wromg population:not only healthcare workers
Ruehl 2014	Thesis (not an article in a peer‐reviewed scientific journal)
Ruotsalainen 2014	Review
Ruotsalainen 2015	Review
Ruotsalainen 2016	Review
Safarzei 2016	Wrong outcomes
Saffari 2021	Wrong intervention
Salles 2013	Wrong outcome
Salyers 2019	Wrong outcome assessment: no use of validated questionnaire
Sampson 2020a	Thesis (not an article in a peer‐reviewed scientific journal)
Sargazi 2018	Wrong outcomes
Seo 2014	Wrong study design
Shaw 2021	Wrong population: not only healthcare workers
Siedsma 2015	Wrong publication type
Silva Junior 2016	Wrong study design
Smith 2019	Not an article
Smith 2021	Wrong population: students
Smoktunowicz 2021	We kindly got the mean and SD for the primary outcome from author E. Smoktunowicz. However, we retrospectively excluded this study as it includes wrong comparison (intervention type 1 vs intervention type 1)
Son 2019	Wrong population: students
Steinberg 2017	Wrong population: not only healthcare workers
Strauss 2021	Wrong population: not only healthcare workers
Taylor 2020	Wrong comparison (intervention type 2 vs intervention type 2)
Tung 2021	Thesis (not an article in a peer‐reviewed scientific journal)
Uchiyama 2013	Wrong outcomes. Trial protocol checked.
Uchiyama 2013b	Wrong intervention: organisational intervention
Valley 2017	Wrong outcomes
Valley 2017a	Wrong outcomes
van Duinen‐van den IJssel 2019	Wrong intervention:organisational intervention
vanDorssen Boog 2021	Wrong outcomes
vanLeeuwen 2021	Wrong outcomes. Study protocol checked.
Villani 2013	Wrong outcomes
Von Baeyer 1983	Wroing outcome use of unvalidated questionnaire
Watanabe 2018	Wrong intervention: pharmacological intervention
Watanabe 2019	Wrong comparison (intervention type 1 vs intervention type 1)
Watanabe 2019a	Not an article
Weitzman 2021	Wrong study design
Wilczek Ruzyczka 2021	Wrong intervention
Wild 2020	Wrong outcomes
Xu 2021	Wrong outcomes
Yamagishi 2008	Wrong intervention
Yang 2018	Wrong outcome (stressor)
Yang 2018a	Wrong population: students
Yong 2020	Wrong study design: not randomised
Zwijsen 2015	Wrong intervention: organisational intervention

### Characteristics of studies awaiting classification [ordered by study ID]

**Table:** Ahmadi 2019

Methods	?
Participants	Nurses
Interventions	Resilience education
Outcomes	Quality of working life
Notes	in Persian awaiting translation

**Table:** Akyurek 2022

Methods	Randomised control trial
Participants	Nurses
Interventions	Workplace Health Promotion Program
Outcomes	ProQol
Notes	Selected with the updated search on the 26th of September 2022.

**Table:** Bo, 2022

Methods	Randomised controlled trial
Participants	Nurses
Interventions	Mindfulness
Outcomes	MBI
Notes	In Chines awaiting translation. Selected with the updated search on the 26th of September 2022.

**Table:** Fainstad 2022

Methods	Randomised controlled trial
Participants	physicians
Interventions	Novel Online Group‐Coaching Program
Outcomes	MBI
Notes	Selected with the updated search on the 26th of September 2022.

**Table:** Fei 2019

Methods	Randomised controlled trial
Participants	Nurses
Interventions	Psychological resilience training
Outcomes	The Perceived Stress Scale, the Positive and Negative Affect Schedule & the Pittsburgh Sleep Quality Index Scale
Notes	No full text available

**Table:** Ferreres‐Galan 2022

Methods	Randomised controlled trial
Participants	Nurses
Interventions	Unified Protocol (UP) prevention program to provide emotional regulation skills to cope with stressful situations.
Outcomes	Emotional symptomatology, emotional regulation, burnout,
Notes	Selected with the updated search on the 26th of September 2022.

**Table:** Fraiman 2022

Methods	Randomised controlled trial
Participants	Paediatric Interns
Interventions	Mindfulness
Outcomes	Maslach Burnout Inventory (MBI)
Notes	Selected with the updated search on the 26th of September 2022.

**Table:** Ghods 2017

Methods	Randomised controlled trial?
Participants	Nurses
Interventions	Lavender essential oil
Outcomes	Taft Anderson job stress questionnaire
Notes	Article in Persian awaiting translation

**Table:** Goktas 2022

Methods	Randomised controlled trial
Participants	Nurses
Interventions	Motivational Messages Sent to Emergency Nurses During the COVID‐19 Pandemic
Outcomes	The Job Satisfaction Scale, Compassion Fatigue Scale, and Communication Skills Scale
Notes	Selected with the updated search on the 26th of September 2022.

**Table:** Hata 2022

Methods	Randomised controlled trial
Participants	physicians, nurse practitioners, and certified nurse midwives.
Interventions	three monthly self‐facilitated groups for faculty
Outcomes	Burnout & work stress
Notes	Selected with the updated search on the 26th of September 2022.

**Table:** Hsieh 2022

Methods	Randomised controlled trial
Participants	Nurses
Interventions	Gong medication
Outcomes	Stress & burnout
Notes	Selected with the updated search on the 26th of September 2022.

**Table:** Imamura 2019

Methods	Randomised controlled trial
Participants	Nurses
Interventions	Internet cognitive behavioural therapy (iCBT)
Outcomes	depressive and anxiety symptoms, measured by using the Depression Anxiety and Stress Scales (DASS)
Notes

**Table:** Joshi 2022

Methods	Randomised controlled trial
Participants	Various healthcare workers
Interventions	Transcendental Meditation (TM) is a mantra meditation practice with potential efficacy in reducing stress.
Outcomes	Psychological distress measured by the Global Severity Index. Secondary outcomes included changes in burnout (measured by the Maslach Burnout Inventory), insomnia (measured by the Insomnia Severity Index), and anxiety (measured by the Generalized Anxiety Disorder‐7 scale).
Notes	Selected with the updated search on the 26th of September 2022.

**Table:** Klatt 2012

Methods	Randomised controlled trial
Participants	Surgical Intensive Care Unit (SICU) personnel
Interventions	Mindfulness‐based worksite intervention
Outcomes	Depression, Anxiety and Stress Scale (DASS‐21)
Notes	No full text available.

**Table:** Klatt 2022

Methods	Randomised controlled trial
Participants	Healthcare workers (not specified)
Interventions	Mindfulness Based Intervention (MBI), Mindfulness in Motion (MIM),
Outcomes	Burnout & perceived stress
Notes	Selected with the updated search on the 26th of September 2022.

**Table:** Lu 2020

Methods	Randomised controlled trial
Participants	pediatric nurses
Interventions	Balint group
Outcomes	MBI
Notes	Article in Chinese awaiting translation

**Table:** Montaner 2022

Methods	Randomised controlled trial
Participants	Dementia healthcare workers
Interventions	Acceptance and Commitment Therapy (ACT)
Outcomes	MBI
Notes	Selected with the updated search on the 26th of September 2022.

**Table:** Moss 2022

Methods	Randomised controlled trial
Participants	Healthcare Professionals
Interventions	Creative Arts Therapy
Outcomes	Burnout
Notes	Selected with the updated search on the 26th of September 2022.

**Table:** Perez 2022

Methods	Randomised controlled trial
Participants	Nurses
Interventions	Mindfulness‐Based Intervention
Outcomes	ProQol
Notes	Selected with the updated search on the 26th of September 2022.

**Table:** Purdie 2022

Methods	Randomised controlled trial
Participants	Resident Physicians
Interventions	Hybrid Mindful Awareness Practices (
Outcomes	MBI
Notes	Selected with the updated search on the 26th of September 2022.

**Table:** Rogala 2016

Methods	Randomised controlled trial
Participants	Professionals working with trauma survivors
Interventions	web‐based intervention, “The Helpers’ Stress”
Outcomes	MBI
Notes	In Polish awaiting translaten

**Table:** Sasaki 2021

Methods	Randomised controlled trial
Participants	Nurses
Interventions	cognitive behavioral therapy
Outcomes	Utrecht Work Engagement Scale–9 item (UWES‐9)
Notes	Trial protocol checked bmjopen‐2018‐025138. In the trial protocol DASS stress is one of the outcomes but not reported in this article.

**Table:** Spilg 2022

Methods	Randomised controlled trial
Participants	Physicians
Interventions	The Stress Management and Resilience Training (SMART) program is an evidence‐based intervention designed to build resilience.
Outcomes	Stress
Notes	Selected with the updated search on the 26th of September 2022.

**Table:** Taft 2021

Methods	Randomised controlled trial
Participants	Cardiac surgery nurses
Interventions	Educational intervention
Outcomes	Nursing Stress Scale (NSS)
Notes	Article in Persian awaiting translation

**Table:** Taylor 2022

Methods	Randomised controlled trial
Participants	Healthcare workers
Interventions	Unguided, digital, mindfulness‐based self‐help (MBSH)
Outcomes	MBI
Notes	Selected with the updated search on the 26th of September 2022.

**Table:** Vajpeyee 2022

Methods	Randomised controlled trial
Participants	Healthcare workers
Interventions	Yoga and music intervention
Outcomes	Stress
Notes	Selected with the updated search on the 26th of September 2022.

**Table:** Valipour 2020

Methods	Randomised controlled trial
Participants	Nurses
Interventions	stress management training
Outcomes	The Job Stress Questionnaire
Notes	No full text available

**Table:** Xiao Yan 2019

Methods	?
Participants	Psychiatric nurses
Interventions	drum circle activity combined with the psychological diary technique
Outcomes	stress
Notes	Article in Chinese awaiting translation

### Characteristics of ongoing studies [ordered by study ID]

**Table:** Al‐Hammouri 2022

Study name	Al‐Hammouri
Methods	Randomised controlled trial
Participants	Inclusion criteria: 1) Jordanian nurses 2) at least one‐year experience in the workplace setting 3) don’t have previous experience with mindfulness meditation
Interventions	The intervention protocol is similar to the brief mindfulness‐based stress reduction program designed by Mackenzie (2006). He synthesises its main elements from Kabat‐Zinn's (1990) traditional mindfulness‐based stress reduction program. The participants will be recruited from nurses inside the hospital for the two intervention groups so that the 65 nurses will receive the mindfulness meditation session inside one of the hospital rooms equipped to deliver the intervention, and the second group of the 65 nurses who will receive the intervention in a natural setting outside the hospital (i.e. park or recreation centre, that will be decided based on the funding to be determined after getting the IRB approval). Intervention groups will practice mindfulness meditation once weekly for 4 weeks as a group (i.e. 4 sessions for each of the two interventional groups). There are no recommendations about group size in the literature, but we will use small group sizes between 10 and 20 participants in each session for convenience. Mindfulness‐based intervention trainers will guide the mindfulness meditation sessions, and each session is 30 minutes long. The intervention comprises four 30‐minute training sessions that cover the following topics: an introduction to mindfulness, typical barriers to practice, the repercussions of attachment and aversion to judging events, and methods for bringing mindfulness into one's daily life. The body scan, sitting meditation, and a brief three‐minute breathing exercise for use in times of acute stress were all experienced components of the sessions (Mackenzie et al., 2006; Poulin et al. 2008). In addition, the participants in the intervention groups will be instructed to practice mindfulness mediation individually at home for at least 10 minutes
Outcomes	Stress Overload Scale Depression will be measured using the Center for Epidemiologic Studies Depression Scale‐Revised (CESD‐R).
Starting date	31/05/2022
Contact information	Jordan University of Science & Technology P.O.Box 3030, Irbid 22110, Jordan Jordan mmalhammouri@just.edu.jo
Notes

**Table:** Baker 2015

Study name	NR
Methods	Randomised controlled trial
Participants	Healthcare workers of care homes
Interventions	Mindfulness‐based stress reduction (MBSR)
Outcomes	The Work Stress Inventory The Karasek Job Content Questionnaire
Starting date	?
Contact information	
Notes

**Table:** Bateman 2020

Study name	STOPTHEBURN
Methods	Randomised controlled Trial
Participants	ICU clinicians & non‐physicians (nurses, pharmacists, therapists).
Interventions	debriefing
Outcomes	Maslach Burnout Inventory (MBI) Score. Patient Health Questionnaire 8 (PHQ‐8) and Generalized Anxiety Disorder 7‐item scale (GAD‐7).
Starting date	?
Contact information	
Notes

**Table:** Bratt 2022

Study name	NR
Methods	Randomised controlled trial
Participants	Healthcare professionals
Interventions	Compassion course A cognitive behavioural stress management course
Outcomes	COPSOQ / PROQOL
Starting date	February 2021
Contact information	Anna Bratt, Linnaeus University anna.bratt@lnu.se
Notes

**Table:** Jeffers 2017

Study name	Jeffers
Methods	Randomised controlled trial
Participants	Nurse currently rostered to work in the RBWH DEM Able to attend program
Interventions	A face‐to‐face self‐care intervention program designed and supervised by an accredited clinical psychologist with over 10 years of experience. Participants will be offered one of two weekly hospital based sessions of approximately 1 hour duration for 5 weeks (10 participants should be in each of the sessions) with a home program.
Outcomes	Stress will be measured using the Depression, Anxiety and Stress Scale‐21 Professional Quality of Life will be measured using the Professional Quality of Life Scale, version 5 (Pro‐QOL‐5)
Starting date	23/10/2017
Contact information	Ms Carol Jeffers Butter field Street Royal Brisbane and Women's Hospital Herston QLD 4029 James.Hughes@health.qld.gov.au
Notes

**Table:** Kuribayashi 2019

Study name	NR
Methods	Randomised controlled trial
Participants	Nurses
Interventions	Internet‐based Cognitive Behavioral Therapy (iCBT)
Outcomes	Psychological distress
Starting date	?
Contact information	kkuribayashi‐jans@umin.ac.jp
Notes

**Table:** Ng 2019

Study name	Brief Daily Body‐Mind‐Spirit Practice
Methods	Multi‐site randomised controlled trial
Participants	Community Mental Health Workers
Interventions	Brief Daily Body‐Mind‐Spirit Practice
Outcomes	Copenhagen Burnout Inventory
Starting date	?
Contact information	SM Ng Department of Social Work and Social Administration, The University of Hong Kong, Pokfulam, Hong Kong
Notes

**Table:** Pérula‐de Torres 2019

Study name	MINDUUDD
Methods	Multicentre cluster‐randomised controlled trial
Participants	Community medicine physicians and nurses
Interventions	Mindfulness and self‐compassion 4‐session programme versus an 8‐session programme
Outcomes	
Starting date	June 2019
Contact information	luisangel.perula@gmail.com
Notes

**Table:** Rees 2018

Study name	Mindful Self‐Care and Resiliency (MSCR)
Methods	Randomised controlled trial
Participants	Rural general practitioners
Interventions	Mindful Self‐Care and Resiliency (MSCR)
Outcomes	Compassion fatigue?
Starting date	?
Contact information	C.Rees@curtin.edu.au
Notes

**Table:** Weiner 2020

Study name	REST
Methods	Randomised controlled trial
Participants	Healthcare workers during the COVID‐19 pandemic
Interventions	Online cognitive behavioural therapy program
Outcomes	Perceived Stress Scale (PSS)
Starting date	September 2021
Contact information	weiner.l@gmail.com
Notes	

**PSQ:** Perceived Stress Questionnaire

## Differences between protocol and review

In the previous version of this review titled "Preventing occupational stress in healthcare workers" (Ruotsalainen 2015) interventions were categorised into cognitive‐behavioural, mental and physical relaxation and organisational‐level interventions. We excluded interventions solely targeting organisational‐level stressors, such as work‐demands, variation at work, because they are covered by the Giga 2018 review. We think the new categorisation into focusing on the experience of stress, focusing away from the experience of stress, focusing on individual work‐related risk factors and any combination of these is more informative.

In the previous version of this review (Ruotsalainen 2015) stressors were pooled with stress symptoms. For example, a stressor such as the reward imbalance questionnaire or the Nursing Stress Scale was pooled with stress symptom questionnaires such as the Perceived Stress Scale (PSS)or the Maslach Burnout Inventory (MBI). We are of the opinion, that stressors and stress symptoms cannot be pooled in one meta‐analysis because these are two different constructs (van der Molen 2020). Stressor questionnaires measure organisational‐level risk factors such as, job demands, jobs control, workplace social support, whereas stress symptoms questionnaires such as the PSS focus on the individual's experience of stress (e.g. have you been angered because of things that were outside your control?) (Cohen 1983). Since we have excluded organisational‐level interventions, stressor questionnaires were also excluded.  

In the previous version of this review (Ruotsalainen 2015) the review authors reported secondary outcomes of physiological parameters such as hair cortisol. We excluded these secondary outcomes as we think that these parameters cannot be interpreted, because there is no consensus on the validation of stress‐related physiological parameters (Schaafsma 2021).  

In the previous version of this review (Ruotsalainen 2015) subgroup analyses were performed for type of healthcare worker. Since interventions targeting organisational‐level interventions were excluded because they are covered by the Giga 2018 review, we did not perform subgroup analysis by type of healthcare worker. We think that the individual‐level interventions work the same way for various healthcare workers (e.g. physicians, nurses), which makes this type of subgroup analysis redundant. We discussed the proposed subgroup analyses based on the duration and intensity of the intervention as stated in the original protocol (Marine 2000). However, no definitions were formulated a priori, and we discussed what a proper grouping would be. We concluded that dividing the studies in shorter or longer and intense or less intense interventions would be an arbitrary ‐ and possibly data driven ‐ approach. Moreover, such a grouping would ideally be based on a mixture of the duration and intensity of the intervention (e.g. number of sessions, the length of the sessions, homework assignments) and the compliance with the intervention. However, we explored whether the arbitrary cut‐off for duration of the intervention of 12 weeks shows an effect in effect size. 

De Wijn (de Wijn 2022) found that stress management interventions for nurses in which the sample was exposed to the majority of the planned sessions reached greater effect sizes compared to interventions in which the compliance to the intervention/attendance to the planned sessions was lower. Although this finding should be interpreted with caution due to a lot of missing data, we explored if the effect sizes based on studies in which participants attended 80% or more of the scheduled sessions would differ from the studies were participants attended less than 80% of the scheduled sessions de Wijn 2022. 

In the previous version of this review (Ruotsalainen 2015), the objective was formulated as: 'to evaluate the effectiveness of work‐ and person‐directed interventions compared to no intervention or alternative interventions in preventing stress at work in healthcare workers.' Since, healthcare workers have higher levels of stress compared to the general population we think that 'reduce stress' better describes what we want to study than 'preventing stress at work'. In our opinion, this is not really a difference between protocol and review, rather a semantic difference. 

In the previous version of this review (Ruotsalainen 2015), the most intensive intervention was included in the analyses in the case of multiple arms. As recommended by the Cochrane Handbook, we have now combined groups to create a single pair‐wise comparison. 

In the previous version of this review (Ruotsalainen 2015), the time frames of the measurement of outcomes were: (i) up to one month (ii) from one month to six months (iii) over six months. We have changed these time frames into short‐term defined as up to and including three months after the intervention has been completed, medium term defined as more than three months up to 12 months, and long term defined as 12 months or longer. The rationale here was to focus more on the long‐term effects.  

In the previous version of this review (Ruotsalainen 2015), one missing standard deviation (SD) was imputed. We have now excluded studies from the meta‐analyses if missing SDs could not be provided by study authors or when they could not be calculated from the available data. 
